# Supplementary material for: Evolutionarily stable gene clusters shed light on the common grounds of pathogenicity in the Acinetobacter calcoaceticus-baumannii complex
Source: PLoS Genet. 2022 Jun 2;18(6):e1010020. doi: 10.1371/journal.pgen.1010020 (PMC9162365; doi:10.1371/journal.pgen.1010020)
Supplement: S1 Text — Contains supplemental sections covering additional information on the taxon set compilation, a statistical exploration of all protein-coding genes, genomes and orthologs in Set-R, details on the method and workflow of the ESGC prediction, and provides additional results and discussions for the predicted ESGCsACB including several clusters not discussed in the main manuscript. (DOCX) [file pgen.1010020.s001.docx]

Table of Contents

[Taxon Set Construction (Set-R) 2](#_Toc89037201)

[Identification of low-quality assemblies 2](#_Toc89037202)

[Phylogenetic diversity analysis to increase taxonomic diversity of Set-R sampled from Set-F 2](#_Toc89037203)

[Qualitative and Quantitative Gene Set Analyses 3](#_Toc89037204)

[Overview of genome assemblies in Set-R 3](#_Toc89037205)

[Pan-genome reconstruction 4](#_Toc89037206)

[Core-genome reconstruction 5](#_Toc89037207)

[Evolutionary Stable Genomic Clusters (ESGCs) 8](#_Toc89037208)

[Workflow of ESGC identification and shortlisting 8](#_Toc89037209)

[ESGC_ACB_-0162 (quorum sensing and biofilm formation) 9](#_Toc89037210)

[Characterization of AbaM 10](#_Toc89037211)

[Integration of the QSLux system with the NRPS cluster 13](#_Toc89037212)

[ESGC_ACB_-0368 and 0369 (Acinetobactin) 14](#_Toc89037213)

[ESGC_ACB_-0498 (Enterobactin) 14](#_Toc89037214)

[ESGC_ACB_-0016 (Carnitine) 14](#_Toc89037215)

[ESGC_ACB_-0497 (PQQ biosynthesis) 15](#_Toc89037216)

[Gene clusters (ESGCs) not discussed in main manuscript 15](#_Toc89037217)

[ESGC_ACB_-0622 (Phenylacetate metabolism) 15](#_Toc89037218)

[ESGC_ACB_-0078 (Tricarballylate metabolism) 16](#_Toc89037219)

[ESGC_ACB_ -0627 (2-aminoethylphosphonate metabolism) 17](#_Toc89037220)

[ESGC_ACB_-0372 (Xanthine metabolism) 17](#_Toc89037221)

[ESGC_ACB_-0555 (Taurine metabolism) 18](#_Toc89037222)

# Taxon Set Construction (Set-R)

The full data set (Set-F) comprises all assemblies in the NCBI RefSeq data base (version 87, accessed March 13, 2018) stating ‘Acinetobacter’ in the ‘organism’ field. Set-R is a subset of Set-F and comprises hand-picked strains of *A. baumannii,* representative genomes of all known species as well as well as genomes of sufficient quality that increase total phylogenetic diversity covered.

## Identification of low-quality assemblies

To identify low-quality assemblies in Set-F, we analyzed the distributions of three variables: two contiguity measures, i.e. the number contigs larger than 1000 bp and the N50, and one completeness measure: the BUSCO completeness score using the Gamma-Proteobacteria odb10 data set. We removed the entry ‘POG090900BT‘ from the BUSCO set because it was absent in all *Acinetobacter* genomes analyzed by us. To reduce bias introduced through scaffolded assemblies we broke scaffolds at their joints. BUSCO v3 [1] was executed with the following set of parameters: ‘-i geneset.fa -m prot -l ./dbs/busco/gammaproteobacteria_odb9’. Finally, assemblies were labelled as low-quality if any of the following three conditions were met: i) number of contigs > 97.5^th^ percentile, ii) N50 <= 2.5^th^ percentile, and iii) %complete BUSCO genes < 2.5^th^ percentile.

## Phylogenetic diversity analysis to increase taxonomic diversity of Set-R sampled from Set-F

The representative set of *Acinetobacter* genomes, Set-R, was compiled as a trade-off between sample size (limited by computational resources), the relevance of strains in the literature, the genome assembly quality, and the phylogenetic diversity of the genus covered. We considered all available type, reference and representative genomes, as well as all validly named species for which a genome sequence was available at the study onset. We further picked genomes of several *A. baumannii* strains that are of interest due to e.g., their context of isolation as well as representatives of eight international clone types. We subsequently added further taxa to increase the phylogenetic diversity of Set-R to reach the final count of 232. Phylogenetic diversity scores of SET-F and SET-R were computed based on the ML tree of SET-F (see below) using PDA v.1.0.3 [2] and the command ‘-k=234:3027 -if=handpicked.list‘. Low quality assemblies were excluded from this analysis.


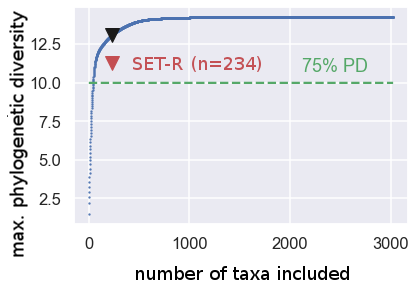


**Fig A**: Incremental maximum phylogenetic diversity (pd). The distribution is shown as a stepwise increase in taxon sample size k from 0 to 3052 (total number of taxa in Set-F). Pd is calculated based on the branch lengths in the maximum likelihood tree for Set-F. Pd saturates at around 1000 taxa. The total pd reaches a value of ~14.26 of which we cover ~80% (11.26) with Set-R. The optimum pd for k=232 taxa (black triangle) is 13.0

The final Set-R covers 79% from the total phylogenetic diversity of the 3052 taxa in Set-F. We did not aim to reach the maximally possible phylogenetic diversity (cf. Fig A), because this would have required to exchange taxa from our focal group, the considerably closely related *Acinetobacter* *calcoaceticus-baumannii* complex, with more distantly related taxa, or alternatively to substantially increase the size of Set-R. While the latter would have been possible, in principle, it would not have added to the message of this manuscript.

During data collection, we observed non-negligible differences in genome length and number of predicted coding sequences (CDS) among existing assemblies of ATCC 19606. Due to its important role as a reference in many studies, we decided to analyze three different assemblies (GCF_00369385.1, 4.02 Mbp with 3763 CDS; GCF_000737145.1, 3.95 Mbp with 3732 CDS; and GCF_900011295.1, 3.97 Mbp, 3854 CDS) in parallel. However, in all summary statistics, we collapsed the information and treated the three taxa as one. For cluster identification and microsynteny analysis, we used GCF_000737145.1 as a reference since it represents the most contiguous assembly among them.

# Qualitative and Quantitative Gene Set Analyses

## Overview of genome assemblies in Set-R

Current assembly status varies across Set-R. N50 values range from 34kbp for the assembly of *A. haemolyticus* TG21157 (assembly status “contigs”) to 4.3Mb (assembly status “complete genome”) for the assembly of *A. lwoffii* TCC 9957. The average (median) *Acinetobacter* genome in Set-R contains 3566 (IQR: 600) CDS of which 3415 (IQR: 590) are single-copy. The smallest genome (2497 CDS) belongs to *A. boissieri* ANC 4422 isolated from floral nectar, while *A. bereziniae* LMG 1003 was isolated approximately 60 years ago from a human wound sample is the largest (4688 CDS). To assess whether differences in contiguity and gene number is likely to affect downstream gene repertoire analyses, we tested each genome for its gene set completeness. For this purpose, we used a set of 451 universal, single-copy orthologs found in gamma-proteobacteria (BUSCO genes). The median of missing BUSCO genes is 1 with an IQR of 3. However, for 3 genomes the proportion of missing BUSCO genes was larger than 10% (*A. baumannii* S46, *A. baumannii* XH743, and XH729). This goes along with a reduced total genome size (-4 to -13% in comparison to ATCC 19606 assembly GCF_000737145.1) and a reduced number of predicted CDS (-2 to -11%). Further analyses will have to reveal whether these findings reflect genuine evolutionary events or an artifact due to low-quality data. In favor of an evolutionary explanation is the fact that the assembly statistics for these genomes are within normal limits. For instance, their N50 sizes range from 132kbp to 193kbp, which is > 25^th^ percentile (131kbp) in Set-R. Despite these outliers, we conclude that gene repertoire analyses will not be impacted by the variance in assembly quality status.

## Pan-genome reconstruction

To assess trends in gene content dynamics, we inferred and extrapolated pan-genome sizes of Set-R based on the orthologous relations of the proteins. The resulting pan-genome comprises 22,350 orthologous groups (OGs) harboring 783,306 proteins. 16,000 proteins remained singletons. 889 genes represent the core-genome of *Acinetobacter* *spp*. Based on the species tree that was reconstructed from the three non-overlapping partitions of the core-genome, we inferred 14293 Hierarchical orthologous groups (HOGs) across the 232 taxa.

Ten percent of the Set-R pan-genome is confined to the three most recently diverged clades on the lineage from the last common ancestor of the genus towards *A. baumannii* (*cf*. Fig 3A in the main text). In turn, about half of the genes are not represented in *A. baumannii*. A rarefaction analysis indicated an open pan genome size of the genus *Acinetobacter* (growth exponent: -0.42 ± 5e-4 at 95% confidence; fit (r2=99.9)). Similarly, Set-R also demonstrates open pan-genomes for the strains of the ACB clade as well as for *A. baumannii* exclusively (Fig B). The results were robust when stratifying Set-R to one (randomly chosen) strain per species. This indicates that throughout the genus, genome content is highly dynamic and therefore it is not an exclusive feature of the virulent *Acinetobacter* species. Consequently, linking phenotype and genotype by binary presence/absence-based analyses of only few focal genes likely yields sparse phyletic patterns.

The pan genome reconstruction further revealed that 10% of the pan-genome is confined to the three most recently diverged lineages (ACB+HA: 554 genes; ACB: 580 genes; *A. baumannii*: 307 genes). In turn, about half of the Set-R pan-genome is not represented in A. baumannii (cumulative numbers - ACB: -5629 genes; NO+S+B: -6855; B: -7794).


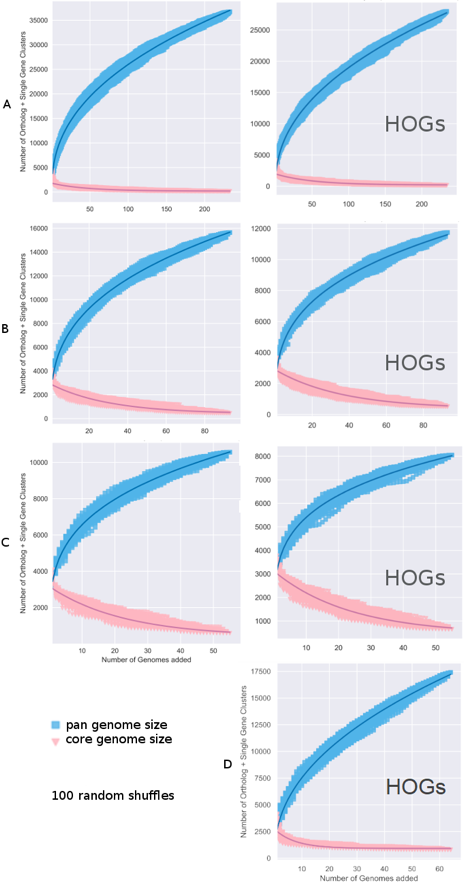


**Fig B**. **Rarefaction analysis of the pan- (blue) and core-genome size (red) through the sequential addition of genomes.** Point estimates of the sizes are represented as dots while the fitted curves are shown as solid lines. Each rarefaction analysis was repeated 100 times with random permutations of the order in which genomes were added. In A the analysis considered the taxa of the entire genus Acinetobacter (n=232), in B the taxa of the ACB clade (91), and in C the genomes of Acinetobacter baumannii (n=53). The left column provides pan- and core-genome estimates based on the stricter grouping of genes as cliques of orthologs (OMA orthologous groups, see Methods). Plots in the right column are based on hierarchical orthologous groups (HOGs). D Depicts a species-stratified version of A. Here, each consecutively added genome is a randomly drawn strain of each named species including one random strain drawn from the collection of all unnamed species (sp.) in Set-R (n=64).

## Core-genome reconstruction

For the phylogenetic reconstruction of Set-R and Set-F, we chose a core-genome-based supermatrix approach. The core-genome was defined as the collection of orthologous groups (OGs) that harbors one representative of each taxon in the respective taxonomic scope. Analogously, we defined the core-genome over the hierarchical orthologous groups (HOGs) as the subset of HOGs with exactly one representative of each organism in Set-R. Given the above definition, a strict interpretation and extrapolation by fitting the exponential decay curve (see equation A) yielded very low estimations of the core size Ω (Table A). This is most likely an underestimation as a consequence of including draft genome sequences with varying genome assembly quality and assembly completeness. To accommodate for the draft status of many genomes, we slightly relaxed the required abundance of a gene from 100% to 99% of the taxa. In other words, we required each core-OG to contain orthologs from at least 231 out of the 232 strains. Note, this threshold is based on the mean percent completeness of the BUSCO v3 protein set specific to Gamma-proteobacteria (*cf.* S2 Table). This resulted in 889 genus core-genes. Note, the value is closest to the Ω calculated by the extrapolation the core-genome size using only one randomly chosen representative per strain (see Table A: HOG genus stratified), which indicates that indeed few genomes impaired the strict core-genome assessment. Among these is *A. baumannii* S46. This strain has a particularly small genome size (3.4Mbp), which is about 12 % smaller compared to the species type strain. 154 of the 889 core genes are missing in this genome. Although the assembly and gene annotation for this strain passed all automated quality checks to have it included into NCBI RefSeq Genome and into our study, a manual curation of assembly quality including mapping of the shotgun reads could still reveal that the deviating statistics are due to undetected errors in the assembly. Further, we found 2090 core genes for the ACB clade and 2176 for the set of all *A. baumannii* genomes.

*F* = κ exp[–*n*/τ] +Ω ,

**Equation A**: Exponential Decay function with n representing the number of taxa added and κ, τ and Ω being free parameters, leads to the following results (cf. Fig B). The free parameter Ω indicates the extrapolated core-genome size at OG and HOG levels, respectively. The estimated parameters after fitting are summarized in Table A.

**Table A. Estimated parameters after fitting the exponential decay function for core-genome extrapolation and the power law function for pan-genome extrapolation**.

| Orthology inference | Taxonomic level | #taxa | Pan-genome extrapolation function: *F = k * n^-α^* | | | Core-genome extrapolation function*: F* = κ exp[–*n*/τ] +Ω | | | |
| --- | --- | --- | --- | --- | --- | --- | --- | --- | --- |
|  |  | n | k | α | r^2^ | κ | τ | Ω | r^2^ |
| OG | genus | 234 | 3781.35 ±9.21 | -0.42 ±0.00 | 0.99 | 1609.38 ±9.21 | 46.12 ±0.56 | 194.31 ±3.88 | 0.86 |
| HOG | genus | 234 | 2421.63 ±6.44 | -0.45 ±0.00 | 0.99 | 1758.93 | 50.10 ±0.51 | 200.98 ±3.77 | 0.91 |
|  |  |  |  |  |  |  |  |  |  |
| OG | ACB clade | 93 | 3316.90 ±9.30 | -0.34 ±0.00 | 0.99 | 2497.33 ±14.398 | 30.38 ±0.54 | 402.11 ±13.21 | 0.93 |
| HOG | ACB clade | 93 | 2933.19 ±8.98 | -0.30 ±0.00 | 0.99 | 2525.64 ±13.54 | 36.27 ±0.63 | 362.46 ±15.24 | 0.94 |
|  |  |  |  |  |  |  |  |  |  |
| OG | *baumannii* | 55 | 3444.11 ±12.15 | -0.28 ±0.00 | 0.99 | 2768.69 ±23.03 | 22.68 ±0.62 | 397.15 ±27.13 | 0.93 |
| HOG | *baumannii* | 55 | 3166.47 ±9.90 | -0.23 ±0.00 | 0.98 | 2674.84 ±21.15 | 23.60 ±0.61 | 443.67 ±25.47 | 0.94 |
|  |  |  |  |  |  |  |  |  |  |
| HOG | genus (stratified) | 64 | 2701.41 ±12.45 | -0.45 ±0.0 | 0.99 | 1804.12 ±26.37 | 7.56 ±0.18 | 916.13 ±5.42 | 0.83 |

# Evolutionary Stable Genomic Clusters (ESGCs)

## Workflow of ESGC identification and shortlisting


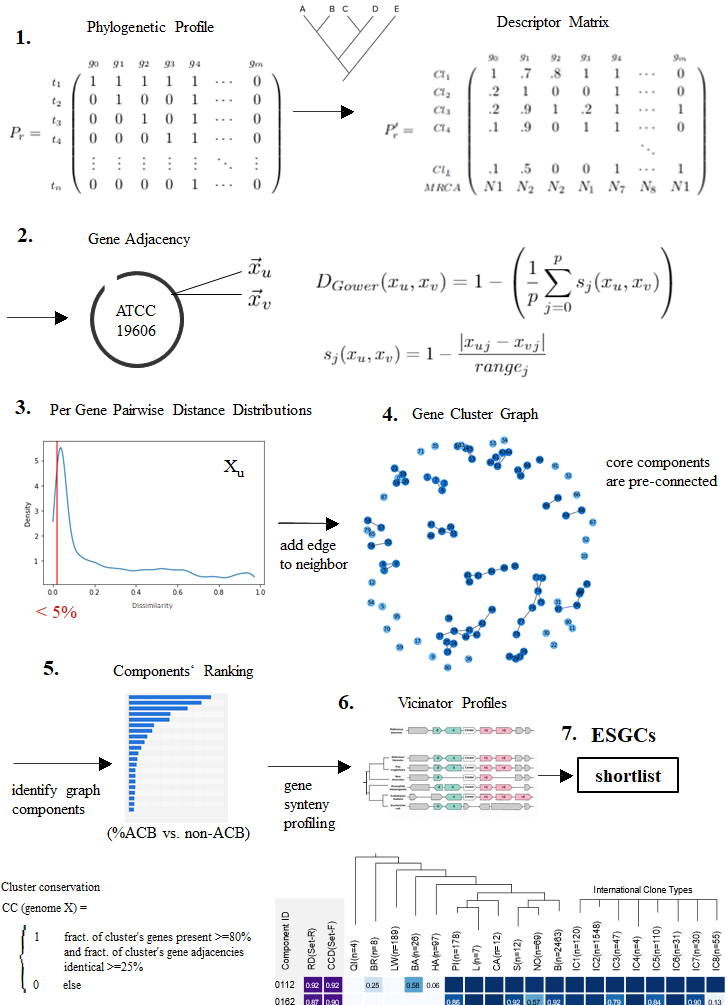


**Fig C. Workflow of ESGC identification**. Based on the phyletic profiles of every ortholog across the pan-genome, we generated 1) a descriptor matrix that transforms binary values into fractions representing relative abundance across a specific clade. The vector of clade-wise fractions extended by the associated inner node label was then used to 2) annotate each gene along the genome of the A. baumannii type strain ATCC 19606. From the genome, we obtained an adjacency matrix, which we used to calculate the Gower distance of each vector to every remaining gene on the replicon (contig). 3) We used the 5^th^ percentile as the threshold that determines whether the profile similarity of the downstream gene x_v_ is greater than expected by chance to the preceding gene x_u_. Next, 4) a directed graph G(V,E) was constructed with V=set of genes in a replicon is generated we first added edges for all neighboring gene that were identified as core-genes (core-genome). We next added an edge between two neighboring genes (x_u_,x_v_) if the vector dissimilarity between the genes (x_u_,x_v_) or (x_u_, x_w_) was below the 5^th^ percentile threshold of x_u_. This process is subsequently repeated in reverse with the percentile threshold of x_v_ and x_w_, respectively. Lastly, we extracted all connected components in the graph with as candidate ESGCs. Components were ranked by relative abundance difference inside versus outside the ACB clade (RD). We approximated the presence/absence of the cluster in a genome using Vicinator tool’s output. A cluster is considered present in a genome if at least 80% of the genes and 25% of the set of all gene pairings of the cluster are conserved (CC). For the final list of the top ranked 150 ESGCs_ACB_ we removed a cluster from the ranked list if the relative cluster conservation difference between all ACB genomes minus all non-ACB genomes was below the cutoff (<0.25) that suggests an unstable or ACB-clade-unspecific cluster.

The workflow of the ESGC identification is described in detail in Fig C. As a reference genome, we used *Ab* ATCC 19606, but the procedure can be applied to any strain as the reference. The *Ab* ATCC 19606 reference assembly, GCF_000737145.1, comprises two scaffolds, likely representing two independent replicons: the chromosome (NZ_KL810966.1) and a plasmid (NZ_KL810967.1), for which edge-introduction (step 4) ran separately. 1370 graph components were identified with 634 of size greater than 1. Note, per definition a connected component can also be of size 1 as there is a connection to itself with path length 0. The size distribution of the connected components is bounded by a maximum of 35 with most clusters comprising between 2 and 5 genes (Fig D).


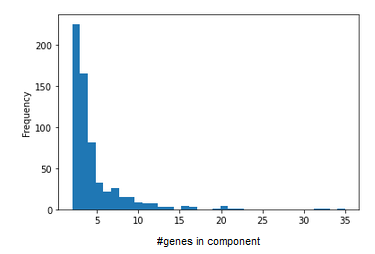


**Fig D. Histogram of the number of genes per connected component comprising two or more genes.**

## ESGC_ACB_-0162 (quorum sensing and biofilm formation)

Persistence in the hospital environment is a hallmark trait of nosocomial pathogens. *A. baumannii* uses a diverse set of factors to form stable biofilms, i.e. bacterial communities encased in an extracellular matrix, on both host tissues and abiotic surfaces, and even on stainless steel. In fact, the colonization of patient-indwelling devices, such as ventilators or urinary catheters, is one of the main causes of *A. baumannii* infections [3]. Biofilms on abiotic surfaces are solely mediated by type I pili formed via the ancient chaperone usher (Csu) pathway [4, 5]. Factors mediating attachment and interaction with the host tissue are substantially more diverse and include BAP [6], OmpA , ATA [7], and CUP [3]. Biofilm formation is a tightly regulated process in *A. baumannii*. It involves at least two different 2-component systems, BfmRS [8] and GacSA [9], controlling the expression of the Csu cluster. Moreover, it is intimately connected to quorum sensing mediated via the AbaI/R system. It appears that a small lipoprotein, Ac-505, is also involved in orchestrating biofilm formation [10]. The phylogenetic profiles of the involved gene clusters reveal that most of this network is restricted to the ACB clade (see main text, Figs 5 and 7A).

Quorum sensing (QS) is central to bacterial physiology, and it is a main determinant of bacterial virulence [11]. Notably, QS is one of only three biological processes we found significantly enriched in the part of the *Acinetobacter* pan-genome that is private to the ACB clade (cf. Table 1 in the main manuscript). The corresponding ESGC_ACB_ 0162 includes an N-acylhomoserine lactone synthase (AbaI), and a transcriptional regulator (AbaR) jointly establishing a Lux-type QS system (QS_Lux_). QS_Lux_ has been shown to be involved in the regulation of motility, biofilm formation and virulence in *A. baumannii* [12-14]. Our results indicate that this type of quorum sensing is central to these processes for all members of the ACB clade, but not in *Acinetobacter* species outside this clade. AbaR and AbaI are separated by a gene encoding a short polypeptide, here tentatively named AbaM, which form a triad $\left( R\vec{M}\vec{I} \right)$ (arrows indicate orientation). AbaM harbors a single Pfam domain of unknown function, DUF4902 (PF16245).

### Characterization of AbaM

We first determined in which species outside the *Acinetobacter sp.* AbaM is present (S14 Table). Given the short length of AbaM, ortholog searches are prone to miss this protein [15], in particular in species distantly related to *A. baumannii*. We therefore increased the search sensitivity and used the presence of this Pfam domain as a proxy of the presence of this protein (Fig E). This revealed that AbaM is rare in the bacterial domain. Only 50 species (56 sequences) in the entire Pfam Database were found to harbor a sequence with the DUF4902 domain (<http://pfam.xfam.org/family/DUF4902>). All 56 sequences are short and their domain architecture consists only of the DUF4902 domain. The majority of taxa belong to the Burkholderiales, among them the human pathogen *Burkholderia pseudomallei*. In contrast, 801 species were found harboring the 1224 sequences with the Autoind_synth Pfam domain (PF00765) characteristic of AbaI (LuxI) ([http://pfam.xfam.org/family/PF00765.17](http://pfam.xfam.org/family/PF00765.17L); Fig E). Lastly, 1133 taxa harbor a total of 2796 sequences with the Autoind_bind Pfam domain (PF03472; <http://pfam.xfam.org/family/PF03472.15>), which is characteristic of AbaR (LuxR). Consulting the OMA database, that provides next to orthology assignments also information about gene order, reveals that only 5 orthologs were found for the AbaM of *A. baumannii* AYE AbaM outside the genus *Acinetobacter*. In all cases, the presence of AbaM conditions the presence of AbaR and AbaI in conserved gene order, but not vice versa (Fig F).

In *Burkholderia*, the genus with the highest prevalence of AbaM homologs in our analysis (see Fig E), QS_Lux_ cluster with the same $\left( R\vec{M}\vec{I} \right)$ organization as it is seen for the ACB clade are prevalent [16]. In this genus, the intervening gene is annotated as a homolog to *rsaM* in the plant pathogen *Pseudomonas fuscovaginae*, a major negative regulator of both AHL biosynthesis and expression of AHL synthase-coding genes in this species [17]. In *Burkholderia thailandensis*, a close relative to the human pathogen *B. pseudomallei*, the rsaM-like gene acts as a negative regulator of the QS_Lux_ system [18]. In line with this finding, Lopez-Martin et al. recently provided initial evidence that in *A. baumannii* Ab5075 AbaM plays a role in the regulation of virulence-related processes in [19]. Integrating all evidences, it is likely that AbaM plays an essential role in the regulatory network controlling motility, biofilm formation, and ultimately virulence in all members of the ACB clade.

| **A** | **B** |
| --- | --- |
| 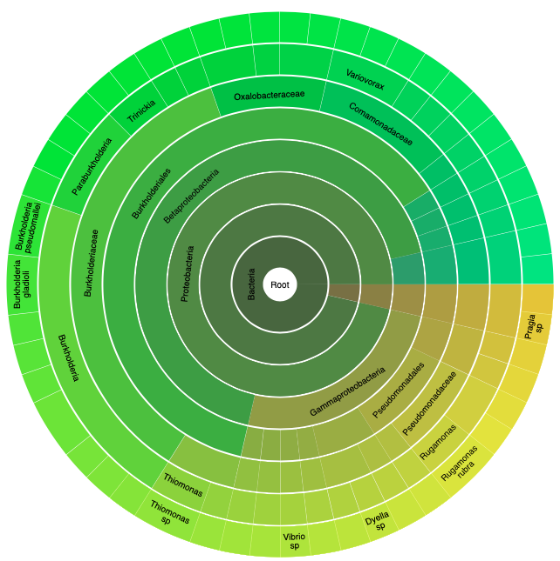 | 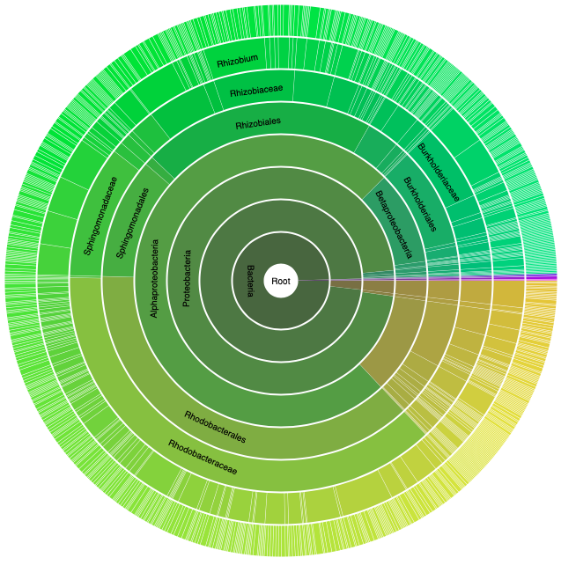 |
| **C** |  |
| 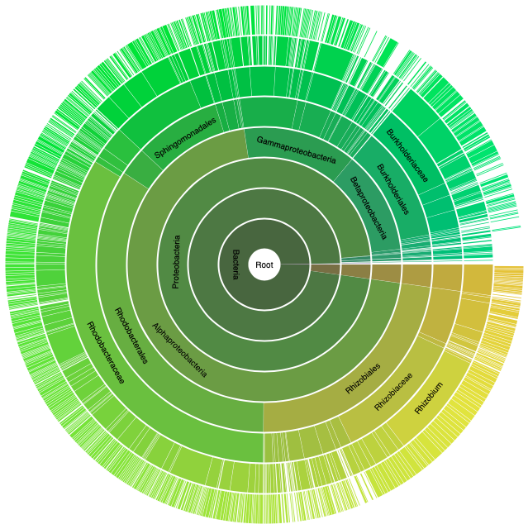 | |

**Fig E**. **The sunburst plot reveals the abundance of the PFAM domains characteristic for the three genes comprising the QS_Lux_ cluster of A. baumannii ATCC 19606 across all sequences in the Pfam database.** A) AbaM - DUF4902 (56 sequences in 50 species), B) AbaI - PF00765 (1224 sequences in 801 species). C) AbaR - PF03472 (2796 sequences in 1133 species). With very few exceptions, the domains are present only in the proteobacteria. Numbers according to Pfam v.33.1 (May 2020, 18259 entries; accessed on Sept. 17 2020).


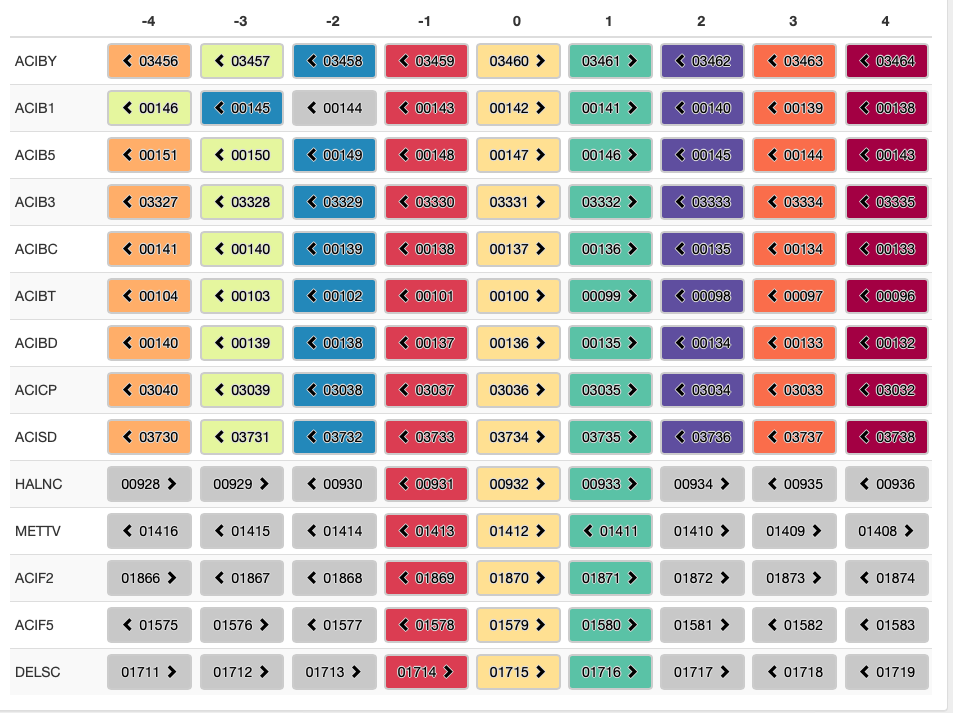


**Fig F. Microsynteny of AbaI, AbaM and AbaR orthologs across multiple Acinetobacter species.** (ACIXX abbreviations, except ACIF2= Acidovorax sp. JS42 and ACIF5= Acidithiobacillus ferrooxidans ATCC 53993, Delftia sp. Cs1-4 (DELSC), Methylobacter tundripaludum ATCC BAA-1195 (METTV) and Halothiobacillus neapolitanus strain ATCC 23641 (HALNC).AbaM(yellow, 0) orthologs are always flanked by AbaI (-1) and AbaR (1). Source: Oma Synteny analysis (<https://omabrowser.org/oma/synteny/5125060/5/4/>). ACIBY represents A. baumannii AYE. Oma lists 5, 137, and 367 orthologs outside of Acinetobacter for AbaM, AbaI and AbaR respectively.

### Integration of the QSLux system with the NRPS cluster

In *A. baumannii* ATCC 19606, the QS_Lux_ cluster is flanked by the A1S_0112-A1S_0119 operon (NRPS cluster; described in strain ATCC 17978 [10, 14, 20]), which is responsible for the biosynthesis of a three-amino acid lipopeptide (Ac-505) [14]. Ac-505 has been implicated to play a central role in regulating bacterial motility and biofilm formation [20]. Disrupting its biogenesis decreases host cell adhesion and virulence of *A. baumannii* markedly by downregulating various factors involved in Biofilm formation on biotic surfaces [10], and most markedly the Chaperon-Usher Pathway pili (CUPs) that are relevant for colonization of the urinary tract [3]. Notably, the genes in the NRPS cluster display the same overall abundance pattern as the QS_Lux_ genes, the adjacency of these two gene clusters is conserved throughout the ACB clade (S1 Data id:0162). In total, 197 (8%) A. baumannii strains have lost one or more genes of this evolutionary unit, with the majority (108, 4.6%) having lost the entire cluster. Retention of the QS in absence of the NRPS occurs rarely (13 strains, 0.5%) while only a single strain was observed where the NRPS cluster was present, but the QS_Lux_ genes were missing. Together this suggests that the two clusters form a co-evolving functional super-cluster where quorum sensing serves as the master regulator that occasionally may act without the NRPS cluster.

### ESGC_ACB_-0368 and 0369 (Acinetobactin)

The acinetobactin biosynthesis clusters *bauA-F*, *basA-I* and barAB (Fig 5, id:0368 and id:0369) are almost confined to the ACB clade, and here in particular to *A. baumannii* and *A. pittii* and to lesser extent to *A. lactucae*. The split into two units is caused by diverging profiles of basF and the neighboring basG (S5 Fig id:0368 and id:0369). Upon closer inspection, microsynteny analysis could correct the bloated abundance of basF that resulted from the inclusion of the basF paralog entB into the hierarchical orthologous group. In contrast, orthologs to basG, a histidine decarboxylase, are nearly exclusive to A. baumannii. These findings indicate that the cluster composition was subject to change in the course of ACB diversification and that *basG* likely represents a recent addition.

### ESGC_ACB_-0498 (Enterobactin)

The presence of ESGC_ACB_-0498 is confined to the ACB-clade, hence represents a second line of ACB-specific iron uptake facilitated by 2,3-dihydroxybenzoic acid synthesis cluster (*entAB*, Fig 5, id:0498) for a siderophore precursor sometime referred to as “enterobactin”. Analysis of the microsynteny (S1 Data id:0498) revealed, that orthology prediction, again, found hits outside of the ACB clade for *entA* paralogs that we could correct via microsynteny analysis (see Fig 8).

### ESGC_ACB_-0016 (Carnitine)

The carnitine metabolism cluster displays highly similar abundance profiles for the individual genes especially within the ACB clade except for one gene located at the center of the cluster, the putative acylcarnitine hydrolase (ach?). While the abundance profile of the hydrolase appears clearly distinct from its neighboring genes, further analysis revealed that this is due to a paralogue at a distant locus, which is found in many Acinetobacter species and whose presence/absence pattern is uncorrelated with the pattern of the complementary carnitine cluster (*cf*. S1 Data id:0016). Assessing the presence-absence pattern of the carnitine cluster across the ACB clade revealed that the cluster is rare across the species *A. calcoaceticus.* Apparently, only two strains harbor this cluster, namely A. calcoaceticus EGD_AQ_BF14 (GCF_001663695.1) and A. calcoaceticus XM1570 (GCF_000341835.1). However, the phylogenetic placement of both strains (cf. Figs 2B and S3 for a higher resolution image) indicate that they likely represent falsely classified A. pittii strains. Thus, we conclude that the carnitine cluster is absent from *A. calcoaceticus*, the sometimes considered only non-pathogenic species within the ACB clade. This further contradicts the previous belief that *A. calcoaceticus* can grow on carnitine as a sole carbon source [21, 22]. This observation was made several decades ago and was conducted with *Acinetobacter* *calcoaceticus* LMD79.41. Since species identification in *Acinetobacter* was/is especially hard, it is likely that this strain, too, is a misclassified member of the ACB clade. Unfortunately, we lack access to genomic sequence data to verify this hypothesis.

### ESGC_ACB_-0497 (PQQ biosynthesis)

Across *Acinetobacter*, the genes of ESGC_ABC_-0497 reside contiguously on the same strand suggesting an operon-like structure. Shen et al. reported an identically structured operon including the dipeptidase they labeled PqqF in *Klebsiella pneumoniae* [23]. They further found that this cluster is abundant among pathogenic bacteria. While this is in line with our findings, we could not confirm the the observation that PqqF was frequently, which prompted the authors to conclude on the non-essentiality of this protein. Across Set-R, PqqF was lost in only a single genome (*A. gernerii* DSM 14967) suggesting that PqqF is essential for the function of the PQQ cluster in *Acinetobacter spp*. Further, as mentioned in the main text, no orthology assignment exist for PqqA as its length is below the minimum length cutoff (40 aa) for the orthology search algorithm. A manual, unidirectional search by sequence similarity, however, suggest a very similar presence/absence profile: Across SetR 54/55 in *A. baumannii*, 91/93 in the ACB, 24/141 in non-ACB genomes in comparison to the other members of the cluster (e.g. PqqB 54,92,24, respectively).

## Gene clusters (ESGCs) not discussed in main manuscript

In the following, we highlight a number of clusters that might be of interest in the context of ACB pathogenicity, which we could not discuss in the main manuscript for space reasons.

### ESGC_ACB_-0622 (Phenylacetate metabolism)

One important route for several aromatic compounds is the phenylacetate catabolic pathway (PAA) encoded by the *paa* operon (Fig G). Many of these routes converge and are directed to the citric acid cycle [9]. Besides a possible selective advantage through metabolic flexibility, it was shown that the capability to metabolize aromatic compounds allows bacteria to better cope with environmental pollutants [24]. Teufel et al. discussed how early intermediates of this metabolic pathway could contribute to virulence through secretion and toxic effects on the host [25].


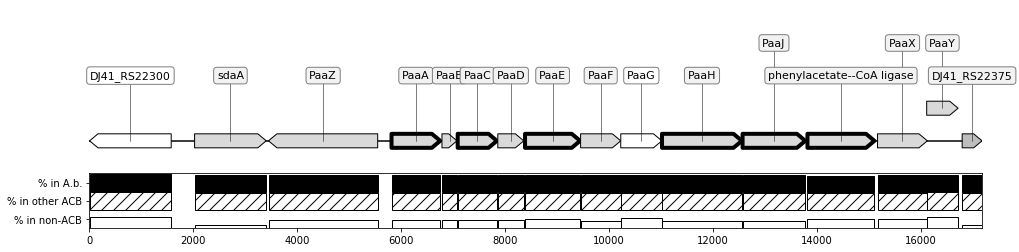
 **Fig G. Phenylacetate metabolism cluster between loci DJ41_RS22300 and DJ41_RS22375.** The enzymes represented: PaaK, phenylacetate-CoA ligase (AMP forming); PaaA-E, ring 1,2-phenylacetyl-CoA epoxidase (NADPH); PaaG, ring 1,2-epoxyphenylacetyl-CoA isomerase (oxepin-CoA forming); PaaZ, oxepin-CoA hydrolase (NADP+); PaaJ, 3-oxoadipyl-CoA; PaaF, 2,3-dehydroadipyl-CoA hydratase; PaaH, 3-hydroxyadipyl-CoA dehydrogenase (NAD+). Resembles organization of the gene cluster responsible for phenylacetate degradation in A. baumannii ATCC 17978. The entire cluster is composed of 15 coding sequences and a single promoter region [identical in [9]].

### ESGC_ACB_-0078 (Tricarballylate metabolism)


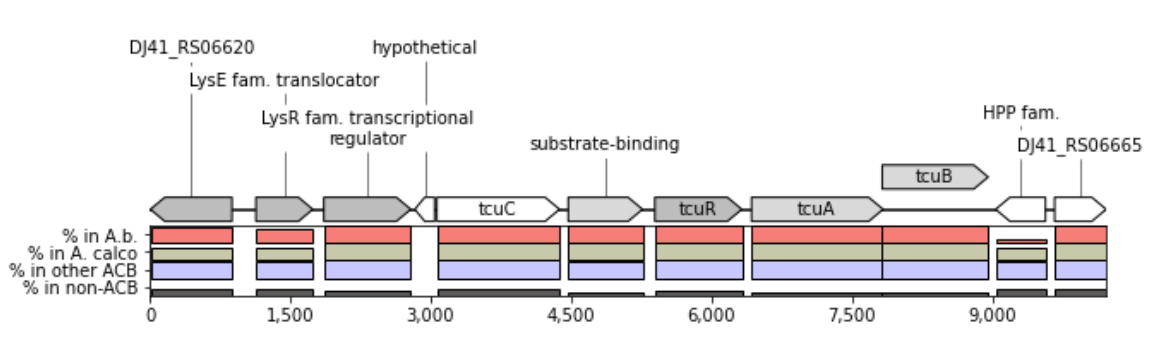


**Fig H. Tricarballylate metabolism cluster in A. baumannii ATCC 19606 located between loci DJ41_RS06625 and DJ41_RS06660.** It encompasses two LysR family transcriptional regulators of which one is annotated

The ESGC_ACB_-0078 encompasses five genes that resemble a tricarballylate utilization operon (Fig H). A similar operon was previously studied in *Salmonella enterica* serovar Typhimurium (*tcuRABC*) [26]. It comprises the FAD-dependent tricarballylate dehydrogenase (*tcuA*), which together with the proteins encoded by *tcuB* (tricarballylate utilization protein) and *tcuC (*tricarballylate proton symporter) catalyzes the oxidization of tricarballylate and similar substrates into cis-aconitate, a substrate of the TCA cycle (Fig H). In *S. enterica,* it enables growth on these substrates as sole carbon sources. The cluster composition in ATCC 19606 suggests two paralogous LysR family transcriptional regulators annotated as *tcuR*. Both genes are separated only by a pseudo gene, *tcuC*, and a putative “molybdate transport system substrate-binding protein”. Microsynteny analysis across the genus revealed that the cluster structure in ATCC 19606 differs from that of the remainder genomes as the transcriptional regulators are paralogous only in the type strain. Usually, the regulator at the cluster start represents *tcuR* and is not a paralog of the putative regulator at the cluster center (S1 Data id:0078). Initial cluster boundaries were manually changed such that the cluster encompasses all genes up to *tcuR* as suggested by the high profile similarities and the stable gene order. Importantly, the loss of the entire cluster, or parts of it is very rare within the ACB clade (23/2728 genomes and 2/2728 genomes, respectively). This indicates a strong selective pressure to maintain cluster presence and integrity. However, why this cluster is highly conserved across the ACB clade is unclear. Especially the clusters’ relevance for human pathogenicity remains unclear given that the model organism of the *tcuRABC*, *Salmonella enterica* is typically an enteropathogen, while *Acinetobacter* isn’t. It can be speculated that Mg2+ chelating capability of tricarballylate is the missing link. In this regard, it might be of relevance that the presence of the entire cluster outside of the ACB clade is highly strain-specific. Orthologs of the full *tcuRABC* were limited to distinct clinical strains of *A. proteolyticus*, *A. bereziniae*, *A. lwoffi,* and *A. courvalini*, a wastewater isolate of *A. tandoii* as well as in the environmental species *A. baylyi* and *A. soli*.

### ESGC_ACB_ -0627 (2-aminoethylphosphonate metabolism)


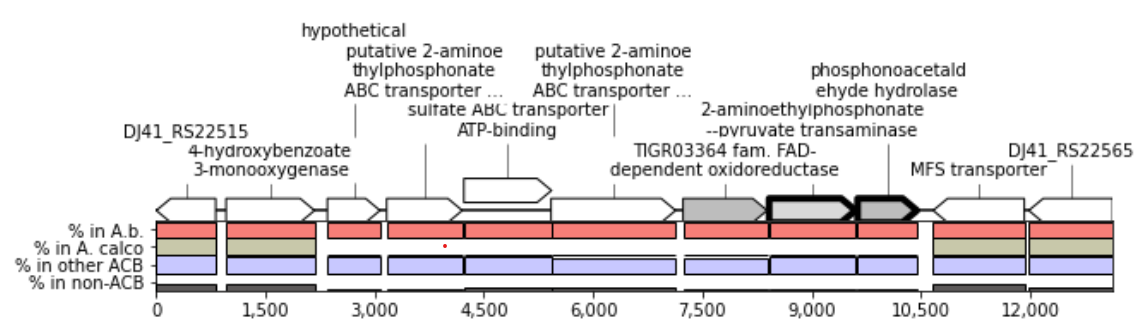


**Fig I. 2-aminoethylphosphonate metabolism cluster located between loci DJ41_RS22520 and DJ41_RS22560.** It encompasses a hypothetical protein, several components of an ABC transporter for 2-aminoethylphosphonate (AEP) including a substrate binding protein, an ATP binding protein, and a permease unit. The cluster also features an generically annotated FAD-dependent oxidoreductase as well the AEP pyruvate transaminase (phnW) and a phosphonoacetaldehyde hydrolase (phnX).

The evolutionarily stable cluster 0627 encompasses seven genes that are functionally related to the transport (ABC transporter) and degradation (*phnX* and *phnW)* of 2-aminoethylphosphonate (AEP). Given the cluster’s highly homogeneous abundance profile (Fig I), there is a strong indication that the two functionally uncharacterized genes encompassed by the ESGC – namely the hypothetical protein at the cluster start and the FAD-dependent oxidoreductase located upstream of *phnW* – are also linked to AEP metabolism. AEP and its N-alkylated derivatives are the most abundant and ubiquitous of the naturally occurring phosphonates [27]. In some lower eukaryotes, they are found to perform essential biochemical functions. In pathogens, in particular, AEP conjugates are relevant for host infection and persistence [28] rendering enzymes of AEP metabolism interesting candidates for drug development as AEP is an important precursor for the biosynthesis of phosphonolipids, phosphonoproteins, and phosphonoglycans [29]. Intriguingly, the entire cluster is absent in all strains of *A. calcoaceticus*, the only species that is largely non-pathogenic within the ACB clade. Taken together, these findings render the catabolism of AEP a prime candidate for experimental characterization regarding its role in *Acinetobacter* virulence.

### ESGC_ACB_-0372 (Xanthine metabolism)


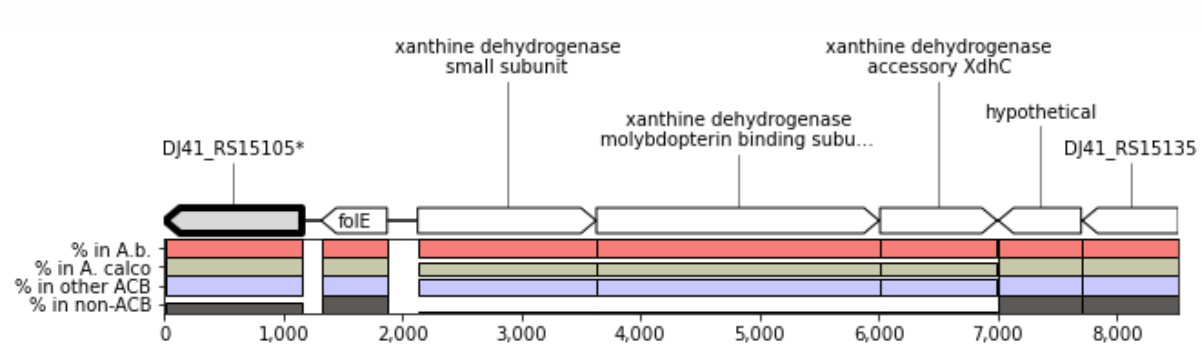


**Fig J. Xanthine metabolism cluster located between the loci DJ41_RS1510 and DJ41_RS15130.** It comprises three genes encoding the small and large xanthine dehydrogenase subunits xdhA and xdhB, respectively [EC:1.17.1.4] as well as the accessory factor xdhC.

The genes of the small (*xdhA*) and large molybdopterin binding subunit (*xdhB*) of the Xanthine dehydrogenase and an accessory factory (*xdhC*) form a cluster (Fig J) that is among the top ranking in terms of ACB clade preferential abundance. Xanthine dehydrogenase catalyzes the irreversible, NAD-dependent and H_2_0-producing oxidization of hypoxanthine to xanthine and from xanthine to uric acid as essential steps in purine metabolism. In humans, *xdh* (also named xanthine oxidoreductase, XOR) further plays a crucial role in the production of reactive oxygen species. It is active in all mammalian tissues and fluids, however, it is tightly regulated not least due to its role in the production of ROS [30, 31]. Crane et al. showed that enteropathogenic Escherichia coli (EPEC) infection triggers the release of ATP from host cells and that this ATP is broken down to ADP, AMP, adenosine, and adenine [31]. The deamination of adenine to hypoxanthine is catalyzed by *ade*, a nearly ubiquitous enzyme across *Acinetobacter* which resides next to a putative xanthine permease (*pbuG*) of which *Acinetobacter* has several. Since xanthine and hypoxanthine are ubiquitously present in human tissue and fluids, exploiting this resource as a potential carbon or nitrogen source could be a selective advantage.

### ESGC_ACB_-0555 (Taurine metabolism)


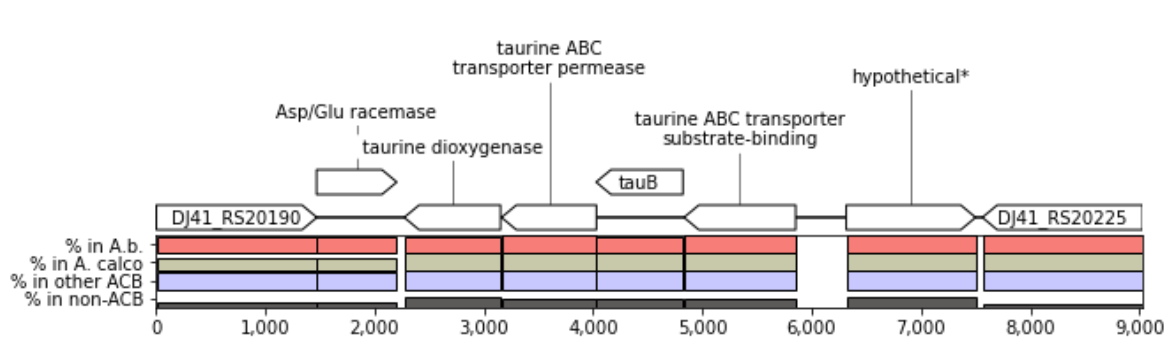


**Fig K. Taurine metabolism cluster located between the loci DJ41_RS20195 and DJ41_RS20220.** It is comprised of a taurine dioxygenase (tauD) and the gene of the taurine ABC transporter which includes the periplasmic binding protein (TauA), the ATP-binding subunit, and the membrane subunit (tauC).

The originally predicted cluster encompassed 11 genes including *tauABCD*, an allantoin racemase (*hpxA*), and cation transporter besides several generically or hypothetically annotated proteins. However, on close inspection with the tool Vicinator, the profile of the large cluster is highly disrupted. Many contig breaks fall within the cluster indicating that genome assembly in this region is complicated. This made an interpretation of the cluster abundance hard (S1 Data id:0555). Still, we find conserved instances of the entire cluster e.g. in strains of *A. pittii* and other members of the ACB clade. Thus, although the entire cluster is evolutionarily unstable, the *tauABCD* sub-cluster is not (Fig K). Only 7 strains that belong to the international clone type 5 (IC5) have lost the entire *tauABCD* cluster and all the remaining members of IC5 have lost *tauB*. Despite in IC5, the loss of one or two genes of this cluster is rare among the strains of *A. baumannii* and the few affected genomes are distributed across the diversity of IC2 resulting in no clear pattern. Outside the ACB clade, the conservation of *tauABCD* is highly species-specific. We find this cluster in all *Acinetobacter* clades, including the distantly related environmental QI clade. Hence, taurine metabolism cannot be confined to an opportunistic pathogenic lifestyle. However, it may convey a selective advantage during infection as taurine is the major organic solute in mammals [32]. Due to uptake from the blood specific cells such as neutrophils can contain very high concentrations [33].

# References

1. Simão FA, Waterhouse RM, Ioannidis P, Kriventseva EV, Zdobnov EM: **BUSCO: assessing genome assembly and annotation completeness with single-copy orthologs.** *Bioinformatics* 2015, **31:**3210-3212.

2. Chernomor O, Minh BQ, Forest F, Klaere S, Ingram T, Henzinger M, von Haeseler A: **Split diversity in constrained conservation prioritization using integer linear programming.** *Methods Ecol Evol* 2015, **6:**83-91.

3. Di Venanzio G, Flores-Mireles AL, Calix JJ, Haurat MF, Scott NE, Palmer LD, Potter RF, Hibbing ME, Friedman L, Wang B, et al: **Urinary tract colonization is enhanced by a plasmid that regulates uropathogenic Acinetobacter baumannii chromosomal genes.** *Nat Commun* 2019, **10:**2763.

4. Tomaras AP, Dorsey CW, Edelmann RE, Actis LA: **Attachment to and biofilm formation on abiotic surfaces by Acinetobacter baumannii: involvement of a novel chaperone-usher pili assembly system.** *Microbiology* 2003, **149:**3473-3484.

5. Pakharukova N, Tuittila M, Paavilainen S, Malmi H, Parilova O, Teneberg S, Knight SD, Zavialov AV: **Structural basis for Acinetobacter baumannii biofilm formation.** *Proc Natl Acad Sci U S A* 2018, **115:**5558-5563.

6. Brossard KA, Campagnari AA: **The Acinetobacter baumannii biofilm-associated protein plays a role in adherence to human epithelial cells.** *Infect Immun* 2012, **80:**228-233.

7. Weidensdorfer M, Ishikawa M, Hori K, Linke D, Djahanschiri B, Iruegas R, Ebersberger I, Riedel-Christ S, Enders G, Leukert L, et al: **The Acinetobacter trimeric autotransporter adhesin Ata controls key virulence traits of Acinetobacter baumannii.** *Virulence* 2019, **10:**68-81.

8. Tomaras AP, Flagler MJ, Dorsey CW, Gaddy JA, Actis LA: **Characterization of a two-component regulatory system from Acinetobacter baumannii that controls biofilm formation and cellular morphology.** *Microbiology* 2008, **154:**3398-3409.

9. Cerqueira GM, Kostoulias X, Khoo C, Aibinu I, Qu Y, Traven A, Peleg AY: **A global virulence regulator in Acinetobacter baumannii and its control of the phenylacetic acid catabolic pathway.** *J Infect Dis* 2014, **210:**46-55.

10. Rumbo-Feal S, Perez A, Ramelot TA, Alvarez-Fraga L, Vallejo JA, Beceiro A, Ohneck EJ, Arivett BA, Merino M, Fiester SE, et al: **Contribution of the A. baumannii A1S_0114 Gene to the Interaction with Eukaryotic Cells and Virulence.** *Front Cell Infect Microbiol* 2017, **7:**108.

11. Rutherford ST, Bassler BL: **Bacterial quorum sensing: its role in virulence and possibilities for its control.** *Cold Spring Harb Perspect Med* 2012, **2**.

12. Niu C, Clemmer KM, Bonomo RA, Rather PN: **Isolation and characterization of an autoinducer synthase from Acinetobacter baumannii.** *J Bacteriol* 2008, **190:**3386-3392.

13. Bhargava N, Sharma P, Capalash N: **Quorum sensing in Acinetobacter: an emerging pathogen.** *Crit Rev Microbiol* 2010, **36:**349-360.

14. Clemmer KM, Bonomo RA, Rather PN: **Genetic analysis of surface motility in Acinetobacter baumannii.** *Microbiology* 2011, **157:**2534-2544.

15. Jain A, Perisa D, Fliedner F, von Haeseler A, Ebersberger I: **The Evolutionary Traceability of a Protein.** *Genome Biol Evol* 2019, **11:**531-545.

16. Choudhary KS, Hudaiberdiev S, Gelencser Z, Goncalves Coutinho B, Venturi V, Pongor S: **The organization of the quorum sensing luxI/R family genes in Burkholderia.** *Int J Mol Sci* 2013, **14:**13727-13747.

17. Uzelac G, Patel HK, Devescovi G, Licastro D, Venturi V: **Quorum sensing and RsaM regulons of the rice pathogen Pseudomonas fuscovaginae.** *Microbiology (Reading)* 2017, **163:**765-777.

18. Le Guillouzer S, Groleau MC, Deziel E: **Two rsaM Homologues Encode Central Regulatory Elements Modulating Quorum Sensing in Burkholderia thailandensis.** *J Bacteriol* 2018, **200**.

19. Lopez-Martin M, Dubern JF, Alexander MR, Williams P: **AbaM Regulates Quorum Sensing, Biofilm Formation, and Virulence in Acinetobacter baumannii.** *J Bacteriol* 2021, **203**.

20. Rumbo-Feal S, Gomez MJ, Gayoso C, Alvarez-Fraga L, Cabral MP, Aransay AM, Rodriguez-Ezpeleta N, Fullaondo A, Valle J, Tomas M, et al: **Whole transcriptome analysis of Acinetobacter baumannii assessed by RNA-sequencing reveals different mRNA expression profiles in biofilm compared to planktonic cells.** *PLoS One* 2013, **8:**e72968.

21. Kleber HP, Seim H, Aurich H, Strack E: **[Utilization of trimethylammonium-compounds by Acinetobacter calcoaceticus (author's transl)].** *Arch Microbiol* 1977, **112:**201-206.

22. Miura-Fraboni J, Kleber H-P, Englard S: **Assimilation of γ-butyrobetaine, and d-and l-carnitine by resting cell suspensions of Acinetobacter calcoaceticus and Pseudomonas putida.** *Archives of Microbiology* 1982, **133:**217-221.

23. Shen YQ, Bonnot F, Imsand EM, RoseFigura JM, Sjolander K, Klinman JP: **Distribution and properties of the genes encoding the biosynthesis of the bacterial cofactor, pyrroloquinoline quinone.** *Biochemistry* 2012, **51:**2265-2275.

24. Nogales J, García JL, Díaz E: **Degradation of Aromatic Compounds in Pseudomonas: A Systems Biology View.** In *Aerobic Utilization of Hydrocarbons, Oils and Lipids.* Edited by Rojo F. Cham: Springer International Publishing; 2017: 1-49

25. Teufel R, Mascaraque V, Ismail W, Voss M, Perera J, Eisenreich W, Haehnel W, Fuchs G: **Bacterial phenylalanine and phenylacetate catabolic pathway revealed.** *Proc Natl Acad Sci U S A* 2010, **107:**14390-14395.

26. Lewis JA, Escalante-Semerena JC: **Tricarballylate catabolism in Salmonella enterica. The TcuB protein uses 4Fe-4S clusters and heme to transfer electrons from FADH2 in the tricarballylate dehydrogenase (TcuA) enzyme to electron acceptors in the cell membrane.** *Biochemistry* 2007, **46:**9107-9115.

27. Hilderbrand RL: **The Effects of Synthetic Phosphonates on Living Systems.** In *The effects of synthetic phosphonates on living systems.* Edited by Hilderbrand RL. Boca Raton: CRC Press; 1983: 139-169

28. Kim AD, Baker AS, Dunaway-Mariano D, Metcalf WW, Wanner BL, Martin BM: **The 2-aminoethylphosphonate-specific transaminase of the 2-aminoethylphosphonate degradation pathway.** *J Bacteriol* 2002, **184:**4134-4140.

29. Rizk SS, Cuneo MJ, Hellinga HW: **Identification of cognate ligands for the Escherichia coli phnD protein product and engineering of a reagentless fluorescent biosensor for phosphonates.** *Protein Sci* 2006, **15:**1745-1751.

30. Battelli MG, Polito L, Bortolotti M, Bolognesi A: **Xanthine Oxidoreductase-Derived Reactive Species: Physiological and Pathological Effects.** *Oxid Med Cell Longev* 2016, **2016:**3527579.

31. Saugstad OD, Becher G, Grossmann M, Merker G, Oddoy A, Lachmann B: **Acute and chronic effects of xanthine oxidase on lung thorax-compliance in guinea pigs.** *Intensive Care Med* 1987, **13:**30-32.

32. Huxtable RJ: **Physiological actions of taurine.** *Physiol Rev* 1992, **72:**101-163.

33. Bouckenooghe T, Remacle C, Reusens B: **Is taurine a functional nutrient?** *Curr Opin Clin Nutr Metab Care* 2006, **9:**728-733.
