# Supplementary figures and images for: Evolutionarily stable gene clusters shed light on the common grounds of pathogenicity in the Acinetobacter calcoaceticus-baumannii complex
Source: PLoS Genet. 2022 Jun 2;18(6):e1010020. doi: 10.1371/journal.pgen.1010020 (PMC9162365; doi:10.1371/journal.pgen.1010020)

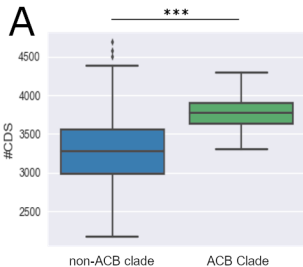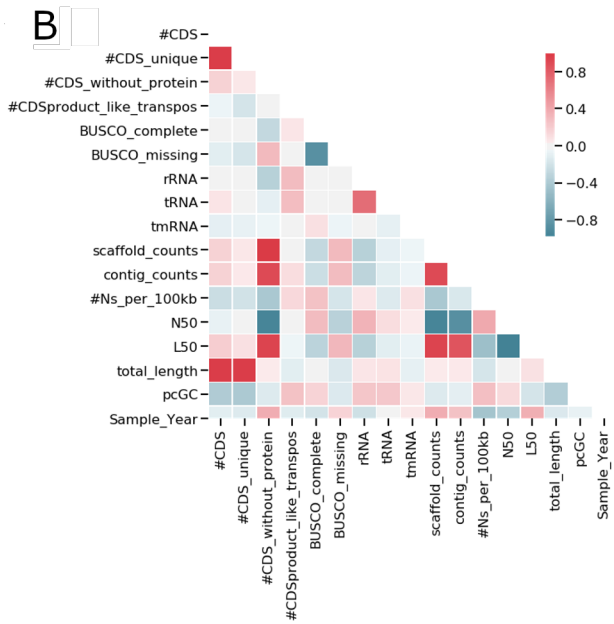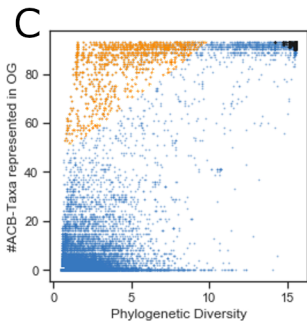

Supplement: S1 Fig — (A) Comparison of the number of coding sequences (CDS) per genome between members and non-members of ACB clade across Acinetobacter. It reveals a significant difference. ACB clade members, on average, contain 14% more protein coding genes. (B) Correlation matrix for a range of summary statistics on genome level across SET-R. Colored cells indicate value of spearman correlation coefficient [–1,1]. The descriptive statistics analyzed are explained in S2 Table. (C) Phylogenetic diversity of the orthologous groups (OGs) calculated from the sum of branch lengths of the subtree spanned by the taxa represented in an OG. This distribution contrasts the taxa belonging to the ACB clade vs. the total phylogenetic diversity. Data points are colored black if the corresponding OG belongs to the set of genus-wide core genes that were also used for phylogeny reconstruction. OGs represented closer to the upper left corner are especially interesting as they are approaching ubiquitous presence within the ACB clade but are rare in the rest of the genus (colored orange for illustrative purposes). (PDF) [file pgen.1010020.s002.pdf]

**A**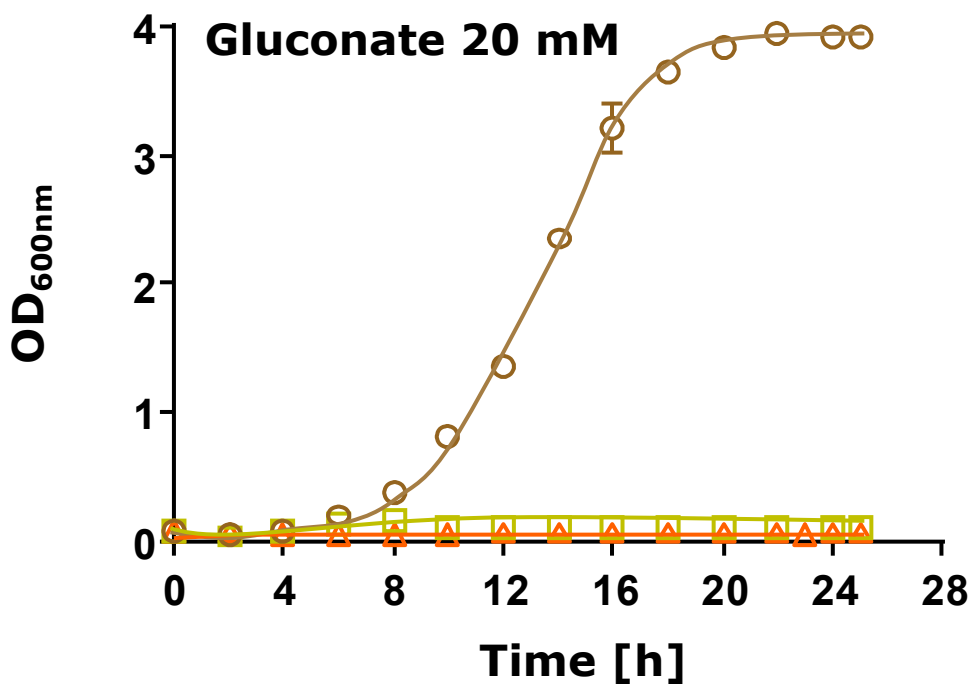**B**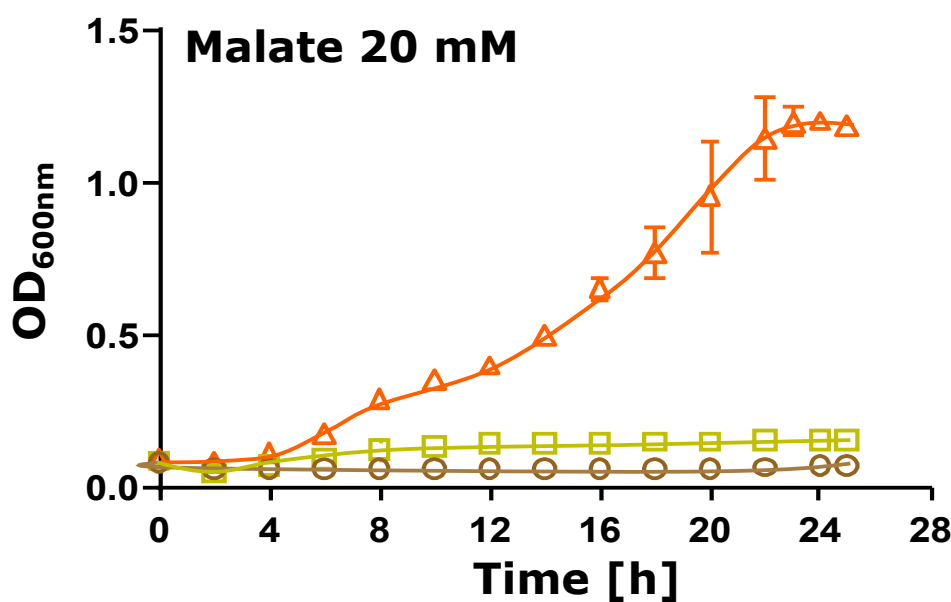**C**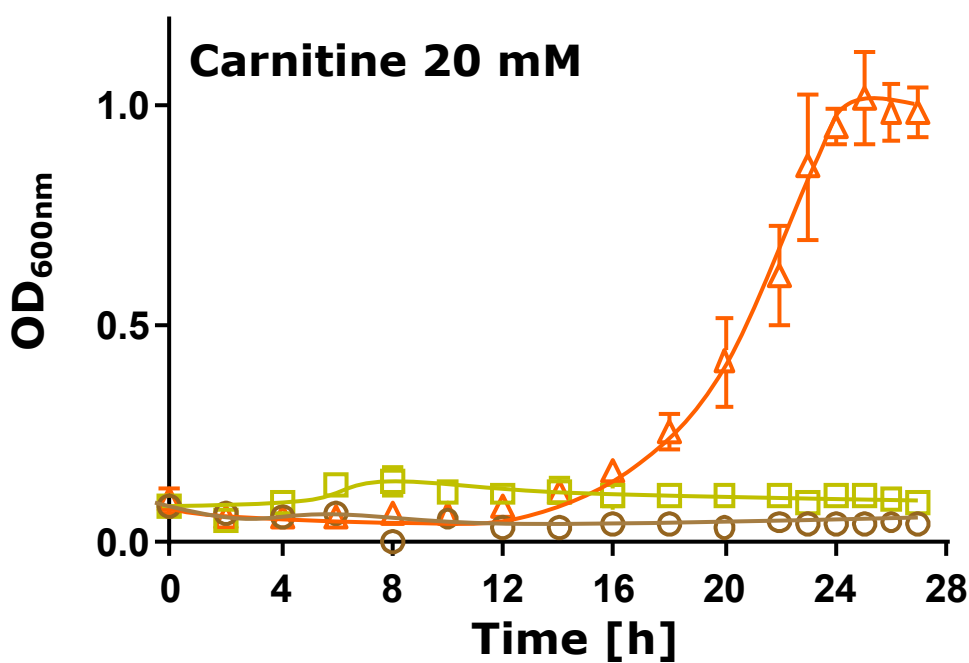

Supplement: S6 Fig — A. baumannii ATCC 19606 (△), A. calcoaceticus (□) and A. baylyi ADP1 (○) were grown in mineral medium with 20 mM D-malate (A), gluconate (B) or carnitine (C) as carbon source. Each value is the mean of +/- S. E.M. of at least three independent measurements. (PDF) [file pgen.1010020.s007.pdf]

**A**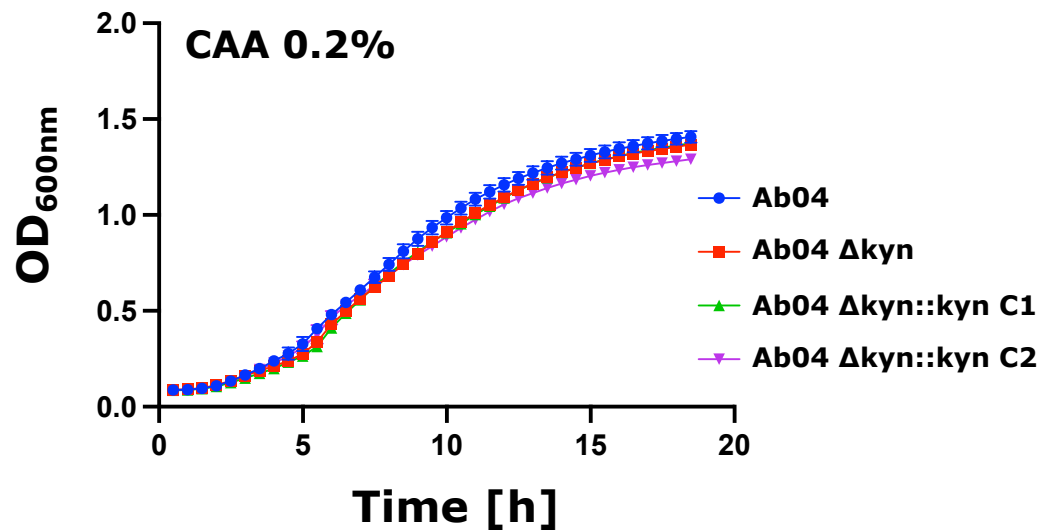**B**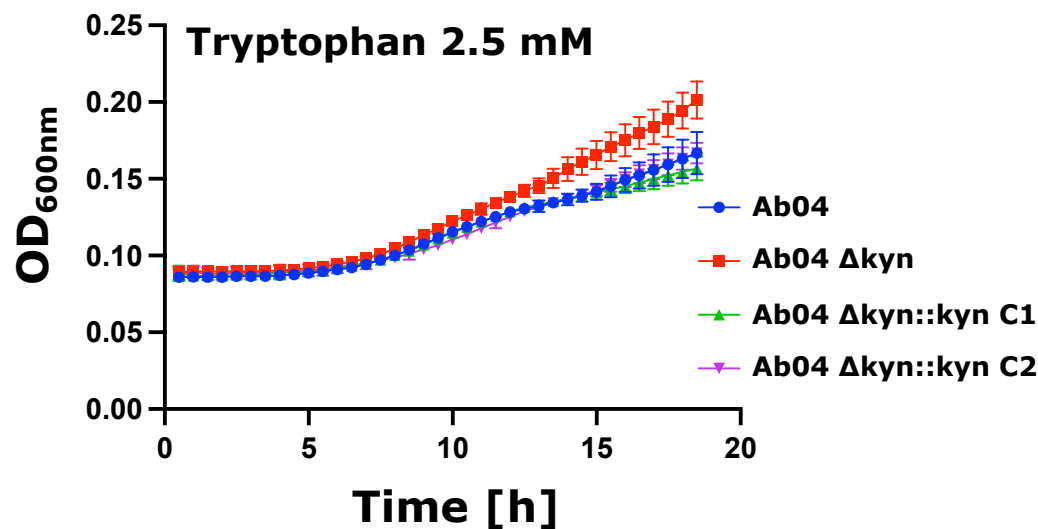

Supplement: S8 Fig — (PDF) [file pgen.1010020.s009.pdf]

## HOG Gain Loss Eventcount

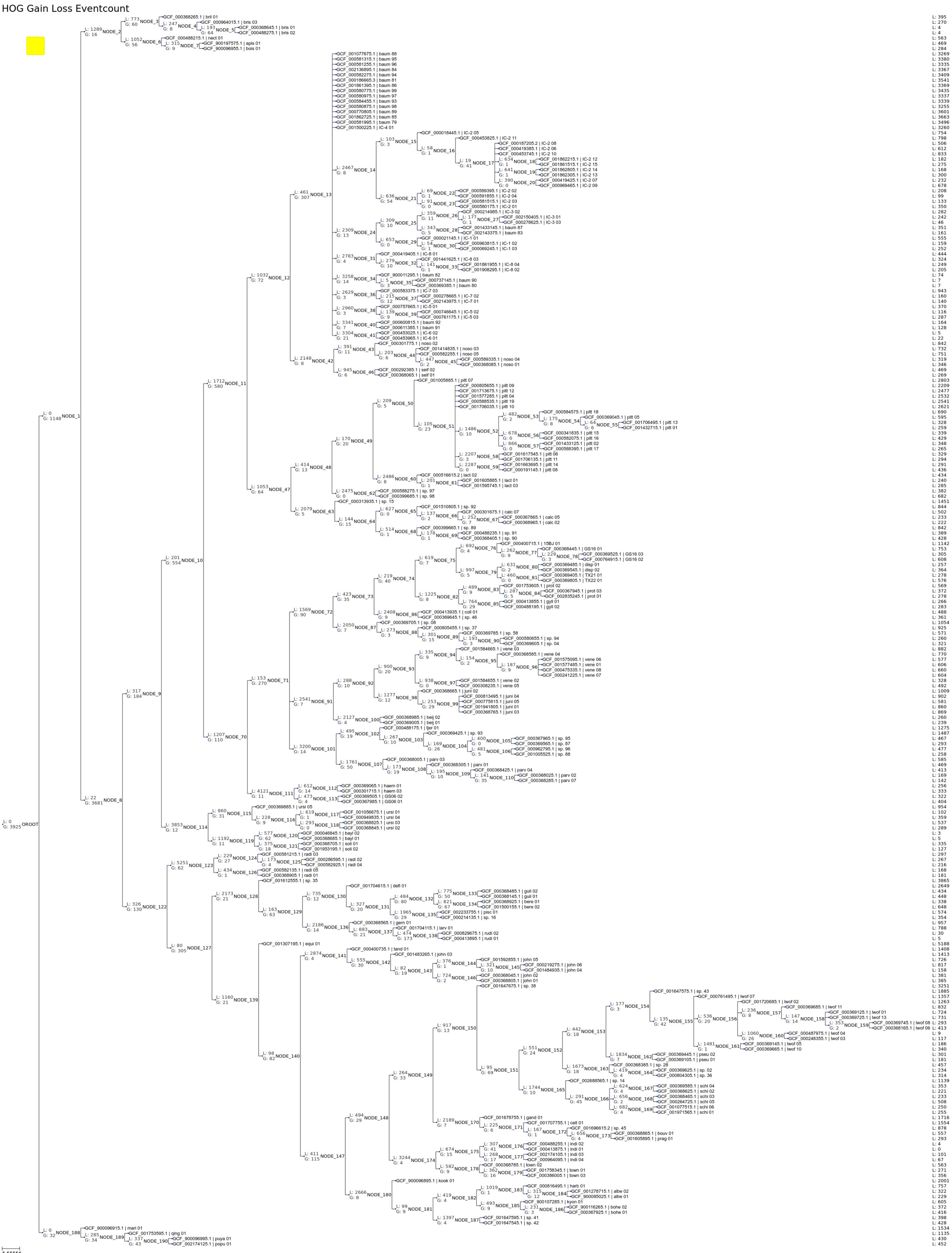

Supplement: S9 Fig — A consensus tree representation of Set-R with each inner node of the tree annotated with the number of HOGs associated to it as well as the number of lost HOGs in the subsumed clade according to the rules of Dollo (+) Parsimony (see Methods). All HOGs are provided in S13 Table. The nodes are labeled with an incremental id. On the lineage of A. baumannii we used the following replacements in the manuscript: NODE_1 = "ACB+BR", NODE_8 = "ACB+LW", NODE_9 = "ACB+BA", NODE_10 = "ACB+HA", NODE_11 = "ACB", NODE_12 = "BNS", NODE_13 = "B". Tip labels are represented as NCBI RefSeq Identifiers. Tip labels also show unique assembly accession and, if applicable either clone type assignment or (corrected) species assignment in form of the first four letters of the species names. (PDF) [file pgen.1010020.s010.pdf]
