# Supplementary material for: Evolutionarily stable gene clusters shed light on the common grounds of pathogenicity in the Acinetobacter calcoaceticus-baumannii complex
Source: PLoS Genet. 2022 Jun 2;18(6):e1010020. doi: 10.1371/journal.pgen.1010020 (PMC9162365; doi:10.1371/journal.pgen.1010020)
Supplement: S2 Fig — A high resolution image of the majority-rule consensus dendrogram of the Set-R taxa as shown in Fig 2A. Branches supported by only two out of three partition trees are indicated with dashed lines, branches supported by only one partition are not resolved. Leaf labels colored in green indicate changed species assignments. Such changes can either (i) correct (i.e. the original species assignment was at odds with the species assignment based on phylogenetic and ANI evidences), (ii) newly specify (i.e. the original species assignment was set to “unknown” (sp.)) or (iii) de-specify (the original species assignment could not be confirmed by phylogenetic evidences, and no alternative assignment was possible. The species label was set to “sp.”) the species assignments as retrieved from NCBI RefSeq at the time of download. (PDF) [file pgen.1010020.s003.pdf]

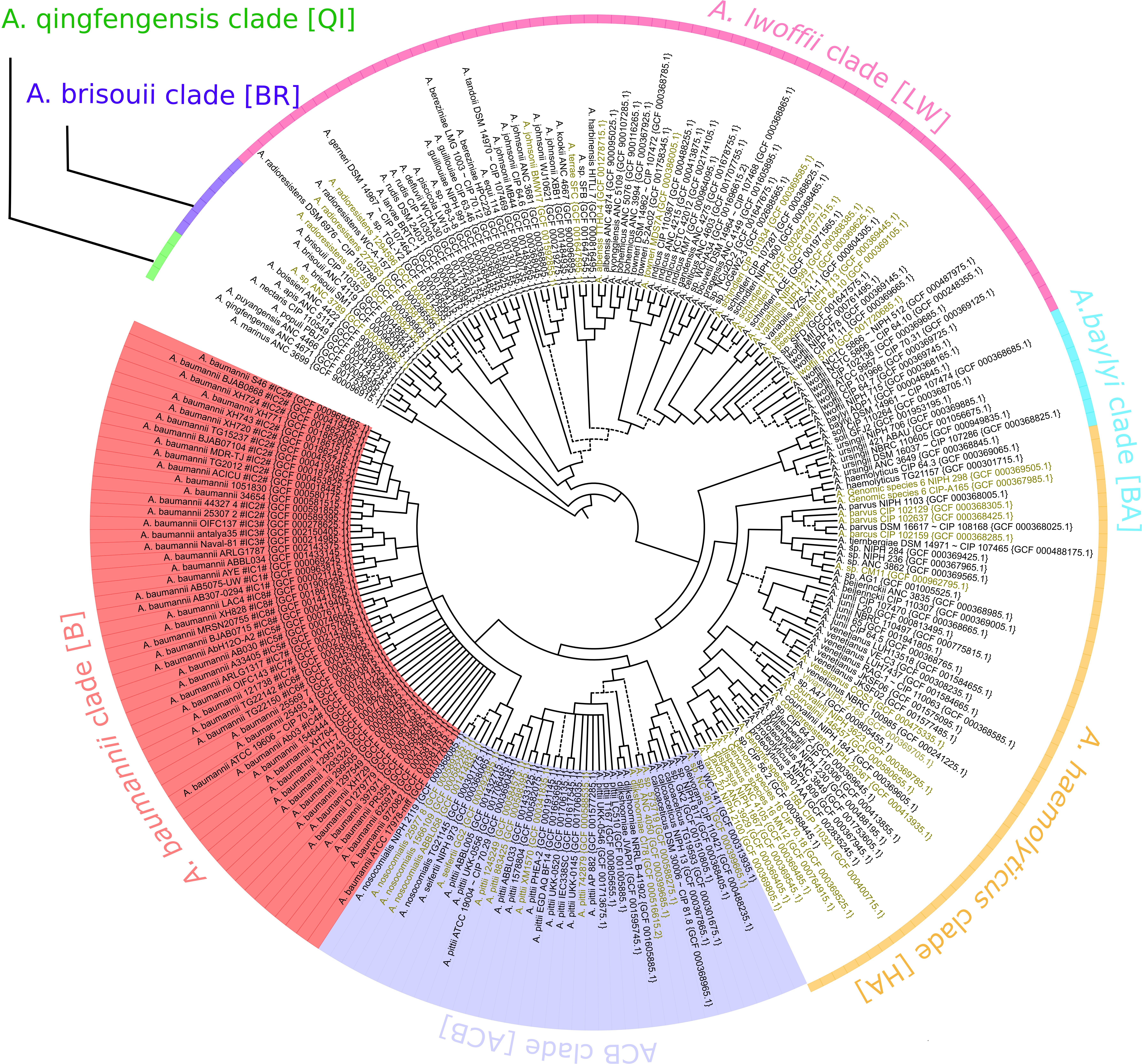

A. qingfengensis clade [QI]
A. brisouii clade [BR]
A. lwoffii clade [LW]
A.baylyi clade [BA]
A. haemolyticus clade [HA]
ACB clade [ACB]
A. baumannii clade [B]
