# Supplementary material for: Evolutionarily stable gene clusters shed light on the common grounds of pathogenicity in the Acinetobacter calcoaceticus-baumannii complex
Source: PLoS Genet. 2022 Jun 2;18(6):e1010020. doi: 10.1371/journal.pgen.1010020 (PMC9162365; doi:10.1371/journal.pgen.1010020)

Top 150 Evolutionary Stable Gene Clusters (ESGC$_{ACB}$s)

Reference Strain: ATCC 19606 (GCF_000737145.1, NCBI RefSeq v87)

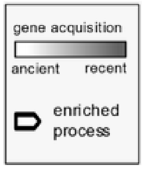

The ESGCs are shown in order of appearance along the replicon NZ_KL810966.1. The locus of the ESGC is depicted embedded within its two upstream and two downstream neighbors. All genes are visualized with their abundance profiles across different taxonomic groups across the Set-R. If no gene symbol was available either through literature search or provided by NCBI RefSeq, the functional annotation of the product was used instead as a label. The left-most and right-most flanking genes are annotated with the locus tag to allow precise localization. Arrow length indicates gene length and direction indicates relative strandedness. Coordinates at the bottom highlight start and end of each coding sequence (CDS) relative to the start of the first CDS. Non-coding genes are not depicted and skipped. Asterisks (“*”) at the end of the function labels demark that the protein was predicted to localize either extracellularly or in the outer membrane. Double exclamation marks (‘!!’) indicate missing orthologous group information due to short protein size (excluded from orthology search).

ESGC_0013 Length: 2

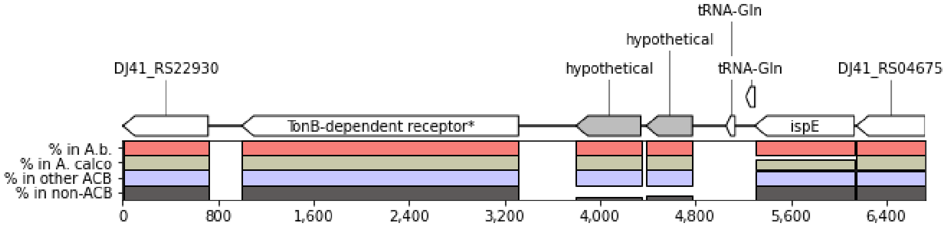

ESGC_0016 Length: 7

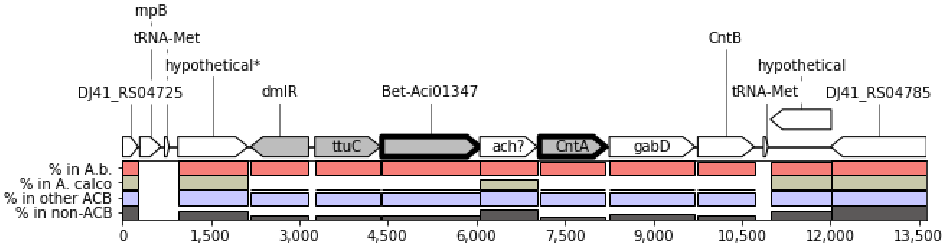

ESGC_0023 Length: 2

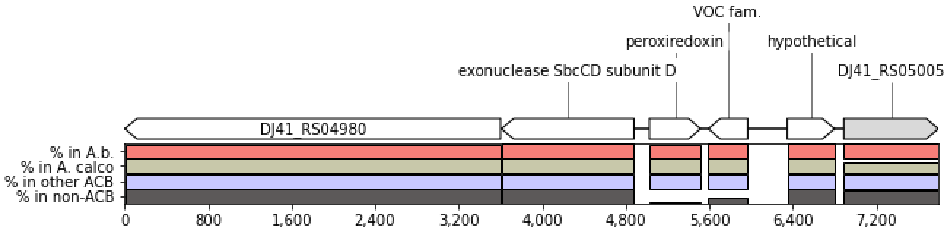

ESGC_0028 Length: 4

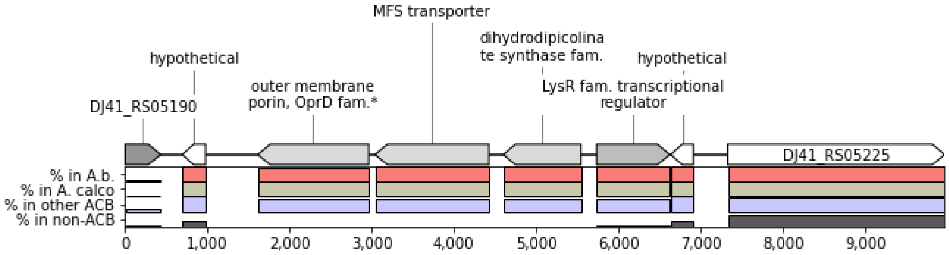

ESGC_0030 Length: 2

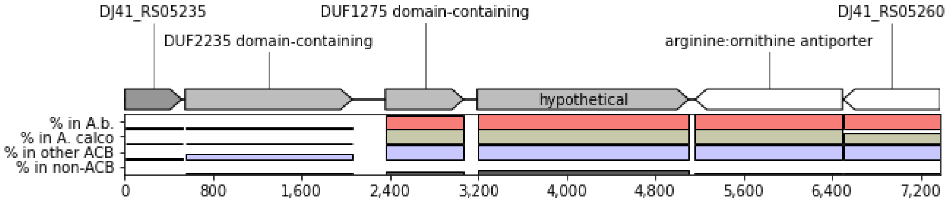

ESGC_0031 Length: 4

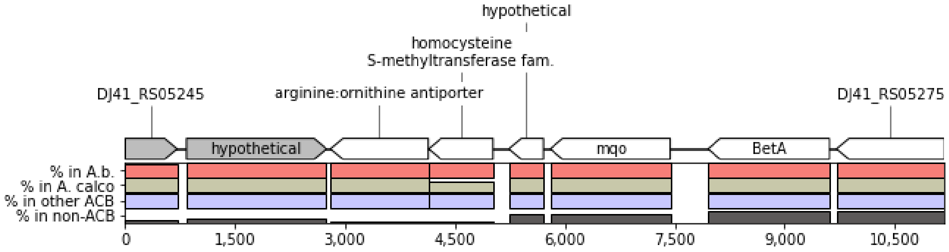

ESGC_0035 Length: 20

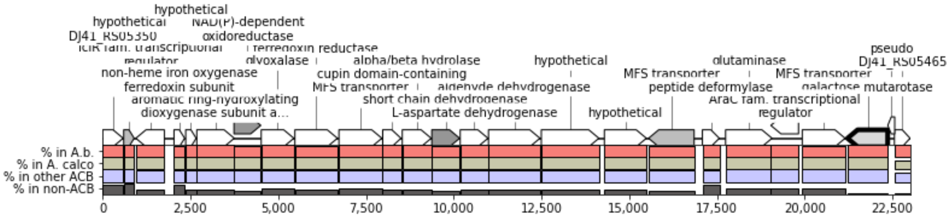

ESGC_0036 Length: 3

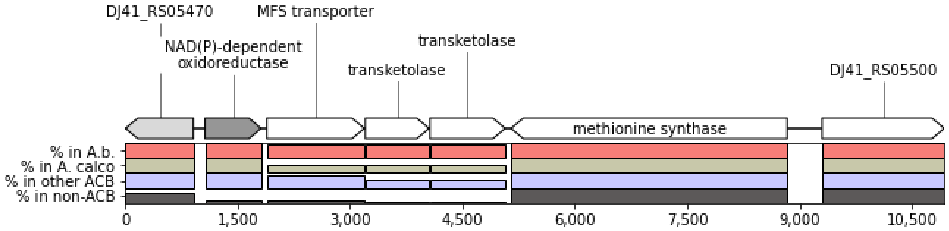

ESGC_0037 Length: 4

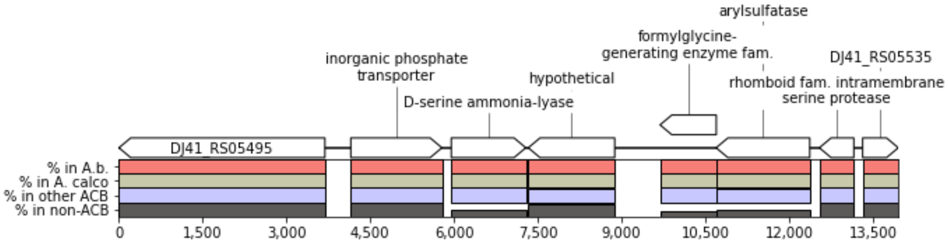

ESGC_0044 Length: 8

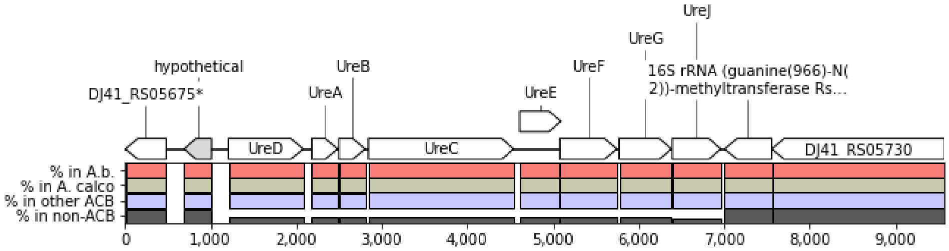

ESGC_0055 Length: 13

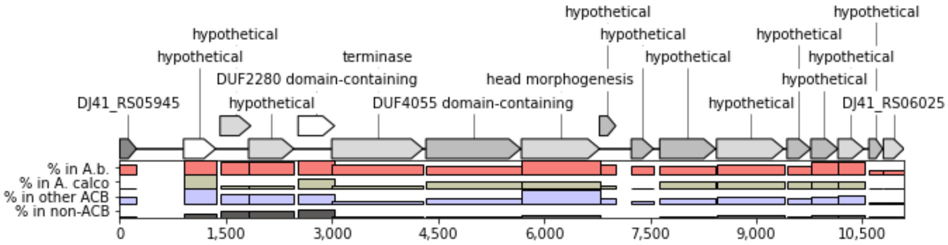

ESGC_0057 Length: 3

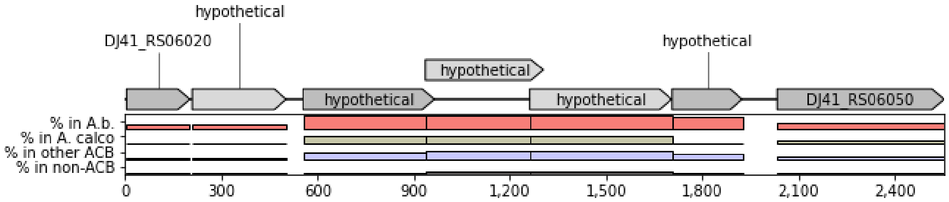

ESGC_0058 Length: 7

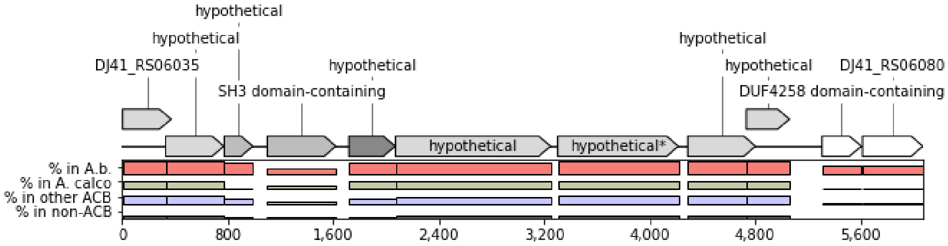

ESGC_0059 Length: 9

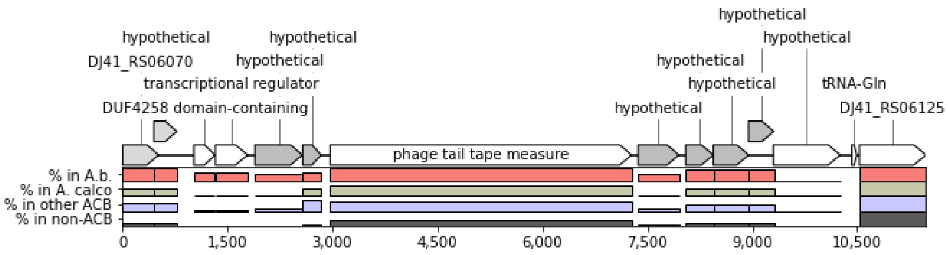

ESGC_0061 Length: 2

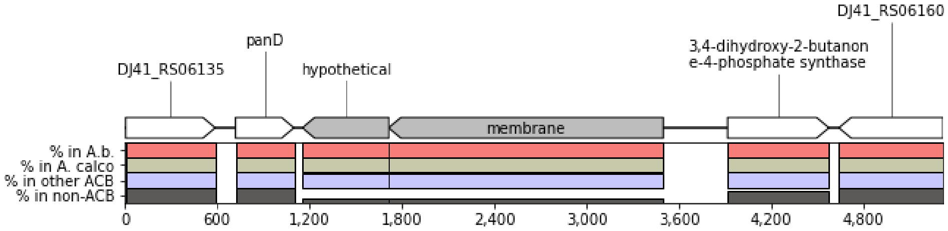

ESGC_0064 Length: 3

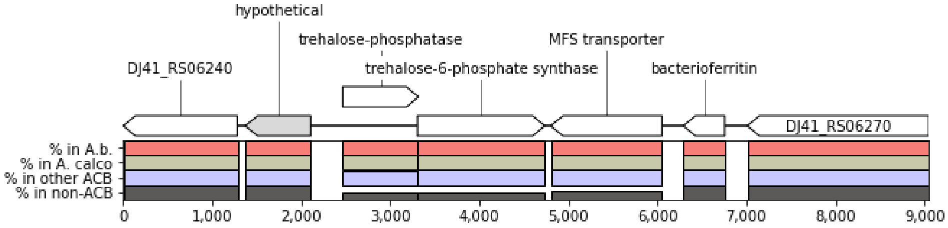

ESGC_0069 Length: 3

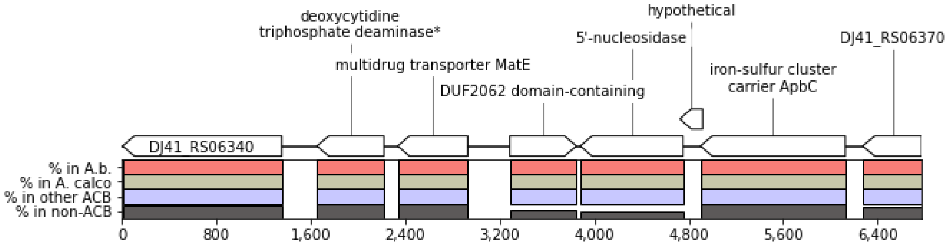

ESGC_0077 Length: 2

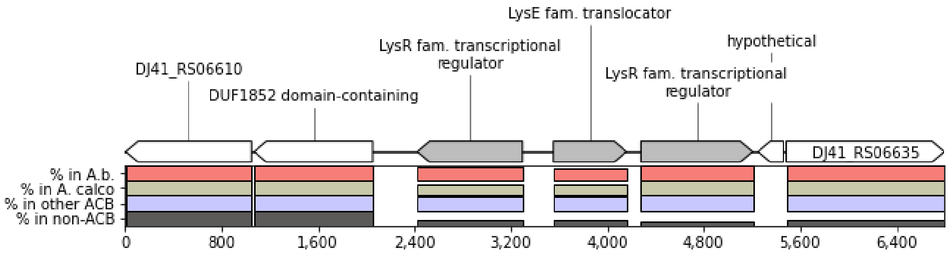

ESGC_0078 Length: 6

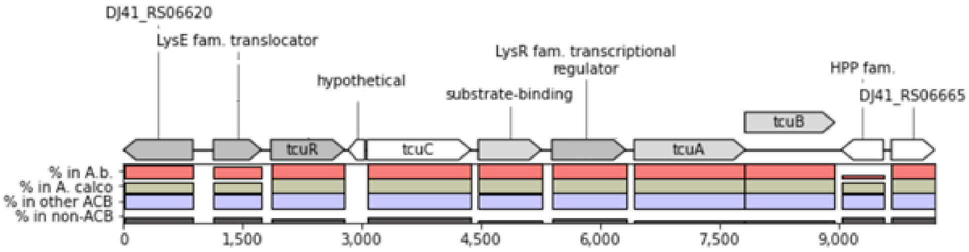

ESGC_0080 Length: 3

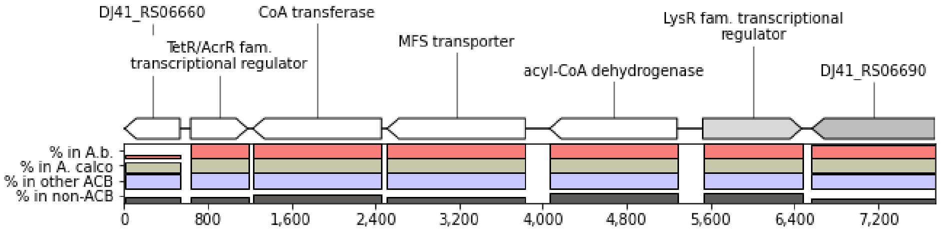

ESGC_0081 Length: 3

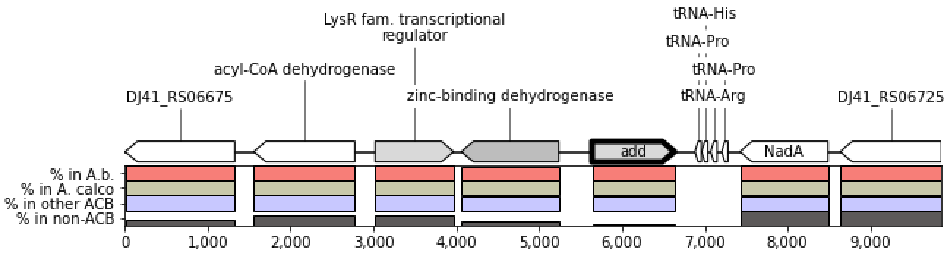

ESGC_0092 Length: 2

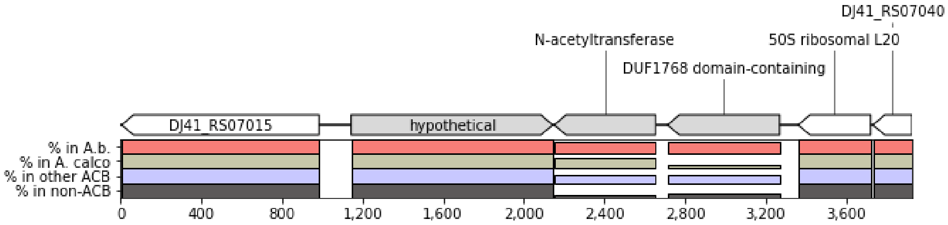

ESGC_0100 Length: 2

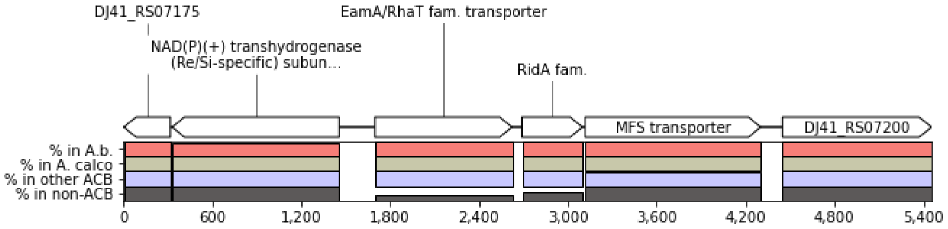

ESGC_0112 Length: 5

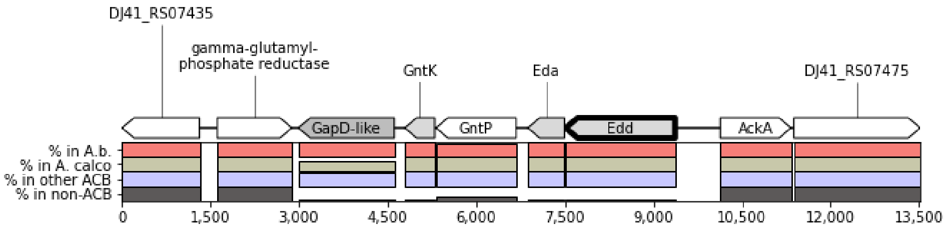

ESGC_0123 Length: 2

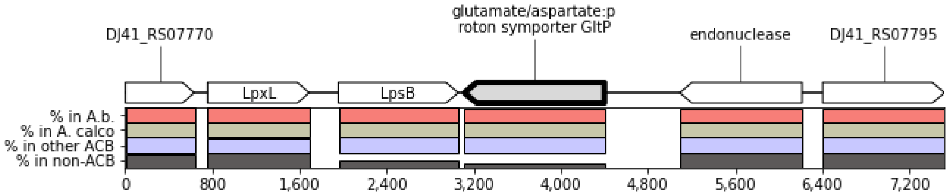

ESGC_0130 Length: 2

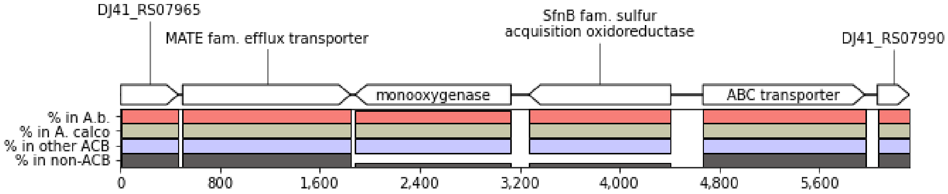

ESGC_0148 Length: 2

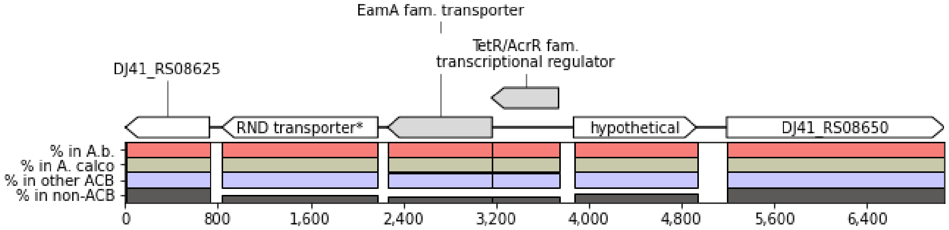

ESGC_0150 Length: 3

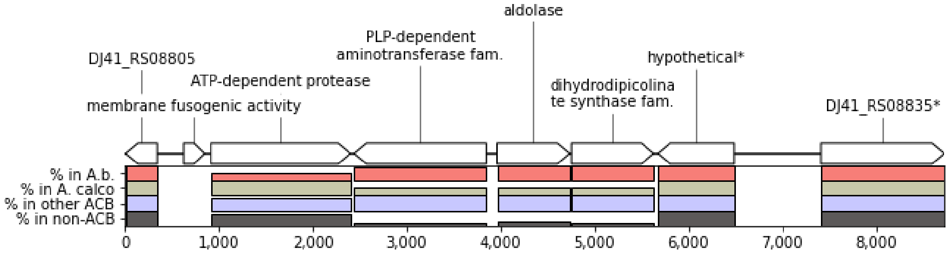

ESGC_0153 Length: 3

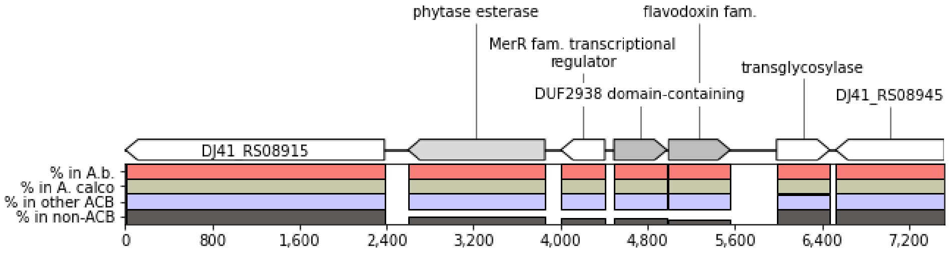

ESGC_0157 Length: 3

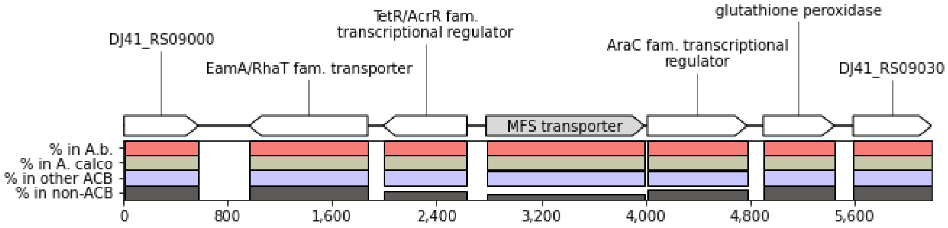

ESGC_0162 Length: 10

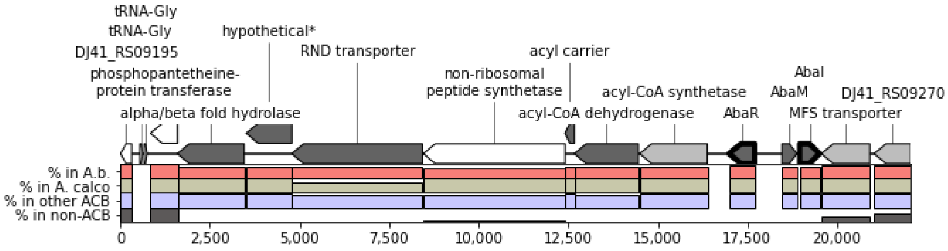

ESGC_0164 Length: 3

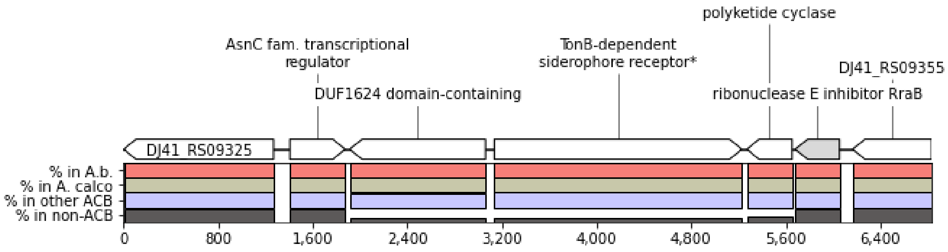

ESGC_0165 Length: 2

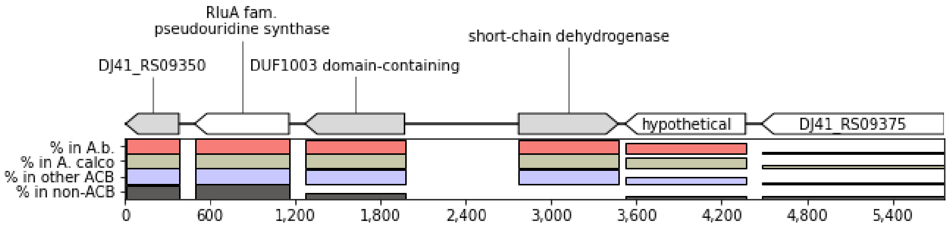

ESGC_0191 Length: 2

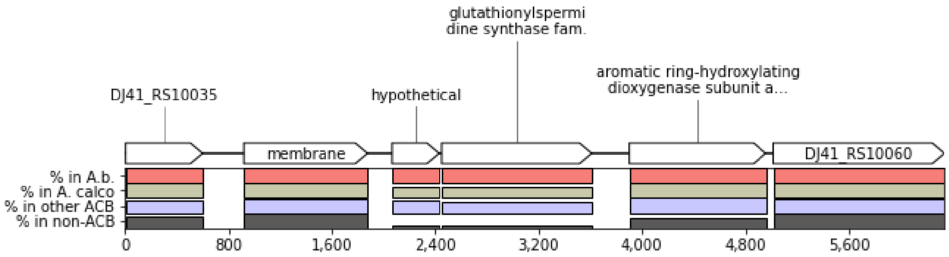

ESGC_0199 Length: 2

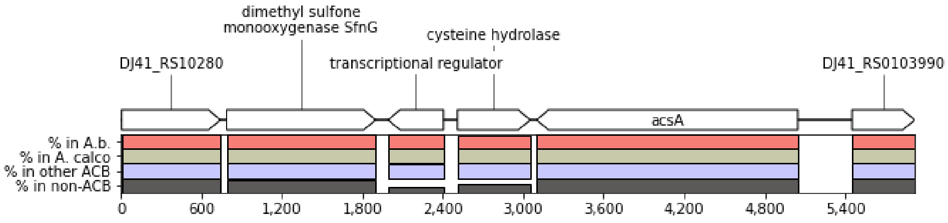

ESGC_0214 Length: 3

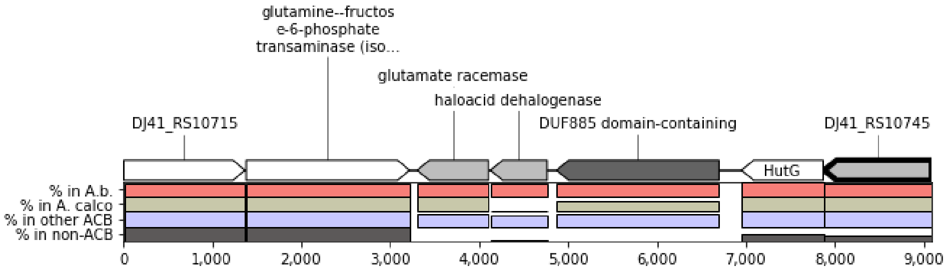

ESGC_0215 Length: 8

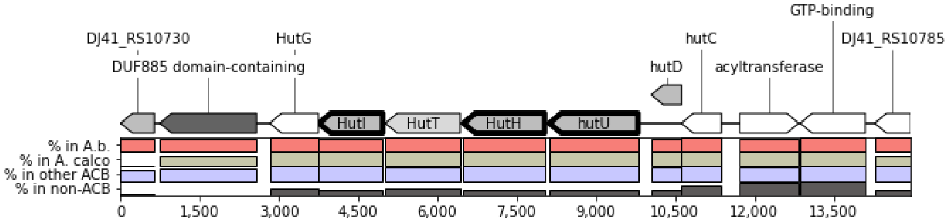

ESGC_0217 Length: 6

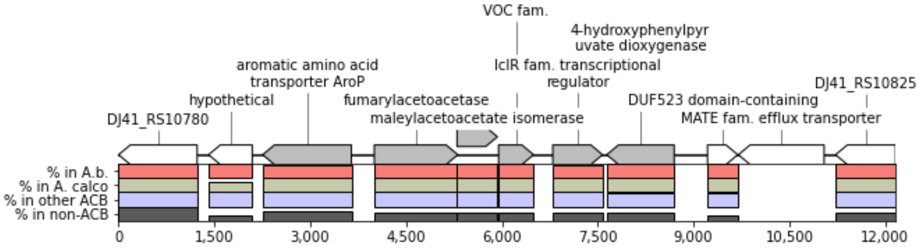

ESGC_0228 Length: 3

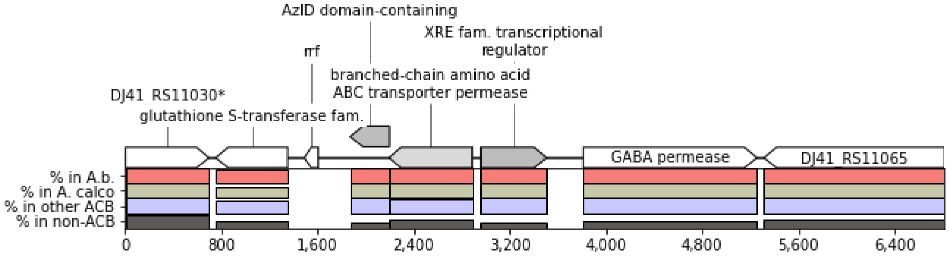

ESGC_0229 Length: 4

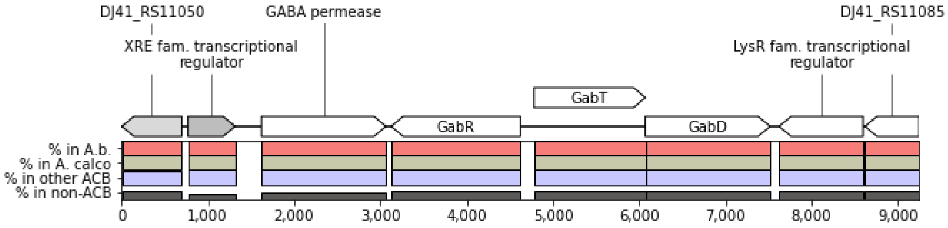

ESGC_0232 Length: 3

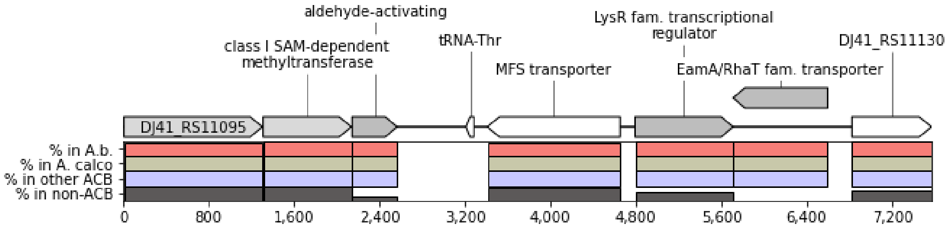

ESGC_0233 Length: 7

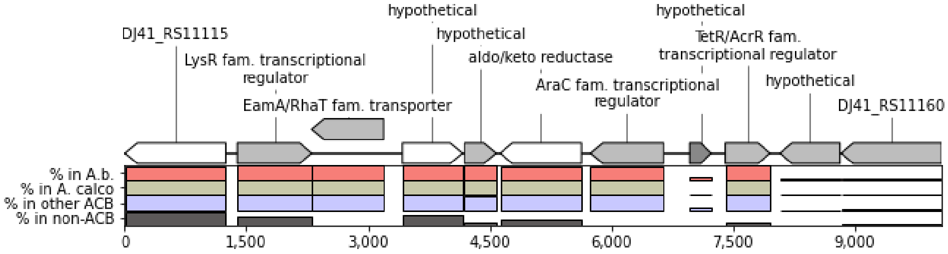

ESGC_0235 Length: 2

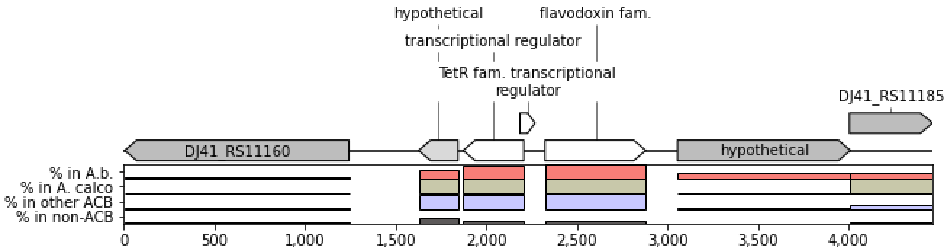

ESGC_0258 Length: 3

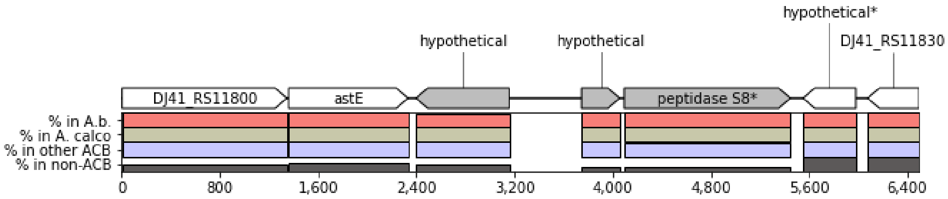

ESGC_0269 Length: 2

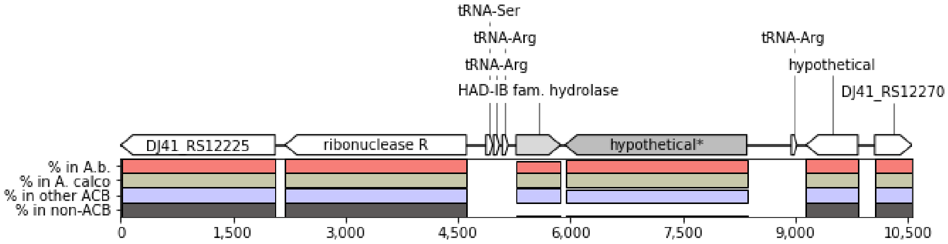

ESGC_0274 Length: 10

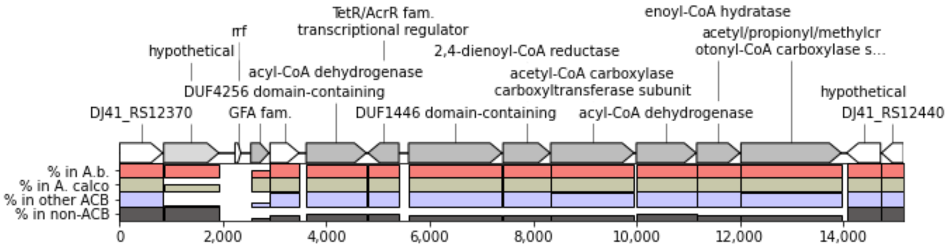

ESGC_0295 Length: 2

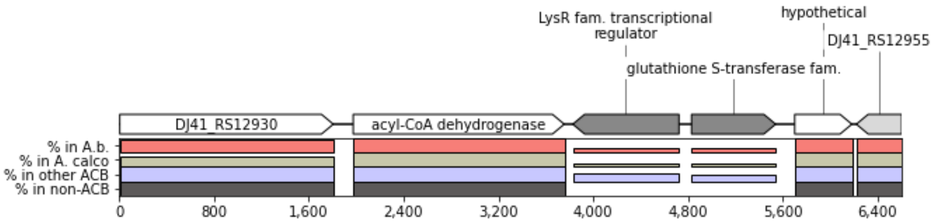

ESGC_0297 Length: 7

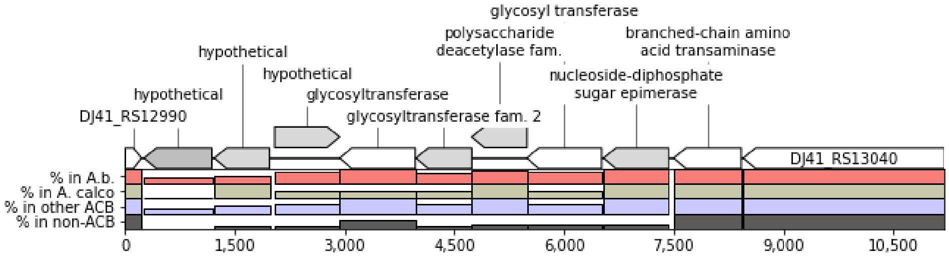

ESGC_0305 Length: 2

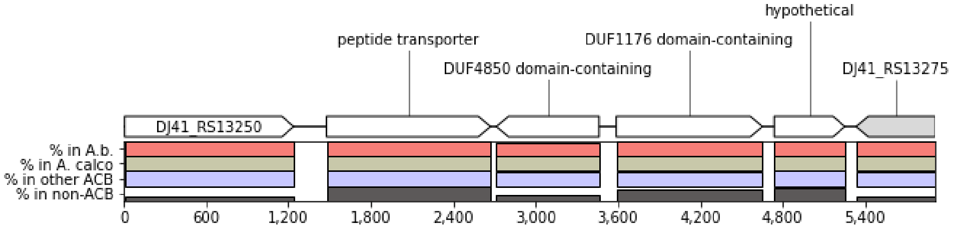

ESGC_0308 Length: 8

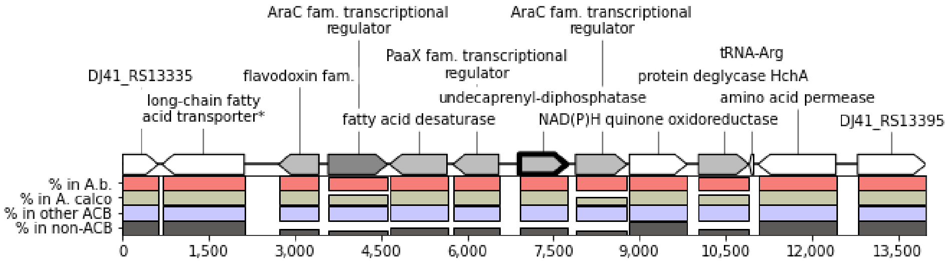

ESGC_0316 Length: 2

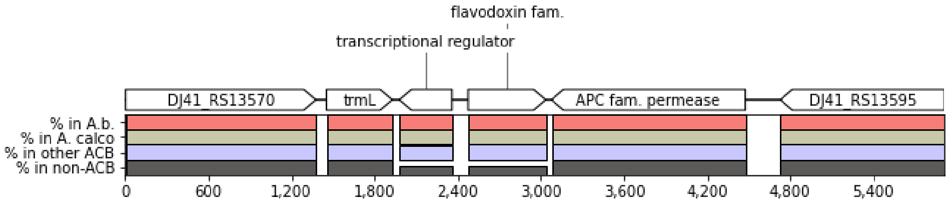

ESGC_0320 Length: 4

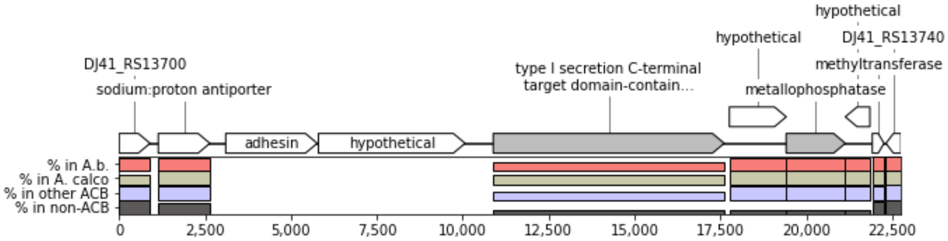

ESGC_0331 Length: 5

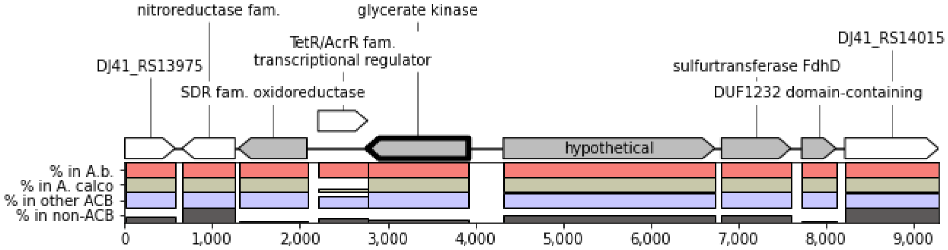

ESGC_0336 Length: 4

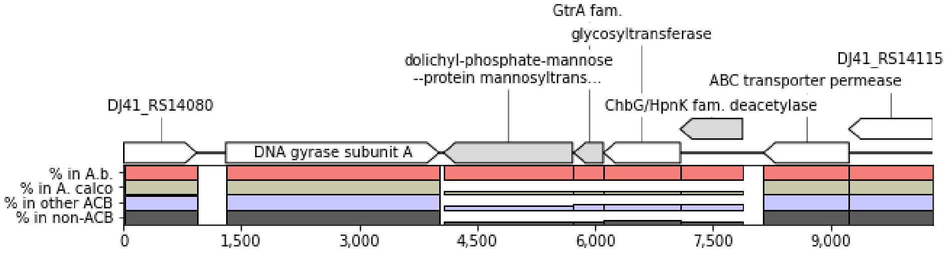

ESGC_0351 Length: 2

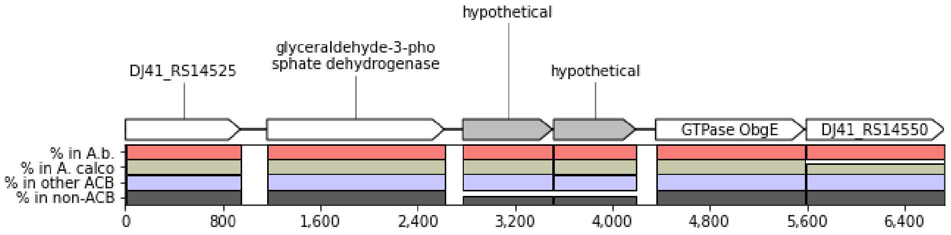

ESGC_0353 Length: 7

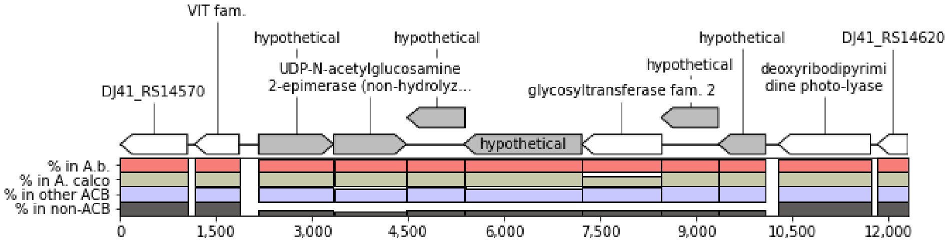

ESGC_0355 Length: 2

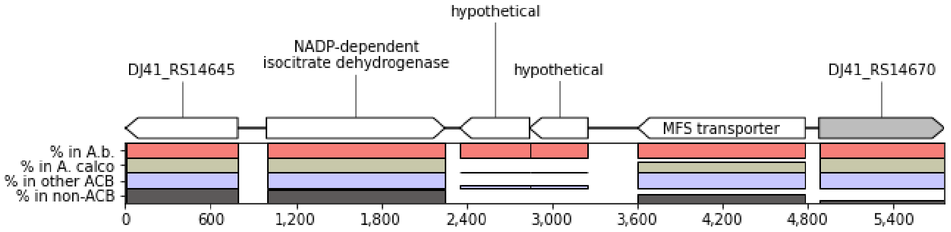

ESGC_0367 Length: 3

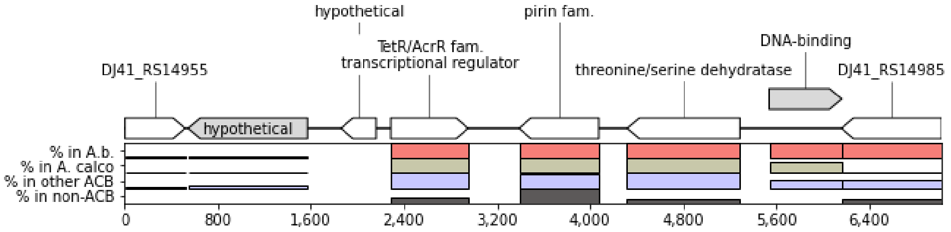

ESGC_0368 Length: 11

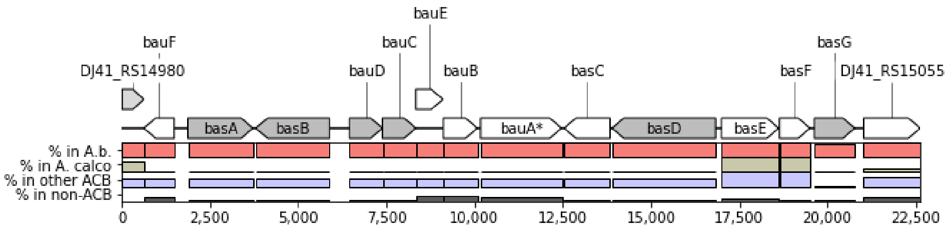

ESGC_0369 Length: 5

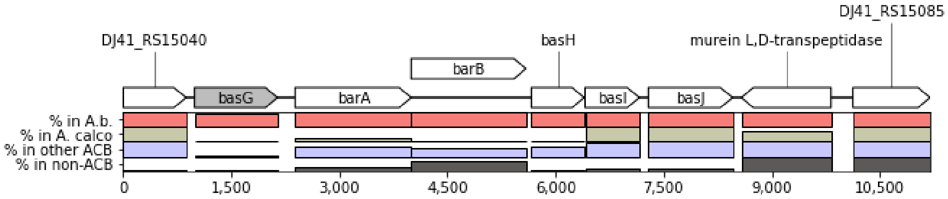

ESGC_0372 Length: 3

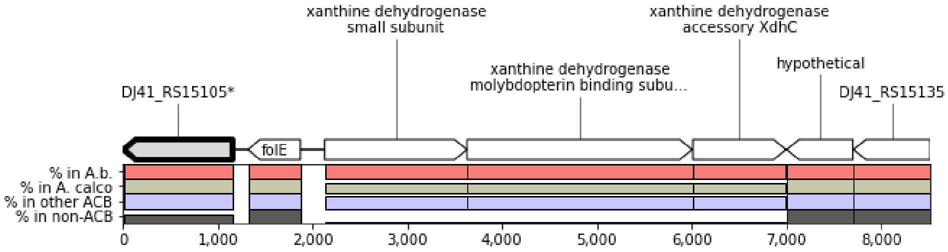

ESGC_0374 Length: 2

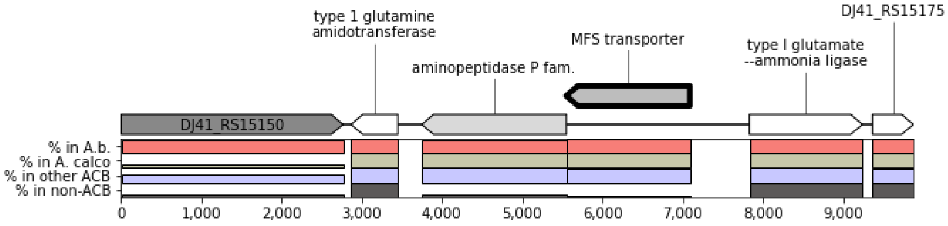

ESGC_0382 Length: 3

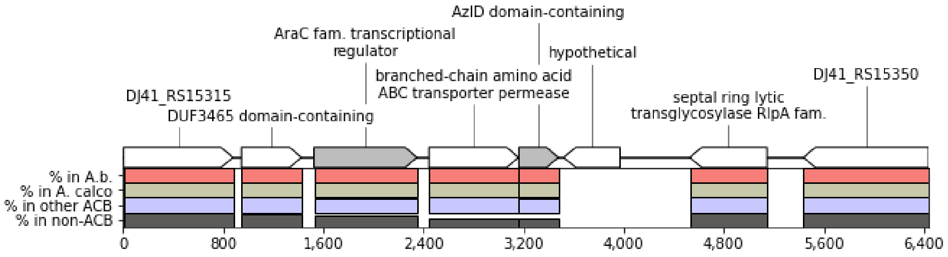

ESGC_0390 Length: 5

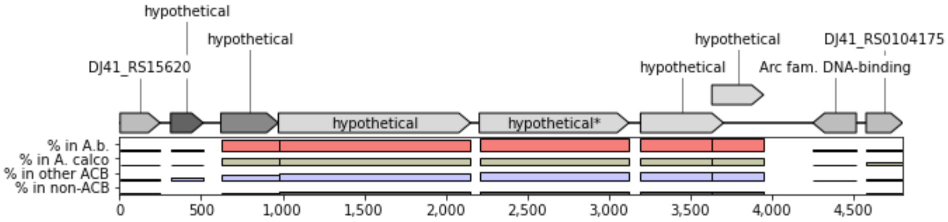

ESGC_0393 Length: 3

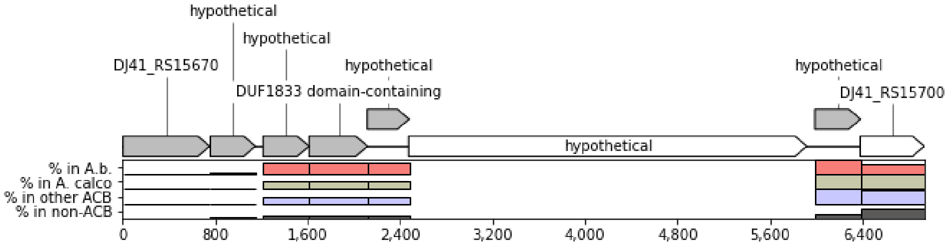

ESGC_0394 Length: 7

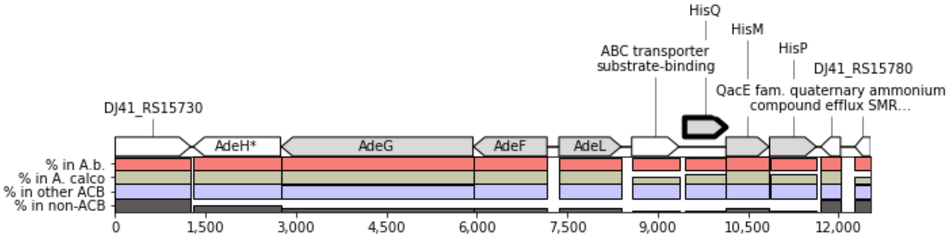

ESGC_0396 Length: 2

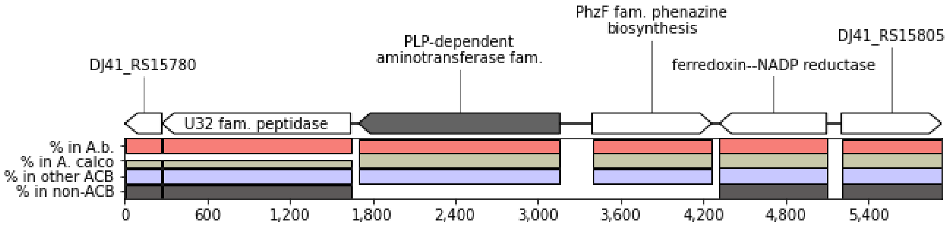

ESGC_0407 Length: 4

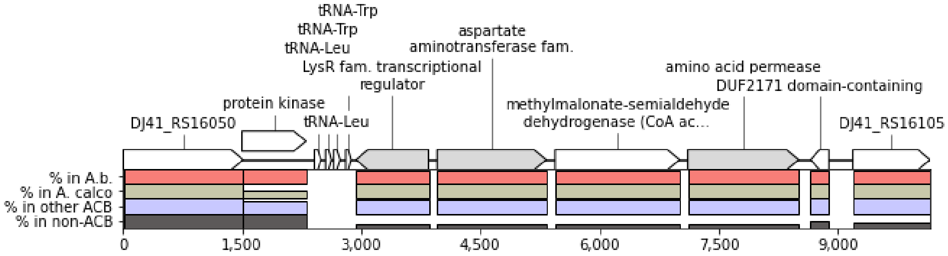

ESGC_0408 Length: 7

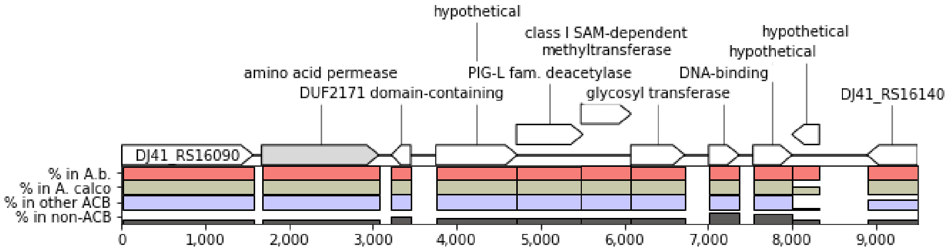

ESGC_0409 Length: 3

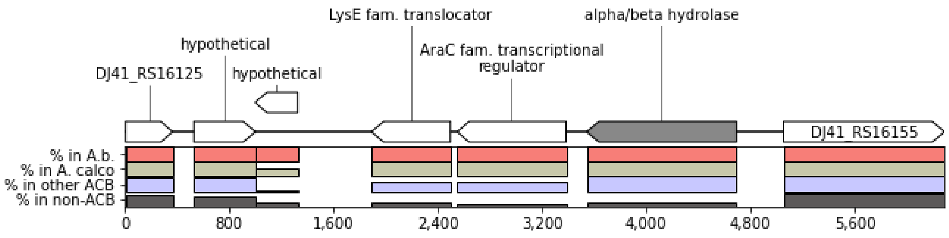

ESGC_0410 Length: 8

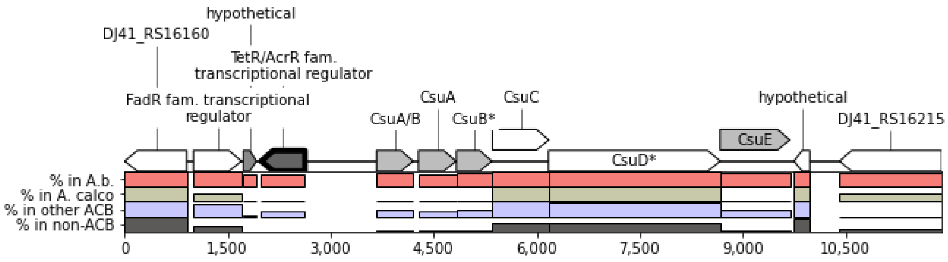

ESGC_0411 Length: 2

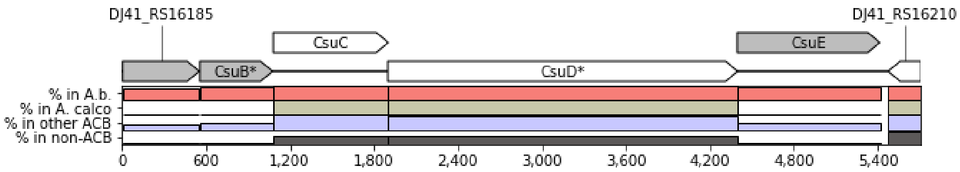

ESGC_0414 Length: 6

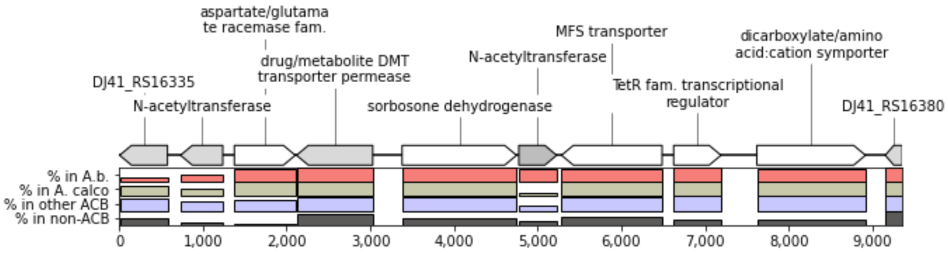

ESGC_0418 Length: 4

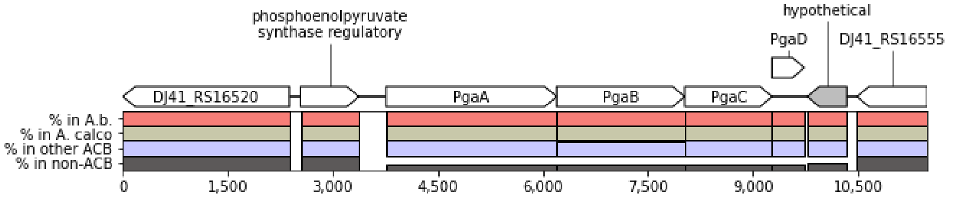

ESGC_0425 Length: 3

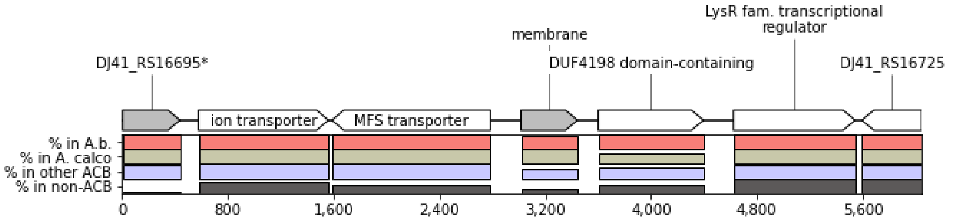

ESGC_0429 Length: 3

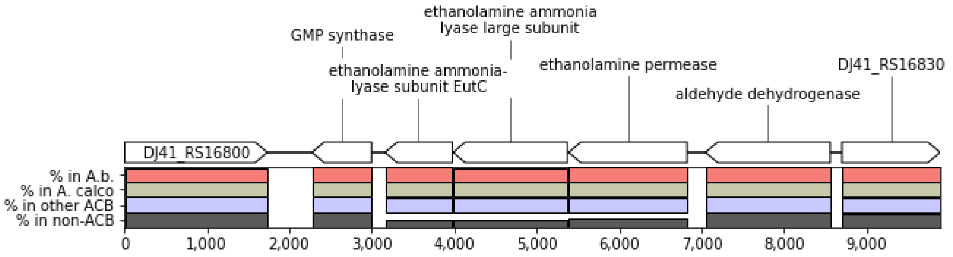

ESGC_0431 Length: 2

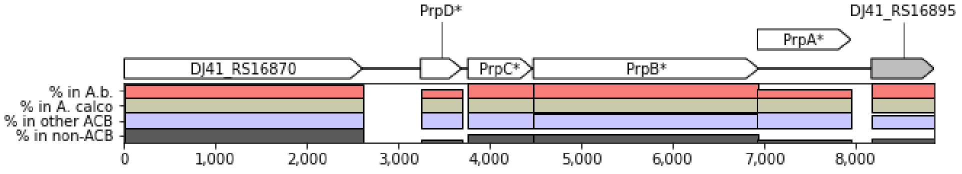

ESGC_0432 Length: 3

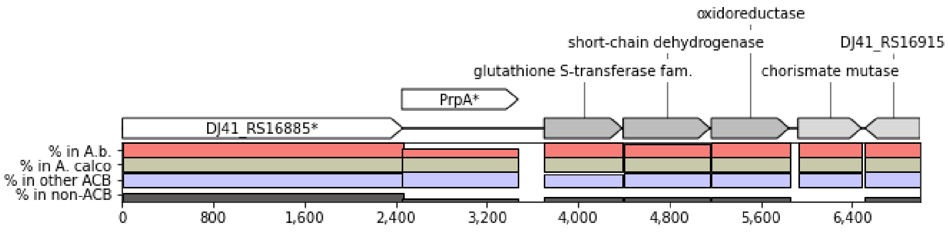

ESGC_0433 Length: 9

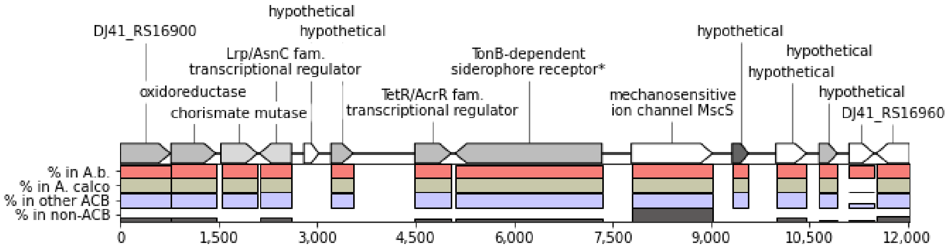

ESGC_0434 Length: 2

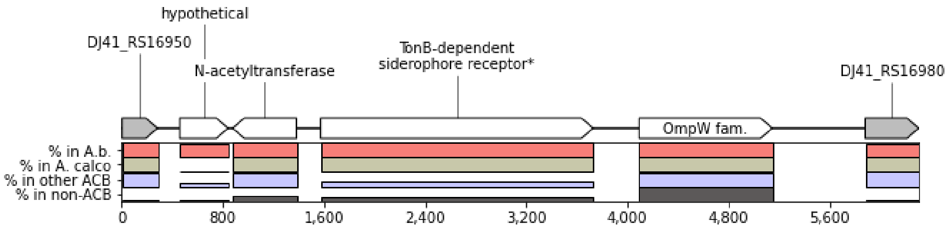

ESGC_0438 Length: 2

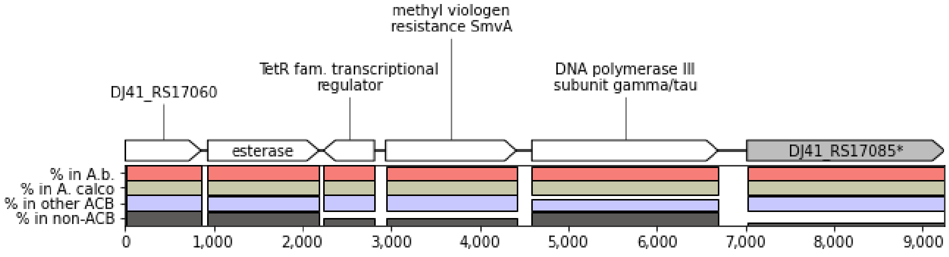

ESGC_0445 Length: 3

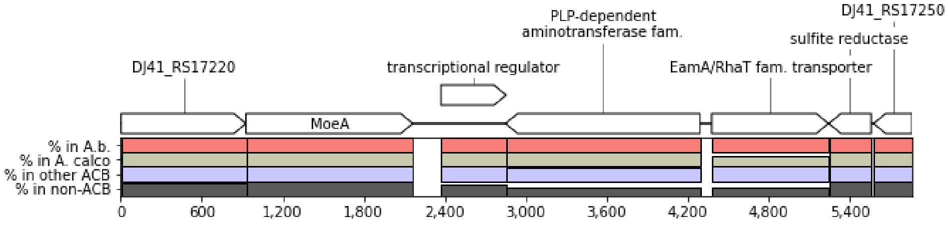

ESGC_0447 Length: 3

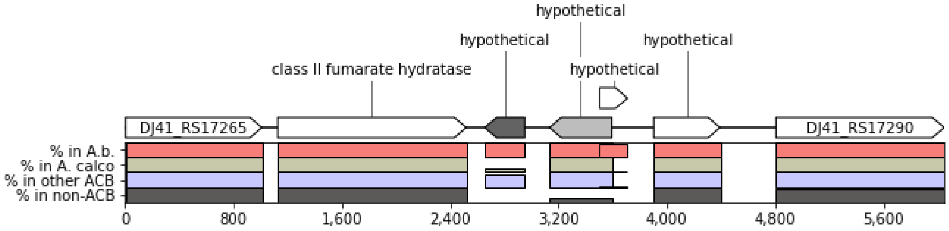

ESGC_0451 Length: 3

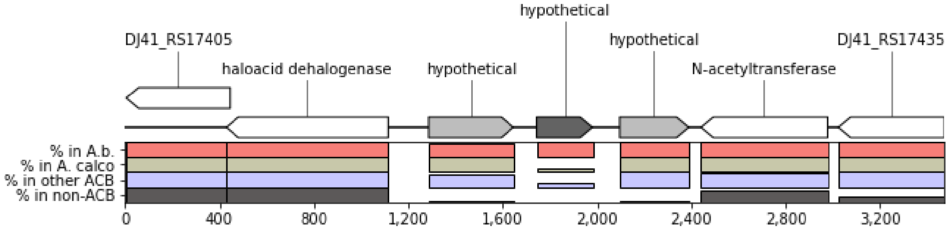

ESGC_0452 Length: 5

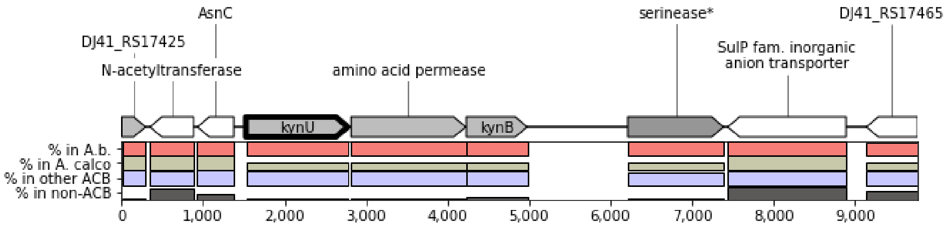

ESGC_0453 Length: 3

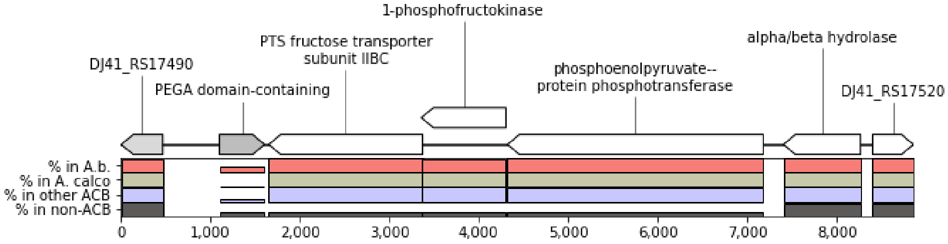

ESGC_0467 Length: 13

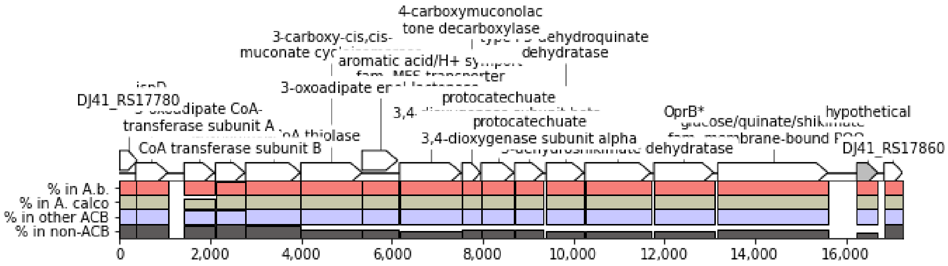

ESGC_0469 Length: 14

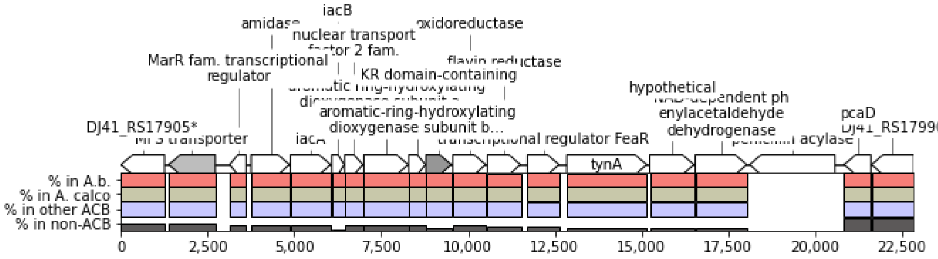

ESGC_0471 Length: 3

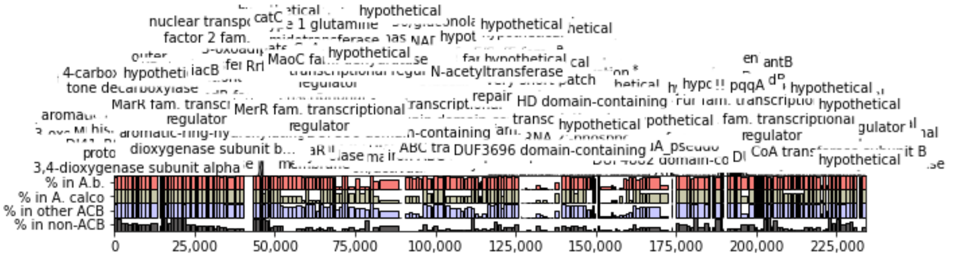

ESGC_0473 Length: 4

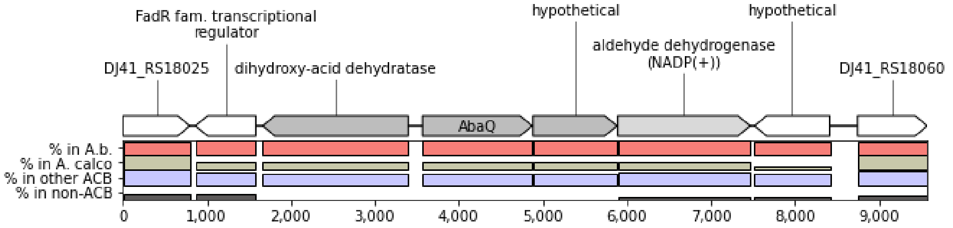

ESGC_0474 Length: 10

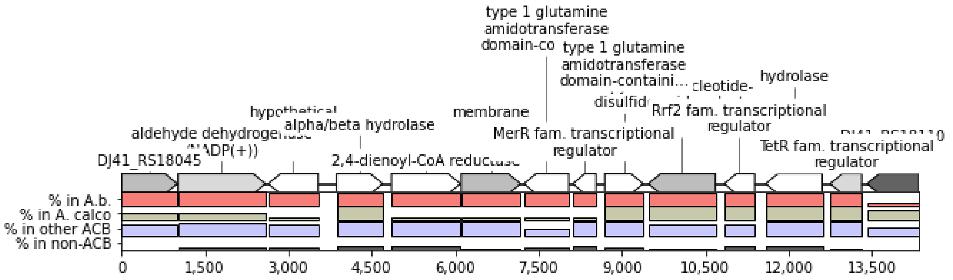

ESGC_0476 Length: 2

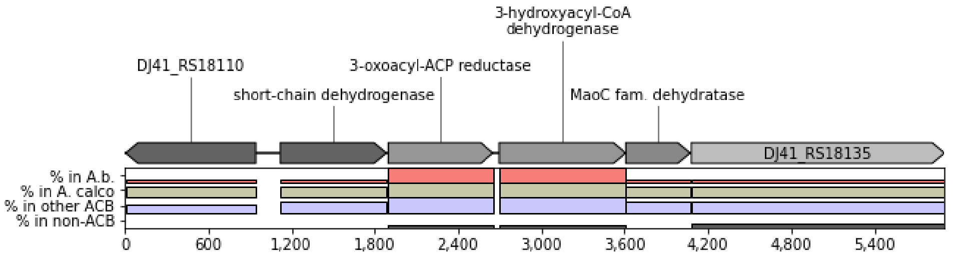

ESGC_0480 Length: 4

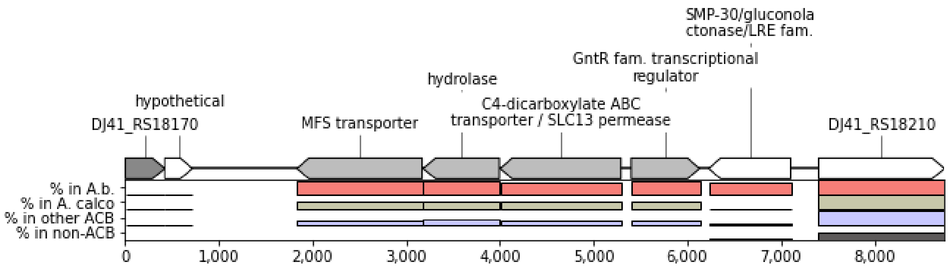

ESGC_0481 Length: 3

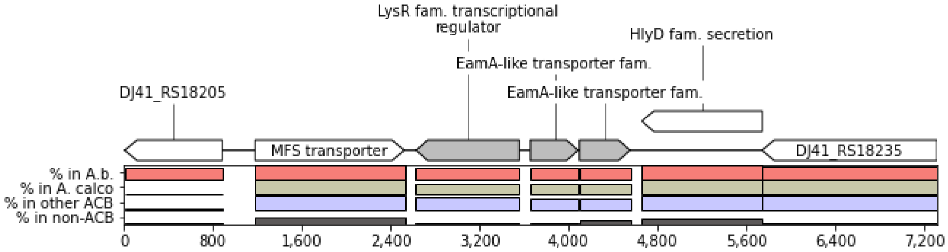

ESGC_0482 Length: 3

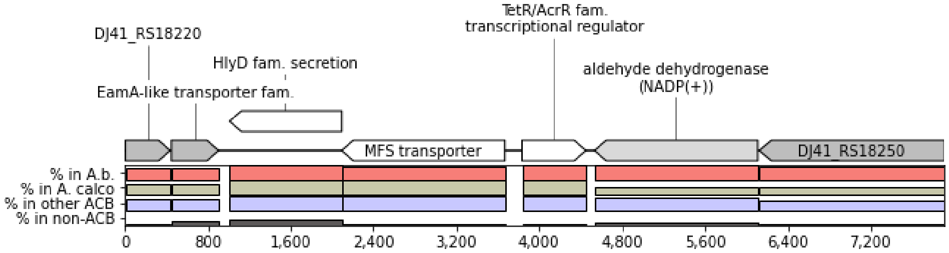

ESGC_0483 Length: 6

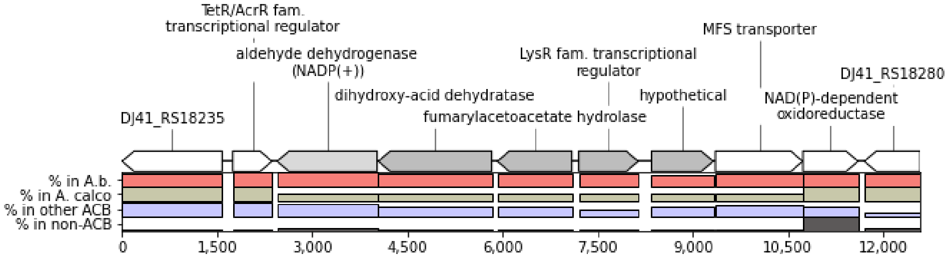

ESGC_0484 Length: 4

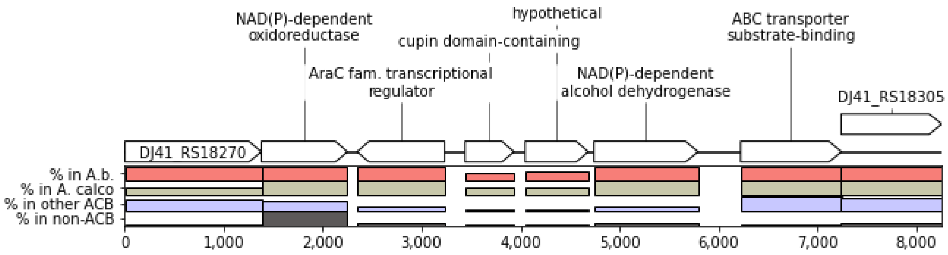

ESGC_0485 Length: 5

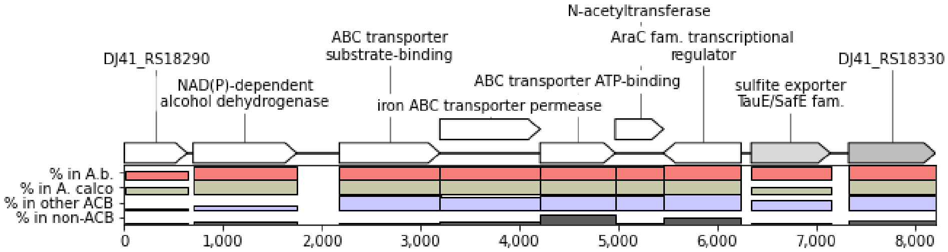

ESGC_0486 Length: 3

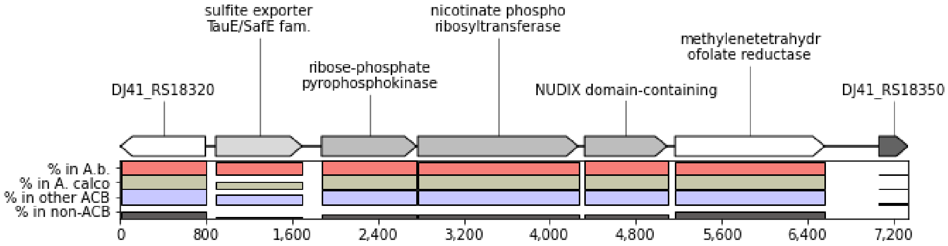

ESGC_0490 Length: 4

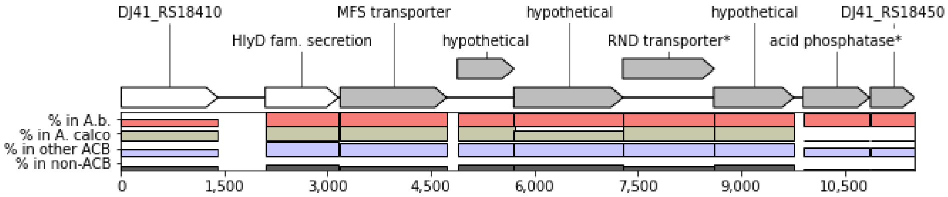

ESGC_0491 Length: 2

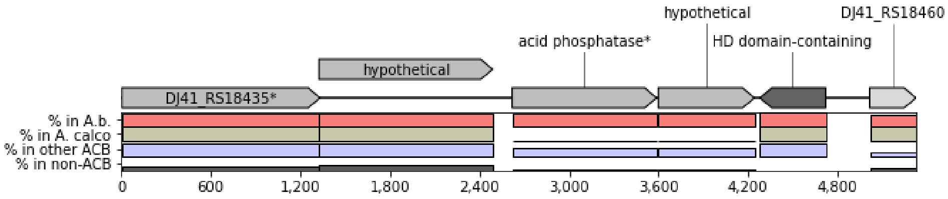

ESGC_0493 Length: 4

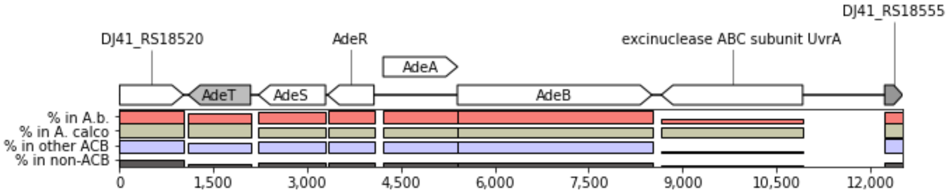

ESGC_0495 Length: 3

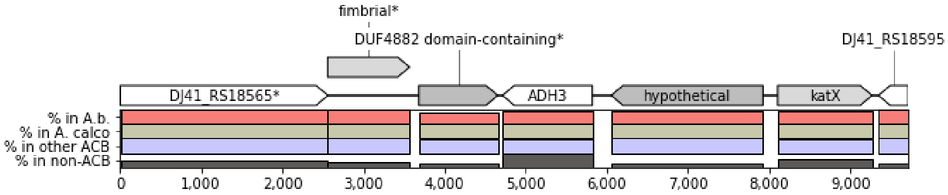

ESGC_0497 Length: 6

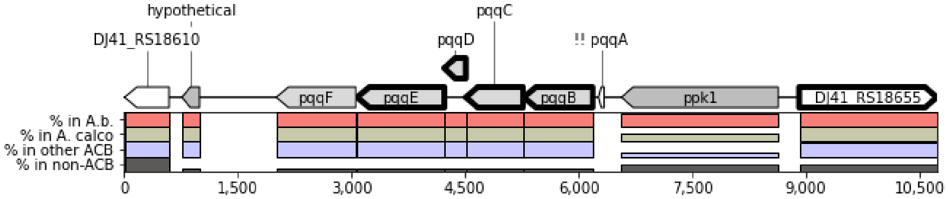

ESGC_0498 Length: 3

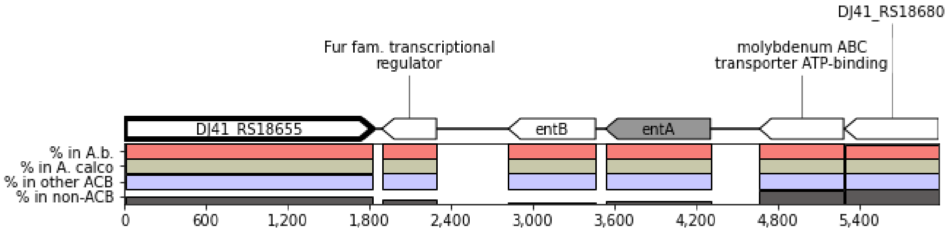

ESGC_0500 Length: 13

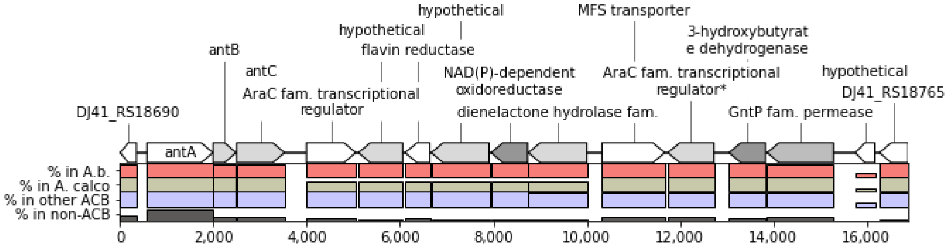

ESGC_0503 Length: 2

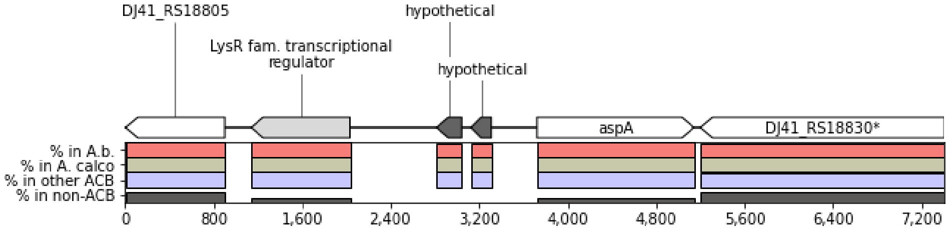

ESGC_0505 Length: 7

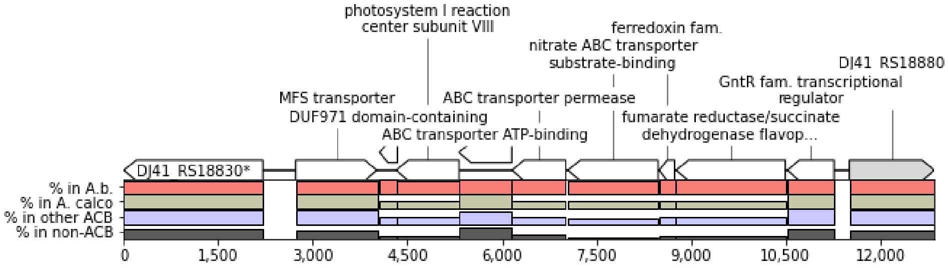

ESGC_0507 Length: 16

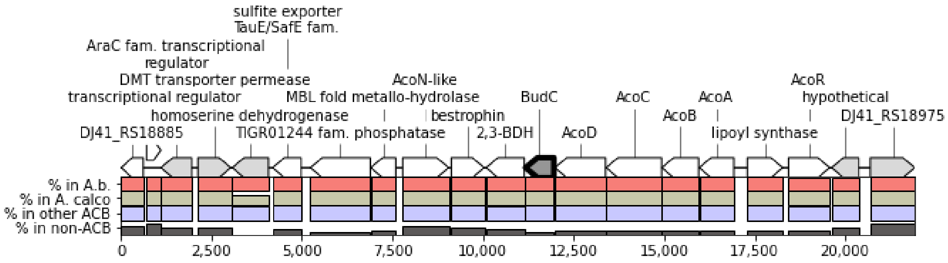

ESGC_0517 Length: 11

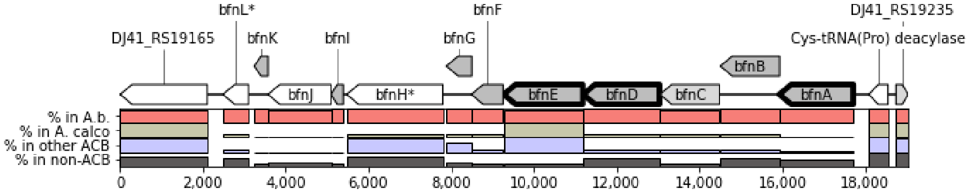

ESGC_0524 Length: 12

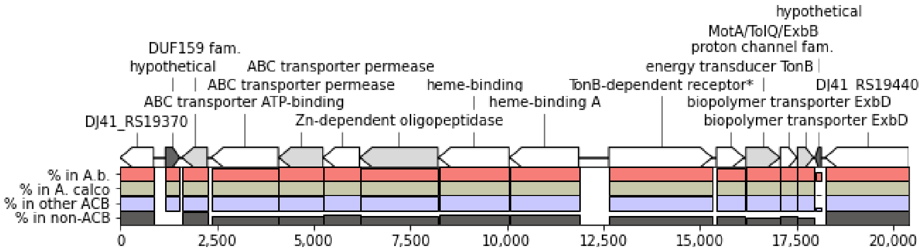

ESGC_0540 Length: 3

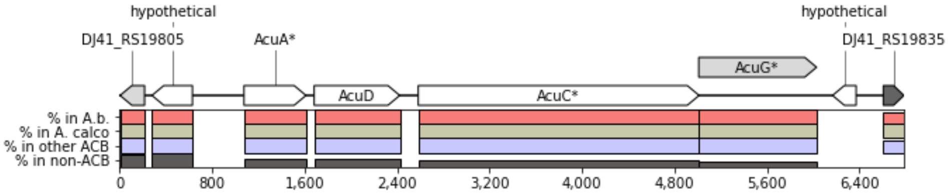

ESGC_0541 Length: 7

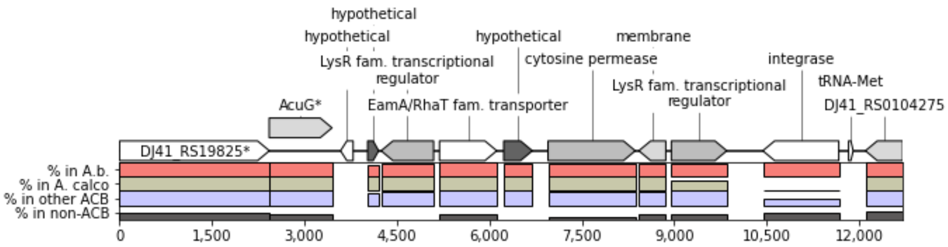

ESGC_0546 Length: 2

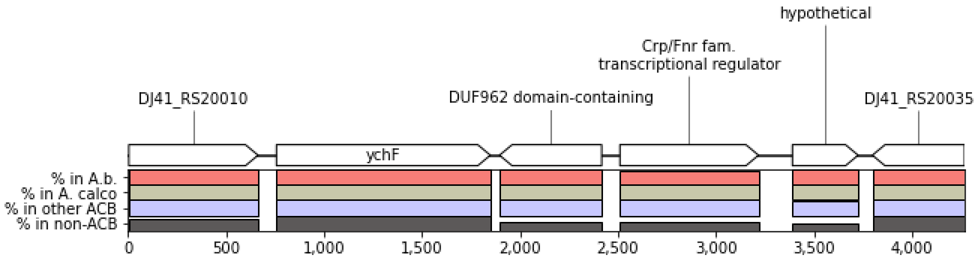

ESGC_0547 Length: 2

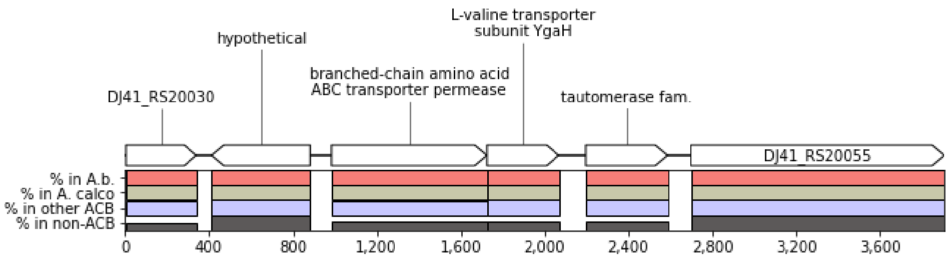

ESGC_0549 Length: 2

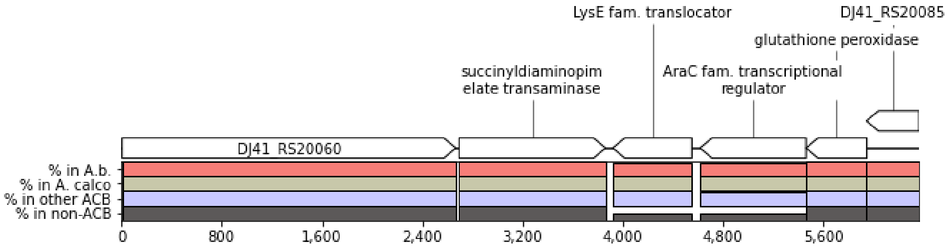

ESGC_0552 Length: 2

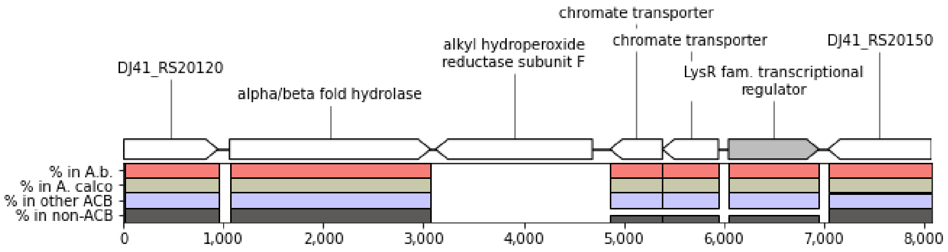

ESGC_0554 Length: 4

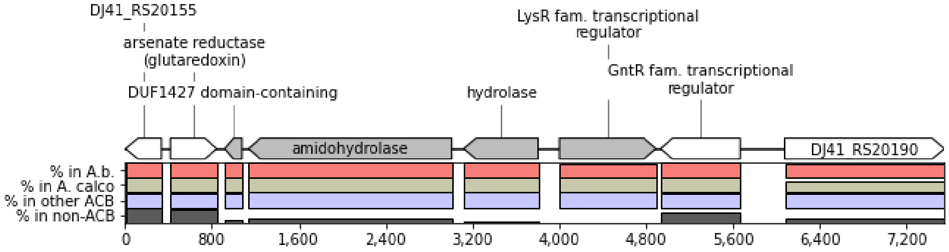

ESGC_0555 Length: 4

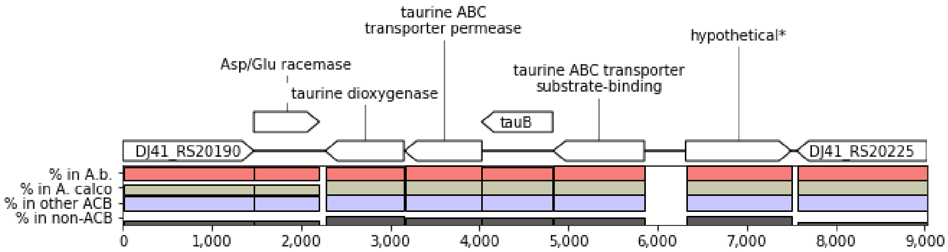

ESGC_0556 Length: 9

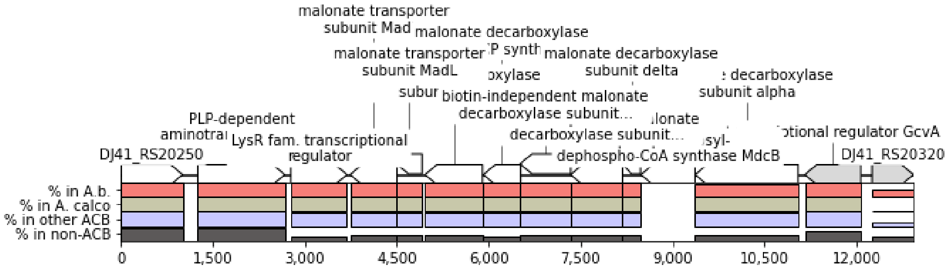

ESGC_0558 Length: 3

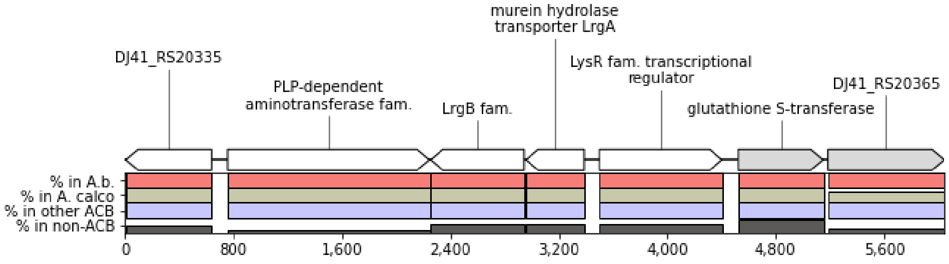

ESGC_0559 Length: 4

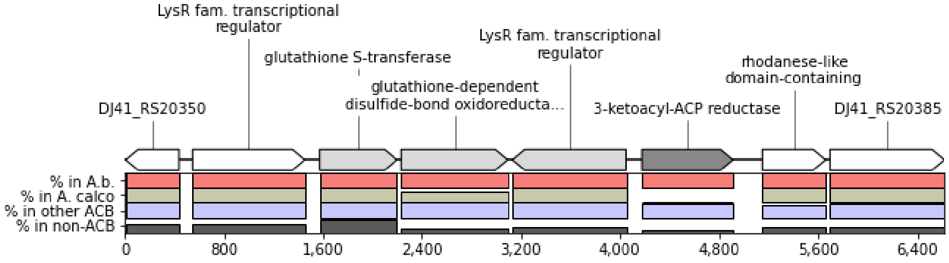

ESGC_0560 Length: 4

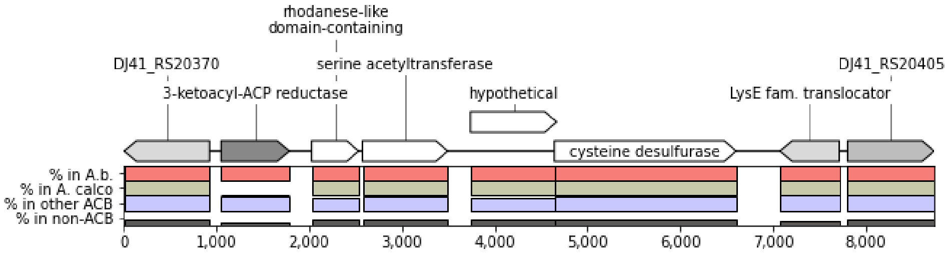

ESGC_0561 Length: 7

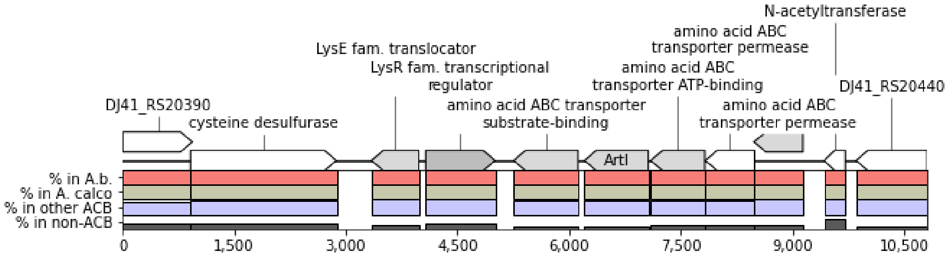

ESGC_0562 Length: 3

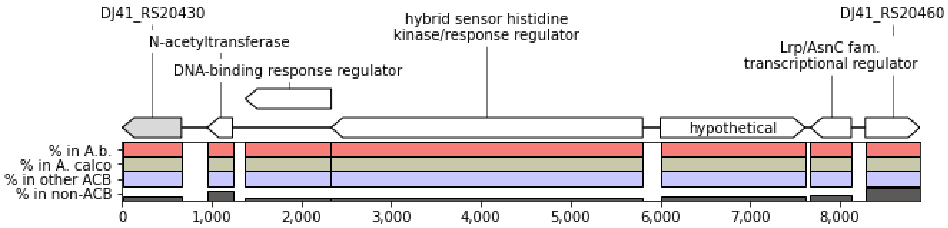

ESGC_0566 Length: 8

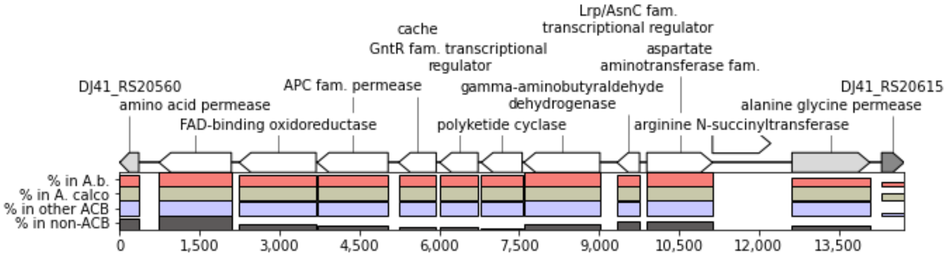

ESGC_0568 Length: 8

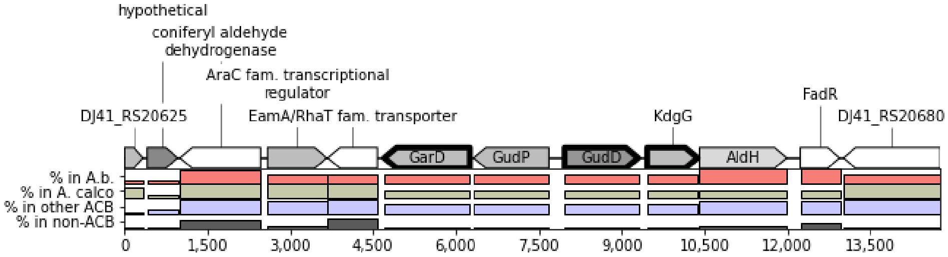

ESGC_0569 Length: 16

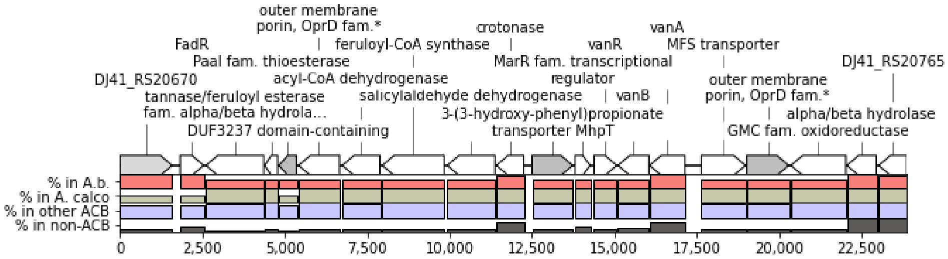

ESGC_0571 Length: 2

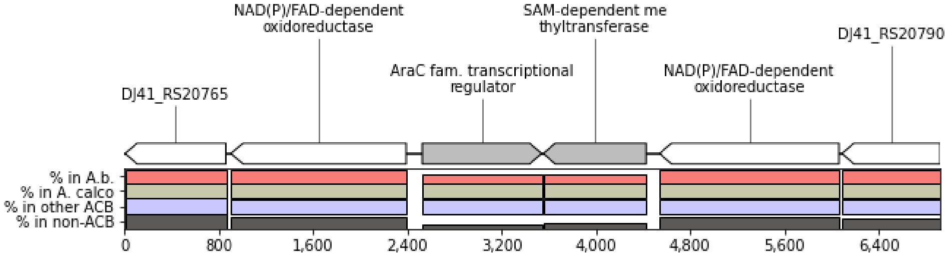

ESGC_0586 Length: 2

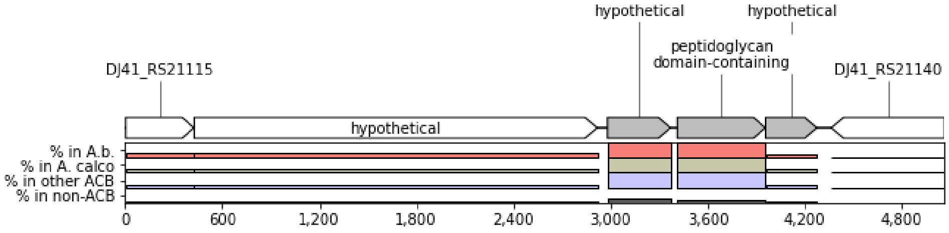

ESGC_0593 Length: 2

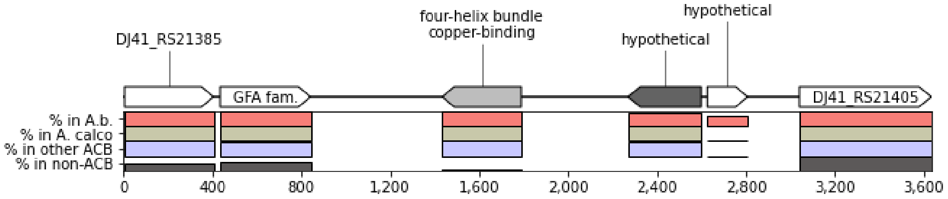

ESGC_0594 Length: 4

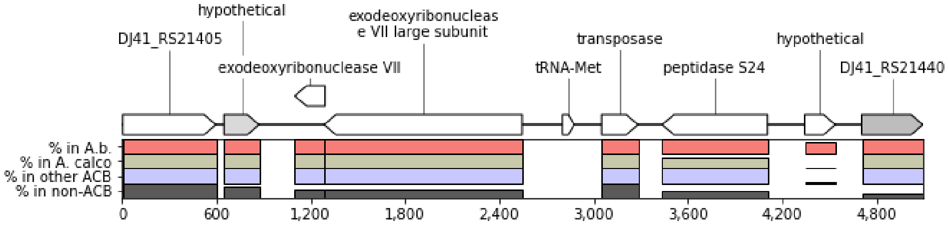

ESGC_0595 Length: 2

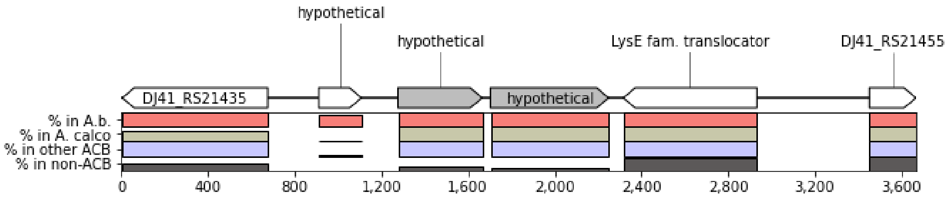

ESGC_0597 Length: 4

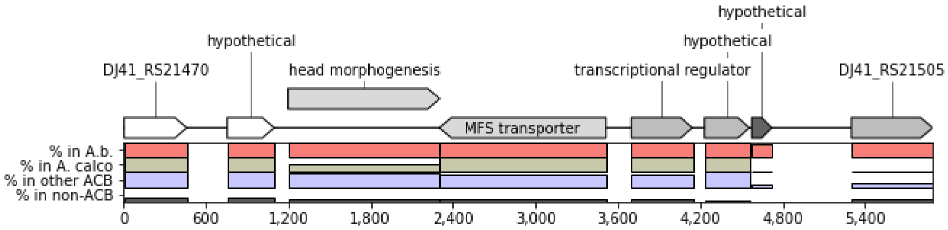

ESGC_0598 Length: 3

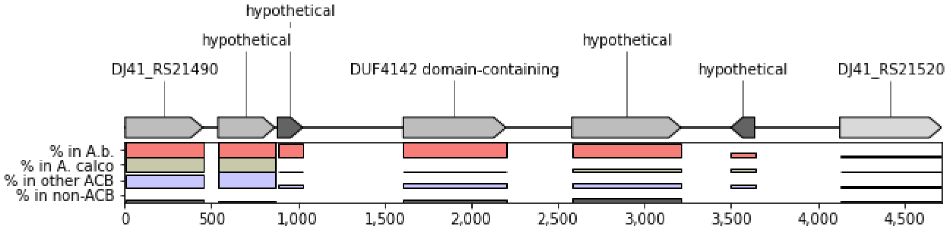

ESGC_0608 Length: 3

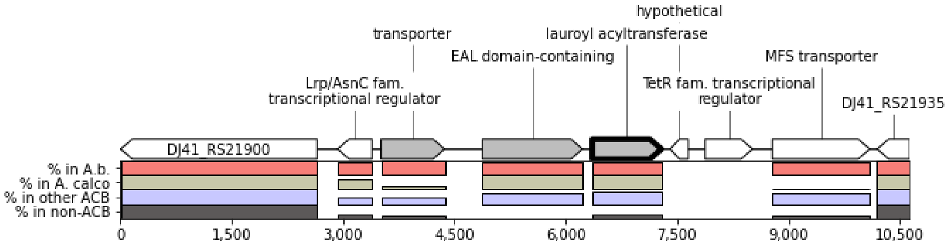

ESGC_0610 Length: 3

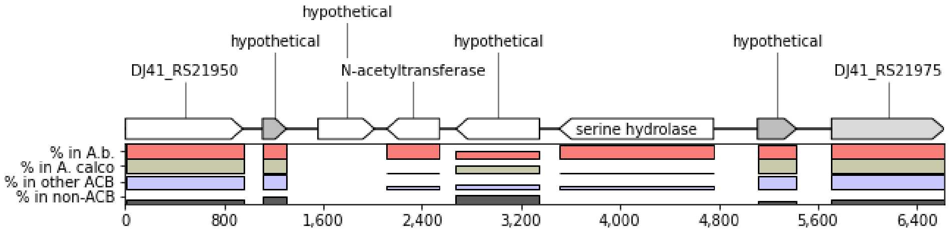

ESGC_0611 Length: 6

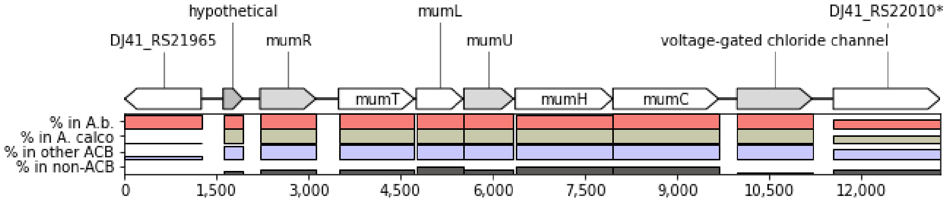

ESGC_0618 Length: 4

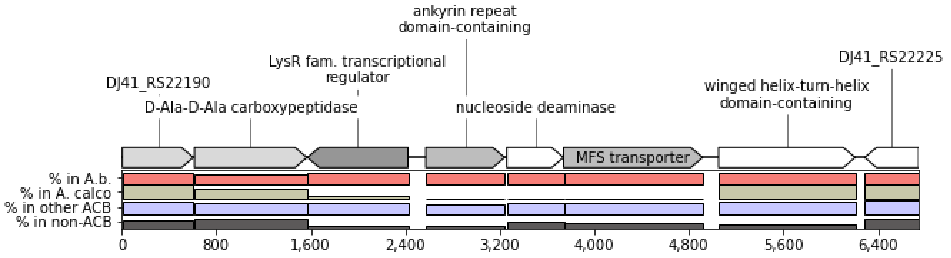

ESGC_0621 Length: 9

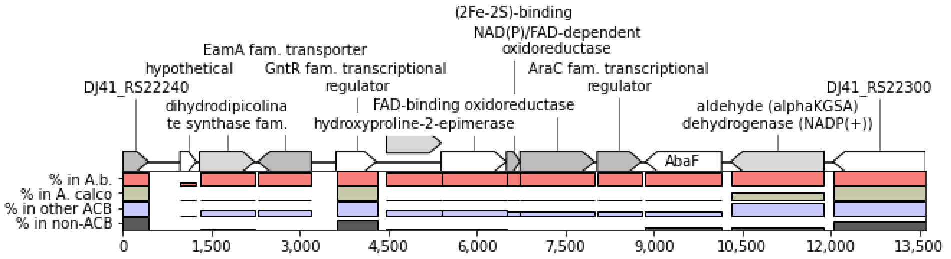

ESGC_0622 Length: 15

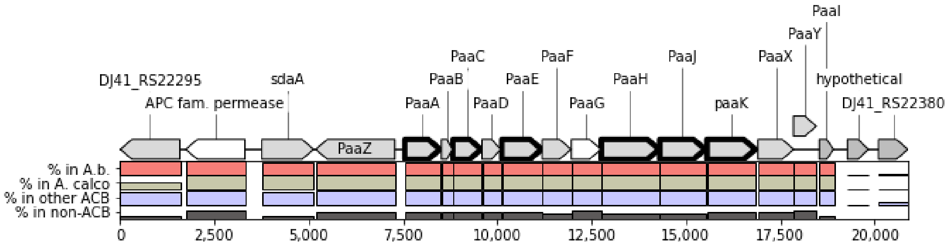

ESGC_0624 Length: 3

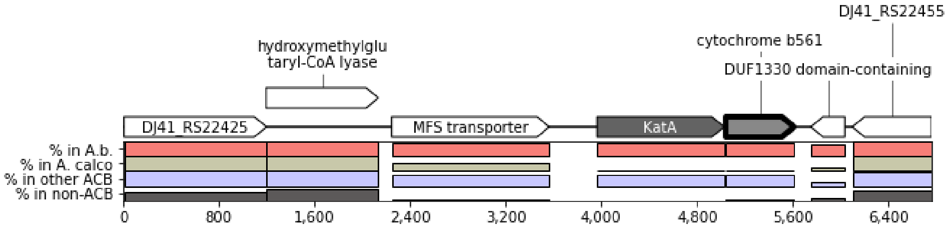

ESGC_0625 Length: 11

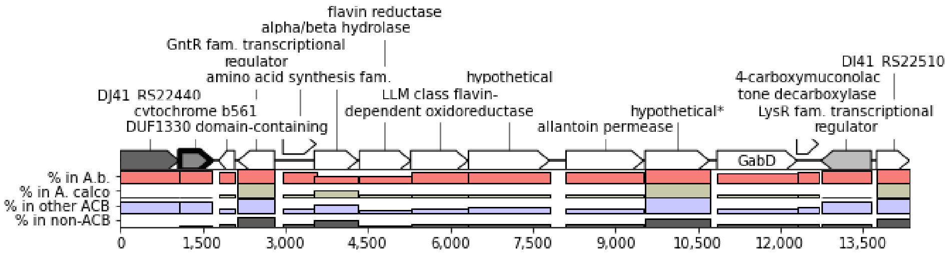

ESGC_0626 Length: 2

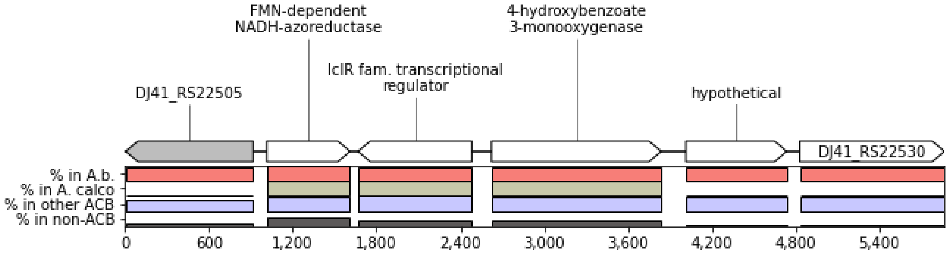

ESGC_0627 Length: 7

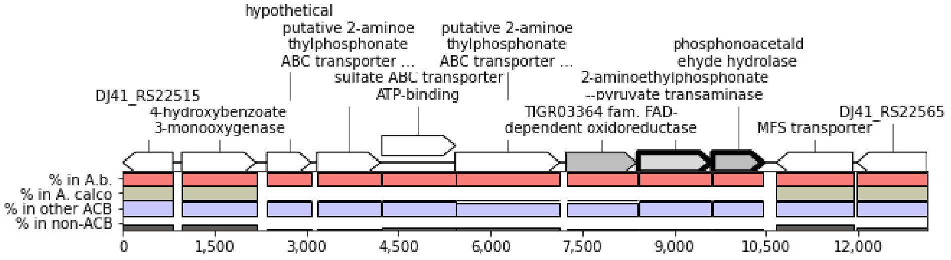

ESGC_0628 Length: 20

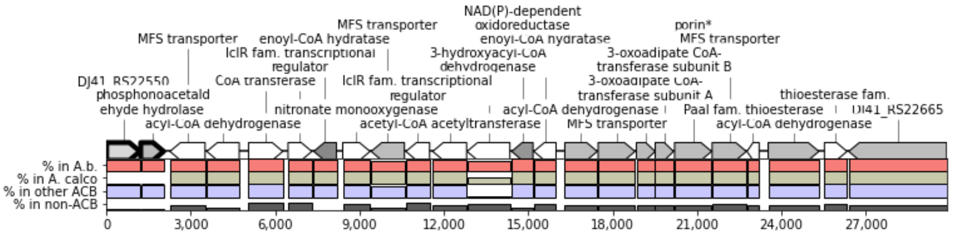

ESGC_0629 Length: 2

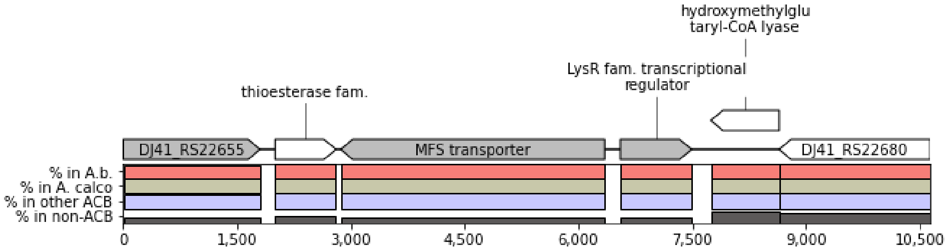

ESGC_0632 Length: 3

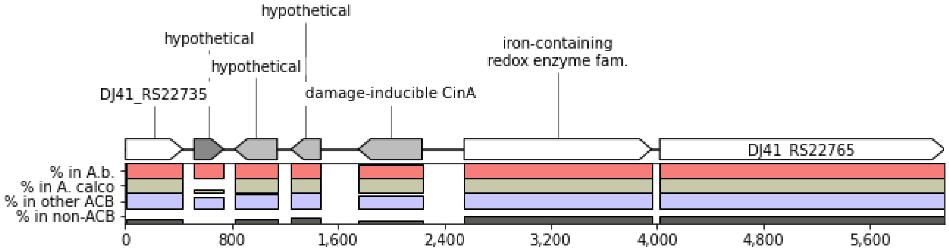

ESGC_0633 Length: 2

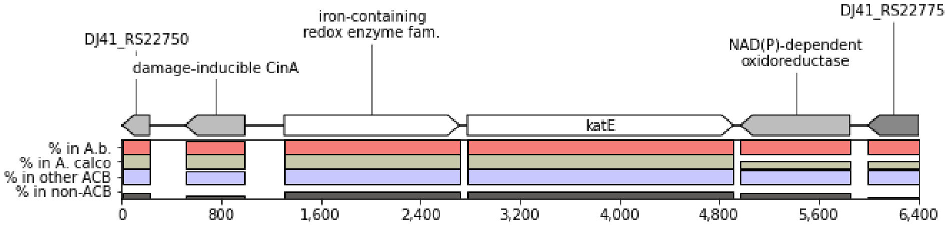

ESGC_0634 Length: 2

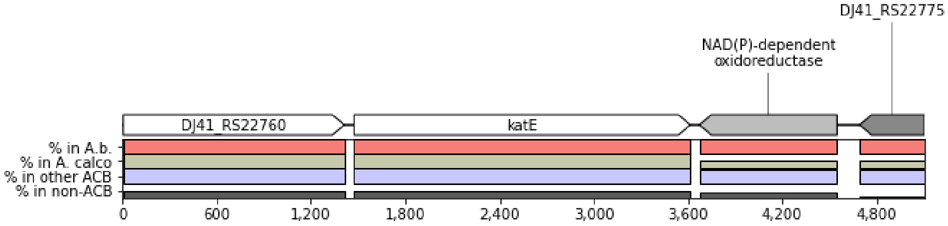

Supplement: S5 Fig — Graphical representations of the genomic regions for each ESGC with RD > 0 (see Methods) along the genome of ATCC 19606 with abbreviated abundance profiles and functional annotations. (PDF) [file pgen.1010020.s006.pdf]
