# Supplementary material for: Evolutionarily stable gene clusters shed light on the common grounds of pathogenicity in the Acinetobacter calcoaceticus-baumannii complex
Source: PLoS Genet. 2022 Jun 2;18(6):e1010020. doi: 10.1371/journal.pgen.1010020 (PMC9162365; doi:10.1371/journal.pgen.1010020)
Supplement: S7 Fig — (PDF) [file pgen.1010020.s008.pdf]

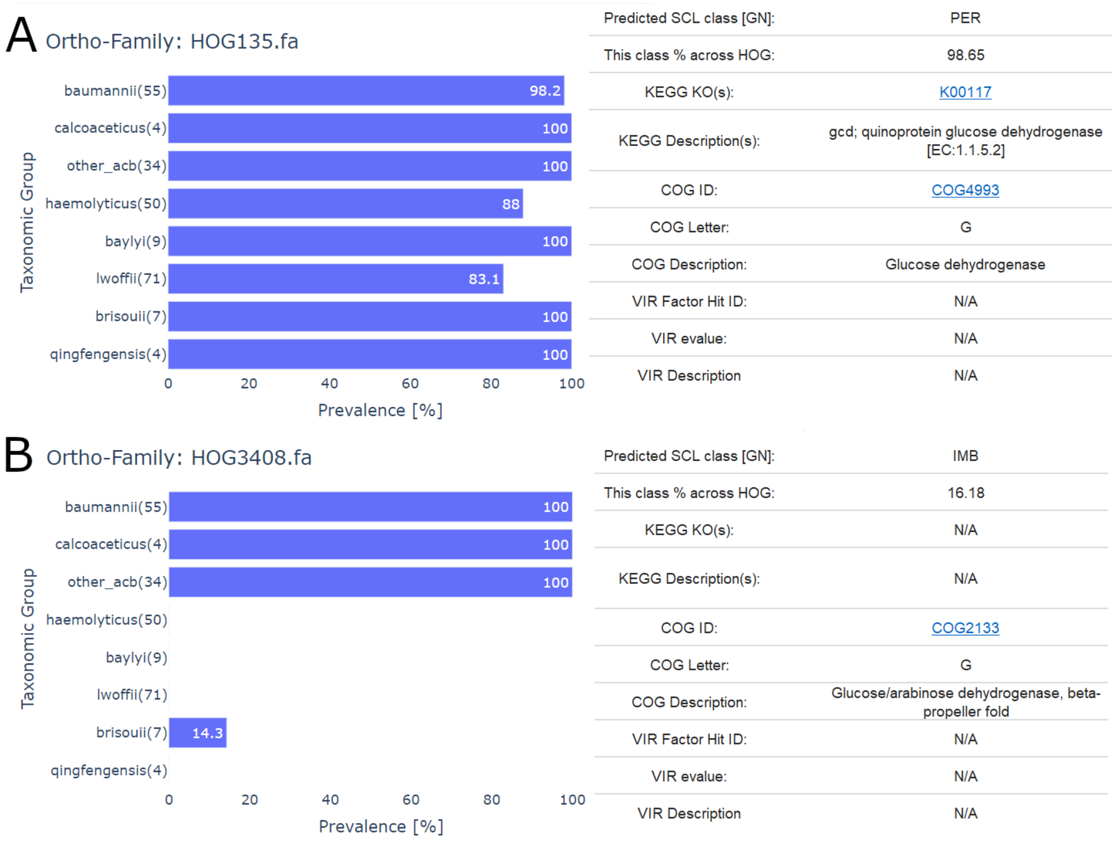

Supplementary Figure F3: Abundance of the two glucose dehydrogenase encoding genes in A. baumannii ATCC 19606. The abundance pattern and annotations of the almost ubiquitously present membrane-bound gdhA (WP_001075590.1). B) The abundance pattern and annotations of the soluble gdhB (WP_001032867.1). This gene is, with a sole exception, confined to the ACB clade. The plots were derived from Aci-Dash (https://aci-dash.ingress.rancher.computational.bio)
