# Supplementary material for: Evolutionarily stable gene clusters shed light on the common grounds of pathogenicity in the Acinetobacter calcoaceticus-baumannii complex
Source: PLoS Genet. 2022 Jun 2;18(6):e1010020. doi: 10.1371/journal.pgen.1010020 (PMC9162365; doi:10.1371/journal.pgen.1010020)
Supplement: S11 Fig — (PDF) [file pgen.1010020.s012.pdf]

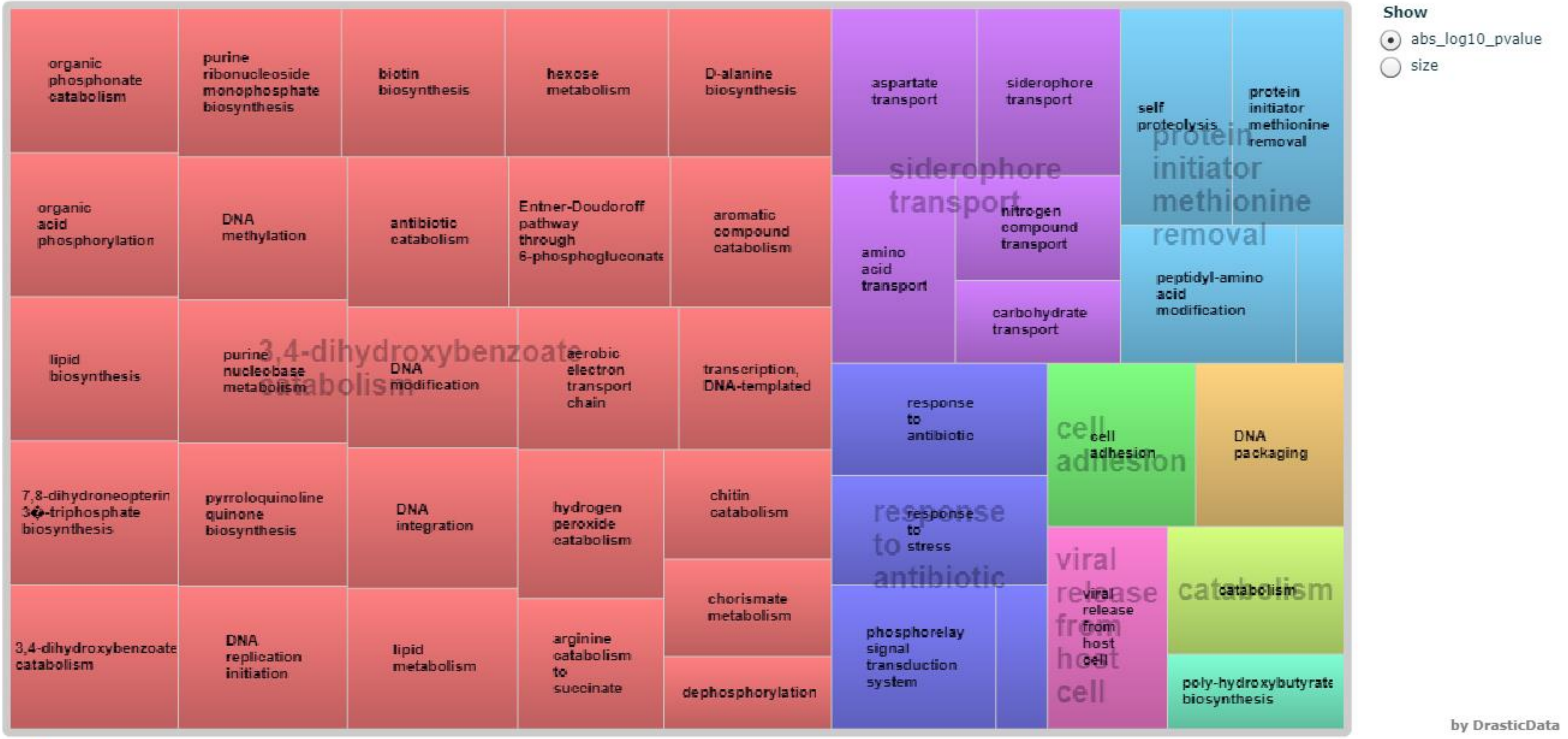

Supplementary Figure D2.1 – Revigo plot of enriched biological processes at the node ACB+BR

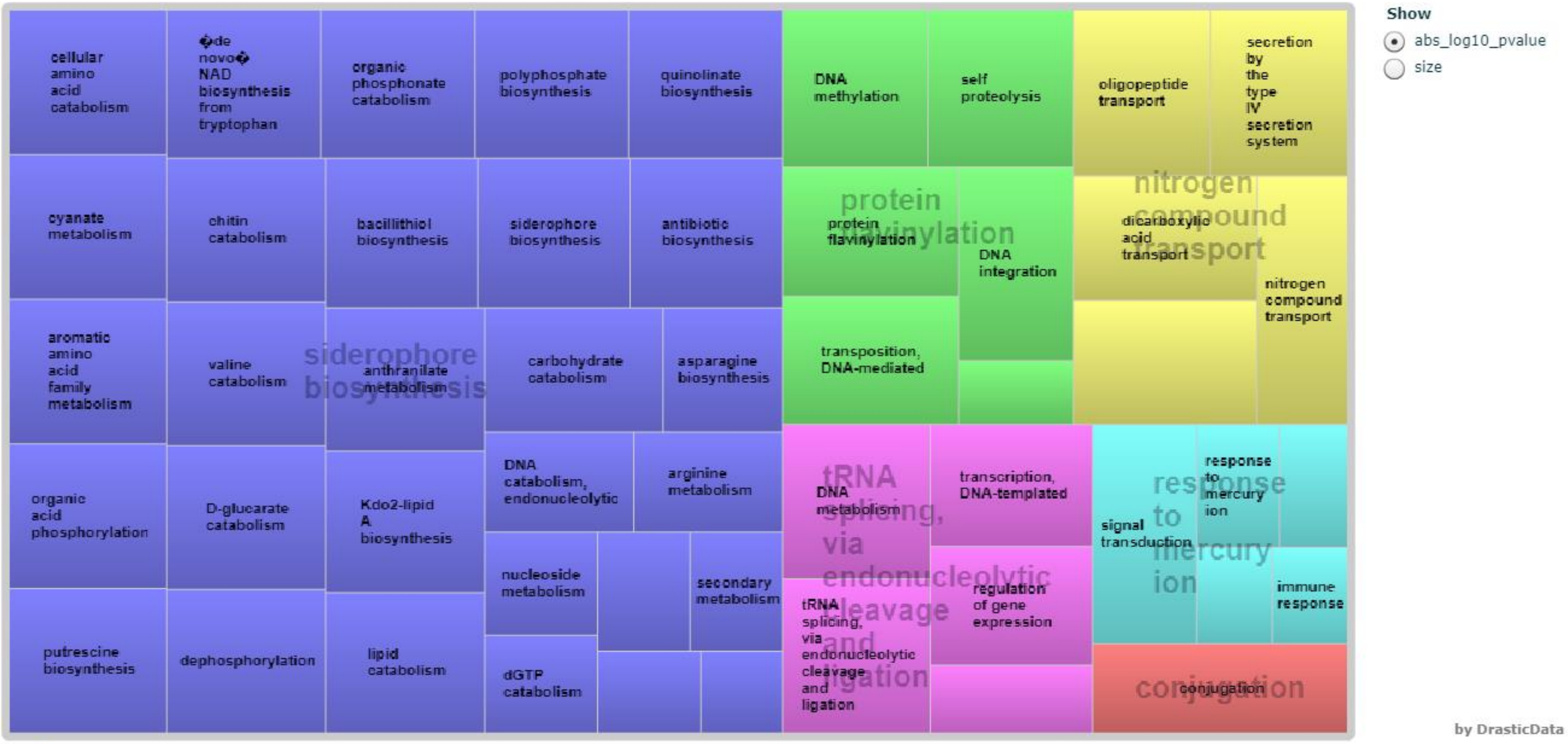

Supplementary Figure D2.2 – Revigo plot of enriched biological processes at the node ACB+LW

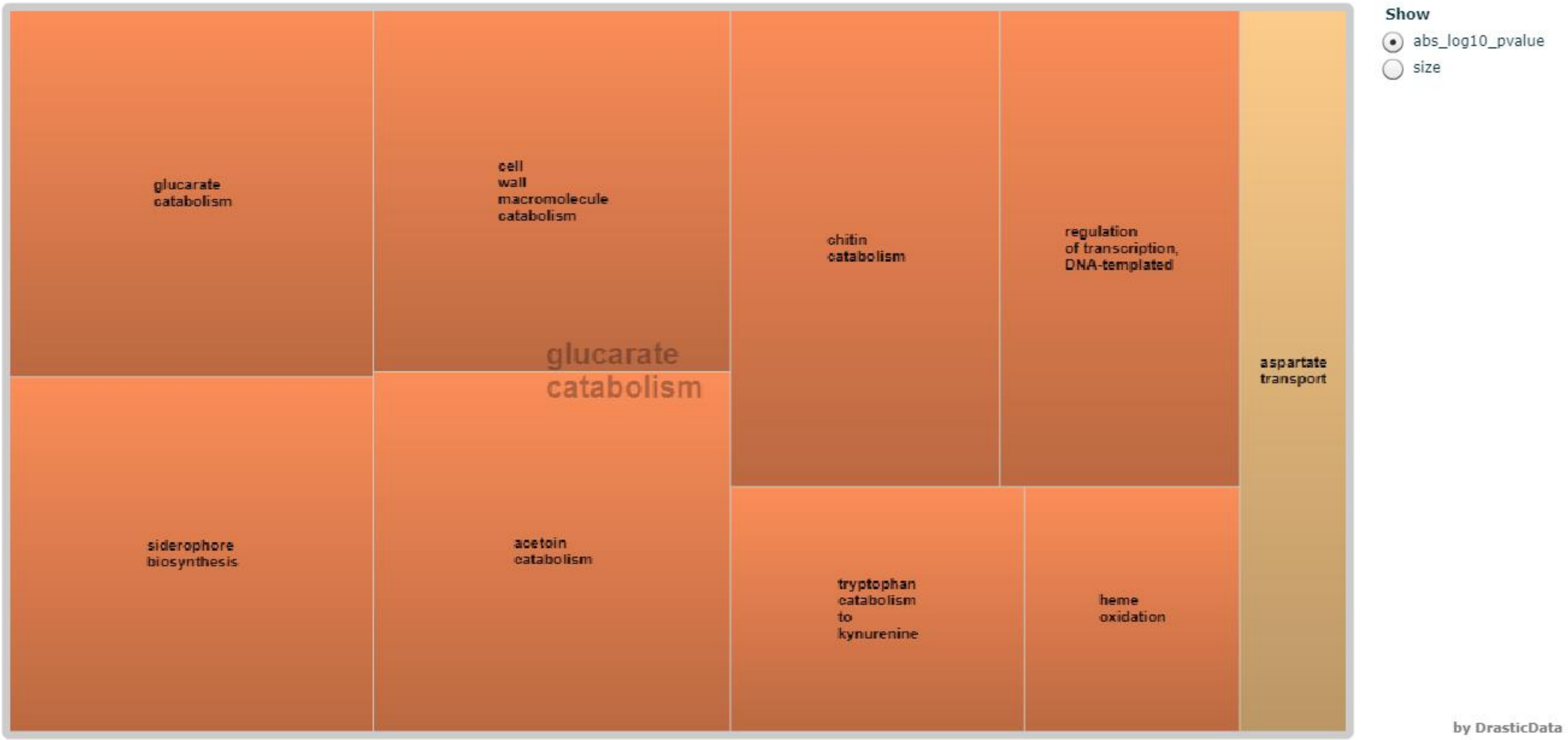

Supplementary Figure D2.3 – Revigo plot of enriched biological processes at the node ACB+BA

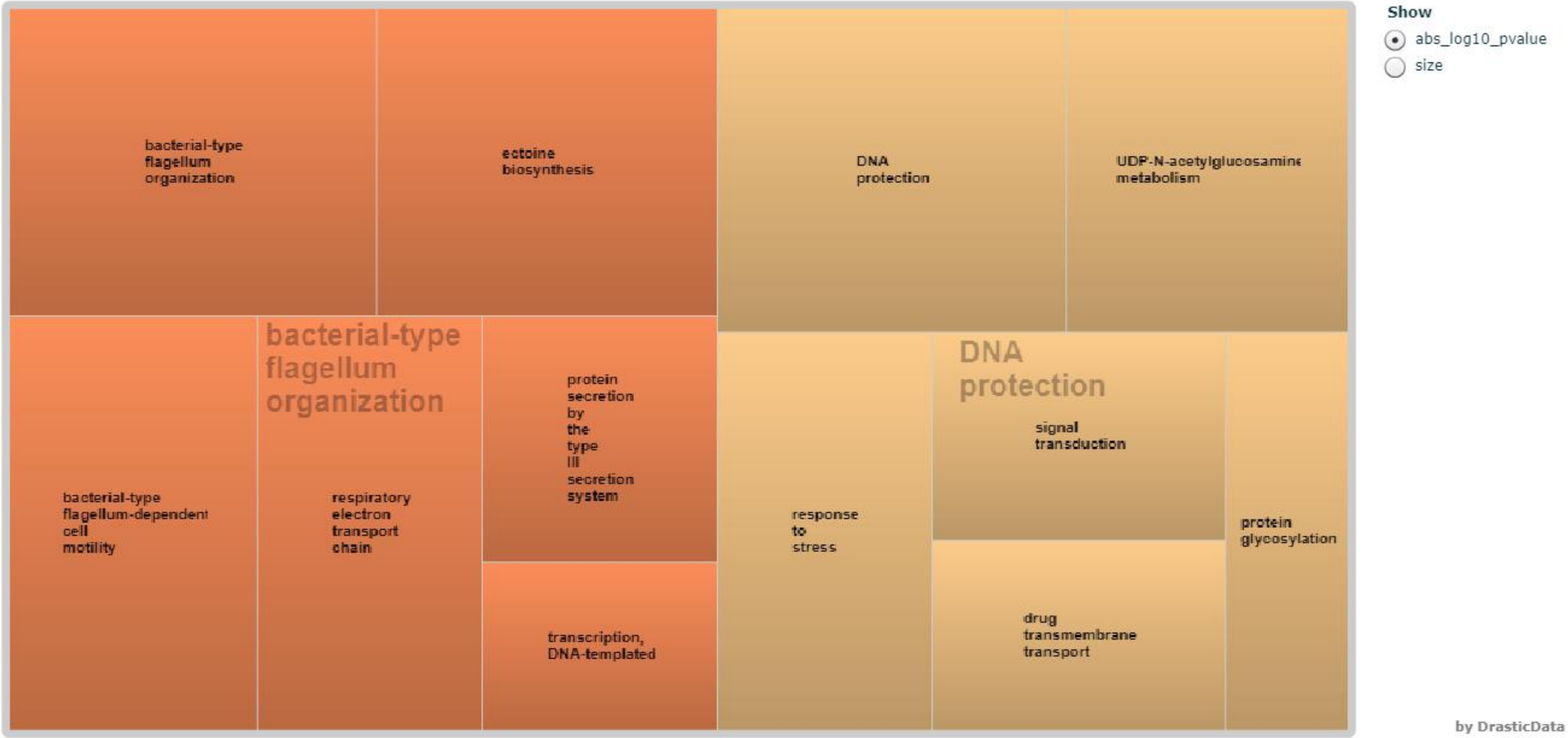

Supplementary Figure D2.4 – Revigo plot of enriched biological processes at the node ACB+HA

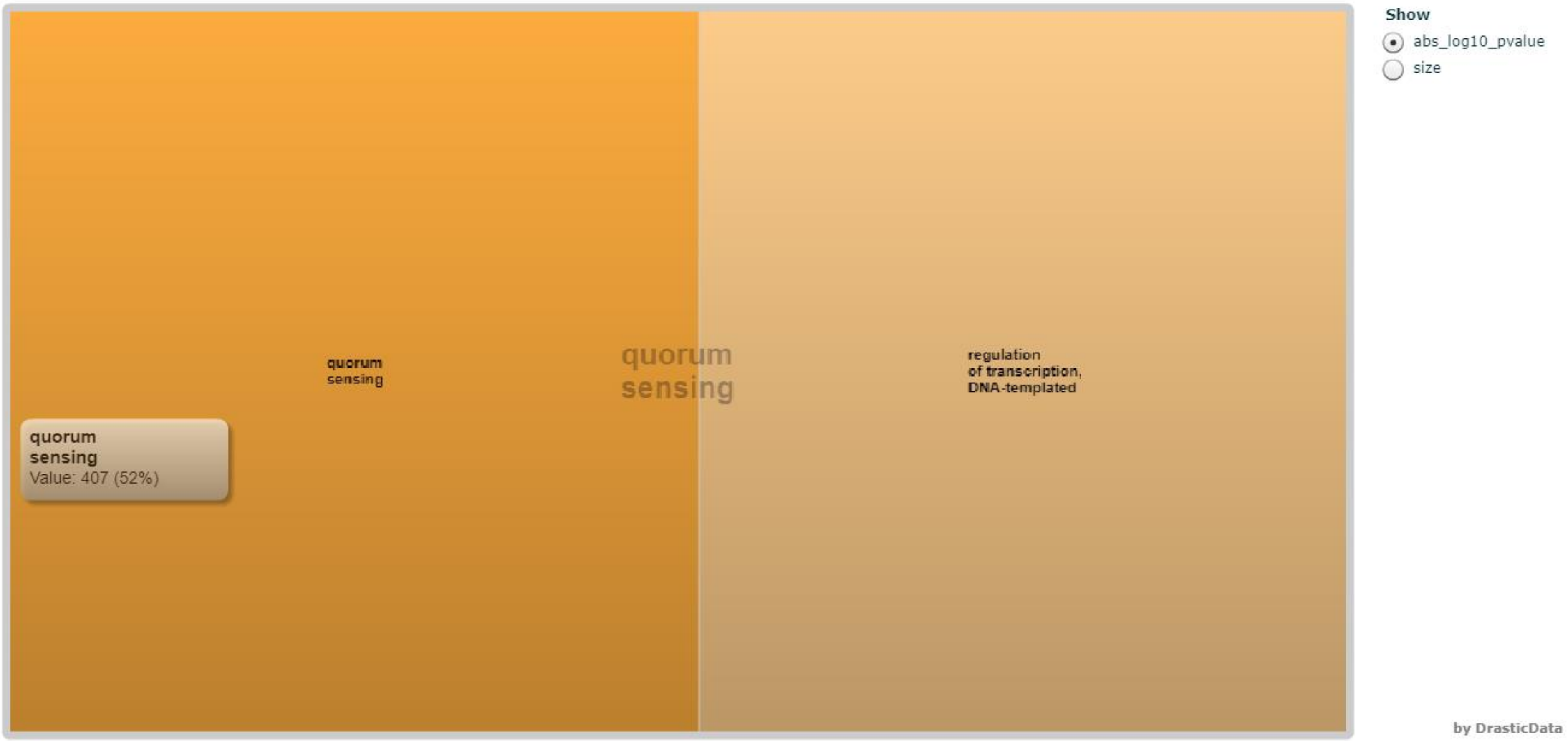

Supplementary Figure D2.5 – Revigo plot of enriched biological processes at the node ACB
