# Supplementary material for: Evolutionarily stable gene clusters shed light on the common grounds of pathogenicity in the Acinetobacter calcoaceticus-baumannii complex
Source: PLoS Genet. 2022 Jun 2;18(6):e1010020. doi: 10.1371/journal.pgen.1010020 (PMC9162365; doi:10.1371/journal.pgen.1010020)
Supplement: S2 Data — (TAR.GZ) [file pgen.1010020.s014.tar.gz › Data_S2/ESGC_0452_Proteobacteria.html]

```
(R)Acinetobacter baumannii  19606 | .(2576) <kynR kynU> aa perm.> kynB> serin prot.> .(1064) |
Acinetobacter calcoaceticus CA12  | .(1629) <kynR .(1)... aa perm.> kynB> .(1)... serin prot.> .(2142) |
Acinetobacter lactucae        	| .(1481) <kynR kynU> aa perm.> kynB> .(1)... serin prot.> .(2090) |
Acinetobacter nosocomialis M2 	| .(3293) <kynR kynU> aa perm.> kynB> serin prot.> .(151). |
Acinetobacter pittii PHEA-2   	| .(2160) <kynR kynU> aa perm.> kynB> serin prot.> .(1434) |
Acinetobacter seifertii       	| .(1524) <kynR kynU> aa perm.> kynB> .(2113) |
Acinetobacter baylyi ADP1     	|
Acinetobacter chinensis       	|
Acinetobacter cumulans        	|
Acinetobacter defluvii        	|
Acinetobacter dispersus       	|
Acinetobacter equi            	|
Acinetobacter guillouiae     	| .(1448) <kynR kynU> aa perm.> .(2690) |
Acinetobacter haemolyticus    	| .(971). <serin prot. .(2070) |
Acinetobacter junii           	| .(990). <serin prot. .(1953) |
Acinetobacter larvae          	|
Acinetobacter lanii           	|
Acinetobacter oleivorans DR1  	| .(1564) <kynR kynU> aa perm.> kynB> .(1)... serin prot.> .(2244) |
Acinetobacter schindleri      	|
Acinetobacter shaoyimingii    	|
Acinetobacter wanghuae        	|

Moraxella bovoculi            	|
Moraxella bovis               	|
Moraxella catarrhalis BBH18   	|
Moraxella cuniculi            	|
Moraxella nonliquefaciens     	|
Moraxella osloensis           	|
Moraxella ovis                	|
Psychrobacter alimentarius    	|
Psychrobacter arcticus 273-4  	| .(1195) kynR> .(907). |
Psychrobacter cryohalolentis..	| .(1145) <kynR .(1323) |
Psychrobacter urativorans     	|
Azotobacter chroococcum       	| .(1478) <serin prot. .(2617) |
Azotobacter salinestris       	| .(2884) <serin prot. .(345). <kynR .(1158) |
Azotobacter vinelandii DJ     	| .(883). <kynR .(3256) serin prot.> .(555). |
Entomomonas moraniae          	|
Oblitimonas alkaliphila       	|
Permianibacter aggregans      	| .(1727) serin prot.> .(852). <kynU .(13).. <serin prot. .(148). serin prot.> .(1014) |
Pseudomonas aeruginosa PAO1H2O	| .(3388) <kynR .(1)... kynU> aa perm.> .(2007) |
Pseudomonas citronellolis     	| .(2012) kynR> .(4068) |
Pseudomonas furukawaii        	| .(1823) <kynR .(1)... kynU> .(1)... aa perm.> .(2219) <kynB .(1527) |
Pseudomonas pseudoalcaligene..	|
Pseudomonas mendocina S5.2    	|
Pseudomonas multiresinivorans 	|
Pseudomonas agarici           	| .(383). serin prot.> .(4335) |
Pseudomonas alkylphenolica    	|
Pseudomonas amygdali pv. tab..	|
Pseudomonas cichorii JBC1     	|
Pseudomonas fuscovaginae      	| .(758). <serin prot. .(4916) |
Pseudomonas syringae pv. tom..	|
Pseudomonas viridiflava       	| .(3516) serin prot.> .(1766) |
Pseudomonas antarctica        	| .(1617) <kynR kynU> aa perm.> .(3952) |
Pseudomonas arsenicoxydans    	| .(4126) <serin prot. .(1645) |
Pseudomonas asplenii          	| .(1730) <serin prot. .(3850) |
Pseudomonas azotoformans      	| .(1511) serin prot.> .(1225) <kynR kynU> aa perm.> .(3178) |
Pseudomonas brenneri          	| .(1722) <serin prot. .(3342) <kynR kynU> aa perm.> .(287). |
Pseudomonas cedrina           	| .(5296) <serin prot. .(489). |
Pseudomonas corrugata         	| .(3255) <serin prot. .(1985) |
Pseudomonas extremorientalis  	| .(2033) serin prot.> .(1140) <kynR kynU> aa perm.> .(2430) |
Pseudomonas fluorescens       	| .(1050) <serin prot. .(3721) <kynR kynU> aa perm.> .(1026) |
Pseudomonas mandelii JR-1     	| .(343). serin prot.> .(5679) |
Pseudomonas mediterranea      	| .(247). serin prot.> .(5204) |
Pseudomonas mucidolens        	| .(1482) <kynR kynU> aa perm.> .(1669) serin prot.> .(1955) |
Pseudomonas orientalis        	| .(912). <kynR kynU> aa perm.> .(1839) <serin prot. .(2373) |
Pseudomonas protegens CHA0    	| .(765). <kynR kynU> aa perm.> .(5371) |
Pseudomonas rhodesiae         	| .(710). <kynR kynU> .(1729) <serin prot. .(2682) |
Pseudomonas synxantha         	| .(1005) <kynR kynU> aa perm.> .(2087) <serin prot. .(2722) |
Pseudomonas trivialis         	| .(3567) serin prot.> .(1323) |
Pseudomonas veronii           	| .(951). <kynR kynU> aa perm.> .(5105) |
Pseudomonas balearica DSM 6083	|
Pseudomonas stutzeri          	|
Pseudomonas xanthomarina      	|
Pseudomonas brassicacearum    	| .(2774) serin prot.> .(3108) |
Pseudomonas chlororaphis      	| .(725). <kynR kynU> aa perm.> .(981). kynB> .(1446) <serin prot. .(2822) |
Pseudomonas lundensis         	| .(3159) kynR> .(1185) |
Pseudomonas entomophila L48   	| .(4032) serin prot.> .(1033) |
Pseudomonas extremaustralis   	|
Pseudomonas fulva             	|
Pseudomonas monteilii         	|
Pseudomonas mosselii          	|
Pseudomonas plecoglossicida   	|
Pseudomonas putida NBRC 14164 	|
Pseudomonas glycinae          	| .(1431) <serin prot. .(4239) |
Pseudomonas granadensis       	| .(2349) <serin prot. .(2777) |
Pseudomonas guangdongensis    	| .(249). kynR> .(2631) |
Pseudomonas knackmussii       	|
Pseudomonas koreensis         	| .(5065) serin prot.> .(374). |
Pseudomonas kribbensis        	| .(2733) serin prot.> .(2904) |
Pseudomonas lactis            	| .(922). <kynR kynU> aa perm.> .(1922) <serin prot. .(2517) |
Pseudomonas lalkuanensis      	| .(1318) kynB> .(1689) <serin prot. .(2419) |
Pseudomonas lini              	| .(3891) <kynR .(42).. <serin prot. .(1767) |
Pseudomonas litoralis         	|
Pseudomonas lurida            	| .(917). <kynR kynU> aa perm.> .(4487) |
Pseudomonas marincola         	|
Pseudomonas oryzae            	| .(3215) <serin prot. .(833). <kynR .(66).. |
Pseudomonas otitidis          	| .(2111) <kynR .(1)... kynU> .(1)... aa perm.> .(746). kynB> .(2653) |
Pseudomonas pohangensis       	|
Pseudomonas prosekii          	| .(5121) serin prot.> .(181). |
Pseudomonas psychrophila      	| .(3150) <kynR .(1508) |
Pseudomonas reinekei          	| .(420). kynB> .(5122) |
Pseudomonas rhizosphaerae     	|
Pseudomonas salegens          	| .(1542) <serin prot. .(1867) |
Pseudomonas sabulinigri       	| .(1372) kynR> .(2247) |
Pseudomonas sediminis         	|
Pseudomonas sihuiensis        	|
Pseudomonas silesiensis       	| .(164). <serin prot. .(5772) |
Pseudomonas simiae            	| .(906). <kynR kynU> aa perm.> .(1991) <serin prot. .(2603) |
Pseudomonas soli              	| .(1590) serin prot.> .(4036) |
Pseudomonas thivervalensis    	| .(3485) serin prot.> .(2159) |
Pseudomonas umsongensis       	| .(746). <kynR kynU> .(1)... aa perm.> .(5153) |
Pseudomonas vancouverensis    	| .(1115) <kynB .(3600) serin prot.> .(1045) |
Pseudomonas versuta           	| .(3625) serin prot.> .(841). |
Pseudomonas xinjiangensis     	| .(542). <serin prot. .(2693) |
Pseudomonas yamanorum         	| .(944). <kynR kynU> aa perm.> .(2265) serin prot.> .(2919) |
Acidihalobacter aeolianus     	|
Acidihalobacter ferrooxydans  	|
Alkalilimnicola ehrlichii ML..	| .(2368) <serin prot. .(498). |
Aquisalimonas sp. 2447        	| .(99).. <kynB .(1094) <serin prot. .(2110) kynR> .(330). |
Halorhodospira halochloris    	| .(2498) <serin prot. .(48).. |
Halorhodospira halophila SL1  	| .(2364) serin prot.> .(71).. |
Spiribacter curvatus          	| .(1059) <kynR .(787). |
Spiribacter roseus            	|
Spiribacter salinus M19-40    	|
Thioalkalivibrio nitratiredu..	| .(114). serin prot.> .(3330) |
Thioalkalivibrio paradoxus A..	| .(946). <serin prot. .(2376) |
Thioalkalivibrio sulfidiphil..	| .(221). serin prot.> .(3044) |
Thioalkalivibrio versutus     	| .(1689) kynR> .(1012) |
Allochromatium vinosum DSM 180	| .(1578) kynU> .(1471) |
Marichromatium purpuratum 984 	| .(1591) kynU> .(1640) |
Nitrosococcus halophilus Nc 4 	| .(1137) kynU> .(922). <serin prot. .(1622) |
Nitrosococcus oceani ATCC 19..	| .(793). <serin prot. .(2300) |
Nitrosococcus watsonii C-113  	| .(955). <serin prot. .(1935) |
Nitrosococcus wardiae         	| .(1626) <serin prot. .(1680) <kynU .(363). |
Thermochromatium tepidum ATC..	|
Thioflavicoccus mobilis 8321  	| .(2160) <serin prot. .(1308) |
Thiocystis violascens DSM 198 	|
Granulosicoccus antarcticus ..	| .(1507) kynU> .(1584) serin prot.> .(1035) <kynB .(2510) |
Sulfuriflexus mobilis         	|
Guyparkeria halophila         	|
Halothiobacillus neapolitanu..	| .(153). <kynR .(1476) serin prot.> .(690). |
Sulfurivermis fontis          	|
Wenzhouxiangella marina       	| .(2114) kynU> .(32).. serin prot.> .(910). |
Woeseia oceani                	| .(845). kynU> .(1809) <serin prot. .(889). |
Actinobacillus delphinicola   	|
Actinobacillus equuli subsp...	|
Actinobacillus pleuropneumon..	|
Actinobacillus porcitonsilla..	|
Actinobacillus suis ATCC 33415	|
Aggregatibacter actinomycete..	|
Aggregatibacter aphrophilus ..	|
Aggregatibacter segnis ATCC ..	|
Avibacterium volantium        	|
Basfia succiniciproducens     	|
Bibersteinia trehalosi USDA-..	|
Bisgaardia hudsonensis        	|
Frederiksenia canicola        	|
Glaesserella parasuis SH0165  	|
Haemophilus aegyptius         	|
Haemophilus haemolyticus      	|
Haemophilus influenzae        	|
Haemophilus pittmaniae        	|
Histophilus somni             	|
Mannheimia haemolytica USMAR..	|
Mannheimia ovis               	|
Mannheimia varigena USDA-ARS..	|
Otariodibacter oris           	|
Pasteurella dagmatis          	|
Pasteurella multocida         	|
Pasteurella skyensis          	|
Rodentibacter heylii          	|
Vespertiliibacter pulmonis    	|
Aeromonas allosaccharophila   	|
Aeromonas encheleia           	|
Aeromonas hydrophila          	|
Aeromonas media               	|
Aeromonas salmonicida         	|
Aeromonas simiae              	|
Aeromonas veronii             	|
Oceanisphaera avium           	|
Oceanisphaera profunda        	|
Tolumonas auensis DSM 9187    	|
Zobellella denitrificans      	| .(763). serin prot.> .(2097) <kynU .(948). |
Aerosticca soli               	| .(1154) <kynU .(1443) |
Ahniella affigens             	| .(2380) <kynU .(1959) <serin prot. .(117). |
Dokdonella koreensis DS-123   	| .(612). <kynU .(857). serin prot.> .(2094) |
Dyella thiooxydans            	| .(1492) <serin prot. .(79).. kynU> .(2197) |
Frateuria aurantia DSM 6220   	| .(1925) kynU> .(1179) |
Luteibacter pinisoli          	| .(1289) <kynU .(247). serin prot.> .(2130) <serin prot. .(430). |
Luteibacter rhizovicinus DSM..	| .(115). kynU> .(2373) serin prot.> .(1781) |
Rhodanobacter denitrificans   	| .(2300) serin prot.> .(352). kynU> .(551). serin prot.> .(630). |
Arenimonas daejeonensis       	| .(762). kynU> .(255). <serin prot. .(610). <serin prot. .(624). |
Luteimonas chenhongjianii     	| .(1100) <serin prot. .(459). <kynU .(1351) |
Luteimonas granuli            	| .(2031) serin prot.> .(438). <kynU .(20).. |
Lysobacter alkalisoli         	| .(976). serin prot.> .(872). kynU> .(1254) serin prot.> .(199). |
Lysobacter antibioticus       	| .(2350) <kynU .(2300) <serin prot. .(149). |
Lysobacter capsici            	| .(2515) kynU> .(1084) <serin prot. .(1497) |
Lysobacter enzymogenes        	| .(1286) serin prot.> .(1043) <kynU .(2504) |
Lysobacter gummosus           	| .(1479) serin prot.> .(1048) <kynU .(186). serin prot.> .(2234) |
Lysobacter lycopersici        	| .(487). <kynU .(2010) |
Lysobacter maris              	| .(1596) <kynU .(705). kynB> .(957). serin prot.> .(140). |
Lysobacter oculi              	| .(770). <kynU .(1627) |
Lysobacter soli               	| .(939). serin prot.> .(1325) kynU> .(1392) |
Pseudolysobacter antarcticus  	| .(2439) kynU> .(207). <serin prot. .(1299) |
Pseudoxanthomonas mexicana    	| .(1616) serin prot.> .(737). <kynU .(1291) |
Pseudoxanthomonas spadix BD-..	| .(1460) <kynU .(885). <serin prot. .(740). |
Stenotrophomonas acidaminiph..	| .(1237) serin prot.> .(923). kynU> .(1446) |
Stenotrophomonas maltophilia  	| .(1528) <kynU .(337). kynR> .(1634) <serin prot. .(737). |
Stenotrophomonas rhizophila   	| .(1492) <kynU .(1656) <serin prot. .(840). |
Thermomonas brevis            	| .(27).. <kynU .(3055) |
Xanthomonas albilineans       	| .(54).. kynR> .(1537) kynU> .(856). <serin prot. .(499). |
Xanthomonas cassavae CFBP 4642	| .(1020) <kynU .(1895) <serin prot. <serin prot. .(889). <kynR .(364). |
Xanthomonas campestris pv. r..	| .(934). serin prot.> serin prot.> serin prot.> .(667). <kynU .(2261) <kynR .(174). |
Xanthomonas citri             	| .(2561) kynU> .(652). <serin prot. <serin prot. <serin prot. .(751). <kynR .(145). |
Xanthomonas cucurbitae        	| .(116). kynR> .(2298) kynU> .(587). <serin prot. <serin prot. .(685). |
Xanthomonas euroxanthea       	| .(2406) kynU> .(693). <serin prot. <serin prot. <serin prot. .(712). <kynR .(171). |
Xanthomonas fragariae         	| .(109). kynR> .(1306) <kynU .(980). <serin prot. .(821). |
Xanthomonas hortorum          	| .(1024) <kynR .(1092) serin prot.> serin prot.> serin prot.> .(1773) kynU> .(258). |
Xanthomonas hyacinthi         	| .(2701) serin prot.> .(630). <kynU .(675). |
Xanthomonas oryzae pv. oryzi..	| .(117). kynR> .(1091) <kynU .(1666) serin prot.> .(595). |
Xanthomonas phaseoli pv. die..	| .(845). <kynU .(1547) <serin prot. <serin prot. .(714). <kynR .(870). |
Xanthomonas vesicatoria ATCC..	| .(183). kynU> .(703). <serin prot. <serin prot. <serin prot. .(756). <kynR .(2459) |
Xylella fastidiosa Temecula1  	|
Xylella taiwanensis           	| .(1552) serin prot.> .(623). |
Agarilytica rhodophyticola    	| .(191). <kynU .(4458) serin prot.> .(860). |
Cellvibrio japonicus Ueda107  	| .(443). <serin prot. .(3172) |
Saccharophagus degradans 2-40 	| .(297). serin prot.> .(3757) |
Simiduia agarivorans SA1 = D..	| .(1188) <serin prot. .(2554) |
Congregibacter litoralis KT71 	| .(8)... serin prot.> .(2335) <kynU .(1461) |
Halioglobus maricola          	| .(1424) serin prot.> .(2441) |
Kineobactrum salinum          	| .(1205) kynB> .(391). serin prot.> .(293). <kynR .(1403) kynU> .(617). |
Microbulbifer aggregans       	| .(798). serin prot.> .(897). <serin prot. .(1591) |
Microbulbifer agarilyticus    	| .(439). <serin prot. .(3017) |
Microbulbifer hydrolyticus    	| .(95).. <serin prot. .(395). <serin prot. .(2952) |
Microbulbifer thermotolerans  	| .(2284) <serin prot. .(935). |
Oceanicoccus sagamiensis      	|
Zhongshania aliphaticivorans  	| .(3147) <serin prot. .(532). |
Alcanivorax borkumensis SK2   	|
Alcanivorax dieselolei B5     	|
Alcanivorax pacificus W11-5   	|
Ketobacter alkanivorans       	| .(2036) <serin prot. .(1505) serin prot.> .(598). |
Bermanella marisrubri         	| .(1762) <kynR .(63).. serin prot.> .(1353) |
Marinobacterium aestuarii     	| .(978). kynU> .(837). serin prot.> .(2644) |
Marinomonas arctica           	| .(1837) kynR> .(2221) |
Marinomonas mediterranea MMB-1	| .(702). <kynU .(1408) <kynR .(1885) <serin prot. .(106). |
Marinomonas posidonica IVIA-..	| .(1547) kynR> .(1929) |
Marinomonas primoryensis      	| .(2057) <kynR .(1570) |
Neptunomonas concharum        	|
Neptunomonas phycophila       	|
Thalassolituus oleivorans MI..	| .(2485) <kynR .(57).. serin prot.> .(970). |
Chromohalobacter salexigens ..	|
Cobetia marina                	|
Halomonas aestuarii           	| .(687). <kynB .(2529) |
Halomonas beimenensis         	| .(3077) <kynB <kynU .(15).. <kynB .(556). |
Halomonas campisalis          	| .(2965) <kynB .(336). <kynU .(741). |
Halomonas chromatireducens    	| .(715). kynB> .(937). kynU> .(1843) |
Halomonas elongata DSM 2581   	| .(519). kynU> .(3186) |
Halomonas huangheensis        	| .(2837) <kynB <kynU .(1196) |
Halomonas hydrothermalis      	|
Halomonas piezotolerans       	| .(1915) <serin prot. .(1609) |
Halomonas socia               	|
Halomonas subglaciescola      	|
Halomonas titanicae           	| .(683). kynB> .(458). serin prot.> .(4)... <kynU .(3558) |
Kushneria konosiri            	|
Kushneria marisflavi          	|
Pistricoccus aurantiacus      	|
Salinicola tamaricis          	| .(184). kynB> .(2841) |
Zymobacter palmae             	|
Endozoicomonas montiporae CL..	| .(536). <serin prot. .(3991) kynU> .(198). |
Gynuella sunshinyii YC6258    	| .(4612) <serin prot. .(563). |
Reinekea forsetii             	| .(587). serin prot.> .(689). <kynU .(1944) |
Saccharospirillum mangrovi    	|
Hahella chejuensis KCTC 2396  	| .(5039) <serin prot. .(1222) |
Spartinivicinus ruber         	| .(352). kynB> .(179). <serin prot. .(2250) <kynU .(2785) |
Kangiella geojedonensis       	| .(331). <serin prot. .(1634) kynU> .(288). |
Kangiella koreensis DSM 16069 	| .(385). <serin prot. .(481). <serin prot. .(1366) kynU> .(365). |
Kangiella profundi            	| .(319). <kynU .(1726) serin prot.> .(375). |
Kangiella sediminilitoris     	| .(344). <serin prot. .(1664) kynU> .(296). |
Litoricola lipolytica         	| .(1157) <serin prot. .(1170) |
Oleiphilus messinensis        	| .(1577) <serin prot. .(2337) <serin prot. .(1495) |
Aliivibrio salmonicida LFI1238	|
Grimontia hollisae            	|
Paraphotobacterium marinum    	|
Photobacterium gaetbulicola ..	|
Salinivibrio kushneri         	| .(248). <serin prot. .(274). |
Vibrio alfacsensis            	| .(277). kynU> .(221). <serin prot. .(705). |
Vibrio antiquarius            	| .(1446) <serin prot. .(117). |
Vibrio diabolicus             	| .(1073) serin prot.> .(528). |
Vibrio azureus                	| .(1248) serin prot.> .(1314) | .(708). <serin prot. .(606). |
Vibrio campbellii             	| .(970). <kynB .(605). <serin prot. .(1272) |
Vibrio harveyi                	| .(1292) kynU> .(657). | .(1818) <serin prot. .(1426) |
Vibrio jasicida 090810c       	| .(1769) <kynU .(202). | .(271). serin prot.> .(2941) |
Vibrio natriegens NBRC 15636..	| .(1219) serin prot.> .(431). |
Vibrio owensii                	| .(1801) kynR> .(221). | .(3005) serin prot.> .(83).. |
Vibrio parahaemolyticus       	| .(1115) <kynU .(435). serin prot.> .(73).. |
Vibrio rotiferianus           	| .(486). <kynU .(94).. <serin prot. .(1126) |
Vibrio anguillarum            	|
Vibrio aphrogenes             	|
Vibrio aquimaris              	| .(830). kynB> .(1896) | .(768). <serin prot. .(327). |
Vibrio astriarenae            	| .(1500) <kynR .(1238) | .(326). serin prot.> .(1117) |
Vibrio atlanticus             	| .(751). serin prot.> .(796). kynR> .(1249) |
Vibrio cholerae MS6           	| .(2413) serin prot.> .(129). |
Vibrio cyclitrophicus         	|
Vibrio europaeus              	| .(607). serin prot.> .(1067) |
Vibrio tubiashii ATCC 19109   	| .(186). <serin prot. .(1408) |
Vibrio fluvialis              	| .(1521) <serin prot. .(1271) |
Vibrio furnissii              	| .(1339) serin prot.> .(1562) |
Vibrio kanaloae               	| .(2354) <kynR .(102). <serin prot. .(202). |
Vibrio metoecus               	|
Vibrio metschnikovii          	| .(627). serin prot.> .(1843) |
Vibrio mediterranei           	| .(1796) <kynR .(66).. | .(2055) serin prot.> .(1160) |
Vibrio navarrensis            	| .(1009) serin prot.> .(14).. |
Vibrio ponticus               	| .(855). <kynB .(1288) <serin prot. .(632). |
Vibrio qinghaiensis           	| .(150). <serin prot. .(2292) |
Vibrio rumoiensis             	|
Vibrio scophthalmi            	|
Vibrio spartinae              	| .(842). <kynR .(149). |
Vibrio tapetis subsp. tapetis 	| .(250). <serin prot. .(1292) |
Vibrio taketomensis           	|
Vibrio tritonius              	| .(498). <kynR .(1090) |
Vibrio vulnificus             	| .(1192) serin prot.> .(208). |
Allofrancisella frigidaquae   	|
Allofrancisella guangzhouensis	| .(708). <serin prot. .(791). |
Francisella adeliensis        	| .(1118) <serin prot. .(770). |
Francisella frigiditurris     	|
Francisella halioticida       	|
Francisella hispaniensis      	|
Francisella marina            	| .(123). serin prot.> .(1821) |
Francisella noatunensis subs..	|
Francisella opportunistica    	|
Francisella orientalis LADL-..	|
Francisella orientalis FNO12  	|
Francisella orientalis FNO24  	|
Francisella persica ATCC VR-..	|
Francisella philomiragia      	| .(1043) <serin prot. .(749). |
Francisella salina            	| .(676). serin prot.> .(1220) |
Francisella uliginis          	|
Beggiatoa leptomitoformis     	| .(384). serin prot.> .(3118) |
Hydrogenovibrio crunogenus X..	|
Hydrogenovibrio marinus       	|
Hydrogenovibrio thermophilus  	|
Methylophaga frappieri        	|
Methylophaga nitratireducent..	|
Piscirickettsia salmonis      	|
Thiomicrospira aerophila AL3  	|
Thiomicrospira cyclica ALM1   	|
Thiomicrorhabdus aquaedulcis  	| .(24).. kynR> .(2036) |
Thiomicrorhabdus indica       	|
Thiosulfatimonas sediminis    	| .(2318) serin prot.> .(158). |
Thiosulfativibrio zosterae    	|
Alteromonas addita            	| .(3365) <serin prot. .(484). |
Alteromonas australica        	| .(3410) serin prot.> .(251). |
Alteromonas mediterranea      	| .(1424) kynU> .(1816) <serin prot. .(494). |
Alteromonas naphthalenivorans 	| .(3591) <serin prot. .(587). |
Alteromonas pelagimontana     	| .(2382) serin prot.> .(1272) |
Alteromonas stellipolaris LM..	| .(3818) serin prot.> .(49).. |
Catenovulum sediminis         	| .(1491) serin prot.> .(1876) |
Glaciecola amylolytica        	| .(3230) serin prot.> .(57).. <kynU .(513). |
Glaciecola nitratireducens F..	| .(2380) <kynU .(902). serin prot.> .(134). |
Hydrocarboniclastica marina   	| .(2355) <serin prot. .(1206) |
Marinobacter adhaerens HP15   	| .(61).. serin prot.> .(76).. |
Marinobacter fonticola        	| .(2656) <serin prot. .(1278) |
Marinobacter hydrocarbonocla..	| .(2483) <serin prot. .(1098) |
Marinobacter psychrophilus    	|
Marinobacter salarius         	| .(1248) serin prot.> .(504). <kynR .(2194) |
Marinobacter salinus          	| .(358). serin prot.> .(452). <kynR .(2889) |
Saliniradius amylolyticus     	| .(487). serin prot.> .(2157) kynU> .(277). |
Salinimonas lutimaris         	| .(3060) <serin prot. .(498). |
Salinimonas sediminis         	| .(1535) <kynU .(1660) <serin prot. .(494). |
Colwellia beringensis         	| .(949). <serin prot. .(349). kynR> .(1829) kynU> .(677). |
Colwellia psychrerythraea 34H 	| .(663). serin prot.> .(3736) |
Litorilituus sediminis        	| .(44).. <serin prot. .(3620) |
Thalassotalea crassostreae    	| .(510). kynB> .(193). kynU> .(2288) <serin prot. <serin prot. .(256). |
Ferrimonas balearica DSM 9799 	| .(2671) <serin prot. .(782). <kynU .(309). |
Idiomarina andamanensis       	| .(875). <serin prot. .(1325) |
Idiomarina loihiensis L2TR    	| .(159). <serin prot. .(2020) kynU> .(461). |
Moritella marina ATCC 15381   	| .(2485) kynU> .(1532) |
Moritella yayanosii           	|
Parashewanella spongiae       	| .(30).. serin prot.> .(4122) |
Parashewanella tropica        	| .(804). <serin prot. .(2801) |
Shewanella algae              	| .(2085) serin prot.> .(931). <kynU .(1264) |
Shewanella amazonensis SB2B   	| .(98).. <serin prot. .(141). kynU> .(2979) <serin prot. .(441). |
Shewanella baltica OS678      	| .(301). kynU> .(2908) serin prot.> .(1160) |
Shewanella bicestrii          	| .(2293) <serin prot. .(1373) <kynU .(316). |
Shewanella decolorationis     	| .(1972) serin prot.> .(279). kynU> .(1771) |
Shewanella denitrificans OS217	| .(321). <kynU .(589). serin prot.> .(1839) serin prot.> .(1013) |
Shewanella donghaensis        	| .(1782) serin prot.> .(2224) |
Shewanella frigidimarina NCI..	| .(2654) serin prot.> .(1346) |
Shewanella halifaxensis HAW-..	| .(973). serin prot.> .(3319) |
Shewanella japonica           	| .(255). <kynU .(458). serin prot.> .(3336) |
Shewanella khirikhana         	| .(1377) serin prot.> .(1817) <kynU .(838). |
Shewanella livingstonensis    	| .(2889) serin prot.> .(1116) |
Shewanella loihica PV-4       	| .(314). kynU> .(1127) <serin prot. .(231). <serin prot. .(2216) |
Shewanella maritima           	|
Shewanella marisflavi         	| .(3362) <kynU .(255). serin prot.> .(31).. |
Shewanella oneidensis MR-1    	| .(177). <kynU .(1070) serin prot.> .(2880) |
Shewanella pealeana ATCC 700..	| .(909). serin prot.> .(2978) <kynU .(345). |
Shewanella piezotolerans WP3  	| .(801). serin prot.> .(895). <serin prot. .(2458) <kynU .(233). |
Shewanella polaris            	| .(1210) <serin prot. .(2652) |
Shewanella psychrophila       	| .(815). serin prot.> .(2474) kynR> .(1984) |
Shewanella putrefaciens CN-32 	| .(388). kynU> .(2043) serin prot.> .(1506) |
Shewanella sediminis HAW-EB3  	| .(1623) <serin prot. .(2905) |
Shewanella violacea DSS12     	| .(1890) serin prot.> .(1997) |
Shewanella woodyi ATCC 51908  	| .(2513) <serin prot. .(961). kynU> .(2)... kynB> .(1391) |
Pseudoalteromonas agarivorans 	| .(2634) serin prot.> .(431). |
Pseudoalteromonas aliena SW19 	| .(19).. kynU> .(72).. | .(139). <serin prot. .(232). |
Pseudoalteromonas arctica A ..	| .(53).. kynU> .(2843) serin prot.> .(393). |
Pseudoalteromonas carrageeno..	| .(51).. kynU> .(2662) serin prot.> .(408). |
Pseudoalteromonas donghaensis 	| .(91).. kynU> .(3027) | .(34).. <serin prot. .(664). |
Pseudoalteromonas espejiana ..	| .(2785) serin prot.> .(415). |
Pseudoalteromonas issachenko..	| .(577). kynU> .(1978) serin prot.> .(414). |
Pseudoalteromonas luteoviola..	| .(2132) <serin prot. .(1191) kynU> .(376). |
Pseudoalteromonas paragorgic..	| .(21).. kynU> .(339). <serin prot. .(316). |
Pseudoalteromonas phenolica   	| .(1065) kynU> .(2243) | .(220). serin prot.> .(623). |
Pseudoalteromonas piratica    	| .(336). serin prot.> .(1085) |
Pseudoalteromonas prydzensis..	| .(294). <kynU .(19).. | .(219). <serin prot. .(716). |
Pseudoalteromonas rubra       	| .(11).. kynU> .(9)... | .(713). <serin prot. .(406). |
Pseudoalteromonas spongiae U..	| .(293). serin prot.> .(1106) |
Pseudoalteromonas tetraodonis 	| .(533). kynU> .(2041) serin prot.> .(399). |
Pseudoalteromonas translucida 	| .(148). kynB> .(384). | .(50).. kynU> .(2416) serin prot.> .(349). |
Pseudoalteromonas tunicata    	| .(18).. serin prot.> .(700). |
Pseudoalteromonas undina      	| .(415). kynU> .(153). | .(142). <serin prot. .(2048) |
Psychromonas ingrahamii 37    	|
Aquicella lusitana            	|
Aquicella siphonis            	| .(765). <serin prot. .(1404) |
Coxiella burnetii RSA 493     	|
Fluoribacter dumoffii Tex-KL  	| .(2129) kynR> .(117). <serin prot. .(877). |
Legionella adelaidensis       	|
Legionella anisa              	| .(655). serin prot.> .(97).. <kynR .(451). <kynU .(2315) |
Legionella clemsonensis       	| .(2210) <serin prot. .(583). |
Legionella fallonii LLAP-10   	| .(1442) serin prot.> .(637). <kynR .(1092) <kynU .(217). |
Legionella hackeliae          	| .(595). serin prot.> .(2344) |
Legionella israelensis        	| .(1899) <serin prot. .(683). |
Legionella lansingensis       	| .(536). serin prot.> .(1209) <kynU .(898). |
Legionella pneumophila        	| .(1974) <serin prot. .(495). kynR> .(488). |
Legionella sainthelensi       	| .(2011) kynR> .(86).. <serin prot. .(1447) |
Legionella spiritensis        	| .(2653) serin prot.> .(302). |
Legionella waltersii          	| .(2151) <serin prot. .(470). <kynR .(676). |
Tatlockia micdadei            	|
Atlantibacter hermannii       	|
Buttiauxella agrestis         	| .(1292) kynR> .(2800) |
Cedecea lapagei               	|
Cedecea neteri                	|
Citrobacter amalonaticus      	|
Citrobacter freundii          	|
Citrobacter portucalensis     	|
Citrobacter werkmanii         	|
Citrobacter rodentium ICC168  	|
Cronobacter condimenti 1330   	| .(558). serin prot.> .(801). <kynR .(2552) |
Cronobacter dublinensis subs..	| .(564). serin prot.> .(845). <kynR .(2630) |
Cronobacter malonaticus LMG ..	| .(2421) kynR> .(842). <serin prot. .(658). |
Cronobacter muytjensii ATCC ..	| .(2293) kynR> .(808). <serin prot. .(822). |
Cronobacter sakazakii         	| .(2553) <serin prot. .(1318) |
Cronobacter universalis NCTC..	| .(557). serin prot.> .(3363) |
Enterobacter asburiae         	|
Enterobacter cancerogenus     	|
Enterobacter chengduensis     	|
Enterobacter cloacae          	|
Enterobacter ludwigii         	|
Enterobacter roggenkampii     	|
Enterobacter sichuanensis     	|
Enterobacter oligotrophicus   	|
Enterobacter soli             	|
Escherichia albertii          	|
Escherichia coli O26 str. RM..	|
Escherichia coli O26 str. RM..	|
Escherichia coli O103 str. R..	|
Escherichia coli O43 str. RM..	|
Escherichia coli O111 str. R..	|
Escherichia coli O121 str. R..	|
Escherichia coli O145 str. R..	|
Escherichia coli O157:H7 str..	|
Escherichia coli str. K-12 s..	|
Escherichia fergusonii        	|
Escherichia marmotae          	|
Klebsiella aerogenes          	|
Klebsiella huaxiensis         	|
Klebsiella michiganensis      	|
Klebsiella pneumoniae subsp...	|
Klebsiella quasipneumoniae    	|
Klebsiella variicola          	|
Kluyvera intermedia           	|
Kosakonia arachidis           	|
Kosakonia cowanii             	|
Kosakonia oryzae              	|
Kosakonia pseudosacchari      	|
Kosakonia radicincitans       	|
Kosakonia sacchari            	|
Leclercia adecarboxylata      	|
Lelliottia amnigena           	|
Lelliottia jeotgali           	|
Lelliottia nimipressuralis    	|
Phytobacter diazotrophicus    	|
Phytobacter ursingii          	|
Pluralibacter gergoviae       	|
Raoultella electrica          	|
Raoultella ornithinolytica    	| .(1236) kynB> .(2)... aa perm.> .(3801) |
Raoultella planticola         	| .(2751) kynB> .(2)... aa perm.> .(2192) |
Raoultella terrigena          	|
Salmonella bongori            	|
Salmonella enterica subsp. e..	|
Salmonella enterica subsp. e..	|
Salmonella enterica subsp. e..	|
Salmonella enterica subsp. e..	|
Scandinavium goeteborgense    	|
Shimwellia blattae DSM 4481 ..	|
Shigella dysenteriae          	|
Shigella flexneri 2a str. 301 	|
Brenneria goodwinii           	|
Brenneria nigrifluens DSM 30..	|
Brenneria rubrifaciens        	|
Dickeya aquatica              	| .(3521) <serin prot. .(339). |
Dickeya chrysanthemi Ech1591  	| .(272). serin prot.> .(3841) |
Dickeya dadantii 3937         	| .(3795) <serin prot. .(422). |
Dickeya dianthicola           	| .(3828) <serin prot. .(371). |
Dickeya fangzhongdai          	| .(2779) serin prot.> .(1500) |
Dickeya paradisiaca Ech703    	|
Dickeya poaceiphila           	|
Dickeya solani IPO 2222       	| .(1375) <serin prot. .(2742) |
Dickeya zeae                  	| .(3653) <serin prot. .(414). |
Lonsdalea britannica          	|
Lonsdalea populi              	|
Pectobacterium atrosepticum   	|
Pectobacterium brasiliense    	|
Pectobacterium carotovorum    	|
Pectobacterium odoriferum     	|
Pectobacterium parmentieri    	|
Pectobacterium polaris        	|
Pectobacterium punjabense     	| .(816). kynR> .(3352) |
Pectobacterium versatile      	|
Pectobacterium wasabiae CFBP..	|
Buchnera aphidicola (Diuraph..	|
Buchnera aphidicola str. Bp ..	|
Erwinia amylovora CFBP1430    	| .(1945) <kynR .(1329) |
Erwinia billingiae Eb661      	|
Erwinia gerundensis           	|
Erwinia pyrifoliae            	| .(2061) <kynR .(1409) |
Erwinia tasmaniensis Et1/99   	|
Mixta calida                  	| .(550). serin prot.> .(1780) <kynR .(1466) |
Mixta gaviniae                	| .(2393) <kynR .(1583) |
Mixta intestinalis            	| .(4026) serin prot.> .(125). |
Pantoea agglomerans           	| .(75).. kynR> .(480). |
Pantoea alhagi                	| .(2973) kynR> .(850). |
Pantoea ananatis PA13         	| .(1575) kynR> .(2579) |
Pantoea eucalypti             	| .(287). <kynR .(203). |
Pantoea stewartii             	|
Pantoea vagans                	| .(374). kynR> .(136). |
Tatumella citrea              	|
Wigglesworthia glossinidia e..	|
Chania multitudinisentens RB..	| .(807). kynR> .(314). kynB> .(2)... aa perm.> .(3628) |
Gibbsiella quercinecans       	| .(1907) kynR> .(2909) |
Rahnella aquatilis CIP 78.65..	|
Rouxiella badensis            	|
Serratia ficaria              	| .(193). serin prot.> .(1475) kynB> .(1)... aa perm.> .(3023) |
Serratia fonticola            	| .(467). kynB> .(2)... aa perm.> .(4778) |
Serratia marcescens           	| .(498). kynB> .(2)... aa perm.> .(3057) serin prot.> .(1062) |
Serratia nematodiphila        	| .(190). serin prot.> .(1629) kynB> .(2)... aa perm.> .(3007) |
Serratia plymuthica AS9       	| .(194). serin prot.> .(1639) kynB> .(2)... aa perm.> .(3099) |
Serratia quinivorans          	| .(205). serin prot.> .(1539) kynB> .(2)... aa perm.> .(3111) |
Serratia rubidaea             	| .(316). kynB> .(1)... aa perm.> .(3074) serin prot.> .(1156) |
Serratia surfactantfaciens    	| .(2323) kynB> .(2)... aa perm.> .(2409) |
Yersinia aldovae 670-83       	| .(1587) kynR> .(2250) |
Yersinia canariae             	| .(2864) serin prot.> .(408). kynR> .(833). |
Yersinia enterocolitica       	| .(788). <kynR .(1753) serin prot.> .(1592) |
Yersinia entomophaga          	| .(3380) <serin prot. .(334). |
Yersinia hibernica            	|
Yersinia intermedia           	| .(727). <kynR .(3595) |
Yersinia mollaretii ATCC 43969	|
Yersinia pestis A1122         	|
Yersinia pseudotuberculosis   	|
Yersinia similis              	|
Yersinia rohdei               	| .(198). <serin prot. .(1470) kynR> .(2041) |
Yersinia ruckeri              	| .(727). serin prot.> .(2435) |
Edwardsiella anguillarum ET0..	|
Edwardsiella hoshinae         	|
Edwardsiella ictaluri 93-146  	| .(1240) kynB> .(1988) |
Edwardsiella tarda            	|
Hafnia alvei                  	|
Leminorella richardii         	|
Limnobaculum parvum           	|
Pragia fontium                	|
Photorhabdus asymbiotica      	|
Photorhabdus laumondii subsp..	| .(1352) kynU> .(3277) |
Photorhabdus thracensis       	|
Providencia alcalifaciens     	|
Providencia heimbachae        	|
Providencia rettgeri          	| .(1870) <kynR .(1941) |
Providencia sneebia DSM 19967 	|
Providencia stuartii MRSN 2154	| .(2994) <kynR .(832). |
Providencia vermicola         	| .(2761) kynR> .(981). |
Proteus terrae subsp. cibarius	|
Proteus hauseri               	|
Proteus mirabilis HI4320      	|
Xenorhabdus bovienii SS-2004  	|
Xenorhabdus doucetiae         	|
Xenorhabdus hominickii        	| .(2380) <serin prot. .(1488) |
Xenorhabdus nematophila       	|
Xenorhabdus poinarii G6       	|
Plesiomonas shigelloides      	|
Sodalis praecaptivus          	|
Cardiobacterium hominis       	|
Dichelobacter nodosus VCS1703A	| .(434). <kynR .(133). serin prot.> .(706). |
Frischella perrara            	|
Gallaecimonas mangrovi        	| .(2871) serin prot.> .(868). |
Pseudohongiella spirulinae    	| .(2688) <kynU .(336). |
Sedimenticola thiotaurini     	| .(1295) kynB> .(1520) kynU> .(592). serin prot.> .(170). |
Thiolapillus brandeum         	|
Immundisolibacter cernigliae  	|
Methylotuvimicrobium alcalip..	| .(2618) serin prot.> .(1188) |
Methylotuvimicrobium buryate..	| .(387). serin prot.> .(3722) |
Methylomicrobium album BG8    	|
Methylococcus capsulatus str..	| .(832). serin prot.> .(2131) |
Methylomonas denitrificans    	| .(1874) aa perm.> .(1175) serin prot.> .(1488) |
Methylomonas rhizoryzae       	| .(373). serin prot.> .(976). serin prot.> .(2566) |
Methylocaldum marinum         	| .(726). <serin prot. .(2547) <kynU .(2039) |
Steroidobacter denitrificans  	|
Sulfuricaulis limicola        	|
Sulfurifustis variabilis      	| .(1260) serin prot.> .(2583) |
Acetobacter ascendens         	| .(2331) <kynR .(91).. |
Acetobacter oryzifermentans   	|
Acetobacter oryzoeni          	|
Acetobacter pasteurianus 386B 	| .(25).. kynR> .(152). |
Acetobacter senegalensis      	|
Acidibrevibacterium fodinaqu..	|
Acidiphilium multivorum AIU301	| .(1444) <kynB .(1913) |
Asaia bogorensis NBRC 16594   	|
Gluconobacter albidus         	|
Gluconobacter oxydans DSM 3504	|
Gluconobacter thailandicus    	|
Granulibacter bethesdensis C..	| .(476). aa perm.> .(458). <serin prot. .(1454) |
Komagataeibacter hansenii     	|
Komagataeibacter medellinens..	|
Komagataeibacter nataicola    	|
Komagataeibacter rhaeticus    	| .(2679) kynB> .(431). |
Komagataeibacter saccharivor..	| .(1492) kynB> .(1220) |
Komagataeibacter xylinus      	|
Kozakia baliensis             	|
Neokomagataea tanensis        	|
Oecophyllibacter saccharovor..	|
Parasaccharibacter apium      	|
Stella humosa                 	| .(202). <kynB .(624). kynB> .(3442) <serin prot. .(1151) |
Swingsia samuiensis           	|
Azospirillum humicireducens   	| .(379). <kynB .(1)... <kynU .(230). |
Azospirillum oryzae           	| .(815). kynU> .(1)... kynB> .(92).. |
Azospirillum ramasamyi        	| .(264). <kynB .(1)... <kynU .(273). |
Azospirillum thermophilum     	| .(1702) kynB> .(548). | .(304). serin prot.> .(178). |
Azospirillum thiophilum       	|
Defluviicoccus vanus          	| .(1258) serin prot.> .(2159) |
Ferrovibrio terrae            	| .(4016) <kynB .(76).. kynB> .(35).. |
Haematospirillum jordaniae    	|
Hypericibacter adhaerens      	| .(2551) kynB> .(340). <kynB .(2278) |
Hypericibacter terrae         	| .(73).. <serin prot. .(3174) kynB> .(331). <kynB .(1729) |
Indioceanicola profundi       	| .(2644) <kynR .(265). <kynU .(316). | .(133). <kynB .(255). |
Magnetospirillum gryphiswald..	|
Magnetospirillum magneticum ..	|
Nitrospirillum amazonense CB..	| .(574). <kynU .(142). kynR> .(513). |
Niveispirillum cyanobacterio..	| .(1008) kynR> .(2122) | .(99).. <kynU .(243). <kynU .(393). <serin prot. .(39).. |
Pararhodospirillum photometr..	| .(2804) <serin prot. .(455). |
Rhodospirillum rubrum F11     	| .(3049) <kynU .(767). |
Skermanella pratensis         	| .(222). <kynU .(2205) kynB> .(2318) serin prot.> .(428). |
Thalassospira indica          	| .(1470) kynU> .(1159) <kynB .(1599) |
Thalassospira marina          	| .(153). kynU> .(565). | .(2032) <kynB .(1880) |
Tistrella mobilis KA081020-065	| .(43).. <kynR .(524). | .(439). kynB> .(154). |
Agrobacterium tumefaciens     	| .(1780) <kynR .(1071) |
Neorhizobium galegae bv. ori..	| .(1856) <kynR .(560). <kynR .(1451) kynU> .(235). <kynB .(319). |
Rhizobium acidisoli           	| .(1378) <kynR .(2866) |
Rhizobium esperanzae          	| .(1820) <kynR .(2409) |
Rhizobium etli                	| .(1734) <kynR .(2283) | .(477). <kynB .(395). |
Rhizobium favelukesii         	| .(1709) <kynR .(2229) |
Rhizobium flavum              	| .(4)... <kynB .(3843) |
Rhizobium hidalgonense        	| .(2342) kynR> .(1744) | .(592). <kynB .(830). |
Rhizobium indicum             	| .(570). <kynB .(431). | .(651). kynR> .(28).. serin prot.> .(4074) |
Rhizobium jaguaris            	| .(1722) <kynR .(2474) | .(1605) kynU> kynB> .(664). | .(126). <serin prot. .(92).. |
Rhizobium oryzihabitans       	| .(2777) kynR> .(202). |
Rhizobium phaseoli            	| .(1771) <kynR .(2460) | .(5)... <serin prot. .(324). |
Rhizobium pseudoryzae         	| .(1069) <kynB .(1)... <kynU .(2401) | .(540). serin prot.> .(551). |
Rhizobium pusense             	| .(126). kynB> .(317). |
Rhizobium rhizoryzae          	| .(542). <kynR .(1760) <kynB .(1)... <kynU .(792). |
Rhizobium tropici CIAT 899    	| .(1509) <kynR .(2129) | .(1232) kynU> kynB> .(595). |
Ciceribacter thiooxidans      	|
Ensifer adhaerens             	| .(328). kynU> .(8)... kynB> .(44).. kynU> .(865). | .(272). serin prot.> .(1246) |
Ensifer alkalisoli            	| .(1312) <kynR .(2033) |
Ensifer mexicanus             	| .(1396) <kynB .(300). |
Ensifer sojae CCBAU 05684     	| .(1345) <kynR .(332). <serin prot. .(1667) |
Sinorhizobium americanum      	| .(549). <serin prot. .(1092) <kynR .(1799) |
Sinorhizobium fredii CCBAU 2..	| .(1487) <kynR .(2299) | .(797). <serin prot. .(1107) |
Sinorhizobium meliloti 2011   	| .(1614) kynR> .(1707) | .(77).. <serin prot. .(65).. <kynB .(1040) |
Georhizobium profundi         	| .(301). kynU> .(932). <kynB .(2433) <serin prot. .(465). |
Liberibacter crescens         	|
Ancylobacter pratisalsi       	| .(12).. <kynB .(2351) <kynR .(1805) |
Azorhizobium caulinodans ORS..	| .(942). kynB> .(2294) serin prot.> .(1292) <kynR .(241). |
Pseudolabrys taiwanensis      	| .(874). serin prot.> .(953). <kynB .(110). kynB> .(1994) serin prot.> .(1306) |
Starkeya novella DSM 506      	| .(1950) <serin prot. .(347). kynB> .(2136) |
Bartonella alsatica           	|
Bartonella ancashensis        	|
Bartonella australis Aust/NH1 	|
Bartonella bacilliformis KC583	|
Bartonella bovis 91-4         	|
Bartonella clarridgeiae 73    	|
Bartonella elizabethae        	|
Bartonella grahamii as4aup    	|
Bartonella henselae           	|
Bartonella kosoyi             	|
Bartonella krasnovii          	|
Bartonella quintana           	|
Bartonella tribocorum CIP 10..	|
Beijerinckia indica subsp. i..	| .(2766) <serin prot. .(806). |
Methylovirgula ligni          	|
Methylocella silvestris BL2   	| .(1603) kynR> .(2220) |
Blastochloris tepida          	| .(2787) serin prot.> .(301). <kynB .(428). |
Blastochloris viridis         	| .(1379) serin prot.> .(1779) <kynB .(66).. |
Devosia ginsengisoli          	|
Hyphomicrobium denitrificans..	|
Hyphomicrobium nitrativorans..	| .(1709) serin prot.> .(1593) |
Maritalea myrionectae         	| .(453). <kynU .(8)... kynB> .(81).. <serin prot. .(2774) |
Methyloceanibacter caenitepidi	|
Pelagibacterium halotolerans  	| .(1329) <serin prot. .(2445) |
Rhodomicrobium vannielii ATC..	| .(2085) kynR> .(1494) |
Youhaiella tibetensis         	| .(3406) kynU> .(562). <kynR .(311). |
Bosea vaviloviae              	| .(72).. serin prot.> .(2637) kynR> .(656). kynB> .(2487) |
Bradyrhizobium amphicarpaeae  	| .(3840) <kynU .(1)... <kynB .(741). <serin prot. .(219). kynR> .(1675) |
Bradyrhizobium arachidis      	| .(4769) <kynU .(529). kynB> .(608). <serin prot. .(230). kynR> .(2858) |
Bradyrhizobium betae          	| .(160). <kynU .(1)... <kynB .(755). <serin prot. .(211). kynR> .(5446) |
Bradyrhizobium cosmicum       	| .(3984) <kynU .(1)... <kynB .(839). <serin prot. .(226). kynR> .(1707) |
Bradyrhizobium diazoefficien..	| .(4796) <kynU .(778). kynB> .(317). <serin prot. .(223). kynR> .(2019) |
Bradyrhizobium erythrophlei   	| .(295). <serin prot. .(104). <kynB .(6599) |
Bradyrhizobium guangdongense  	| .(515). <kynU .(244). | .(1730) <kynR .(199). serin prot.> .(1645) <kynU .(1)... <kynB .(3316) |
Bradyrhizobium guangzhouense  	| .(371). <kynU .(387). | .(1748) <kynR .(990). kynB> .(1)... kynU> .(95).. serin prot.> .(3780) |
Bradyrhizobium guangxiense    	| .(189). <kynU .(555). | .(4153) <kynU .(1)... <kynB .(785). <serin prot. .(221). kynR> .(1561) |
Bradyrhizobium icense         	| .(2186) serin prot.> .(3424) kynR> .(171). kynU> .(6)... <kynB .(1684) |
Bradyrhizobium japonicum USD..	| .(5343) <kynU .(1)... <kynB .(1247) kynR> .(1500) serin prot.> .(173). |
Bradyrhizobium oligotrophicu..	| .(1698) kynU> .(1)... kynB> .(3103) <serin prot. .(2279) |
Bradyrhizobium ottawaense     	| .(6075) <kynU .(1)... <kynB .(986). <serin prot. .(219). kynR> .(321). |
Bradyrhizobium paxllaeri      	| .(1813) <kynU .(160). <kynR .(73).. kynB> .(139). serin prot.> .(5372) |
Bradyrhizobium symbiodeficiens	| .(131). <serin prot. .(218). kynR> .(5445) <kynU .(1)... <kynB .(616). |
Bradyrhizobium vignae         	| .(4044) <kynU .(1)... <kynB .(735). <serin prot. .(209). kynR> .(2222) |
Bradyrhizobium zhanjiangense  	| .(4880) <kynU .(427). kynB> .(485). <serin prot. .(202). kynR> .(2224) |
Nitrobacter hamburgensis X14  	| .(2602) <serin prot. .(1290) |
Nitrobacter winogradskyi Nb-..	| .(2179) <serin prot. .(831). |
Afipia carboxidovorans OM5    	| .(1225) <kynB .(2142) |
Rhodopseudomonas palustris    	| .(2621) kynB> .(2154) |
Variibacter gotjawalensis     	| .(3808) <serin prot. .(115). <kynB .(484). |
Brucella abortus 2308         	|
Brucella canis ATCC 23365     	|
Brucella ceti TE10759-12      	|
Brucella inopinata            	|
Brucella melitensis bv. 1 st..	|
Brucella microti CCM 4915     	|
Brucella ovis ATCC 25840      	|
Brucella suis 1330            	|
Ochrobactrum anthropi         	| .(614). <kynR .(164). kynU> .(1031) <kynB .(64).. |
Ochrobactrum quorumnocens     	| .(924). kynR> .(829). |
Hartmannibacter diazotrophicus	|
Pseudorhodoplanes sinuspersici	| .(2024) <serin prot. .(1338) kynR> .(2197) kynB> .(81).. |
Hoeflea phototrophica DFL-43  	| .(2291) kynR> .(330). <kynU .(633). serin prot.> .(37).. <kynB .(814). |
Mesorhizobium amorphae CCNWG..	| .(200). <kynB .(476). | .(2076) <kynB <kynU .(2560) <serin prot. .(1172) |
Mesorhizobium australicum WS..	| .(494). kynB> .(1417) <kynU .(3501) <serin prot. .(347). |
Mesorhizobium ciceri biovar ..	| .(1513) <kynR .(1701) kynU> .(1435) kynB> .(910). serin prot.> .(310). |
Mesorhizobium erdmanii        	| .(2380) <kynU .(1224) kynB> .(2126) <serin prot. .(382). |
Mesorhizobium huakuii         	| .(3136) <serin prot. .(2745) <kynB .(427). |
Mesorhizobium jarvisii        	| .(103). <kynB .(2079) <kynU .(3958) <serin prot. .(373). |
Mesorhizobium japonicum MAFF..	| .(2167) kynB> .(481). serin prot.> .(3404) kynU> .(492). |
Mesorhizobium oceanicum       	| .(2588) kynU> .(153). <kynB <kynR .(2190) |
Mesorhizobium opportunistum ..	| .(2278) <kynB .(2)... <kynU .(3808) <serin prot. .(365). |
Mesorhizobium terrae          	| .(4090) <serin prot. .(1002) <kynU .(1)... kynB> .(277). |
Oricola thermophila           	| .(1387) kynR> .(1)... kynU> .(1704) <kynB .(708). |
Phyllobacterium zundukense    	| .(803). <kynR .(2968) | .(165). kynU> .(28).. | .(184). <kynB .(388). |
Roseitalea porphyridii        	| .(2961) <kynU .(434). |
Salaquimonas pukyongi         	| .(1830) <kynU .(1019) <kynB .(257). |
Labrenzia alexandrii DFL-11   	| .(1439) <serin prot. .(594). kynU> .(1495) kynB> .(1302) |
Stappia indica                	| .(3442) kynU> .(782). <kynB .(344). |
Lichenihabitans psoromatis    	| .(1636) <kynB .(2560) |
Martelella endophytica        	|
Martelella mediterranea DSM ..	| .(651). <kynB .(3574) |
Methylobacterium brachiatum   	| .(1374) <kynB .(2961) <serin prot. .(1294) |
Methylobacterium currus       	| .(4527) kynB> .(1261) | .(366). serin prot.> .(470). |
Methylobacterium durans       	| .(1923) <serin prot. .(3926) |
Methylobacterium mesophilicu..	| .(2987) <kynB .(3037) |
Methylobacterium nodulans OR..	| .(1642) <kynB .(5447) | .(37).. <serin prot. .(12).. |
Methylobacterium oryzae CBMB20	| .(3333) kynB> .(1031) serin prot.> .(1252) |
Methylobacterium phyllosphae..	| .(1874) <serin prot. .(981). <kynB .(1494) <kynB .(1082) |
Methylobacterium radiotolera..	| .(54).. kynB> .(433). | .(4158) serin prot.> .(1501) |
Methylobacterium terrae       	| .(5237) <serin prot. .(107). kynB> .(143). |
Methylorubrum extorquens PA1  	|
Methylorubrum populi          	| .(3491) kynB> .(1415) |
Microvirga ossetica           	| .(463). serin prot.> .(3381) <kynB .(1423) | .(1045) serin prot.> .(100). |
Microvirga thermotolerans     	| .(134). serin prot.> .(1795) kynB> .(1605) |
Methylocystis bryophila       	|
Methylocystis heyeri          	|
Methylocystis parvus          	|
Parvibaculum lavamentivorans..	|
Altererythrobacter atlanticus 	| .(2946) serin prot.> .(189). |
Altererythrobacter epoxidivo..	| .(1630) <serin prot. .(1025) kynU> .(67).. |
Altererythrobacter ishigakie..	| .(666). kynU> .(1198) serin prot.> .(662). |
Aurantiacibacter atlanticus   	| .(584). kynU> .(1521) <serin prot. .(711). |
Croceicoccus marinus          	| .(1254) serin prot.> .(1633) |
Erythrobacter aureus          	| .(688). kynU> .(2102) | .(427). <serin prot. .(61).. |
Erythrobacter litoralis       	| .(266). kynU> .(472). serin prot.> .(2304) |
Erythrobacter mangrovi        	| .(146). <serin prot. .(1139) kynU> .(1670) |
Erythrobacter neustonensis    	| .(210). <serin prot. .(506). <kynU .(2122) |
Paraurantiacibacter namhicola 	| .(240). <kynU .(715). serin prot.> .(1557) |
Pelagerythrobacter marensis   	| .(1626) <serin prot. .(391). <kynU .(665). |
Qipengyuania flava            	| .(182). <kynU .(1142) <serin prot. .(1367) |
Qipengyuania sediminis        	| .(1239) <serin prot. .(320). kynU> .(759). |
Qipengyuania seohaensis       	| .(1662) kynU> .(493). serin prot.> .(701). |
Tsuneonella amylolytica       	| .(604). <serin prot. .(381). <kynU .(1693) |
Tsuneonella dongtanensis      	| .(1587) serin prot.> .(871). <kynU .(431). |
Tsuneonella mangrovi          	| .(989). <kynU .(1064) serin prot.> .(516). |
Blastomonas fulva             	| .(2948) <serin prot. .(458). serin prot.> .(204). kynU> .(33).. |
Novosphingobium aromaticivor..	| .(1842) serin prot.> .(1524) |
Novosphingobium ginsenosidim..	| .(340). <serin prot. .(1286) <kynB .(1403) |
Novosphingobium pentaromativ..	| .(3165) serin prot.> .(488). |
Parasphingopyxis algicola     	| .(1147) kynU> .(2345) |
Rhizorhabdus dicambivorans    	| .(1800) <kynU .(123). kynB> .(2535) serin prot.> .(170). |
Sphingopyxis alaskensis RB2256	| .(549). <kynR .(828). <kynU .(1572) serin prot.> .(225). |
Sphingopyxis fribergensis     	| .(541). <serin prot. .(680). <kynR .(2034) <kynU .(1351) |
Sphingopyxis lindanitolerans  	| .(1201) kynU> .(1449) <serin prot. .(1200) |
Sphingopyxis macrogoltabida   	| .(127). kynU> aa perm.> .(3439) serin prot.> .(276). kynR> .(1104) |
Sphingomonas alpina           	| .(3310) kynU> .(1326) serin prot.> .(27).. |
Sphingomonas daechungensis    	|
Sphingomonas ginsengisoli An..	| .(1464) <serin prot. .(1464) |
Sphingomonas hengshuiensis    	| .(2103) serin prot.> .(2595) |
Sphingomonas lacunae          	| .(1028) serin prot.> .(199). kynB> .(1550) |
Sphingomonas lutea            	| .(158). <serin prot. .(1826) kynU> .(370). |
Sphingomonas melonis          	| .(1444) aa perm.> .(876). <kynU .(819). serin prot.> .(229). |
Sphingomonas paucimobilis     	| .(2058) serin prot.> .(1594) |
Sphingomonas panacis          	|
Sphingomonas rhizophila       	| .(1925) <serin prot. .(354). |
Sphingomonas sanxanigenens D..	| .(1936) <kynU .(3271) serin prot.> .(464). |
Sphingomonas sediminicola     	| .(1967) serin prot.> .(453). |
Sphingomonas taxi             	| .(721). aa perm.> .(469). <kynU .(579). <serin prot. .(1735) |
Sphingomonas wittichii RW1    	| .(691). kynU> .(3)... aa perm.> .(3176) <serin prot. .(1075) |
Sphingobium barthaii          	| .(548). <serin prot. .(2625) |
Sphingobium cloacae           	|
Sphingobium herbicidovorans   	| .(514). serin prot.> .(2275) |
Sphingobium hydrophobicum     	| .(2281) <serin prot. .(566). |
Sphingobium indicum B90A      	| .(1342) serin prot.> .(2087) |
Sphingobium japonicum UT26S   	| .(1474) <serin prot. .(1822) |
Allosphingosinicella indica   	| .(603). kynU> .(2139) |
Sphingorhabdus lacus          	| .(572). <serin prot. .(2273) <kynU .(293). kynB> .(93).. |
Tardibacter chloracetimidivo..	| .(2418) <serin prot. .(978). |
Zymomonas mobilis subsp. mob..	|
Sphingosinicella microcystin..	| .(1265) <kynU .(506). <kynB .(605). serin prot.> .(1351) |
Anaplasma centrale str. Israel	|
Anaplasma marginale str. Flo..	|
Anaplasma ovis str. Haibei    	|
Anaplasma phagocytophilum st..	|
Anaplasma platys              	|
Ehrlichia canis str. Jake     	|
Ehrlichia chaffeensis str. W..	|
Ehrlichia muris AS145         	|
Ehrlichia ruminantium         	|
Neorickettsia findlayensis    	|
Neorickettsia helminthoeca s..	|
Neorickettsia risticii str. ..	|
Neorickettsia sennetsu str. ..	|
Wolbachia pipientis           	|
Orientia tsutsugamushi        	|
Rickettsia akari str. Hartford	|
Rickettsia asiatica           	|
Rickettsia australis str. Cu..	|
Rickettsia conorii str. Mali..	|
Rickettsia helvetica C9P9     	|
Rickettsia heilongjiangensis  	|
Rickettsia japonica           	|
Rickettsia monacensis         	|
Rickettsia raoultii           	|
Rickettsia rickettsii str. I..	|
Rickettsia sibirica 246       	|
Rickettsia slovaca 13-B       	|
Rickettsia bellii RML369-C    	|
Rickettsia canadensis str. C..	|
Rickettsia prowazekii str. C..	|
Rickettsia typhi str. TH1527  	|
Asticcacaulis excentricus CB..	|
Brevundimonas subvibrioides ..	| .(914). kynB> .(2391) |
Brevundimonas vancanneytii    	| .(311). <kynB .(2169) serin prot.> .(583). |
Brevundimonas vesicularis     	| .(948). kynB> .(621). <serin prot. .(1771) |
Caulobacter flavus            	| .(486). <kynR .(875). <serin prot. .(3705) |
Caulobacter mirabilis         	| .(1465) kynU> .(2502) <serin prot. .(316). |
Caulobacter rhizosphaerae     	| .(70).. kynR> .(664). serin prot.> .(4390) |
Caulobacter segnis            	|
Caulobacter vibrioides NA1000 	|
Phenylobacterium zucineum HLK1	| .(1188) <serin prot. .(2689) |
Terricaulis silvestris        	| .(113). <kynB .(697). serin prot.> .(3064) |
Celeribacter ethanolicus      	| .(1023) kynB> .(2867) |
Celeribacter indicus          	| .(1145) kynR> .(3212) |
Celeribacter marinus          	|
Celeribacter manganoxidans    	|
Defluviimonas alba            	| .(1810) kynB> .(2392) |
Dinoroseobacter shibae DFL 1..	| .(706). <kynB .(1664) kynU> .(969). serin prot.> .(254). |
Epibacterium mobile F1926     	| .(534). kynU> .(1804) <kynB .(692). | .(654). serin prot.> .(488). |
Haematobacter massiliensis    	| .(149). <kynU kynB> .(2221) |
Halocynthiibacter arcticus    	| .(1634) kynB> .(836). <kynR .(1372) |
Ketogulonicigenium robustum   	|
Ketogulonicigenium vulgare    	|
Leisingera aquaemixtae        	| .(944). kynB> .(324). <serin prot. .(1518) kynU> .(814). |
Leisingera methylohalidivora..	| .(383). kynR> .(1880) kynU> .(544). <kynB .(1014) |
Marinovum algicola DG 898     	| .(160). <kynU .(1)... kynB> .(15).. | .(2101) <serin prot. .(1433) |
Octadecabacter antarcticus 307	| .(614). kynB> .(20).. kynU> .(3689) |
Octadecabacter arcticus 238   	| .(3276) <kynU .(18).. <kynB .(1290) |
Octadecabacter temperatus     	| .(2612) <kynU .(23).. <kynB .(568). |
Paracoccus aminophilus JCM 7..	| .(45).. kynU> .(322). | .(264). <kynB .(1)... |
Paracoccus aminovorans        	| .(71).. serin prot.> .(72).. |
Paracoccus contaminans        	| .(795). kynU> .(148). kynB> .(1760) |
Paracoccus denitrificans      	| .(1293) <kynU .(1490) | .(332). kynB> .(73).. <serin prot. .(190). |
Paracoccus jeotgali           	| .(2093) kynU> .(496). <kynB .(292). |
Paracoccus kondratievae       	| .(1925) <kynU .(247). | .(297). kynB> .(115). |
Paracoccus liaowanqingii      	| .(237). kynU> .(895). kynB> .(1895) | .(95).. serin prot.> .(184). |
Paracoccus mutanolyticus      	| .(2427) serin prot.> .(79).. |
Paracoccus yeei               	| .(2770) <serin prot. .(668). |
Paracoccus zhejiangensis      	| .(2113) <kynU kynB> .(1616) <serin prot. .(55).. |
Parasedimentitalea marina     	| .(124). kynB> .(1113) <kynU .(2878) |
Paraoceanicella profunda      	| .(10).. <kynR .(219). | .(950). <kynU .(766). kynB> .(671). <serin prot. .(130). kynR> .(978). |
Pelagibaca abyssi             	| .(77).. <kynU .(2258) serin prot.> .(287). kynB> .(1436) |
Phaeobacter gallaeciensis     	| .(85).. <kynU .(21).. | .(650). kynR> .(549). <kynU .(1758) <kynB .(486). |
Phaeobacter inhibens          	| .(392). kynR> .(519). <kynU .(1811) <kynB .(701). |
Phaeobacter porticola         	| .(43).. <kynU .(76).. | .(506). kynB> .(2237) <kynR .(607). |
Planktomarina temperata RCA23 	| .(200). <kynU .(2280) <serin prot. .(496). <kynB .(123). |
Polymorphum gilvum SL003B-26A1	| .(1867) kynB> .(1701) <kynU .(750). |
Profundibacter amoris         	| .(381). kynB> .(203). <kynR .(1801) kynU> .(1095) |
Pseudohalocynthiibacter aest..	| .(1649) serin prot.> .(1087) kynB> .(912). |
Pseudooceanicola algae        	| .(1753) <kynB .(1664) |
Pseudopuniceibacterium antar..	| .(876). <kynU .(1205) <kynB .(1900) |
Rhodobaca barguzinensis       	| .(1818) <kynU .(1351) kynB> .(447). |
Rhodobacter blasticus         	| .(571). kynB> .(8)... kynU> .(2816) |
Rhodobacter capsulatus        	| .(405). <kynB .(323). <kynU .(2596) |
Rhodobacter sphaeroides ATCC..	| .(1877) kynU> .(784). <kynB .(321). |
Rhodovulum sulfidophilum      	| .(748). <kynU .(2222) <serin prot. .(690). kynB> .(94).. |
Roseicitreum antarcticum      	| .(1185) <kynB .(1013) kynU> .(1009) |
Roseobacter denitrificans     	| .(1031) <kynB .(1066) <serin prot. .(307). kynU> .(1397) |
Roseobacter litoralis Och 149 	| .(1098) <kynR .(2260) <kynU .(370). serin prot.> .(292). <kynB .(133). |
Roseobacter ponti             	| .(2208) <kynU .(1228) <kynB .(223). |
Roseibacterium elongatum DSM..	| .(876). <kynB .(928). <kynU .(1372) |
Roseovarius indicus           	| .(3229) kynB> .(1)... kynU> .(849). kynB> .(1115) |
Ruegeria pomeroyi DSS-3       	| .(563). kynR> .(2262) kynU> .(1066) | .(211). <serin prot. .(205). kynB> .(36).. |
Silicimonas algicola          	| .(1320) <kynU .(87).. <kynB .(1787) <kynB .(150). serin prot.> .(889). |
Sulfitobacter pseudonitzschiae	| .(2281) <kynU .(1058) <kynB .(295). kynR> .(120). |
Tabrizicola piscis            	| .(32).. <kynR .(100). | .(6)... kynU> .(3077) <kynB .(154). <kynB .(727). |
Thalassobius gelatinovorus    	| .(1959) <kynB .(1388) kynU> .(342). |
Thioclava nitratireducens     	| .(1840) <kynU kynB> .(989). <serin prot. .(836). |
Paremcibacter congregatus     	| .(1929) <kynB .(1)... kynU> .(904). <serin prot. .(157). aa perm.> .(619). |
Glycocaulis alkaliphilus      	| .(1104) <serin prot. .(1430) <kynU .(279). |
Hirschia baltica ATCC 49814   	| .(476). kynR> .(2)... kynU> .(2581) <serin prot. .(41).. |
Hyphomonas neptunium ATCC 15..	| .(2519) kynR> .(799). <serin prot. .(154). |
Kordiimonas pumila            	| .(2506) <kynU .(8)... kynB> .(1019) |
Magnetococcus marinus MC-1    	|
Micavibrio aeruginosavorus A..	|
Phreatobacter cathodiphilus   	| .(49).. serin prot.> .(2368) kynB> .(1589) <serin prot. .(257). |
Phreatobacter stygius         	| .(3700) serin prot.> .(3072) <kynB .(54).. |
Parvularcula bermudensis HTC..	| .(2165) <serin prot. .(471). |
Achromobacter denitrificans   	| .(1071) kynB> .(1830) <kynR kynU> .(3246) |
Achromobacter insolitus       	| .(1261) <kynR kynU> aa perm.> .(42).. <kynB .(4548) |
Achromobacter spanius         	| .(2972) <kynB .(1686) <kynR kynU> aa perm.> .(988). |
Achromobacter xylosoxidans    	| .(621). <kynR kynU> aa perm.> .(4510) <kynB .(519). |
Advenella kashmirensis WT001  	| .(1158) kynR> .(739). kynU> .(196). <serin prot. .(1128) |
Advenella mimigardefordensis..	| .(2403) kynU> .(235). <serin prot. .(1603) |
Alcaligenes aquatilis         	| .(357). serin prot.> serin prot.> .(208). kynB> .(377). <kynR kynU> aa perm.> .(1514) <kynB .(939). |
Alcaligenes faecalis          	| .(122). <kynB .(1050) serin prot.> .(246). kynB> .(718). <kynR kynU> aa perm.> .(1606) |
Algicoccus marinus            	|
Basilea psittacipulmonis DSM..	|
Bordetella avium 197N         	| .(201). <kynR kynU> .(2304) <serin prot. .(213). <kynB .(586). |
Bordetella bronchialis        	| .(4162) <kynR .(467). kynB> .(599). |
Bordetella bronchiseptica     	| .(265). <kynR kynU> .(3933) kynB> .(567). |
Bordetella flabilis           	| .(603). <kynB .(1569) serin prot.> .(1805) <kynR .(1157) |
Bordetella hinzii             	| .(2384) kynB> .(735). <kynR kynU> .(1259) |
Bordetella holmesii           	| .(167). <kynR kynU> .(2604) kynB> .(461). |
Bordetella parapertussis      	| .(397). <kynU kynR> .(686). <kynB .(3076) |
Bordetella pertussis 18323    	| .(214). <kynR kynU> .(2956) <kynB .(323). |
Bordetella petrii             	| .(548). <kynU .(3694) <kynR .(134). kynB> .(530). |
Bordetella pseudohinzii       	| .(265). <kynR kynU> .(3365) kynB> .(468). |
Castellaniella defragrans 65..	| .(216). <kynR aa perm.> .(2444) <serin prot. .(832). |
Kerstersia gyiorum            	| .(3142) <kynB .(183). |
Orrella dioscoreae            	|
Pigmentiphaga aceris          	| .(1180) kynR> .(2447) kynU> .(444). kynB> .(1147) |
Taylorella asinigenitalis MCE3	|
Taylorella equigenitalis      	|
Acidovorax carolinensis       	| .(936). <kynU .(2674) |
Acidovorax citrulli AAC00-1   	| .(1594) kynB> .(591). serin prot.> .(1414) kynU> .(1133) |
Acidovorax monticola          	| .(3412) kynB> .(129). <kynU .(208). |
Alicycliphilus denitrificans  	|
Comamonas kerstersii          	| .(447). kynB> .(130). kynR> .(734). aa perm.> .(1877) |
Comamonas koreensis           	| .(171). kynB> .(2721) aa perm.> .(1)... kynU> .(1727) |
Comamonas piscis              	| .(163). kynB> .(2731) aa perm.> .(1)... kynU> .(1678) |
Comamonas serinivorans        	| .(606). <serin prot. .(1207) kynB> .(1956) |
Delftia lacustris             	| .(368). <kynB .(2284) aa perm.> .(5)... aa perm.> .(1)... kynU> .(3564) |
Delftia tsuruhatensis         	| .(232). <kynB .(2435) aa perm.> .(5)... aa perm.> .(1)... kynU> .(3815) |
Diaphorobacter aerolatus      	| .(101). serin prot.> .(3521) |
Diaphorobacter polyhydroxybu..	| .(301). <kynU .(3370) |
Diaphorobacter ruginosibacter 	| .(3566) aa perm.> .(839). <kynU .(78).. |
Hydrogenophaga crassostreae   	| .(2876) <kynB .(194). kynU> .(1424) |
Hydrogenophaga pseudoflava    	| .(2744) <kynB .(56).. <serin prot. .(383). kynU> .(1392) |
Ottowia oryzae                	| .(991). <kynU .(705). <kynB .(943). <kynR .(819). |
Polaromonas naphthalenivoran..	| .(3002) kynU> .(1105) | .(318). <serin prot. |
Polaromonas vacuolata         	| .(957). kynB> .(2405) |
Pulveribacter suum            	| .(1242) <kynU .(1742) <kynR .(28).. |
Ramlibacter tataouinensis TT..	| .(1046) <kynU .(1552) <kynB .(1071) kynB> .(236). |
Rhodoferax antarcticus        	| .(943). <kynR .(2628) |
Rhodoferax ferrireducens T118 	|
Rhodoferax koreense           	| .(442). kynB> .(925). kynU> .(2010) aa perm.> .(298). <kynB .(1138) <kynB .(464). |
Rhodoferax saidenbachensis    	| .(2577) kynU> .(994). kynB> .(397). |
Rhodoferax sediminis          	| .(3306) kynU> .(776). |
Schlegelella thermodepolymer..	| .(1840) <serin prot. .(100). <kynR .(434). kynU> .(1145) <kynB .(44).. |
Serpentinomonas mccroryi      	| .(2186) <serin prot. .(143). |
Serpentinomonas raichei       	| .(2181) <serin prot. .(154). |
Simplicispira suum            	| .(581). <kynB .(75).. kynU> .(2821) |
Variovorax paradoxus S110     	| .(1430) <kynU .(83).. kynB> .(3758) |
Verminephrobacter eiseniae E..	| .(2127) aa perm.> .(2778) |
Aquabacterium olei            	| .(1284) <kynB .(2101) |
Inhella inkyongensis          	| .(1274) serin prot.> .(353). <kynU .(2107) |
Leptothrix cholodnii SP-6     	| .(335). serin prot.> .(2612) <kynB .(1392) |
Methylibium petroleiphilum PM1	| .(256). serin prot.> .(3567) |
Rhizobacter gummiphilus       	| .(5573) <serin prot. .(264). |
Roseateles depolymerans       	| .(3663) kynU> .(211). <serin prot. .(944). |
Sphaerotilus natans subsp. s..	| .(810). <serin prot. .(1994) kynU> .(674). |
Thiomonas arsenitoxydans      	| .(1130) <kynU .(2150) kynB> .(163). |
Thiomonas intermedia          	| .(2224) kynB> .(308). <kynU .(433). |
Xylophilus rhododendri        	| .(3616) <kynB .(664). kynR> .(945). |
Burkholderia cepacia          	| .(620). <kynU .(1)... kynR> .(56).. aa perm.> .(956). <kynB .(1679) | .(2390) <serin prot. .(603). |
Burkholderia cenocepacia      	| .(601). <kynU .(1)... kynR> .(58).. aa perm.> .(860). <kynB .(1740) | .(1055) serin prot.> .(1642) |
Burkholderia dolosa AU0158    	| .(499). <kynU .(1)... kynR> .(57).. aa perm.> .(2528) | .(676). <serin prot. .(1171) |
Burkholderia metallica        	| .(569). <kynU .(1)... kynR> .(56).. aa perm.> .(860). <kynB .(1687) | .(1015) serin prot.> .(1538) |
Burkholderia multivorans ATC..	| .(484). <kynU .(1)... kynR> .(57).. aa perm.> .(2592) | .(668). serin prot.> .(1313) |
Burkholderia pyrrocinia       	| .(381). <kynB .(2251) <kynU .(1)... kynR> .(60).. aa perm.> .(494). | .(615). <serin prot. .(2150) |
Burkholderia seminalis        	| .(582). <kynU .(1)... kynR> .(58).. aa perm.> .(835). <kynB .(1650) | .(1078) serin prot.> .(1598) |
Burkholderia stagnalis        	| .(589). <kynU .(1)... kynR> .(59).. aa perm.> .(425). <kynR .(3)... aa perm.> .(2229) | .(539). <kynB .(1995) | .(426). <serin prot. .(298). |
Burkholderia stabilis         	| .(656). aa perm.> .(415). | .(351). aa perm.> .(58).. kynR> .(1)... <kynU .(2498) <kynB .(494). | .(2191) serin prot.> .(903). |
Burkholderia ubonensis        	| .(637). <kynU .(1)... kynR> .(62).. aa perm.> .(2897) | .(1708) <serin prot. .(918). |
Burkholderia glumae           	| .(1648) <kynU .(1)... kynR> .(948). <serin prot. .(489). |
Burkholderia mallei           	| .(225). <kynU .(1)... kynR> .(1094) <kynB .(1664) | .(1084) <serin prot. .(559). |
Burkholderia oklahomensis C6..	| .(2832) <kynU .(1)... kynR> .(53).. aa perm.> .(717). | .(1662) <kynB .(91).. serin prot.> .(458). aa perm.> .(3)... <kynR .(253). |
Burkholderia pseudomallei     	| .(150). kynB> .(511). aa perm.> .(56).. kynR> .(1)... <kynU .(2703) | .(2232) aa perm.> .(3)... <kynR .(194). <serin prot. .(10).. |
Burkholderia thailandensis E..	| .(706). <kynU .(1)... kynR> .(55).. aa perm.> .(2509) | .(379). serin prot.> .(1892) <kynR .(3)... aa perm.> .(88).. |
Burkholderia plantarii        	| .(543). <kynU .(1)... kynR> .(985). <serin prot. .(2034) |
Caballeronia insecticola      	| .(2129) <kynR .(1)... kynU> .(599). |
Chitinimonas arctica          	| .(18).. <serin prot. .(411). <kynU .(677). kynR> .(474). serin prot.> .(773). serin prot.> .(2264) |
Cupriavidus basilensis        	| .(1073) <kynU .(1)... kynR> .(998). <kynB .(753). serin prot.> .(181). aa perm.> .(1024) |
Cupriavidus gilardii          	| .(1036) kynB> .(5)... kynB> .(911). | .(2071) serin prot.> .(366). <kynR .(1)... kynU> .(596). |
Cupriavidus malaysiensis      	| .(1867) kynB> .(1025) | .(642). <kynU .(1)... kynR> .(1732) serin prot.> .(1551) |
Cupriavidus nantongensis      	| .(439). serin prot.> .(3091) <kynR .(1)... kynU> .(221). <kynB .(519). |
Cupriavidus neocaledonicus    	| .(396). <kynR .(3)... aa perm.> .(2017) | .(954). <serin prot. .(1391) <kynR .(1)... kynU> .(166). <kynB .(741). |
Cupriavidus necator H16       	| .(567). kynB> .(1976) | .(914). <kynU .(1)... kynR> .(1044) <serin prot. .(1673) |
Cupriavidus oxalaticus        	| .(992). <serin prot. .(1454) <kynR .(1)... kynU> .(191). <kynB .(762). |
Cupriavidus taiwanensis LMG ..	| .(1378) <kynB .(794). | .(894). <kynU .(1)... kynR> .(1138) serin prot.> .(1065) |
Ephemeroptericola cinctioste..	|
Lautropia mirabilis           	| .(1139) <serin prot. .(1383) |
Mycoavidus cysteinexigens     	|
Mycetohabitans rhizoxinica H..	| .(678). kynR> .(566). kynB> .(1000) |
Pandoraea apista              	| .(1331) kynB> .(839). aa perm.> .(1185) <kynR .(1)... aa perm.> .(28).. kynU> .(1429) |
Pandoraea faecigallinarum     	| .(1266) <kynU .(27).. <aa perm. .(1)... kynR> .(122). <kynB .(796). aa perm.> .(2264) |
Pandoraea fibrosis            	| .(759). kynB> .(1124) <aa perm. .(913). <kynR .(1)... aa perm.> .(27).. kynU> .(1979) |
Pandoraea norimbergensis      	| .(3118) <aa perm. .(814). kynB> .(131). <kynR .(1)... aa perm.> .(28).. kynU> .(1199) |
Pandoraea oxalativorans       	| .(1291) <kynU .(26).. <aa perm. .(1)... kynR> .(124). <kynB .(828). aa perm.> .(2455) |
Pandoraea pnomenusa           	| .(1340) <kynU .(28).. kynR> .(105). <kynB .(735). aa perm.> .(78).. <kynB .(2399) |
Pandoraea pulmonicola         	| .(3421) kynB> .(141). <kynR .(1)... aa perm.> .(27).. kynU> .(1337) |
Pandoraea sputorum            	| .(2747) <aa perm. .(908). kynB> .(132). <kynR .(1)... aa perm.> .(26).. kynU> .(1119) |
Pandoraea thiooxydans         	| .(3149) aa perm.> .(190). <kynR .(1)... kynU> .(707). |
Pandoraea vervacti            	| .(1280) <kynU .(26).. <aa perm. .(1)... kynR> .(128). <kynB .(785). aa perm.> .(2493) |
Paraburkholderia aromaticivo..	| .(1679) <kynU .(1)... kynR> .(2147) |
Paraburkholderia caffeinilyt..	| .(1198) <kynR .(1)... kynU> .(2741) | .(1075) <serin prot. .(1790) serin prot.> .(202). |
Paraburkholderia caribensis   	| .(1199) <kynR .(1)... kynU> .(1935) | .(1879) <serin prot. .(644). |
Paraburkholderia dokdonella   	| .(463). kynU> .(1)... <kynR .(1576) aa perm.> .(689). | .(126). aa perm.> .(972). |
Paraburkholderia graminis     	| .(2791) <kynR .(1)... kynU> .(698). |
Paraburkholderia phymatum ST..	| .(501). <kynU .(1)... kynR> .(2542) | .(1321) serin prot.> .(285). |
Paraburkholderia phytofirman..	| .(1429) aa perm.> .(1712) <kynR .(1)... kynU> .(741). |
Paraburkholderia sprentiae W..	| .(3136) <kynU .(1)... kynR> .(48).. |
Paraburkholderia terricola    	| .(2819) <kynR .(1)... kynU> .(706). | .(725). serin prot.> .(1902) |
Paraburkholderia terrae       	| .(503). <kynU .(1)... kynR> .(2619) | .(635). <serin prot. .(2038) |
Paraburkholderia tropica      	| .(687). aa perm.> .(1682) <kynR .(1)... kynU> .(487). |
Paraburkholderia xenovorans ..	| .(3551) <kynR .(1)... kynU> .(749). | .(562). <kynB .(690). |
Paraburkholderia acidophila   	| .(658). <kynU .(1)... kynR> .(62).. aa perm.> .(2654) | .(596). <kynB .(1187) <serin prot. .(1129) |
Polynucleobacter acidiphobus  	|
Polynucleobacter asymbioticu..	| .(1198) <kynB .(466). <kynB .(451). |
Polynucleobacter difficilis   	| .(1577) <kynB .(416). |
Polynucleobacter duraquae     	|
Polynucleobacter necessarius  	|
Polynucleobacter paneuropaeus 	|
Polynucleobacter wuianus      	|
Ralstonia insidiosa           	| .(643). aa perm.> .(545). <kynB .(387). aa perm.> .(92).. | .(761). kynU> .(1)... <kynR .(2026) <serin prot. .(905). |
Ralstonia mannitolilytica     	| .(439). aa perm.> .(856). | .(1001) <serin prot. .(1493) kynU> .(1)... <kynR .(674). |
Ralstonia pseudosolanacearum  	| .(765). <serin prot. .(1849) <kynR .(1)... kynU> .(751). |
Ralstonia solanacearum        	| .(542). <kynR .(1)... kynU> .(1508) <serin prot. .(1057) |
Collimonas arenae             	| .(935). <kynU .(1)... kynR> .(3163) |
Collimonas fungivorans        	| .(3700) <kynR .(1)... kynU> .(1116) |
Collimonas pratensis          	| .(3790) <kynR .(1)... kynU> .(993). <kynB .(171). |
Herminiimonas arsenitoxidans  	| .(2873) kynB> .(615). |
Herbaspirillum frisingense    	| .(691). <kynR .(872). serin prot.> .(2188) kynB> .(1056) |
Herbaspirillum huttiense      	| .(909). <kynR .(425). kynB> .(2592) kynB> .(197). serin prot.> .(846). |
Herbaspirillum robiniae       	| .(48).. aa perm.> .(3)... <kynU .(738). kynR> .(1892) <kynB .(2151) |
Herbaspirillum rubrisubalbic..	| .(846). <kynR .(735). serin prot.> .(2280) kynB> .(909). |
Herbaspirillum seropedicae    	| .(835). <kynR .(320). kynB> .(1213) serin prot.> .(1291) kynB> .(1104) |
Janthinobacterium agaricidam..	| .(1386) <kynU .(1086) serin prot.> .(2582) |
Janthinobacterium lividum     	| .(2072) <kynU .(3460) |
Janthinobacterium svalbarden..	| .(2134) <kynU .(1976) serin prot.> .(1335) |
Massilia albidiflava          	| .(2232) <kynB .(903). <serin prot. .(1490) kynU> .(1419) |
Massilia armeniaca            	| .(3749) kynU> .(1102) serin prot.> .(387). |
Massilia flava                	| .(1275) kynU> .(995). <serin prot. .(3586) |
Massilia lutea                	| .(1099) <kynB .(798). kynU> .(3627) <serin prot. .(570). |
Massilia oculi                	| .(2797) serin prot.> .(1431) <kynU .(763). |
Massilia putida               	| .(2281) kynR> .(489). serin prot.> serin prot.> .(1622) <kynU .(1561) |
Massilia umbonata             	| .(305). kynB> .(1175) serin prot.> .(524). <kynR .(1815) <kynU .(2257) |
Massilia violaceinigra        	| .(110). <serin prot. .(3463) <kynU .(2684) |
Oxalobacter formigenes        	| .(1703) serin prot.> .(501). |
Undibacterium parvum          	| .(58).. <kynB .(1541) <kynR .(1)... kynU> .(2581) |
Sutterella faecalis           	|
Sutterella megalosphaeroides  	|
Alysiella filiformis          	| .(526). <kynR .(1710) |
Chitinolyticbacter meiyuanen..	| .(2780) <serin prot. <serin prot. .(1271) |
Conchiformibius steedae       	| .(1177) kynR> .(817). |
Crenobacter cavernae          	|
Eikenella corrodens           	| .(660). kynR> .(1431) |
Eikenella exigua              	| .(1519) kynR> .(324). |
Kingella oralis               	| .(779). <kynR .(1675) |
Neisseria animaloris          	| .(1490) kynR> .(539). |
Neisseria animalis            	| .(1213) <kynR .(754). |
Neisseria bacilliformis       	| .(1985) <kynR .(303). |
Neisseria brasiliensis        	| .(495). kynR> .(1952) |
Neisseria canis               	| .(310). kynR> .(1985) |
Neisseria chenwenguii         	| .(1717) <kynR .(562). |
Neisseria cinerea             	| .(205). <kynR .(1485) |
Neisseria elongata            	| .(1259) kynR> .(928). |
Neisseria flavescens          	| .(1314) kynR> .(709). |
Neisseria gonorrhoeae         	| .(356). kynR> .(1666) |
Neisseria lactamica           	| .(266). <kynR .(1809) |
Neisseria meningitidis        	| .(183). <kynR .(1759) |
Neisseria musculi             	| .(1843) kynR> .(773). |
Neisseria polysaccharea       	| .(1365) <kynR .(486). |
Neisseria shayeganii          	| .(2090) <kynR .(180). |
Neisseria subflava            	| .(62).. <kynR .(1878) |
Neisseria wadsworthii         	| .(1081) <kynR .(1186) |
Neisseria weaveri             	| .(593). kynR> .(1416) |
Neisseria zalophi             	| .(616). kynR> .(1536) |
Neisseria zoodegmatis         	| .(805). <kynR .(1455) |
Simonsiella muelleri ATCC 29..	| .(954). <kynR .(1381) |
Snodgrassella alvi wkB2       	| .(816). <kynR .(706). kynU> aa perm.> .(683). |
Vitreoscilla filiformis       	|
Aquitalea denitrificans       	| .(2459) kynR> .(1598) |
Chitinibacter fontanus        	| .(2302) <serin prot. .(1004) |
Chromobacterium haemolyticum  	| .(80).. <serin prot. .(4202) <kynR .(1)... kynU> aa perm.> .(502). |
Chromobacterium paludis       	| .(3364) <serin prot. .(570). |
Chromobacterium phragmitis    	|
Chromobacterium vaccinii      	|
Chromobacterium violaceum AT..	|
Aromatoleum aromaticum EbN1   	| .(639). serin prot.> .(3278) |
Oryzomicrobium terrae         	| .(1769) kynR> .(1333) |
Azoarcus olearius             	|
Azoarcus pumilus              	| .(2449) serin prot.> .(649). |
Thauera aromatica K172        	| .(2151) serin prot.> .(1134) |
Thauera chlorobenzoica        	| .(1384) serin prot.> .(1980) |
Thauera humireducens          	|
Thauera hydrothermalis        	|
Dechloromonas aromatica RCB   	| .(513). kynR> .(3700) |
Casimicrobium huifangae       	| .(211). kynB> .(344). kynU> .(1048) serin prot.> .(603). <serin prot. .(1744) |
Denitratisoma oestradiolicum  	|
Sulfuritalea hydrogenivorans..	| .(2692) kynB> .(886). |
Ferriphaselus amnicola        	|
Gallionella capsiferriforman..	|
Sideroxydans lithotrophicus ..	|
Methylobacillus flagellatus KT	|
Methylovorus glucosetrophus ..	|
Methylophilus medardicus      	|
Methylotenera mobilis JLW8    	|
Methylotenera versatilis 301  	|
Nitrosospira briensis C-128   	|
Nitrosospira lacus            	| .(1393) serin prot.> .(1)... serin prot.> .(1430) |
Nitrosospira multiformis ATC..	| .(1869) <serin prot. .(863). |
Nitrosomonas europaea ATCC 1..	| .(1543) serin prot.> .(981). |
Nitrosomonas stercoris        	| .(340). serin prot.> .(1771) |
Nitrosomonas ureae            	| .(1418) <serin prot. .(1466) |
Sulfuritortus calidifontis    	|
Sulfuricella denitrificans s..	|
Sulfurimicrobium lacus        	|
Sulfuriferula nivalis         	| .(1858) <kynR .(1348) |
Sulfuriferula plumbiphila     	|
Acidithiobacillus caldus      	|
Acidithiobacillus ferridurans 	|
Acidithiobacillus ferrooxida..	|
Acidithiobacillus ferrivoran..	|
Acidithiobacillus thiooxidan..	| .(1240) <kynR .(2121) |
Aliarcobacter faecis          	|
Arcobacter butzleri ED-1      	|
Aliarcobacter cibarius        	|
Arcobacter cryaerophilus ATC..	|
Aliarcobacter lanthieri       	|
Arcobacter skirrowii CCUG 10..	|
Arcobacter trophiarum LMG 25..	|
Arcobacter anaerophilus       	| .(1112) aa perm.> .(1818) |
Arcobacter aquimarinus        	|
Arcobacter cloacae            	|
Arcobacter defluvii           	|
Arcobacter ellisii            	|
Arcobacter nitrofigilis DSM ..	|
Arcobacter peruensis          	|
Arcobacter suis CECT 7833     	|
Arcobacter venerupis          	|
Halarcobacter bivalviorum     	|
Halarcobacter ebronensis      	|
Malaciobacter canalis         	|
Malaciobacter halophilus      	|
Malaciobacter marinus         	|
Malaciobacter molluscorum LM..	|
Malaciobacter mytili LMG 24559	|
Malaciobacter pacificus       	| .(1762) serin prot.> .(813). |
Poseidonibacter lekithochrous 	| .(2513) serin prot.> .(808). |
Poseidonibacter parvus        	|
Pseudoarcobacter acticola     	|
Campylobacter armoricus       	|
Campylobacter avium LMG 24591 	|
Campylobacter blaseri         	| .(507). kynB> .(1291) |
Campylobacter canadensis      	|
Campylobacter corcagiensis    	|
Campylobacter concisus        	|
Campylobacter cuniculorum DS..	|
Campylobacter curvus          	|
Campylobacter fetus           	|
Campylobacter geochelonis     	|
Campylobacter gracilis        	|
Campylobacter hepaticus       	|
Campylobacter helveticus      	|
Campylobacter hyointestinali..	|
Campylobacter iguaniorum      	|
Campylobacter insulaenigrae ..	|
Campylobacter jejuni subsp. ..	|
Campylobacter lanienae NCTC ..	|
Campylobacter lari RM2100     	|
Campylobacter mucosalis       	|
Campylobacter ornithocola     	|
Campylobacter peloridis       	|
Campylobacter pinnipediorum ..	|
Campylobacter rectus          	|
Campylobacter showae          	|
Campylobacter sputorum bv. p..	|
Campylobacter subantarcticus..	|
Campylobacter upsaliensis RM..	|
Campylobacter volucris LMG 2..	|
Sulfurospirillum barnesii SE..	|
Sulfurospirillum cavolei      	|
Sulfurospirillum deleyianum ..	|
Sulfurospirillum halorespira..	|
Sulfurospirillum multivorans..	|
Helicobacter acinonychis str..	|
Helicobacter apodemus         	|
Helicobacter bizzozeronii CI..	|
Helicobacter canadensis       	|
Helicobacter cetorum MIT 99-..	|
Helicobacter cinaedi          	|
Helicobacter felis ATCC 49179 	|
Helicobacter hepaticus ATCC ..	|
Helicobacter himalayensis     	|
Helicobacter mustelae         	|
Helicobacter pylori Puno135   	|
Helicobacter pylori 26695-1CL 	|
Helicobacter pylori 26695-1CH 	|
Helicobacter typhlonius       	|
Helicobacter winghamensis     	|
Wolinella succinogenes DSM 1..	|
Nitratifractor salsuginis DS..	| .(251). <serin prot. .(1844) |
Sulfurovum lithotrophicum     	|
Sulfurimonas autotrophica DS..	|
Sulfurimonas denitrificans D..	|
Sulfurimonas gotlandica GD1   	|
Sulfurimonas paralvinellae    	|
Sulfuricurvum kujiense DSM 1..	|
Caminibacter mediatlanticus ..	|
Cetia pacifica                	|
Nautilia profundicola AmH     	|
Nitratiruptor labii           	| .(1072) <serin prot. .(894). |
Anaeromyxobacter dehalogenan..	| .(248). serin prot.> .(4211) |
Corallococcus coralloides DS..	| .(1714) <serin prot. .(392). <serin prot. .(4984) kynU> .(859). |
Corallococcus macrosporus DS..	| .(912). <kynU .(662). <serin prot. .(5468) |
Myxococcus hansupus           	| .(5041) serin prot.> .(542). kynU> .(1570) |
Myxococcus stipitatus DSM 14..	| .(916). <kynU .(651). <serin prot. .(6337) |
Myxococcus xanthus DK 1622    	| .(1984) <serin prot. .(3754) serin prot.> .(573). kynU> .(879). |
Melittangium boletus DSM 14713	| .(3893) kynU> .(1579) serin prot.> .(2486) |
Vulgatibacter incomptus       	| .(1922) <kynU .(875). kynR> .(786). |
Chondromyces crocatus         	| .(2489) serin prot.> .(5574) |
Pajaroellobacter abortibovis  	| .(1006) aa perm.> .(666). |
Sorangium cellulosum So ce56  	| .(1927) kynU> .(6800) serin prot.> .(688). |
Labilithrix luteola           	| .(2268) <kynR .(2952) kynU> .(490). <serin prot. .(4744) |
Minicystis rosea              	| .(1799) kynU> .(6129) <serin prot. .(4566) |
Sandaracinus amylolyticus     	| .(1823) <kynU .(6198) <serin prot. .(617). |
Haliangium ochraceum DSM 14365	| .(198). <serin prot. .(142). kynR> .(1182) kynU> .(3248) <serin prot. .(1944) |
Bradymonas sediminis          	| .(2173) serin prot.> .(454). kynU> .(1087) |
Persicimonas caeni            	| .(54).. serin prot.> .(1569) kynU> .(4257) |
Desulfurella acetivorans A63  	|
Hippea maritima DSM 10411     	|
Desulfobacca acetoxidans DSM..	|
Desulfomonile tiedjei DSM 6799	|
Syntrophus aciditrophicus SB  	|
Desulfoglaeba alkanexedens A..	| .(285). serin prot.> .(2607) |
Syntrophobacter fumaroxidans..	| .(2215) <serin prot. .(1756) |
Desulfocurvibacter africanus..	| .(1386) <serin prot. .(2362) |
Desulfovibrio alaskensis G20  	|
Desulfovibrio carbinolicus    	|
Desulfovibrio carbinoliphilu..	|
Desulfovibrio fairfieldensis  	|
Desulfovibrio ferrophilus     	|
Desulfovibrio gigas DSM 1382..	|
Desulfovibrio marinus         	| .(98).. <kynU .(3990) |
Desulfovibrio magneticus RS-1 	| .(3492) serin prot.> .(1004) |
Desulfovibrio sulfodismutans..	| .(3597) <serin prot. .(302). |
Desulfovibrio vulgaris RCH1   	|
Desulfovibrio hydrothermalis..	|
Desulfovibrio salexigens DSM..	|
Desulfolutivibrio sulfoxidir..	|
Lawsonia intracellularis N343 	| .(446). <kynU .(805). |
Pseudodesulfovibrio aespoeen..	|
Pseudodesulfovibrio piezophi..	|
Pseudodesulfovibrio profundus 	|
Desulfomicrobium baculatum D..	|
Desulfomicrobium orale DSM 1..	|
Desulfohalobium retbaense DS..	|
Desulfatibacillum aliphatici..	|
Desulfosarcina alkanivorans   	| .(5749) <serin prot. .(533). |
Desulfosarcina ovata subsp. ..	| .(6126) <serin prot. .(940). |
Desulfosarcina widdelii       	| .(6175) <serin prot. .(376). |
Desulfobacterium autotrophic..	| .(2524) <serin prot. .(2194) |
Desulfobacter hydrogenophilus 	| .(269). <serin prot. .(4092) |
Desulfobacter postgatei 2ac9  	|
Desulfococcus multivorans     	| .(2041) serin prot.> .(1725) |
Desulfococcus oleovorans Hxd3 	|
Desulfobacula toluolica Tol2  	|
Desulfurivibrio alkaliphilus..	|
Desulfobulbus oralis          	|
Desulfobulbus propionicus DS..	|
Desulfotalea psychrophila LS..	|
Desulfocapsa sulfexigens DSM..	|
Desulfarculus baarsii DSM 2075	|
Desulfuromonas soudanensis    	| .(3265) <serin prot. .(237). |
Pelobacter propionicus DSM 2..	|
Geobacter bemidjiensis Bem    	| .(2993) <serin prot. .(987). |
Geobacter bremensis           	|
Geobacter daltonii FRC-32     	| .(70).. <serin prot. .(3674) |
Geobacter lovleyi SZ          	| .(1691) <serin prot. .(1893) |
Geobacter metallireducens GS..	| .(748). <serin prot. .(1)... <serin prot. .(2800) |
Geobacter pickeringii         	| .(1049) serin prot.> .(2190) |
Geobacter sulfurreducens PCA  	| .(2029) <serin prot. .(1361) |
Geobacter uraniireducens Rf4  	| .(305). <serin prot. .(4151) |
Geoalkalibacter subterraneus  	| .(1613) serin prot.> .(1612) |
Syntrophotalea acetylenica    	| .(182). serin prot.> .(2653) |
Pelobacter carbinolicus DSM ..	|
Bacteriovorax stolpii         	| .(1490) serin prot.> .(2334) |
Halobacteriovorax marinus SJ  	| .(206). <kynU .(1053) serin prot.> .(2007) |
Bdellovibrio bacteriovorus H..	| .(2294) <serin prot. .(136). <serin prot. .(1110) |
Bdellovibrio exovorus JSS     	| .(129). <kynR .(36).. kynU> .(654). serin prot.> .(838). <serin prot. .(654). serin prot.> .(253). <serin prot. .(25).. |
Silvanigrella aquatica        	| .(1711) <kynU .(1089) |
Hydrogenophilus thermoluteolus	|
Mariprofundus aestuarium      	|
Mariprofundus ferrinatatus    	|
```
