# Supplementary material for: Evolutionarily stable gene clusters shed light on the common grounds of pathogenicity in the Acinetobacter calcoaceticus-baumannii complex
Source: PLoS Genet. 2022 Jun 2;18(6):e1010020. doi: 10.1371/journal.pgen.1010020 (PMC9162365; doi:10.1371/journal.pgen.1010020)
Supplement: S2 Data — (TAR.GZ) [file pgen.1010020.s014.tar.gz › Data_S2/pseudomonas_kyn_cluster_Proteobacteria.html]

```
Window reference accessions:	[{'NP_250769.1'}, {'NP_250770.1'}, {'NP_250771'}, {'NP_250772.1'}, {'NP_251269'}]
Pseudomonas aeruginosa PAO1                                 	| .(2080) <aa perm. <kynU <kynB kynR> .(492). <kynA .(2995) |
Pseudomonas aeruginosa PAO1H2O                              	| .(2007) <aa perm. <kynU <kynB kynR> .(478). <kynA .(2909) |
Pseudomonas citronellolis                                   	|
Pseudomonas furukawaii                                      	| .(3747) <aa perm. .(1)... <kynU <kynB kynR> .(1823) |
Pseudomonas pseudoalcaligenes CECT 5344                     	|
Pseudomonas mendocina S5.2                                  	|
Pseudomonas multiresinivorans                               	| .(3856) <kynB .(2009) |
Pseudomonas agarici                                         	|
Pseudomonas alkylphenolica                                  	|
Pseudomonas amygdali pv. tabaci str. ATCC 11528             	|
Pseudomonas cichorii JBC1                                   	|
Pseudomonas fuscovaginae                                    	| .(3229) kynB> .(2445) |
Pseudomonas syringae pv. tomato str. DC3000                 	|
Pseudomonas viridiflava                                     	|
Pseudomonas antarctica                                      	| .(3952) <aa perm. <kynU kynR> .(10).. <kynA <kynB .(1605) |
Pseudomonas arsenicoxydans                                  	|
Pseudomonas asplenii                                        	| .(4885) <kynB .(695). |
Pseudomonas azotoformans                                    	| .(3178) <aa perm. <kynU kynR> .(9)... <kynA <kynB .(2726) |
Pseudomonas brenneri                                        	| .(287). <aa perm. <kynU kynR> .(9)... <kynA <kynB .(5054) |
Pseudomonas cedrina                                         	|
Pseudomonas corrugata                                       	|
Pseudomonas extremorientalis                                	| .(2430) <aa perm. <kynU kynR> .(9)... <kynA <kynB .(3163) |
Pseudomonas fluorescens                                     	| .(1026) <aa perm. <kynU kynR> .(9)... <kynA <kynB .(4761) |
Pseudomonas mandelii JR-1                                   	|
Pseudomonas mediterranea                                    	|
Pseudomonas mucidolens                                      	| .(3625) <aa perm. <kynU kynR> .(2)... <kynA <kynB .(1478) |
Pseudomonas orientalis                                      	| .(4213) <aa perm. <kynU kynR> .(9)... <kynA <kynB .(901). |
Pseudomonas protegens CHA0                                  	| .(5371) <aa perm. <kynU kynR> .(7)... <kynA <kynB .(756). |
Pseudomonas rhodesiae                                       	| .(4412) <kynU kynR> .(1)... <kynA <kynB .(707). |
Pseudomonas synxantha                                       	| .(4810) <aa perm. <kynU kynR> .(9)... <kynA <kynB .(994). |
Pseudomonas trivialis                                       	|
Pseudomonas veronii                                         	| .(5105) <aa perm. <kynU kynR> .(9)... <kynA <kynB .(940). |
Pseudomonas balearica DSM 6083                              	|
Pseudomonas stutzeri                                        	|
Pseudomonas xanthomarina                                    	|
Pseudomonas brassicacearum                                  	| .(2086) kynB> .(3796) |
Pseudomonas chlororaphis                                    	| .(5251) <aa perm. <kynU kynR> .(5)... <kynA <kynB .(718). |
Pseudomonas lundensis                                       	|
Pseudomonas entomophila L48                                 	|
Pseudomonas extremaustralis                                 	|
Pseudomonas fulva                                           	|
Pseudomonas monteilii                                       	|
Pseudomonas mosselii                                        	|
Pseudomonas plecoglossicida                                 	|
Pseudomonas putida NBRC 14164                               	|
Pseudomonas glycinae                                        	|
Pseudomonas granadensis                                     	|
Pseudomonas guangdongensis                                  	|
Pseudomonas knackmussii                                     	|
Pseudomonas koreensis                                       	|
Pseudomonas kribbensis                                      	|
Pseudomonas lactis                                          	| .(4440) <aa perm. <kynU kynR> .(9)... <kynA <kynB .(911). |
Pseudomonas lalkuanensis                                    	|
Pseudomonas lini                                            	|
Pseudomonas litoralis                                       	|
Pseudomonas lurida                                          	| .(4487) <aa perm. <kynU kynR> .(9)... <kynA <kynB .(906). |
Pseudomonas marincola                                       	|
Pseudomonas oryzae                                          	|
Pseudomonas otitidis                                        	| .(2655) <kynA .(744). <aa perm. .(1)... <kynU <kynB kynR> .(2111) |
Pseudomonas pohangensis                                     	|
Pseudomonas prosekii                                        	|
Pseudomonas psychrophila                                    	|
Pseudomonas reinekei                                        	| .(4787) <kynA .(755). |
Pseudomonas rhizosphaerae                                   	|
Pseudomonas salegens                                        	|
Pseudomonas sabulinigri                                     	|
Pseudomonas sediminis                                       	|
Pseudomonas sihuiensis                                      	|
Pseudomonas silesiensis                                     	|
Pseudomonas simiae                                          	| .(4595) <aa perm. <kynU kynR> .(9)... <kynA <kynB .(895). |
Pseudomonas soli                                            	|
Pseudomonas thivervalensis                                  	| .(1585) <kynB .(4059) |
Pseudomonas umsongensis                                     	| .(5153) <aa perm. .(1)... <kynU kynR> .(5)... <kynA <kynB .(739). |
Pseudomonas vancouverensis                                  	|
Pseudomonas versuta                                         	|
Pseudomonas xinjiangensis                                   	|
Pseudomonas yamanorum                                       	| .(5185) <aa perm. <kynU kynR> .(10).. <kynA <kynB .(932). |
Azotobacter chroococcum                                     	|
Azotobacter salinestris                                     	|
Azotobacter vinelandii DJ                                   	| .(2507) <kynR .(2188) |
Entomomonas moraniae                                        	|
Oblitimonas alkaliphila                                     	|
Permianibacter aggregans                                    	| .(1177) kynU> .(2340) <kynA .(239). |
Acinetobacter baumannii                                     	| .(1066) <aa perm. <kynU kynR> .(2576) |
Acinetobacter calcoaceticus                                 	| .(2145) <aa perm. <kynU kynR> .(1629) |
Acinetobacter lactucae                                      	| .(2093) <aa perm. <kynU kynR> .(1481) |
Acinetobacter nosocomialis M2                               	| .(153). <aa perm. <kynU kynR> .(3293) |
Acinetobacter pittii PHEA-2                                 	| .(1436) <aa perm. <kynU kynR> .(2160) |
Acinetobacter seifertii                                     	| .(2114) <aa perm. <kynU kynR> .(1524) |
Acinetobacter baylyi ADP1                                   	| .(1604) <kynB .(1608) |
Acinetobacter chinensis                                     	|
Acinetobacter cumulans                                      	|
Acinetobacter defluvii                                      	|
Acinetobacter dispersus                                     	| .(150). <kynR .(3442) |
Acinetobacter equi                                          	|
Acinetobacter guillouiae                                    	| .(2690) <aa perm. <kynU kynR> .(1448) |
Acinetobacter haemolyticus                                  	|
Acinetobacter junii                                         	|
Acinetobacter larvae                                        	|
Acinetobacter lanii                                         	|
Acinetobacter oleivorans DR1                                	| .(2247) <aa perm. <kynU kynR> .(1564) |
Acinetobacter schindleri                                    	|
Acinetobacter shaoyimingii                                  	|
Acinetobacter wanghuae                                      	|
Moraxella bovoculi                                          	|
Moraxella bovis                                             	|
Moraxella catarrhalis BBH18                                 	|
Moraxella cuniculi                                          	|
Moraxella nonliquefaciens                                   	|
Moraxella osloensis                                         	|
Moraxella ovis                                              	|
Psychrobacter alimentarius                                  	|
Psychrobacter arcticus 273-4                                	|
Psychrobacter cryohalolentis K5                             	|
Psychrobacter urativorans                                   	|
Acidihalobacter aeolianus                                   	|
Acidihalobacter ferrooxydans                                	|
Alkalilimnicola ehrlichii MLHE-1                            	|
Aquisalimonas sp. 2447                                      	|
Halorhodospira halochloris                                  	|
Halorhodospira halophila SL1                                	|
Spiribacter curvatus                                        	|
Spiribacter roseus                                          	|
Spiribacter salinus M19-40                                  	|
Thioalkalivibrio nitratireducens DSM 14787                  	|
Thioalkalivibrio paradoxus ARh 1                            	|
Thioalkalivibrio sulfidiphilus HL-EbGr7                     	|
Thioalkalivibrio versutus                                   	|
Allochromatium vinosum DSM 180                              	|
Marichromatium purpuratum 984                               	|
Nitrosococcus halophilus Nc 4                               	| .(1136) kynA> kynU> .(2545) |
Nitrosococcus oceani ATCC 19707                             	| .(1907) <kynB .(1186) |
Nitrosococcus watsonii C-113                                	|
Nitrosococcus wardiae                                       	| .(3307) <kynU <kynA .(362). |
Thermochromatium tepidum ATCC 43061                         	|
Thioflavicoccus mobilis 8321                                	|
Thiocystis violascens DSM 198                               	|
Granulosicoccus antarcticus IMCC3135                        	| .(687). kynB> .(818). kynA> kynU> .(5131) |
Sulfuriflexus mobilis                                       	|
Guyparkeria halophila                                       	|
Halothiobacillus neapolitanus c2                            	|
Sulfurivermis fontis                                        	|
Wenzhouxiangella marina                                     	| .(1394) kynR> .(395). <kynA .(323). kynU> .(494). <kynB .(448). |
Woeseia oceani                                              	| .(845). kynU> .(294). kynA> .(2404) |
Actinobacillus delphinicola                                 	|
Actinobacillus equuli subsp. equuli                         	|
Actinobacillus pleuropneumoniae                             	|
Actinobacillus porcitonsillarum                             	|
Actinobacillus suis ATCC 33415                              	|
Aggregatibacter actinomycetemcomitans                       	|
Aggregatibacter aphrophilus ATCC 33389                      	|
Aggregatibacter segnis ATCC 33393                           	|
Avibacterium volantium                                      	| .(318). kynB> .(1687) |
Basfia succiniciproducens                                   	|
[Mannheimia] succiniciproducens MBEL55E                     	|
Bibersteinia trehalosi USDA-ARS-USMARC-188                  	|
Bisgaardia hudsonensis                                      	|
Frederiksenia canicola                                      	|
Glaesserella parasuis SH0165                                	|
Haemophilus aegyptius                                       	|
[Haemophilus] ducreyi                                       	|
Haemophilus haemolyticus                                    	|
Haemophilus influenzae                                      	|
Haemophilus pittmaniae                                      	|
Histophilus somni                                           	|
Mannheimia haemolytica USMARC_2286                          	| .(146). kynB> .(2382) |
Mannheimia ovis                                             	| .(482). <kynB .(1483) |
Mannheimia varigena USDA-ARS-USMARC-1312                    	|
Otariodibacter oris                                         	|
Pasteurella dagmatis                                        	|
Pasteurella multocida                                       	| .(1073) kynB> .(975). |
Pasteurella skyensis                                        	|
Rodentibacter heylii                                        	|
Vespertiliibacter pulmonis                                  	|
Aeromonas allosaccharophila                                 	|
Aeromonas encheleia                                         	|
Aeromonas hydrophila                                        	|
Aeromonas media                                             	|
Aeromonas salmonicida                                       	|
Aeromonas simiae                                            	|
Aeromonas veronii                                           	|
Oceanisphaera avium                                         	|
Oceanisphaera profunda                                      	|
Tolumonas auensis DSM 9187                                  	|
Zobellella denitrificans                                    	| .(2861) <kynU <kynB <kynA .(946). |
Aerosticca soli                                             	| .(649). kynA> .(504). <kynU .(1443) |
Ahniella affigens                                           	| .(531). <kynB .(1545) kynU> .(2350) <kynA .(29).. |
Dokdonella koreensis DS-123                                 	| .(612). <kynU .(195). kynA> .(2756) |
Dyella thiooxydans                                          	| .(1572) kynU> .(1916) kynA> .(280). |
Frateuria aurantia DSM 6220                                 	| .(1101) kynA> .(823). kynU> .(1179) |
Luteibacter pinisoli                                        	| .(833). kynA> .(1975) kynU> .(1289) |
Luteibacter rhizovicinus DSM 16549                          	| .(733). <kynB .(1347) <kynA .(2073) <kynU .(115). |
Rhodanobacter denitrificans                                 	| .(2653) kynU> .(670). <kynA .(446). <kynB .(64).. |
Arenimonas daejeonensis                                     	| .(854). kynA> .(636). <kynU .(762). |
Luteimonas chenhongjianii                                   	| kynA> .(1559) <kynU .(1351) |
Luteimonas granuli                                          	| .(1680) kynA> .(789). <kynU .(20).. |
Lysobacter alkalisoli                                       	| .(1454) <kynU .(1272) <kynA .(576). |
Lysobacter antibioticus                                     	| .(1321) kynB> .(1128) kynU> .(1393) <kynA .(956). |
Lysobacter capsici                                          	| .(2515) kynU> .(861). <kynB .(611). <kynA .(1108) |
Lysobacter enzymogenes                                      	| .(1338) kynB> .(1165) kynU> .(1438) <kynA .(891). |
Lysobacter gummosus                                         	| .(1086) kynA> .(1441) <kynU .(1098) <kynB .(1322) |
Lysobacter lycopersici                                      	| .(487). <kynU .(1670) kynA> .(339). |
Lysobacter maris                                            	| .(716). kynA> .(879). <kynU .(1804) |
Lysobacter oculi                                            	| .(770). <kynU .(911). <kynA .(715). |
Lysobacter soli                                             	| .(636). kynA> .(1628) kynU> .(1392) |
Pseudolysobacter antarcticus                                	| .(2439) kynU> .(816). <kynA .(690). |
Pseudoxanthomonas mexicana                                  	| .(1203) kynA> .(1150) <kynU .(1291) |
Pseudoxanthomonas spadix BD-a59                             	| .(1460) <kynU .(1415) <kynA .(210). |
Stenotrophomonas acidaminiphila                             	| .(42).. kynR> .(428). kynA> .(974). <kynU .(2161) |
Stenotrophomonas maltophilia                                	| .(373). kynA> .(1154) <kynU .(2659) <kynR .(50).. |
Stenotrophomonas rhizophila                                 	| .(243). kynA> .(1248) <kynU .(2497) |
Thermomonas brevis                                          	| .(27).. <kynU .(1368) <kynA .(1686) |
Xanthomonas albilineans                                     	| .(420). kynA> .(935). <kynU .(1537) <kynR .(54).. |
Xanthomonas cassavae CFBP 4642                              	| .(615). kynA> .(2535) kynU> .(1020) |
Xanthomonas campestris pv. raphani                          	| .(427). kynA> .(2008) kynU> .(1604) |
Xanthomonas citri                                           	| .(429). kynA> .(1122) <kynU .(2561) |
Xanthomonas cucurbitae                                      	| .(395). kynA> .(878). <kynU .(2415) |
Xanthomonas euroxanthea                                     	| .(428). kynA> .(1151) <kynU .(2406) |
Xanthomonas fragariae                                       	| .(1416) <kynU .(1741) <kynA .(60).. |
Xanthomonas hortorum                                        	| .(258). <kynU .(3116) <kynA .(776). |
Xanthomonas hyacinthi                                       	| .(2095) kynA> .(1236) <kynU .(675). |
Xanthomonas oryzae pv. oryzicola                            	| .(292). kynA> .(916). <kynU .(2262) |
Xanthomonas phaseoli pv. dieffenbachiae LMG 695             	| .(1147) kynA> .(1986) kynU> .(845). |
Xanthomonas vesicatoria ATCC 35937                          	| .(2731) kynA> .(1190) <kynU .(183). |
Xylella fastidiosa Temecula1                                	|
Xylella taiwanensis                                         	|
Agarilytica rhodophyticola                                  	| .(191). <kynU .(5319) |
Cellvibrio japonicus Ueda107                                	|
Saccharophagus degradans 2-40                               	|
Simiduia agarivorans SA1 = DSM 21679                        	| .(3298) kynA> .(444). |
Congregibacter litoralis KT71                               	| .(2344) <kynU .(1461) |
Halioglobus maricola                                        	| .(187). <kynB .(3678) |
Kineobactrum salinum                                        	| .(569). <kynB .(2725) kynU> .(617). |
Microbulbifer aggregans                                     	| .(1474) <kynA .(1813) |
Microbulbifer agarilyticus                                  	| .(2759) kynA> .(387). kynB> .(309). |
Microbulbifer hydrolyticus                                  	| .(413). <kynB .(450). <kynA .(2579) |
Microbulbifer thermotolerans                                	| .(574). <kynA .(2645) |
Oceanicoccus sagamiensis                                    	|
Zhongshania aliphaticivorans                                	|
Alcanivorax borkumensis SK2                                 	|
Alcanivorax dieselolei B5                                   	|
Alcanivorax pacificus W11-5                                 	|
Ketobacter alkanivorans                                     	|
Bermanella marisrubri                                       	|
Marinobacterium aestuarii                                   	| .(3482) <kynU <kynB <kynA .(976). |
Marinomonas arctica                                         	| .(1164) kynB> .(2894) |
Marinomonas mediterranea MMB-1                              	| .(3401) kynU> .(702). |
Marinomonas posidonica IVIA-Po-181                          	|
Marinomonas primoryensis                                    	|
Neptunomonas concharum                                      	|
Neptunomonas phycophila                                     	|
Thalassolituus oleivorans MIL-1                             	|
Chromohalobacter salexigens DSM 3043                        	|
Cobetia marina                                              	|
Halomonas aestuarii                                         	|
Halomonas beimenensis                                       	| .(457). <kynA .(114). kynU> .(3078) |
Halomonas campisalis                                        	| .(3302) <kynU <kynB <kynA .(739). |
Halomonas chromatireducens                                  	| .(1652) kynA> kynU> .(1843) |
Halomonas elongata DSM 2581                                 	| .(519). kynU> kynA> .(3185) |
Halomonas huangheensis                                      	| .(2838) <kynU .(1196) |
Halomonas hydrothermalis                                    	|
Halomonas piezotolerans                                     	|
Halomonas socia                                             	|
Halomonas subglaciescola                                    	|
Halomonas titanicae                                         	| .(1147) <kynU <kynA .(2221) <kynR .(1335) |
Kushneria konosiri                                          	|
Kushneria marisflavi                                        	|
Pistricoccus aurantiacus                                    	|
Salinicola tamaricis                                        	| .(185). kynA> .(2840) |
Zymobacter palmae                                           	|
Endozoicomonas montiporae CL-33                             	| .(4527) kynA> .(199). |
Gynuella sunshinyii YC6258                                  	|
Reinekea forsetii                                           	| .(1277) <kynU .(1944) |
Saccharospirillum mangrovi                                  	|
Hahella chejuensis KCTC 2396                                	|
Spartinivicinus ruber                                       	| .(2785) kynU> .(2323) kynA> .(459). |
Kangiella geojedonensis                                     	| .(888). <kynA .(1077) kynU> .(288). |
Kangiella koreensis DSM 16069                               	| .(365). <kynU .(1145) kynA> .(1088) |
Kangiella profundi                                          	| .(319). <kynU .(1097) kynA> .(1004) |
Kangiella sediminilitoris                                   	| .(931). <kynA .(1077) kynU> .(296). |
Litoricola lipolytica                                       	|
Oleiphilus messinensis                                      	|
Aliivibrio salmonicida LFI1238                              	|
Grimontia hollisae                                          	| .(288). <kynB .(2493) |
Paraphotobacterium marinum                                  	| .(279). kynB> .(735). |
Photobacterium gaetbulicola Gung47                          	|
Salinivibrio kushneri                                       	|
Vibrio alfacsensis                                          	|
Vibrio antiquarius                                          	|
Vibrio diabolicus                                           	|
Vibrio azureus                                              	|
Vibrio campbellii                                           	| .(248). <kynA .(1416) |
Vibrio harveyi                                              	|
Vibrio jasicida 090810c                                     	|
Vibrio natriegens NBRC 15636 = ATCC 14048 = DSM 759         	|
Vibrio owensii                                              	|
Vibrio parahaemolyticus                                     	|
Vibrio rotiferianus                                         	|
Vibrio anguillarum                                          	|
Vibrio aphrogenes                                           	|
Vibrio aquimaris                                            	| .(868). kynA> .(227). |
Vibrio astriarenae                                          	|
Vibrio atlanticus                                           	|
Vibrio cholerae MS6                                         	|
Vibrio cyclitrophicus                                       	|
Vibrio europaeus                                            	|
Vibrio tubiashii ATCC 19109                                 	|
Vibrio fluvialis                                            	|
Vibrio furnissii                                            	|
Vibrio kanaloae                                             	|
Vibrio metoecus                                             	|
Vibrio metschnikovii                                        	|
Vibrio mediterranei                                         	|
Vibrio navarrensis                                          	|
Vibrio ponticus                                             	|
Vibrio qinghaiensis                                         	|
Vibrio rumoiensis                                           	|
Vibrio scophthalmi                                          	|
Vibrio spartinae                                            	|
Vibrio tapetis subsp. tapetis                               	|
Vibrio taketomensis                                         	|
Vibrio tritonius                                            	|
Vibrio vulnificus                                           	|
Allofrancisella frigidaquae                                 	|
Allofrancisella guangzhouensis                              	|
Francisella adeliensis                                      	|
Francisella frigiditurris                                   	|
Francisella halioticida                                     	|
Francisella hispaniensis                                    	|
Francisella marina                                          	|
Francisella noatunensis subsp. noatunensis FSC774           	|
Francisella opportunistica                                  	|
Francisella orientalis LADL--07-285A                        	|
Francisella orientalis FNO12                                	|
Francisella orientalis FNO24                                	|
Francisella persica ATCC VR-331                             	|
Francisella philomiragia                                    	|
Francisella salina                                          	|
Francisella uliginis                                        	|
Beggiatoa leptomitoformis                                   	|
Hydrogenovibrio crunogenus XCL-2                            	|
Hydrogenovibrio marinus                                     	|
Hydrogenovibrio thermophilus                                	|
Methylophaga frappieri                                      	|
Methylophaga nitratireducenticrescens                       	|
Piscirickettsia salmonis                                    	| .(1269) <kynB .(1689) |
Thiomicrospira aerophila AL3                                	|
Thiomicrospira cyclica ALM1                                 	|
Thiomicrorhabdus aquaedulcis                                	|
Thiomicrorhabdus indica                                     	|
Thiosulfatimonas sediminis                                  	|
Thiosulfativibrio zosterae                                  	|
Alteromonas addita                                          	| .(1883) <kynA .(1966) |
Alteromonas australica                                      	|
Alteromonas mediterranea                                    	| .(949). kynA> .(474). kynU> .(2311) |
Alteromonas naphthalenivorans                               	| .(2427) <kynA .(1751) |
Alteromonas pelagimontana                                   	| .(2830) kynA> .(824). |
Alteromonas stellipolaris LMG 21856                         	| .(2281) <kynA .(1586) |
Catenovulum sediminis                                       	|
Glaciecola amylolytica                                      	| .(3233) kynA> .(54).. <kynU .(513). |
Glaciecola nitratireducens FR1064                           	| .(2380) <kynU .(1037) |
Hydrocarboniclastica marina                                 	|
Marinobacter adhaerens HP15                                 	|
Marinobacter fonticola                                      	|
Marinobacter hydrocarbonoclasticus ATCC 49840               	|
Marinobacter psychrophilus                                  	|
Marinobacter salarius                                       	|
Marinobacter salinus                                        	|
Saliniradius amylolyticus                                   	| .(829). kynA> .(1815) kynU> .(277). |
Salinimonas lutimaris                                       	| .(2579) <kynA .(979). |
Salinimonas sediminis                                       	| .(993). kynA> .(541). <kynU .(2155) |
Colwellia beringensis                                       	| .(675). <kynA .(1)... <kynU .(3129) |
Colwellia psychrerythraea 34H                               	|
Litorilituus sediminis                                      	| .(3379) <kynR .(285). |
Thalassotalea crassostreae                                  	| .(2546) <kynU .(200). kynA> .(503). |
Ferrimonas balearica DSM 9799                               	| .(1531) kynB> .(1922) <kynU .(309). |
Idiomarina andamanensis                                     	| .(269). kynA> .(1931) |
Idiomarina loihiensis L2TR                                  	| .(2180) kynU> .(461). |
Moritella marina ATCC 15381                                 	| .(2485) kynU> .(1532) |
Moritella yayanosii                                         	|
Parashewanella spongiae                                     	|
Parashewanella tropica                                      	|
Shewanella algae                                            	| .(3017) <kynU <kynA .(1263) |
Shewanella amazonensis SB2B                                 	| .(3421) <kynU .(240). |
Shewanella baltica OS678                                    	| .(301). kynU> .(4069) |
Shewanella bicestrii                                        	| .(3667) <kynU .(316). |
Shewanella decolorationis                                   	| .(2252) kynU> .(1771) |
Shewanella denitrificans OS217                              	| .(3443) kynU> .(321). |
Shewanella donghaensis                                      	| .(3447) kynA> .(559). |
Shewanella frigidimarina NCIMB 400                          	|
Shewanella halifaxensis HAW-EB4                             	|
Shewanella japonica                                         	| .(255). <kynU .(3795) |
Shewanella khirikhana                                       	| .(3195) <kynU .(838). |
Shewanella livingstonensis                                  	|
Shewanella loihica PV-4                                     	| .(3576) <kynU .(314). |
Shewanella maritima                                         	| .(2573) kynA> .(1202) |
Shewanella marisflavi                                       	| .(3362) <kynU .(287). |
Shewanella oneidensis MR-1                                  	| .(177). <kynU .(3951) |
Shewanella pealeana ATCC 700345                             	| .(3888) <kynU .(345). |
Shewanella piezotolerans WP3                                	| .(233). kynU> .(4156) |
Shewanella polaris                                          	|
Shewanella psychrophila                                     	| .(1875) <kynA .(3399) |
Shewanella putrefaciens CN-32                               	| .(388). kynU> .(3550) |
Shewanella sediminis HAW-EB3                                	|
Shewanella violacea DSS12                                   	|
Shewanella woodyi ATCC 51908                                	| .(1392) <kynA .(1)... <kynU .(3475) |
Pseudoalteromonas agarivorans                               	|
Pseudoalteromonas aliena SW19                               	| .(19).. kynU> .(72).. |
Pseudoalteromonas arctica A 37-1-2                          	| .(53).. kynU> .(3237) |
Pseudoalteromonas carrageenovora                            	| .(51).. kynU> .(3071) |
Pseudoalteromonas donghaensis                               	| .(91).. kynU> .(3027) |
Pseudoalteromonas espejiana DSM 9414                        	|
Pseudoalteromonas issachenkonii                             	| .(577). kynU> .(2393) |
Pseudoalteromonas luteoviolacea                             	| .(3324) kynU> .(376). |
Pseudoalteromonas paragorgicola KMM 3548                    	| .(21).. kynU> .(656). |
Pseudoalteromonas phenolica                                 	| .(1065) kynU> .(2243) |
Pseudoalteromonas piratica                                  	| .(861). <kynA .(560). |
Pseudoalteromonas prydzensis ACAM 620                       	| .(294). <kynU .(19).. |
Pseudoalteromonas rubra                                     	| .(11).. kynU> .(9)... |
Pseudoalteromonas spongiae UST010723-006                    	| .(570). kynA> .(829). |
Pseudoalteromonas tetraodonis                               	| .(533). kynU> .(2441) |
Pseudoalteromonas translucida                               	| .(50).. kynU> .(2766) |
Pseudoalteromonas tunicata                                  	|
Pseudoalteromonas undina                                    	| .(415). kynU> .(153). |
Psychromonas ingrahamii 37                                  	|
Aquicella lusitana                                          	|
Aquicella siphonis                                          	| .(1997) kynB> .(172). |
Coxiella burnetii RSA 493                                   	|
Fluoribacter dumoffii Tex-KL                                	|
Legionella adelaidensis                                     	|
Legionella anisa                                            	| .(2315) kynU> .(1205) |
Legionella clemsonensis                                     	| .(933). kynB> .(1860) |
Legionella fallonii LLAP-10                                 	| .(217). kynU> .(1916) <kynB .(1256) |
Legionella hackeliae                                        	| .(1868) <kynB .(1071) |
Legionella israelensis                                      	|
Legionella lansingensis                                     	| .(851). kynB> .(1793) |
Legionella pneumophila                                      	|
Legionella sainthelensi                                     	|
Legionella spiritensis                                      	|
Legionella waltersii                                        	| .(2047) kynB> .(754). <kynB .(496). |
Tatlockia micdadei                                          	|
Atlantibacter hermannii                                     	|
Buttiauxella agrestis                                       	|
Cedecea lapagei                                             	|
Cedecea neteri                                              	|
Citrobacter amalonaticus                                    	|
Citrobacter freundii                                        	|
Citrobacter portucalensis                                   	|
Citrobacter werkmanii                                       	|
Citrobacter rodentium ICC168                                	|
Cronobacter condimenti 1330                                 	|
Cronobacter dublinensis subsp. dublinensis LMG 23823        	|
Cronobacter malonaticus LMG 23826                           	|
Cronobacter muytjensii ATCC 51329                           	|
Cronobacter sakazakii                                       	|
Cronobacter universalis NCTC 9529                           	|
Enterobacter asburiae                                       	|
Enterobacter cancerogenus                                   	|
Enterobacter chengduensis                                   	|
Enterobacter cloacae                                        	|
Enterobacter ludwigii                                       	|
Enterobacter roggenkampii                                   	|
Enterobacter sichuanensis                                   	|
Enterobacter oligotrophicus                                 	|
Enterobacter soli                                           	|
[Enterobacter] lignolyticus                                 	|
Pluralibacter gergoviae                                     	| .(3279) <kynB .(1607) |
Escherichia albertii                                        	|
Escherichia coli O26 str. RM8426                            	|
Escherichia coli O26 str. RM10386                           	|
Escherichia coli O103 str. RM8385                           	|
Escherichia coli O43 str. RM10042                           	|
Escherichia coli O111 str. RM9322                           	|
Escherichia coli O121 str. RM8352                           	|
Escherichia coli O145 str. RM9872                           	|
Escherichia coli O157:H7 str. Sakai                         	|
Escherichia coli str. K-12 substr. MG1655                   	|
Escherichia fergusonii                                      	|
Escherichia marmotae                                        	|
Klebsiella aerogenes                                        	|
Klebsiella huaxiensis                                       	|
Klebsiella michiganensis                                    	|
Klebsiella pneumoniae subsp. pneumoniae HS11286             	|
Klebsiella quasipneumoniae                                  	| .(1144) kynB> .(3682) |
Klebsiella variicola                                        	| .(1144) kynB> .(4021) |
Kluyvera intermedia                                         	|
Kosakonia arachidis                                         	|
Kosakonia cowanii                                           	|
Kosakonia oryzae                                            	|
Kosakonia pseudosacchari                                    	|
Kosakonia radicincitans                                     	|
Kosakonia sacchari                                          	|
Leclercia adecarboxylata                                    	|
Lelliottia amnigena                                         	|
Lelliottia jeotgali                                         	|
Lelliottia nimipressuralis                                  	|
Phytobacter diazotrophicus                                  	|
Phytobacter ursingii                                        	|
Raoultella electrica                                        	|
Raoultella ornithinolytica                                  	| .(3801) <aa perm. .(1239) |
Raoultella planticola                                       	| .(2192) <aa perm. .(2754) |
Raoultella terrigena                                        	|
Salmonella bongori                                          	|
Salmonella enterica subsp. enterica serovar Muenchen        	|
Salmonella enterica subsp. enterica serovar Thompson        	|
Salmonella enterica subsp. enterica serovar Typhimurium st..	|
Salmonella enterica subsp. enterica serovar Enteritidis st..	|
Scandinavium goeteborgense                                  	|
Shimwellia blattae DSM 4481 = NBRC 105725                   	|
Shigella dysenteriae                                        	|
Shigella flexneri 2a str. 301                               	|
Brenneria goodwinii                                         	|
Brenneria nigrifluens DSM 30175 = ATCC 13028                	|
Brenneria rubrifaciens                                      	|
Dickeya aquatica                                            	| .(1541) kynB> .(2319) |
Dickeya chrysanthemi Ech1591                                	|
Dickeya dadantii 3937                                       	|
Dickeya dianthicola                                         	|
Dickeya fangzhongdai                                        	|
Dickeya paradisiaca Ech703                                  	|
Dickeya poaceiphila                                         	|
Dickeya solani IPO 2222                                     	|
Dickeya zeae                                                	|
Lonsdalea britannica                                        	|
Lonsdalea populi                                            	|
Pectobacterium atrosepticum                                 	|
Pectobacterium brasiliense                                  	|
Pectobacterium carotovorum                                  	|
Pectobacterium odoriferum                                   	|
Pectobacterium parmentieri                                  	|
Pectobacterium polaris                                      	|
Pectobacterium punjabense                                   	|
Pectobacterium versatile                                    	|
Pectobacterium wasabiae CFBP 3304                           	|
Buchnera aphidicola (Diuraphis noxia)                       	|
Buchnera aphidicola str. Bp (Baizongia pistaciae)           	|
Erwinia amylovora CFBP1430                                  	|
Erwinia billingiae Eb661                                    	| .(875). <kynB .(3680) |
Erwinia gerundensis                                         	| .(810). <kynB .(2538) |
Erwinia pyrifoliae                                          	|
Erwinia tasmaniensis Et1/99                                 	|
Mixta calida                                                	|
Mixta gaviniae                                              	|
Mixta intestinalis                                          	| .(12).. kynB> .(72).. |
Pantoea agglomerans                                         	|
Pantoea alhagi                                              	|
Pantoea ananatis PA13                                       	|
Pantoea eucalypti                                           	| .(221). <kynB .(269). |
Pantoea stewartii                                           	|
Pantoea vagans                                              	|
Tatumella citrea                                            	|
Wigglesworthia glossinidia endosymbiont of Glossina morsit..	|
Chania multitudinisentens RB-25                             	| .(3613) <kynA .(14).. <aa perm. .(1125) |
Gibbsiella quercinecans                                     	| .(4173) <kynB .(643). |
Rahnella aquatilis CIP 78.65 = ATCC 33071                   	|
Rouxiella badensis                                          	|
Serratia ficaria                                            	| .(2937) <kynA .(85).. <aa perm. .(1671) |
Serratia fonticola                                          	| .(4745) <kynA .(32).. <aa perm. .(470). |
Serratia marcescens                                         	| .(4061) <kynA .(58).. <aa perm. .(501). |
Serratia nematodiphila                                      	| .(2941) <kynA .(65).. <aa perm. .(1823) |
Serratia plymuthica AS9                                     	| .(3025) <kynA .(73).. <aa perm. .(1837) |
Serratia quinivorans                                        	| .(3047) <kynA .(63).. <aa perm. .(1748) |
Serratia rubidaea                                           	| .(4153) <kynA .(77).. <aa perm. .(318). |
Serratia surfactantfaciens                                  	| .(2337) <kynA .(71).. <aa perm. .(2326) |
Yersinia aldovae 670-83                                     	|
Yersinia canariae                                           	|
Yersinia enterocolitica                                     	|
Yersinia entomophaga                                        	|
Yersinia hibernica                                          	|
Yersinia intermedia                                         	|
Yersinia mollaretii ATCC 43969                              	|
Yersinia pestis A1122                                       	|
Yersinia pseudotuberculosis                                 	|
Yersinia similis                                            	|
Yersinia rohdei                                             	|
Yersinia ruckeri                                            	|
Edwardsiella anguillarum ET080813                           	|
Edwardsiella hoshinae                                       	| .(2133) kynB> .(1098) |
Edwardsiella ictaluri 93-146                                	|
Edwardsiella tarda                                          	|
Hafnia alvei                                                	|
Leminorella richardii                                       	|
Limnobaculum parvum                                         	|
Pragia fontium                                              	|
Photorhabdus asymbiotica                                    	|
Photorhabdus laumondii subsp. laumondii TTO1                	| .(1351) kynA> .(2605) <kynB .(672). |
Photorhabdus thracensis                                     	| .(2013) kynB> .(2056) |
Providencia alcalifaciens                                   	|
Providencia heimbachae                                      	|
Providencia rettgeri                                        	|
Providencia sneebia DSM 19967                               	|
Providencia stuartii MRSN 2154                              	|
Providencia vermicola                                       	|
Proteus terrae subsp. cibarius                              	|
Proteus hauseri                                             	|
Proteus mirabilis HI4320                                    	|
Xenorhabdus bovienii SS-2004                                	|
Xenorhabdus doucetiae                                       	|
Xenorhabdus hominickii                                      	|
Xenorhabdus nematophila                                     	|
Xenorhabdus poinarii G6                                     	| .(2246) kynB> .(967). |
Plesiomonas shigelloides                                    	|
Sodalis praecaptivus                                        	| .(30).. kynB> .(3988) |
Cardiobacterium hominis                                     	|
Dichelobacter nodosus VCS1703A                              	|
Frischella perrara                                          	|
Gallaecimonas mangrovi                                      	| .(1171) kynA> .(2568) |
Pseudohongiella spirulinae                                  	| .(2688) <kynU .(336). |
Sedimenticola thiotaurini                                   	|
Thiolapillus brandeum                                       	|
Immundisolibacter cernigliae                                	|
Methylotuvimicrobium alcaliphilum 20Z                       	|
Methylotuvimicrobium buryatense                             	|
Methylomicrobium album BG8                                  	| .(1898) <kynB .(1845) |
Methylococcus capsulatus str. Bath                          	|
Methylomonas denitrificans                                  	|
Methylomonas rhizoryzae                                     	|
Methylocaldum marinum                                       	| .(2039) kynU> .(74).. <kynB .(3158) kynA> .(40).. |
Steroidobacter denitrificans                                	|
Sulfuricaulis limicola                                      	|
Sulfurifustis variabilis                                    	|
Acetobacter ascendens                                       	|
Acetobacter oryzifermentans                                 	|
Acetobacter oryzoeni                                        	|
Acetobacter pasteurianus 386B                               	|
Acetobacter senegalensis                                    	|
Acidibrevibacterium fodinaquatile                           	| .(2931) <kynB .(636). |
Acidiphilium multivorum AIU301                              	|
Asaia bogorensis NBRC 16594                                 	|
Gluconobacter albidus                                       	|
Gluconobacter oxydans DSM 3504                              	|
Gluconobacter thailandicus                                  	|
Granulibacter bethesdensis CGDNIH4                          	|
Komagataeibacter hansenii                                   	|
Komagataeibacter medellinensis NBRC 3288                    	|
Komagataeibacter nataicola                                  	|
Komagataeibacter rhaeticus                                  	|
Komagataeibacter saccharivorans                             	| .(579). kynB> .(2133) |
Komagataeibacter xylinus                                    	| .(2798) kynB> .(202). |
Kozakia baliensis                                           	|
Neokomagataea tanensis                                      	|
Oecophyllibacter saccharovorans                             	|
Parasaccharibacter apium                                    	|
Stella humosa                                               	|
Swingsia samuiensis                                         	|
Azospirillum humicireducens                                 	| .(380). <kynA <kynU .(230). |
Azospirillum oryzae                                         	| .(815). kynU> kynA> .(93).. |
Azospirillum ramasamyi                                      	| .(265). <kynA <kynU .(273). |
Azospirillum thermophilum                                   	| .(244). <kynB .(283). |
Azospirillum thiophilum                                     	|
Defluviicoccus vanus                                        	|
Ferrovibrio terrae                                          	| .(366). kynA> .(3762) |
Haematospirillum jordaniae                                  	|
Hypericibacter adhaerens                                    	| .(4795) <kynB .(375). |
Hypericibacter terrae                                       	|
Indioceanicola profundi                                     	| .(3)... kynB> .(385). | .(2910) <kynU .(113). kynA> .(202). |
Magnetospirillum gryphiswaldense MSR-1 v2                   	|
Magnetospirillum magneticum AMB-1                           	|
Nitrospirillum amazonense CBAmc                             	| .(656). kynU> kynA> kynB> .(572). |
Niveispirillum cyanobacteriorum                             	| .(40).. kynB> .(58).. <kynU .(243). <kynU .(433). |
Pararhodospirillum photometricum DSM 122                    	|
Rhodospirillum rubrum F11                                   	|
Skermanella pratensis                                       	| .(221). <kynA <kynU .(4953) |
Thalassospira indica                                        	| .(1470) kynU> kynA> .(2758) |
Thalassospira marina                                        	| .(119). kynB> .(444). <kynA <kynU .(153). |
Tistrella mobilis KA081020-065                              	|
Agrobacterium tumefaciens                                   	| .(64).. kynB> .(1896) |
Neorhizobium galegae bv. orientalis str. HAMBI 540          	| .(2417) <kynR .(1451) kynU> .(555). | .(23).. kynA> .(1596) |
Rhizobium acidisoli                                         	| .(2638) <kynR .(1606) |
Rhizobium esperanzae                                        	| .(3057) <kynR .(1172) |
Rhizobium etli                                              	| .(88).. kynB> .(318). | .(2877) <kynR .(1140) |
Rhizobium favelukesii                                       	| .(2689) <kynR .(1249) |
Rhizobium flavum                                            	| .(2228) <kynR .(99).. kynB> .(1519) |
Rhizobium hidalgonense                                      	| .(1201) kynR> .(2885) |
Rhizobium indicum                                           	| .(3872) kynR> .(882). |
Rhizobium jaguaris                                          	| .(1605) kynU> .(665). | .(3099) kynB> .(845). kynA> .(251). |
Rhizobium oryzihabitans                                     	| .(2777) kynR> .(202). |
Rhizobium phaseoli                                          	| .(2999) <kynR .(1232) |
Rhizobium pseudoryzae                                       	| .(1198) <kynR .(1202) kynU> kynA> .(1070) |
Rhizobium pusense                                           	| .(1820) <kynB .(31).. | .(1682) <kynR .(974). |
Rhizobium rhizoryzae                                        	| .(1166) <kynR .(1137) <kynA <kynU .(792). |
Rhizobium tropici CIAT 899                                  	| .(2426) <kynR .(333). kynB> .(878). | .(1181) <kynA .(50).. kynU> .(596). |
Ciceribacter thiooxidans                                    	| .(781). <kynB .(2675) | .(319). <kynR .(886). |
Ensifer adhaerens                                           	| .(1768) kynB> .(698). kynR> .(1414) | .(328). kynU> .(6)... <kynA .(46).. kynU> .(865). |
Ensifer alkalisoli                                          	| .(1369) <kynR .(1976) |
Ensifer mexicanus                                           	| .(1901) <kynR .(791). kynB> .(1235) |
Ensifer sojae CCBAU 05684                                   	| .(1405) <kynR .(590). kynB> .(1349) |
Sinorhizobium americanum                                    	| .(1802) kynR> .(442). kynB> .(1196) |
Sinorhizobium fredii CCBAU 25509                            	| .(1663) kynR> .(432). kynB> .(1690) |
Sinorhizobium meliloti 2011                                 	| .(1557) kynR> .(573). kynB> .(1190) |
Georhizobium profundi                                       	| .(301). kynU> .(1883) kynA> .(1948) |
Liberibacter crescens                                       	|
Ancylobacter pratisalsi                                     	| .(2103) <kynB .(2066) |
Azorhizobium caulinodans ORS 571                            	|
Pseudolabrys taiwanensis                                    	| .(3915) <kynA kynU> .(1324) |
Starkeya novella DSM 506                                    	| .(3542) kynB> .(892). |
Bartonella alsatica                                         	|
Bartonella ancashensis                                      	|
Bartonella australis Aust/NH1                               	|
Bartonella bacilliformis KC583                              	|
Bartonella bovis 91-4                                       	|
Bartonella clarridgeiae 73                                  	|
Bartonella elizabethae                                      	|
Bartonella grahamii as4aup                                  	|
Bartonella henselae                                         	|
Bartonella kosoyi                                           	|
Bartonella krasnovii                                        	|
Bartonella quintana                                         	|
Bartonella tribocorum CIP 105476                            	|
Beijerinckia indica subsp. indica ATCC 9039                 	| .(3243) <kynB .(329). |
Methylovirgula ligni                                        	|
Methylocella silvestris BL2                                 	|
Blastochloris tepida                                        	|
Blastochloris viridis                                       	|
Devosia ginsengisoli                                        	|
Hyphomicrobium denitrificans ATCC 51888                     	|
Hyphomicrobium nitrativorans NL23                           	|
Maritalea myrionectae                                       	| .(2865) kynU> kynA> .(452). |
Methyloceanibacter caenitepidi                              	|
Pelagibacterium halotolerans                                	|
Rhodomicrobium vannielii ATCC 17100                         	|
Youhaiella tibetensis                                       	|
Bosea vaviloviae                                            	| .(813). kynA> .(4756) kynB> .(284). |
Bradyrhizobium amphicarpaeae                                	| .(2638) kynA> kynU> .(3840) |
Bradyrhizobium arachidis                                    	| .(4227) kynA> kynU> .(4769) |
Bradyrhizobium betae                                        	| .(160). <kynU <kynA .(6415) |
Bradyrhizobium cosmicum                                     	| .(2775) kynA> kynU> .(1825) kynB> .(2158) |
Bradyrhizobium diazoefficiens USDA 110                      	| .(3339) kynA> kynU> .(4796) |
Bradyrhizobium erythrophlei                                 	| .(3005) <kynB .(3994) |
Bradyrhizobium guangdongense                                	| .(3317) kynA> kynU> .(3576) | .(244). <kynU <kynA .(514). |
Bradyrhizobium guangzhouense                                	| .(2298) kynB> .(441). kynA> kynU> .(3876) | .(370). <kynA <kynU .(387). |
Bradyrhizobium guangxiense                                  	| .(2570) kynA> kynU> .(4153) | .(555). <kynU <kynA .(188). |
Bradyrhizobium icense                                       	| .(275). <kynR .(1414) <kynA <kynU .(5783) |
Bradyrhizobium japonicum USDA 6                             	| .(2392) kynB> .(530). kynA> kynU> .(5343) |
Bradyrhizobium oligotrophicum S58                           	| .(1698) kynU> kynA> .(5384) |
Bradyrhizobium ottawaense                                   	| .(6075) <kynU <kynA .(1529) |
Bradyrhizobium paxllaeri                                    	| .(2125) <kynB .(3621) kynU> kynA> .(1812) |
Bradyrhizobium symbiodeficiens                              	| .(617). kynA> kynU> .(1682) kynB> .(4113) |
Bradyrhizobium vignae                                       	| .(4044) <kynU <kynA .(3169) |
Bradyrhizobium zhanjiangense                                	| .(2744) kynB> .(595). kynA> kynU> .(4880) |
Nitrobacter hamburgensis X14                                	|
Nitrobacter winogradskyi Nb-255                             	|
Afipia carboxidovorans OM5                                  	| .(652). kynB> .(2715) |
Rhodopseudomonas palustris                                  	| .(3401) <kynB .(1374) |
Variibacter gotjawalensis                                   	| .(3080) <kynB .(1328) |
Brucella abortus 2308                                       	|
Brucella canis ATCC 23365                                   	|
Brucella ceti TE10759-12                                    	|
Brucella inopinata                                          	|
Brucella melitensis bv. 1 str. 16M                          	|
Brucella microti CCM 4915                                   	|
Brucella ovis ATCC 25840                                    	|
Brucella suis 1330                                          	|
Ochrobactrum anthropi                                       	| .(1101) kynB> .(774). |
Ochrobactrum quorumnocens                                   	|
Hartmannibacter diazotrophicus                              	|
Pseudorhodoplanes sinuspersici                              	| .(2860) <kynB .(1433) <kynA kynU> .(1131) <kynB .(215). |
Hoeflea phototrophica DFL-43                                	| .(1982) kynB> .(638). <kynA <kynU .(542). <kynR .(943). |
Mesorhizobium amorphae CCNWGS0123                           	| .(159). kynB> .(517). | .(668). <kynR .(1408) <kynU .(689). <kynA .(3043) |
Mesorhizobium australicum WSM2073                           	| .(3849) kynU> <kynA .(1911) |
Mesorhizobium ciceri biovar biserrulae                      	| .(2657) <kynU <kynA .(3214) |
Mesorhizobium erdmanii                                      	| .(3734) kynU> <kynA .(2379) |
Mesorhizobium huakuii                                       	| .(5885) kynA> .(424). |
Mesorhizobium jarvisii                                      	| .(4332) kynU> <kynA .(2182) |
Mesorhizobium japonicum MAFF 303099                         	| .(491). kynA> <kynU .(6054) |
Mesorhizobium oceanicum                                     	| .(2588) kynU> kynA> .(1176) kynB> .(1167) |
Mesorhizobium opportunistum WSM2075                         	| .(4174) kynU> <kynA .(2280) |
Mesorhizobium terrae                                        	| .(279). kynU> kynA> .(5092) |
Oricola thermophila                                         	| .(1389) kynU> kynA> .(220). <kynB .(2191) |
Phyllobacterium zundukense                                  	| .(165). kynU> kynA> .(27).. |
Roseitalea porphyridii                                      	| .(2382) kynB> .(578). <kynU .(434). |
Salaquimonas pukyongi                                       	| .(1277) kynU> kynA> .(713). <kynB .(1115) |
Labrenzia alexandrii DFL-11                                 	| .(2798) <kynU .(772). <kynB .(683). kynA> .(577). |
Stappia indica                                              	| .(1125) <kynB <kynA <kynU .(3442) |
Lichenihabitans psoromatis                                  	| .(612). kynB> .(3584) |
Martelella endophytica                                      	| .(2073) kynR> .(2335) |
Martelella mediterranea DSM 17316                           	|
Methylobacterium brachiatum                                 	|
Methylobacterium currus                                     	|
Methylobacterium durans                                     	|
Methylobacterium mesophilicum SR1.6/6                       	|
Methylobacterium nodulans ORS 2060                          	|
Methylobacterium oryzae CBMB20                              	|
Methylobacterium phyllosphaerae                             	|
Methylobacterium radiotolerans JCM 2831                     	|
Methylobacterium terrae                                     	| .(3743) <kynB .(1745) |
Methylorubrum extorquens PA1                                	| .(3324) kynB> .(1515) |
Methylorubrum populi                                        	|
Microvirga ossetica                                         	| .(1227) kynA> .(4041) |
Microvirga thermotolerans                                   	| .(556). kynA> .(2979) |
Methylocystis bryophila                                     	| .(203). <kynB .(3870) |
Methylocystis heyeri                                        	|
Methylocystis parvus                                        	| .(264). kynB> .(3539) |
Parvibaculum lavamentivorans DS-1                           	|
Altererythrobacter atlanticus                               	|
Altererythrobacter epoxidivorans                            	| .(67).. <kynU <kynB <kynA .(2654) |
Altererythrobacter ishigakiensis                            	| .(1861) <kynU <kynB <kynA .(664). |
Aurantiacibacter atlanticus                                 	| .(583). kynA> kynU> .(2233) |
Croceicoccus marinus                                        	|
Erythrobacter aureus                                        	| .(2102) <kynU <kynB <kynA .(686). |
Erythrobacter litoralis                                     	| .(2777) <kynU <kynB <kynA .(264). |
Erythrobacter mangrovi                                      	| .(1670) <kynU <kynB <kynA .(1284) |
Erythrobacter neustonensis                                  	| .(717). <kynU <kynA .(2121) |
Paraurantiacibacter namhicola                               	| .(240). <kynU <kynA .(2272) |
Pelagerythrobacter marensis                                 	| .(281). kynB> .(382). kynA> kynU> .(2018) |
Qipengyuania flava                                          	| .(182). <kynU <kynB <kynA .(2508) |
Qipengyuania sediminis                                      	| .(759). <kynU <kynB <kynA .(1558) |
Qipengyuania seohaensis                                     	| .(1195) <kynU <kynB <kynA .(1660) |
Tsuneonella amylolytica                                     	| .(986). <kynU .(222). <kynA .(1470) |
Tsuneonella dongtanensis                                    	| .(229). <kynA .(2229) <kynU .(431). |
Tsuneonella mangrovi                                        	| .(989). <kynU .(468). kynA> .(1112) |
Blastomonas fulva                                           	| .(1226) kynB> .(1753) kynA> .(631). kynU> .(33).. |
Novosphingobium aromaticivorans DSM 12444                   	|
Novosphingobium ginsenosidimutans                           	| .(235). kynA> .(2795) |
Novosphingobium pentaromativorans US6-1                     	| .(2870) <kynA .(783). |
Parasphingopyxis algicola                                   	| .(1147) kynU> .(65).. <kynB .(1652) <kynA .(626). |
Rhizorhabdus dicambivorans                                  	| .(1800) <kynU <kynA .(2829) |
Sphingopyxis alaskensis RB2256                              	| .(1378) <kynU <kynB <kynA .(1796) |
Sphingopyxis fribergensis                                   	| .(3257) <kynU <kynB <kynA .(1349) |
Sphingopyxis lindanitolerans                                	| .(1200) kynA> kynU> .(2650) |
Sphingopyxis macrogoltabida                                 	| .(2548) kynA> kynB> .(2271) <aa perm. <kynU .(127). |
Sphingomonas alpina                                         	| .(878). kynB> .(475). <kynU <kynA .(3309) |
Sphingomonas daechungensis                                  	| .(869). <kynA .(1421) |
Sphingomonas ginsengisoli An et al. 2013                    	| .(2152) kynA> .(776). |
Sphingomonas hengshuiensis                                  	| .(1465) kynB> .(2753) <kynA .(479). |
Sphingomonas lacunae                                        	| .(1088) <kynA .(1690) |
Sphingomonas lutea                                          	| .(370). <kynU .(1400) kynR> .(246). <kynA .(337). |
Sphingomonas melonis                                        	| .(1049) kynU> .(876). <aa perm. .(968). <kynA .(475). |
Sphingomonas paucimobilis                                   	|
Sphingomonas panacis                                        	| .(4000) kynB> .(517). |
Sphingomonas rhizophila                                     	| .(2083) kynA> .(196). |
Sphingomonas sanxanigenens DSM 19645 = NX02                 	| .(1936) <kynU .(2932) kynA> .(803). |
Sphingomonas sediminicola                                   	| .(124). <kynB .(2045) kynA> .(250). |
Sphingomonas taxi                                           	| .(802). kynA> .(1512) kynU> .(469). <aa perm. .(721). |
Sphingomonas wittichii RW1                                  	| .(1497) kynB> .(2754) <aa perm. .(3)... <kynU .(691). |
Sphingobium barthaii                                        	| .(597). <kynB .(378). |
Sphingobium cloacae                                         	|
Sphingobium herbicidovorans                                 	|
Sphingobium hydrophobicum                                   	|
Sphingobium indicum B90A                                    	|
Sphingobium japonicum UT26S                                 	|
Allosphingosinicella indica                                 	| .(603). kynU> .(267). kynB> .(80).. <kynA .(1790) |
Sphingorhabdus lacus                                        	| .(2462) <kynB .(383). <kynU .(153). <kynA .(233). |
Tardibacter chloracetimidivorans                            	|
Zymomonas mobilis subsp. mobilis ZM4 = ATCC 31821           	|
Sphingosinicella microcystinivorans                         	| .(1264) <kynA <kynU .(672). <kynB .(1791) |
Anaplasma centrale str. Israel                              	|
Anaplasma marginale str. Florida                            	|
Anaplasma ovis str. Haibei                                  	|
Anaplasma phagocytophilum str. JM                           	|
Anaplasma platys                                            	|
Ehrlichia canis str. Jake                                   	|
Ehrlichia chaffeensis str. West Paces                       	|
Ehrlichia muris AS145                                       	|
Ehrlichia ruminantium                                       	|
Neorickettsia findlayensis                                  	|
Neorickettsia helminthoeca str. Oregon                      	|
Neorickettsia risticii str. Illinois                        	|
Neorickettsia sennetsu str. Miyayama                        	|
Wolbachia pipientis                                         	|
Orientia tsutsugamushi                                      	|
Rickettsia akari str. Hartford                              	|
Rickettsia asiatica                                         	|
Rickettsia australis str. Cutlack                           	|
Rickettsia conorii str. Malish 7                            	|
Rickettsia helvetica C9P9                                   	|
Rickettsia heilongjiangensis                                	|
Rickettsia japonica                                         	|
Rickettsia monacensis                                       	|
Rickettsia raoultii                                         	|
Rickettsia rickettsii str. Iowa                             	|
Rickettsia sibirica 246                                     	|
Rickettsia slovaca 13-B                                     	|
Rickettsia bellii RML369-C                                  	|
Rickettsia canadensis str. CA410                            	|
Rickettsia prowazekii str. Chernikova                       	|
Rickettsia typhi str. TH1527                                	|
Asticcacaulis excentricus CB 48                             	|
Brevundimonas subvibrioides ATCC 15264                      	| .(739). kynA> .(2566) |
Brevundimonas vancanneytii                                  	| .(260). kynA> .(2804) |
Brevundimonas vesicularis                                   	| .(1854) kynA> .(1487) |
Caulobacter flavus                                          	|
Caulobacter mirabilis                                       	| .(1464) kynA> kynU> .(2819) |
Caulobacter rhizosphaerae                                   	| .(2142) <kynA .(2983) |
Caulobacter segnis                                          	|
Caulobacter vibrioides NA1000                               	| .(2987) <kynA .(898). |
Phenylobacterium zucineum HLK1                              	| .(650). <kynU <kynA .(1485) <kynB .(1740) |
Terricaulis silvestris                                      	| .(1391) <kynA .(1813) kynB> .(670). |
Celeribacter ethanolicus                                    	|
Celeribacter indicus                                        	|
Celeribacter marinus                                        	|
Celeribacter manganoxidans                                  	|
Defluviimonas alba                                          	| .(3194) kynB> .(1008) |
Dinoroseobacter shibae DFL 12 = DSM 16493                   	| .(1224) <kynU .(2371) |
Epibacterium mobile F1926                                   	| .(533). kynA> kynU> .(2497) |
Haematobacter massiliensis                                  	| .(149). <kynU .(2222) | .(28).. kynB> .(314). |
Halocynthiibacter arcticus                                  	|
Ketogulonicigenium robustum                                 	|
Ketogulonicigenium vulgare                                  	|
Leisingera aquaemixtae                                      	| .(813). <kynA <kynU .(2788) |
Leisingera methylohalidivorans DSM 14336                    	| .(2264) kynU> kynA> .(1558) |
Marinovum algicola DG 898                                   	| .(148). <kynB .(29).. | .(160). <kynU <kynA .(16).. |
Octadecabacter antarcticus 307                              	| .(635). kynU> .(2775) kynA> .(913). |
Octadecabacter arcticus 238                                 	| .(2280) kynB> .(995). <kynU .(1309) |
Octadecabacter temperatus                                   	| .(2612) <kynU .(592). |
Paracoccus aminophilus JCM 7686                             	| .(45).. kynU> .(322). | .(2505) kynB> .(961). | .(265). <kynA |
Paracoccus aminovorans                                      	|
Paracoccus contaminans                                      	| .(795). kynU> .(1909) |
Paracoccus denitrificans                                    	| .(356). kynR> .(936). <kynU .(1490) |
Paracoccus jeotgali                                         	| .(2093) kynU> .(613). kynB> .(175). |
Paracoccus kondratievae                                     	| .(1925) <kynU .(247). | .(473). <kynB .(964). | .(61).. <kynR .(351). |
Paracoccus liaowanqingii                                    	| .(237). kynU> .(2486) kynA> .(304). |
Paracoccus mutanolyticus                                    	| .(1766) <kynB .(740). |
Paracoccus yeei                                             	| .(1926) <kynB .(1512) |
Paracoccus zhejiangensis                                    	| .(1449) kynR> .(655). kynA> .(7)... <kynU .(719). kynB> .(953). |
Parasedimentitalea marina                                   	| .(2399) <kynB .(478). kynU> .(1)... kynA> .(1236) |
Paraoceanicella profunda                                    	| .(920). <kynB .(4)... <kynB .(24).. <kynU .(1)... <kynA .(1567) kynR> .(978). |
Pelagibaca abyssi                                           	| .(3983) kynU> .(77).. |
Phaeobacter gallaeciensis                                   	| .(2245) kynU> kynA> .(1199) |
Phaeobacter inhibens                                        	| .(911). <kynA <kynU .(2513) |
Phaeobacter porticola                                       	| .(43).. <kynU .(76).. | .(908). <kynA .(2443) |
Planktomarina temperata RCA23                               	| .(2901) kynU> .(200). |
Polymorphum gilvum SL003B-26A1                              	| .(750). kynU> kynA> .(1)... kynB> .(3566) |
Profundibacter amoris                                       	| .(92).. <kynB .(1002) <kynU .(2387) |
Pseudohalocynthiibacter aestuariivivens                     	| .(2740) <kynB .(909). |
Pseudooceanicola algae                                      	|
Pseudopuniceibacterium antarcticum                          	| .(1607) kynR> .(2375) |
Rhodobaca barguzinensis                                     	| .(1818) <kynU .(1799) |
Rhodobacter blasticus                                       	| .(2592) kynB> .(222). <kynA <kynU .(580). |
Rhodobacter capsulatus                                      	| .(729). <kynU .(2596) |
Rhodobacter sphaeroides ATCC 17029                          	| .(1877) kynU> .(1106) | .(34).. <kynB .(1029) |
Rhodovulum sulfidophilum                                    	| .(2062) <kynB .(945). kynU> .(748). |
Roseicitreum antarcticum                                    	| .(2199) kynU> .(1009) |
Roseobacter denitrificans                                   	| .(1397) <kynU .(124). kynB> .(2281) |
Roseobacter litoralis Och 149                               	| .(632). <kynB .(164). kynU> .(3359) |
Roseobacter ponti                                           	| .(1451) kynA> kynU> .(1436) kynB> .(771). |
Roseibacterium elongatum DSM 19469                          	| .(1805) <kynU .(391). kynB> .(980). |
Roseovarius indicus                                         	| .(3230) kynA> kynU> .(1622) <kynB .(342). |
Ruegeria pomeroyi DSS-3                                     	| .(750). <kynB .(2075) kynU> .(1066) | .(418). kynA> .(35).. |
Silicimonas algicola                                        	| .(1320) <kynU .(2916) |
Sulfitobacter pseudonitzschiae                              	| .(2281) <kynU <kynA .(1110) kynB> .(363). |
Tabrizicola piscis                                          	| .(3662) kynB> .(297). <kynU .(6)... |
Thalassobius gelatinovorus                                  	| .(342). <kynU <kynA .(923). kynB> .(2423) |
Thioclava nitratireducens                                   	| .(1827) kynU> .(1840) |
Paremcibacter congregatus                                   	| .(1928) kynA> .(2)... kynU> .(1330) <kynB .(351). |
Glycocaulis alkaliphilus                                    	| .(278). kynB> kynU> .(966). <kynA .(1568) |
Hirschia baltica ATCC 49814                                 	|
Hyphomonas neptunium ATCC 15444                             	| .(2522) kynU> .(951). |
Kordiimonas pumila                                          	| .(1028) kynU> kynA> .(570). kynB> .(1934) |
Magnetococcus marinus MC-1                                  	|
Micavibrio aeruginosavorus ARL-13                           	| .(1383) kynA> kynU> .(914). |
Phreatobacter cathodiphilus                                 	|
Phreatobacter stygius                                       	| .(4041) <kynA .(1471) kynB> .(1314) |
Parvularcula bermudensis HTCC2503                           	|
Achromobacter denitrificans                                 	| .(2902) <kynR kynU> .(3179) kynB> kynA> .(65).. |
Achromobacter insolitus                                     	| .(1564) <kynA <kynB .(3025) <aa perm. <kynU kynR> .(1261) |
Achromobacter spanius                                       	| .(988). <aa perm. <kynU kynR> .(2780) <kynA <kynB .(1877) |
Achromobacter xylosoxidans                                  	| .(1568) <kynA <kynB .(3460) <aa perm. <kynU kynR> .(621). |
Advenella kashmirensis WT001                                	| .(1897) kynB> kynU> kynA> .(1324) |
Advenella mimigardefordensis DPN7                           	| .(1838) <kynA <kynU <kynB .(2402) |
Alcaligenes aquatilis                                       	| .(2454) <aa perm. <kynU kynR> .(378). <kynA .(566). |
Alcaligenes faecalis                                        	| .(1606) <aa perm. <kynU kynR> .(719). <kynA .(1419) |
Algicoccus marinus                                          	|
Basilea psittacipulmonis DSM 24701                          	|
Bordetella avium 197N                                       	| .(201). <kynR kynU> .(2114) kynB> kynA> .(989). |
Bordetella bronchialis                                      	|
Bordetella bronchiseptica                                   	| .(2823) kynB> kynA> .(1676) <kynU kynR> .(265). |
Bordetella flabilis                                         	|
Bordetella hinzii                                           	| .(272). kynB> kynA> .(985). <kynU kynR> .(3120) |
Bordetella holmesii                                         	| .(2122) kynB> kynA> .(942). <kynU kynR> .(167). |
Bordetella parapertussis                                    	| .(397). <kynU kynR> .(2655) kynA> .(1107) |
Bordetella pertussis 18323                                  	| .(214). <kynR kynU> .(2150) kynB> kynA> .(1128) |
Bordetella petrii                                           	| .(548). <kynU .(1283) <kynA <kynB .(3075) |
Bordetella pseudohinzii                                     	| .(2953) kynB> kynA> .(879). <kynU kynR> .(265). |
Castellaniella defragrans 65Phen                            	| .(3277) <aa perm. kynR> .(216). |
Kerstersia gyiorum                                          	| .(353). kynR> .(2972) |
Orrella dioscoreae                                          	|
Pigmentiphaga aceris                                        	| .(3627) kynB> kynU> kynA> .(1591) |
Taylorella asinigenitalis MCE3                              	|
Taylorella equigenitalis                                    	|
Acidovorax carolinensis                                     	| .(2673) kynB> kynU> kynA> .(935). |
Acidovorax citrulli AAC00-1                                 	| .(3600) kynB> kynU> kynA> .(1132) |
Acidovorax monticola                                        	| .(207). kynB> kynU> kynA> .(3541) |
Alicycliphilus denitrificans                                	| .(1372) <kynB .(2906) |
Comamonas kerstersii                                        	| .(1875) <kynB .(1)... <aa perm. .(1313) |
Comamonas koreensis                                         	| .(1718) <kynA .(8)... <kynU <kynB <aa perm. .(2893) |
Comamonas piscis                                            	| .(1671) <kynA .(6)... <kynU <kynB <aa perm. .(2895) |
Comamonas serinivorans                                      	|
Delftia lacustris                                           	| .(3561) <kynA .(2)... <kynU <kynB <aa perm. .(3)... <kynA .(1)... <aa perm. .(2653) |
Delftia tsuruhatensis                                       	| .(3812) <kynA .(2)... <kynU <kynB <aa perm. .(3)... <kynA .(1)... <aa perm. .(2668) |
Diaphorobacter aerolatus                                    	| .(1018) kynB> .(2604) |
Diaphorobacter polyhydroxybutyrativorans                    	| .(3369) kynB> kynU> kynA> .(300). |
Diaphorobacter ruginosibacter                               	| .(78).. kynU> .(839). <aa perm. .(2367) kynB> .(2)... kynA> .(1195) |
Hydrogenophaga crassostreae                                 	| .(1423) <kynA <kynU <kynB .(3070) |
Hydrogenophaga pseudoflava                                  	| .(3184) kynB> kynU> kynA> .(1391) |
Ottowia oryzae                                              	| .(989). <kynA .(1)... <kynU <kynB .(2468) |
Polaromonas naphthalenivorans CJ2                           	| .(1104) <kynA <kynU <kynB .(3001) |
Polaromonas vacuolata                                       	|
Pulveribacter suum                                          	| .(1770) kynB> kynU> kynA> .(1241) |
Ramlibacter tataouinensis TTB310                            	| .(1045) <kynA <kynU <kynB .(2860) |
Rhodoferax antarcticus                                      	|
Rhodoferax ferrireducens T118                               	|
Rhodoferax koreense                                         	| .(1902) <aa perm. .(2010) <kynU .(310). <kynA .(1057) |
Rhodoferax saidenbachensis                                  	| .(2577) kynU> kynA> .(1391) |
Rhodoferax sediminis                                        	| .(775). <kynA <kynU <kynB .(3305) |
Schlegelella thermodepolymerans                             	| .(2375) kynB> kynU> kynA> .(1189) |
Serpentinomonas mccroryi                                    	|
Serpentinomonas raichei                                     	|
Simplicispira suum                                          	| .(2820) <kynA <kynU <kynB .(656). |
Variovorax paradoxus S110                                   	| .(3841) kynB> kynU> kynA> .(1429) |
Verminephrobacter eiseniae EF01-2                           	| .(2778) <aa perm. .(2127) |
Aquabacterium olei                                          	|
Inhella inkyongensis                                        	| .(2106) kynB> kynU> kynA> .(1627) |
Leptothrix cholodnii SP-6                                   	|
Methylibium petroleiphilum PM1                              	|
Rhizobacter gummiphilus                                     	|
Roseateles depolymerans                                     	| .(1155) <kynA <kynU <kynB .(3662) |
Sphaerotilus natans subsp. sulfidivorans                    	| .(673). <kynA <kynU <kynB .(2804) |
Thiomonas arsenitoxydans                                    	| .(2313) kynB> kynU> kynA> .(1129) |
Thiomonas intermedia                                        	| .(432). kynB> kynU> kynA> .(2532) |
Xylophilus rhododendri                                      	| .(4281) kynR> .(945). |
Burkholderia cepacia                                        	| .(2636) <aa perm. .(56).. <kynR kynB> kynU> kynA> .(619). |
Burkholderia cenocepacia                                    	| .(2601) <aa perm. .(58).. <kynR kynB> kynU> kynA> .(600). |
Burkholderia dolosa AU0158                                  	| .(2528) <aa perm. .(57).. <kynR kynB> kynU> kynA> .(498). |
Burkholderia metallica                                      	| .(2548) <aa perm. .(56).. <kynR kynB> kynU> kynA> .(568). |
Burkholderia multivorans ATCC BAA-247                       	| .(2592) <aa perm. .(57).. <kynR kynB> kynU> kynA> .(483). |
Burkholderia pyrrocinia                                     	| .(494). <aa perm. .(60).. <kynR kynB> kynU> kynA> .(2632) |
Burkholderia seminalis                                      	| .(2486) <aa perm. .(58).. <kynR kynB> kynU> kynA> .(581). |
Burkholderia stagnalis                                      	| .(2229) <aa perm. .(489). <kynR kynB> kynU> kynA> .(588). |
Burkholderia stabilis                                       	| .(415). <aa perm. .(656). | .(2992) <kynA <kynU <kynB kynR> .(410). |
Burkholderia ubonensis                                      	| .(2897) <aa perm. .(62).. <kynR kynB> kynU> kynA> .(636). |
Burkholderia glumae                                         	| .(1647) <kynA <kynU <kynB kynR> .(1438) |
Burkholderia mallei                                         	| .(224). <kynA <kynU <kynB kynR> .(2759) |
Burkholderia oklahomensis C6786                             	| .(257). <aa perm. .(2213) | .(771). kynR> <kynB <kynU <kynA .(2831) |
Burkholderia pseudomallei                                   	| .(209). <aa perm. .(2232) | .(2702) <kynA <kynU <kynB kynR> .(719). |
Burkholderia thailandensis E264                             	| .(88).. <aa perm. .(2276) | .(2565) kynR> <kynB <kynU <kynA .(705). |
Burkholderia plantarii                                      	| .(542). <kynA <kynU <kynB kynR> .(3020) |
Caballeronia insecticola                                    	| .(598). <kynA <kynU <kynB kynR> .(2129) |
Chitinimonas arctica                                        	| .(4188) kynA> .(2)... kynU> .(430). |
Cupriavidus basilensis                                      	| .(1024) <aa perm. .(1934) <kynR kynB> kynU> kynA> .(1072) |
Cupriavidus gilardii                                        	| .(595). <kynA <kynU <kynB kynR> .(2438) |
Cupriavidus malaysiensis                                    	| .(641). <kynA <kynU <kynB kynR> .(3284) |
Cupriavidus nantongensis                                    	| .(740). <kynA <kynU <kynB kynR> .(3531) |
Cupriavidus neocaledonicus                                  	| .(400). <aa perm. .(2017) | .(2346) kynR> <kynB <kynU <kynA .(907). |
Cupriavidus necator H16                                     	| .(913). <kynA <kynU <kynB kynR> .(2718) |
Cupriavidus oxalaticus                                      	| .(953). <kynA <kynU <kynB kynR> .(2447) |
Cupriavidus taiwanensis LMG 19424                           	| .(893). <kynA <kynU <kynB kynR> .(2204) |
Ephemeroptericola cinctiostellae                            	|
Lautropia mirabilis                                         	|
Mycoavidus cysteinexigens                                   	|
Mycetohabitans rhizoxinica HKI 454                          	| .(678). kynR> .(1567) |
Pandoraea apista                                            	| .(1428) <kynA <kynU <kynB .(27).. <aa perm. .(1)... kynR> .(1185) <aa perm. .(2171) |
Pandoraea faecigallinarum                                   	| .(2264) <aa perm. .(919). <kynR .(1)... aa perm.> .(26).. kynB> kynU> kynA> .(1265) |
Pandoraea fibrosis                                          	| .(1978) <kynA <kynU <kynB .(26).. <aa perm. .(1)... kynR> .(913). aa perm.> .(1884) |
Pandoraea norimbergensis                                    	| .(1198) <kynA <kynU <kynB .(27).. <aa perm. .(1)... kynR> .(946). aa perm.> .(3118) |
Pandoraea oxalativorans                                     	| .(2455) <aa perm. .(953). <kynR .(1)... aa perm.> .(25).. kynB> kynU> kynA> .(1290) |
Pandoraea pnomenusa                                         	| .(2478) <aa perm. .(841). <kynR .(27).. kynB> kynU> kynA> .(1339) |
Pandoraea pulmonicola                                       	| .(1336) <kynA <kynU <kynB .(26).. <aa perm. .(1)... kynR> .(3563) |
Pandoraea sputorum                                          	| .(1118) <kynA <kynU <kynB .(25).. <aa perm. .(1)... kynR> .(1041) aa perm.> .(2747) |
Pandoraea thiooxydans                                       	| .(706). <kynA <kynU <kynB kynR> .(190). <aa perm. .(3149) |
Pandoraea vervacti                                          	| .(2493) <aa perm. .(914). <kynR .(1)... aa perm.> .(25).. kynB> kynU> kynA> .(1279) |
Paraburkholderia aromaticivorans                            	| .(1678) <kynA <kynU <kynB kynR> .(2147) |
Paraburkholderia caffeinilytica                             	| .(2740) <kynA <kynU <kynB kynR> .(1198) |
Paraburkholderia caribensis                                 	| .(1934) <kynA <kynU <kynB kynR> .(1199) |
Paraburkholderia dokdonella                                 	| .(126). <aa perm. .(972). | .(462). <kynA <kynU <kynB kynR> .(2266) |
Paraburkholderia graminis                                   	| .(697). <kynA <kynU <kynB kynR> .(2791) | .(260). kynA> .(173). |
Paraburkholderia phymatum STM815                            	| .(500). <kynA <kynU <kynB kynR> .(2542) |
Paraburkholderia phytofirmans PsJN                          	| .(740). <kynA <kynU <kynB kynR> .(1712) <aa perm. .(1429) |
Paraburkholderia sprentiae WSM5005                          	| .(3135) <kynA <kynU <kynB kynR> .(48).. |
Paraburkholderia terricola                                  	| .(705). <kynA <kynU <kynB kynR> .(2819) |
Paraburkholderia terrae                                     	| .(502). <kynA <kynU <kynB kynR> .(2619) |
Paraburkholderia tropica                                    	| .(486). <kynA <kynU <kynB kynR> .(1682) <aa perm. .(687). |
Paraburkholderia xenovorans LB400                           	| .(748). <kynA <kynU <kynB kynR> .(3551) |
Paraburkholderia acidophila                                 	| .(2654) <aa perm. .(62).. <kynR kynB> kynU> kynA> .(657). |
Polynucleobacter acidiphobus                                	|
Polynucleobacter asymbioticus QLW-P1DMWA-1                  	|
Polynucleobacter difficilis                                 	|
Polynucleobacter duraquae                                   	|
Polynucleobacter necessarius                                	|
Polynucleobacter paneuropaeus                               	|
Polynucleobacter wuianus                                    	|
Ralstonia insidiosa                                         	| .(643). <aa perm. .(1026) | .(760). <kynA <kynU <kynB kynR> .(2932) |
Ralstonia mannitolilytica                                   	| .(439). <aa perm. .(856). | .(2494) <kynA <kynU <kynB kynR> .(674). |
Ralstonia pseudosolanacearum                                	| .(750). <kynA <kynU <kynB kynR> .(2615) |
Ralstonia solanacearum                                      	| .(2565) <kynA <kynU <kynB kynR> .(542). |
Collimonas arenae                                           	| .(934). <kynA <kynU <kynB kynR> .(3163) |
Collimonas fungivorans                                      	| .(1115) <kynA <kynU <kynB kynR> .(3700) |
Collimonas pratensis                                        	| .(1164) <kynA <kynU <kynB kynR> .(3790) |
Herminiimonas arsenitoxidans                                	|
Herbaspirillum frisingense                                  	|
Herbaspirillum huttiense                                    	|
Herbaspirillum robiniae                                     	| .(4783) kynU> .(3)... <aa perm. .(48).. |
Herbaspirillum rubrisubalbicans M1                          	|
Herbaspirillum seropedicae                                  	|
Janthinobacterium agaricidamnosum NBRC 102515 = DSM 9628    	| .(1385) <kynA <kynU .(3669) |
Janthinobacterium lividum                                   	| .(2071) <kynA <kynU .(3460) |
Janthinobacterium svalbardensis                             	| .(2133) <kynA <kynU .(3312) |
Massilia albidiflava                                        	| .(2708) <kynB .(1918) kynU> kynA> .(1418) |
Massilia armeniaca                                          	| .(3749) kynU> kynA> .(1489) |
Massilia flava                                              	| .(4581) <kynA <kynU .(526). <kynB .(748). |
Massilia lutea                                              	| .(1898) kynU> kynA> .(3919) <kynB .(277). |
Massilia oculi                                              	| .(4228) <kynA <kynU .(763). |
Massilia putida                                             	| .(1561) kynU> kynA> .(4394) |
Massilia umbonata                                           	| .(3821) <kynA <kynU .(679). <kynB .(1577) |
Massilia violaceinigra                                      	| .(3573) <kynA <kynU .(2684) |
Oxalobacter formigenes                                      	|
Undibacterium parvum                                        	| .(2580) <kynA <kynU <kynB kynR> .(1600) |
Sutterella faecalis                                         	| .(1253) <kynB .(1138) |
Sutterella megalosphaeroides                                	| .(2008) <kynB .(58).. |
Alysiella filiformis                                        	|
Chitinolyticbacter meiyuanensis                             	|
Conchiformibius steedae                                     	|
Crenobacter cavernae                                        	|
Eikenella corrodens                                         	|
Eikenella exigua                                            	|
Kingella oralis                                             	|
Neisseria animaloris                                        	|
Neisseria animalis                                          	|
Neisseria bacilliformis                                     	|
Neisseria brasiliensis                                      	|
Neisseria canis                                             	|
Neisseria chenwenguii                                       	|
Neisseria cinerea                                           	|
Neisseria elongata                                          	|
Neisseria flavescens                                        	|
Neisseria gonorrhoeae                                       	|
Neisseria lactamica                                         	|
Neisseria meningitidis                                      	|
Neisseria musculi                                           	|
Neisseria polysaccharea                                     	|
Neisseria shayeganii                                        	|
Neisseria subflava                                          	|
Neisseria wadsworthii                                       	|
Neisseria weaveri                                           	|
Neisseria zalophi                                           	|
Neisseria zoodegmatis                                       	|
Simonsiella muelleri ATCC 29453                             	|
Snodgrassella alvi wkB2                                     	| .(683). <aa perm. <kynU .(1523) |
Vitreoscilla filiformis                                     	|
Aquitalea denitrificans                                     	|
Chitinibacter fontanus                                      	|
Chromobacterium haemolyticum                                	| .(502). <aa perm. <kynU <kynB kynR> .(3871) kynA> .(411). |
Chromobacterium paludis                                     	|
Chromobacterium phragmitis                                  	|
Chromobacterium vaccinii                                    	|
Chromobacterium violaceum ATCC 12472                        	|
Aromatoleum aromaticum EbN1                                 	|
Oryzomicrobium terrae                                       	|
Azoarcus olearius                                           	| .(2068) kynB> .(1965) |
Azoarcus pumilus                                            	|
Thauera aromatica K172                                      	|
Thauera chlorobenzoica                                      	|
Thauera humireducens                                        	|
Thauera hydrothermalis                                      	|
Dechloromonas aromatica RCB                                 	|
Casimicrobium huifangae                                     	| .(3396) <kynA <kynU .(556). |
Denitratisoma oestradiolicum                                	|
Sulfuritalea hydrogenivorans sk43H                          	|
Ferriphaselus amnicola                                      	|
Gallionella capsiferriformans ES-2                          	|
Sideroxydans lithotrophicus ES-1                            	|
Methylobacillus flagellatus KT                              	|
Methylovorus glucosetrophus SIP3-4                          	|
Methylophilus medardicus                                    	|
Methylotenera mobilis JLW8                                  	|
Methylotenera versatilis 301                                	|
Nitrosospira briensis C-128                                 	|
Nitrosospira lacus                                          	|
Nitrosospira multiformis ATCC 25196                         	|
Nitrosomonas europaea ATCC 19718                            	|
Nitrosomonas stercoris                                      	|
Nitrosomonas ureae                                          	|
Sulfuritortus calidifontis                                  	|
Sulfuricella denitrificans skB26                            	|
Sulfurimicrobium lacus                                      	|
Sulfuriferula nivalis                                       	|
Sulfuriferula plumbiphila                                   	|
Acidithiobacillus caldus                                    	|
Acidithiobacillus ferridurans                               	|
Acidithiobacillus ferrooxidans ATCC 23270                   	|
Acidithiobacillus ferrivorans SS3                           	|
Acidithiobacillus thiooxidans ATCC 19377                    	|
Aliarcobacter faecis                                        	|
Arcobacter butzleri ED-1                                    	|
Aliarcobacter cibarius                                      	|
Arcobacter cryaerophilus ATCC 43158                         	|
Aliarcobacter lanthieri                                     	|
[Arcobacter] porcinus                                       	|
Arcobacter skirrowii CCUG 10374                             	|
Arcobacter trophiarum LMG 25534                             	|
Arcobacter anaerophilus                                     	|
Arcobacter aquimarinus                                      	|
Arcobacter cloacae                                          	|
Arcobacter defluvii                                         	|
Arcobacter ellisii                                          	|
Arcobacter nitrofigilis DSM 7299                            	|
Arcobacter peruensis                                        	|
Arcobacter suis CECT 7833                                   	|
Arcobacter venerupis                                        	|
Halarcobacter bivalviorum                                   	|
Halarcobacter ebronensis                                    	|
Malaciobacter canalis                                       	|
Malaciobacter halophilus                                    	|
Malaciobacter marinus                                       	|
Malaciobacter molluscorum LMG 25693                         	|
Malaciobacter mytili LMG 24559                              	|
Malaciobacter pacificus                                     	|
Poseidonibacter lekithochrous                               	|
Poseidonibacter parvus                                      	|
Pseudoarcobacter acticola                                   	|
Campylobacter armoricus                                     	|
Campylobacter avium LMG 24591                               	|
Campylobacter blaseri                                       	|
Campylobacter canadensis                                    	|
Campylobacter corcagiensis                                  	|
Campylobacter concisus                                      	|
Campylobacter cuniculorum DSM 23162 = LMG 24588             	|
Campylobacter curvus                                        	|
Campylobacter fetus                                         	|
Campylobacter geochelonis                                   	|
Campylobacter gracilis                                      	|
Campylobacter hepaticus                                     	|
Campylobacter helveticus                                    	|
Campylobacter hyointestinalis subsp. lawsonii               	|
Campylobacter iguaniorum                                    	|
Campylobacter insulaenigrae NCTC 12927                      	|
Campylobacter jejuni subsp. jejuni NCTC 11168 = ATCC 700819 	|
Campylobacter lanienae NCTC 13004                           	|
Campylobacter lari RM2100                                   	|
Campylobacter mucosalis                                     	|
Campylobacter ornithocola                                   	|
Campylobacter peloridis                                     	|
Campylobacter pinnipediorum subsp. pinnipediorum            	|
Campylobacter rectus                                        	|
Campylobacter showae                                        	|
Campylobacter sputorum bv. paraureolyticus LMG 11764        	|
Campylobacter subantarcticus LMG 24377                      	|
Campylobacter upsaliensis RM3940                            	|
Campylobacter volucris LMG 24379                            	|
Sulfurospirillum barnesii SES-3                             	|
Sulfurospirillum cavolei                                    	|
Sulfurospirillum deleyianum DSM 6946                        	|
Sulfurospirillum halorespirans DSM 13726                    	|
Sulfurospirillum multivorans DSM 12446                      	|
Helicobacter acinonychis str. Sheeba                        	|
Helicobacter apodemus                                       	|
Helicobacter bizzozeronii CIII-1                            	|
Helicobacter canadensis                                     	|
Helicobacter cetorum MIT 99-5656                            	|
Helicobacter cinaedi                                        	|
Helicobacter felis ATCC 49179                               	|
Helicobacter hepaticus ATCC 51449                           	|
Helicobacter himalayensis                                   	|
Helicobacter mustelae                                       	|
Helicobacter pylori Puno135                                 	|
Helicobacter pylori 26695-1CL                               	|
Helicobacter pylori 26695-1CH                               	|
Helicobacter typhlonius                                     	|
Helicobacter winghamensis                                   	|
Wolinella succinogenes DSM 1740                             	|
Nitratifractor salsuginis DSM 16511                         	|
Sulfurovum lithotrophicum                                   	|
Sulfurimonas autotrophica DSM 16294                         	|
Sulfurimonas denitrificans DSM 1251                         	|
Sulfurimonas gotlandica GD1                                 	|
Sulfurimonas paralvinellae                                  	|
Sulfuricurvum kujiense DSM 16994                            	| .(2379) kynB> .(148). |
Caminibacter mediatlanticus TB-2                            	|
Cetia pacifica                                              	|
Nautilia profundicola AmH                                   	|
Nitratiruptor labii                                         	|
Anaeromyxobacter dehalogenans 2CP-1                         	| .(3310) <kynA .(1149) |
Corallococcus coralloides DSM 2259                          	| .(859). <kynU .(885). kynR> .(6098) <kynA .(107). |
Corallococcus macrosporus DSM 14697                         	| .(115). kynA> .(6015) kynU> .(691). kynR> .(220). |
Myxococcus hansupus                                         	| .(5584) kynU> .(994). kynA> .(575). |
Myxococcus stipitatus DSM 14675                             	| .(916). <kynU .(4995) <kynB .(1912) <kynA .(80).. |
Myxococcus xanthus DK 1622                                  	| .(879). <kynU .(6222) <kynA .(90).. |
Melittangium boletus DSM 14713                              	| .(4066) <kynU .(485). <kynB .(350). kynA> .(3056) |
Vulgatibacter incomptus                                     	| .(786). <kynR .(347). <kynA .(527). kynU> .(1922) |
Chondromyces crocatus                                       	| .(353). <kynA .(7710) |
Pajaroellobacter abortibovis                                	|
Sorangium cellulosum So ce56                                	| .(955). <kynA .(6533) <kynU .(381). kynB> .(1545) |
Labilithrix luteola                                         	| .(5220) kynA> kynU> .(1043) <kynB .(2323) <kynA .(1867) |
Minicystis rosea                                            	| .(1799) kynU> .(6138) <kynA .(4557) |
Sandaracinus amylolyticus                                   	| .(617). <kynA .(1205) <kynU .(6816) |
Haliangium ochraceum DSM 14365                              	| .(223). kynA> .(4969) <kynU .(1524) |
Bradymonas sediminis                                        	| .(2628) kynU> .(403). kynA> .(683). |
Persicimonas caeni                                          	| .(1624) kynU> .(1938) kynA> .(2318) |
Desulfurella acetivorans A63                                	|
Hippea maritima DSM 10411                                   	|
Desulfobacca acetoxidans DSM 11109                          	|
Desulfomonile tiedjei DSM 6799                              	| .(1983) kynB> .(1606) kynB> .(1684) |
Syntrophus aciditrophicus SB                                	|
Desulfoglaeba alkanexedens ALDC                             	|
Syntrophobacter fumaroxidans MPOB                           	| .(504). <kynB .(3467) |
Desulfocurvibacter africanus subsp. africanus str. Walvis ..	| .(2008) <kynB .(1740) |
Desulfovibrio alaskensis G20                                	|
Desulfovibrio carbinolicus                                  	| .(2046) <kynB .(1888) |
Desulfovibrio carbinoliphilus subsp. oakridgensis           	| .(776). kynB> .(2836) |
Desulfovibrio fairfieldensis                                	|
Desulfovibrio ferrophilus                                   	| .(756). <kynB .(2531) |
Desulfovibrio gigas DSM 1382 = ATCC 19364                   	|
Desulfovibrio marinus                                       	| .(3885) kynB> .(203). |
Desulfovibrio magneticus RS-1                               	| .(2081) <kynB .(2415) |
Desulfovibrio sulfodismutans DSM 3696                       	| .(3657) <kynB .(242). |
Desulfovibrio vulgaris RCH1                                 	|
Desulfovibrio hydrothermalis AM13 = DSM 14728               	| .(2586) kynB> .(612). |
Desulfovibrio salexigens DSM 2638                           	| .(2455) kynB> .(1350) |
Desulfolutivibrio sulfoxidireducens                         	| .(2326) <kynB .(1199) |
Lawsonia intracellularis N343                               	| .(446). <kynU .(805). |
Pseudodesulfovibrio aespoeensis Aspo-2                      	| .(324). kynB> .(2956) |
Pseudodesulfovibrio piezophilus C1TLV30                     	| .(2289) kynB> .(923). |
Pseudodesulfovibrio profundus                               	| .(2081) kynB> .(1759) |
Desulfomicrobium baculatum DSM 4028                         	|
Desulfomicrobium orale DSM 12838                            	|
Desulfohalobium retbaense DSM 5692                          	| .(1795) <kynB .(618). |
Desulfatibacillum aliphaticivorans                          	| .(2881) kynB> .(2362) |
Desulfosarcina alkanivorans                                 	| .(5068) kynB> .(1214) |
Desulfosarcina ovata subsp. sediminis                       	| .(3123) <kynB .(3943) |
Desulfosarcina widdelii                                     	|
Desulfobacterium autotrophicum HRM2                         	| .(605). kynB> .(4113) |
Desulfobacter hydrogenophilus                               	|
Desulfobacter postgatei 2ac9                                	|
Desulfococcus multivorans                                   	|
Desulfococcus oleovorans Hxd3                               	|
Desulfobacula toluolica Tol2                                	|
Desulfurivibrio alkaliphilus AHT 2                          	|
Desulfobulbus oralis                                        	|
Desulfobulbus propionicus DSM 2032                          	| .(2130) <kynB .(1200) |
Desulfotalea psychrophila LSv54                             	|
Desulfocapsa sulfexigens DSM 10523                          	|
Desulfarculus baarsii DSM 2075                              	| .(1489) kynB> .(1802) |
Desulfuromonas soudanensis                                  	|
Pelobacter propionicus DSM 2379                             	|
Geobacter bemidjiensis Bem                                  	| .(3474) kynB> .(506). |
Geobacter bremensis                                         	| .(3459) <kynB .(403). |
Geobacter daltonii FRC-32                                   	| .(1048) kynB> .(2696) |
Geobacter lovleyi SZ                                        	|
Geobacter metallireducens GS-15                             	| .(42).. kynB> .(3508) |
Geobacter pickeringii                                       	| .(34).. kynB> .(3205) |
Geobacter sulfurreducens PCA                                	| .(3274) kynB> .(116). |
Geobacter uraniireducens Rf4                                	| .(170). kynB> .(4286) |
Geoalkalibacter subterraneus                                	|
Syntrophotalea acetylenica                                  	| .(1272) <kynB .(1563) |
Pelobacter carbinolicus DSM 2380                            	|
Bacteriovorax stolpii                                       	| .(3600) kynA> .(224). |
Halobacteriovorax marinus SJ                                	| .(207). <kynA .(3060) |
Bdellovibrio bacteriovorus HD100                            	| .(1625) <kynA .(1916) |
Bdellovibrio exovorus JSS                                   	| .(1022) kynA> .(1572) |
Silvanigrella aquatica                                      	| .(1712) <kynA .(1088) |
Hydrogenophilus thermoluteolus                              	|
Mariprofundus aestuarium                                    	|
Mariprofundus ferrinatatus                                  	|
```
