# Supplementary material for: Evolutionarily stable gene clusters shed light on the common grounds of pathogenicity in the Acinetobacter calcoaceticus-baumannii complex
Source: PLoS Genet. 2022 Jun 2;18(6):e1010020. doi: 10.1371/journal.pgen.1010020 (PMC9162365; doi:10.1371/journal.pgen.1010020)
Supplement: S8 Table — (PDF) [file pgen.1010020.s023.pdf]

RefSeq Assem. Acc.	NCBI Species/Strain Name	*Corrected (short)*	Sampled/Isolated from
GCF_000367945.1	*A. sp*. NIPH 809	*prot*	ear, Czech Republic
GCF_000368145.1	*A. guillouiae* CIP 63.46	*guil*	not available
GCF_000368445.1	*A. sp.* CIP 56.2	*GS16*	patient liver abscess, France
GCF_000368485.1	*A. guillouiae* NIPH 991	*guil*	ear swab (outpatient), Czech Republic
GCF_000368825.1	*A. ursingii* DSM 16037	*ursi*	blood of patient, Czech Republic
GCF_000368845.1	*A. ursingii* ANC 3649	*ursi*	blood of patient, Turkey
GCF_000368925.1	*A. bereziniae* LMG 1003	*bere*	wound of patient
GCF_000369405.1	*A. sp.* ANC 3929	TX21	patient urine, Czech Republic
GCF_000369525.1	*A. sp.* CIP 70.18	GS16	patient urine
GCF_000369545.1	*A. sp.* NIPH 1867	*disp*	isolated from leg ulceration, France
GCF_000369565.1	*A. sp.* ANC 3862	*sp.*	wound of patient, Czech Republic
GCF_000369605.1	*A. courvalinii* NIPH 1847	*cour*	human eye conjunctiva, Brazil
GCF_000369625.1	*A. sp.* NIPH 2171	*vari*	patient urine, Sweden
GCF_000369645.1	*A. sp.* CIP 64.2	*sp.*	human eye conjunctiva of patient with conjunctivitis, Germany
GCF_000369705.1	*A. sp.* NIPH 2168	*vivi*	patient (clinical specimen), Netherlands
GCF_000369785.1	*A. courvalinii* NIPH 3623	*cour*	human eye conjunctiva, Czech Republic
GCF_000369805.1	*A. sp.* NIPH 2100	TX22	patient wound, France
GCF_000400715.1	*A. sp.* CIP 110321	15BJ	human skin, Hungary
GCF_000413855.1	*A. gyllenbergii* CIP 110306	*gyll*	patient urine, Netherlands
GCF_000413935.1	*A. sp.* NIPH 2036	*coll*	catheter of patient, Belgium
GCF_000488195.1	*A. gyllenbergii* NIPH 230	*gyll*	vagina, Czech Republic
GCF_000580655.1	*A. baumannii* 146457	*cour*	patient in hospital medical or surgical intensive care unit; perirectal swab, USA
GCF_000805455.1	*A. sp.* A47	*sp.*	patient soft tissue with soft tissue infection, Argentina
GCF_000816495.1	*A. harbinensis* HITLi 7	*harb*	river water, China
GCF_000949835.1	*A. ursingii* NBRC 110605	*ursi*	human abscess, Japan
GCF_001056675.1	*A. ursingii* 421_ABAU	*ursi*	patient in clinic, USA
GCF_001500155.1	*A. bereziniae* HPC229	*bere*	clinical patient blood, Argentina
GCF_001704615.1	*A. defluvii* WCHA30	*defl*	microbial sample of hospital sewage, China
GCF_001707755.1	*A. celticus* ANC 4603	*celt*	forest spring water with sludge, Czech Republic
GCF_001753605.1	*A. proteolyticus* ANC 3849	*prot*	leg wound of patient, Czech Republic
GCF_002835245.1	*A. proteolyticus* 2P01AA	*prot*	spacecraft associated surface, USA
