# Supplementary material for: Evolutionarily stable gene clusters shed light on the common grounds of pathogenicity in the Acinetobacter calcoaceticus-baumannii complex
Source: PLoS Genet. 2022 Jun 2;18(6):e1010020. doi: 10.1371/journal.pgen.1010020 (PMC9162365; doi:10.1371/journal.pgen.1010020)
Supplement: S10 Fig — (PDF) [file pgen.1010020.s011.pdf]

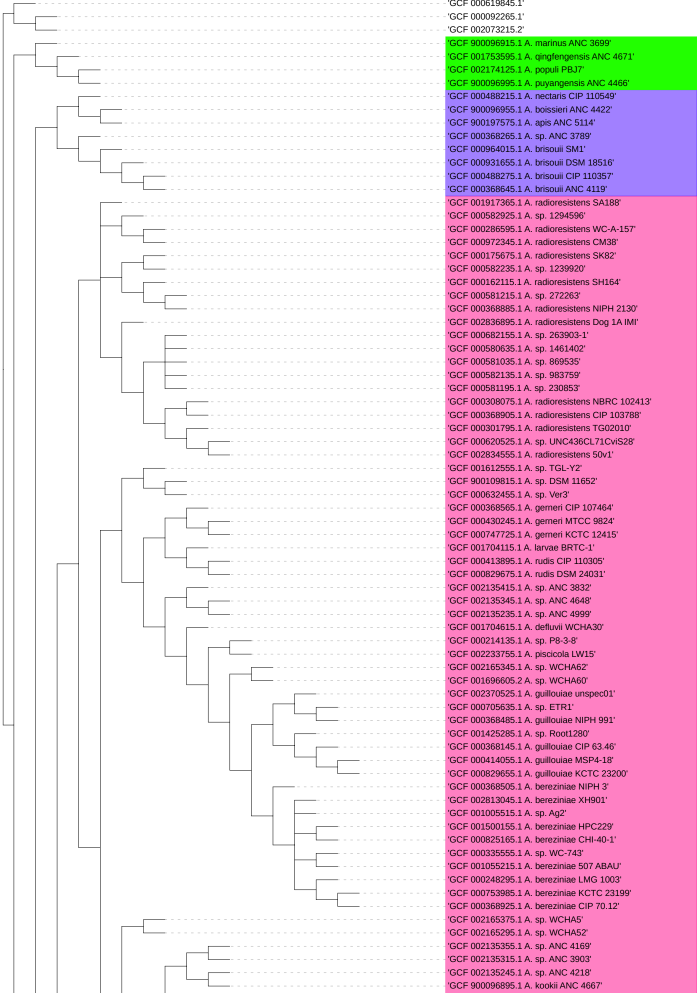

'GCF 000619845.1'
'GCF 000092265.1'
'GCF 002073215.2'
'GCF 900096915.1 A. marinus ANC 3699'
'GCF 001753595.1 A. qingfengensis ANC 4671'
'GCF 002174125.1 A. populi PBJ7'
'GCF 900096995.1 A. puyangensis ANC 4466'
'GCF 000488215.1 A. nectaris CIP 110549'
'GCF 900096955.1 A. boissieri ANC 4422'
'GCF 900197575.1 A. apis ANC 5114'
'GCF 000368265.1 A. sp. ANC 3789'
'GCF 000964015.1 A. brisouii SM1'
'GCF 000931655.1 A. brisouii DSM 18516'
'GCF 000488275.1 A. brisouii CIP 110357'
'GCF 000368645.1 A. brisouii ANC 4119'
'GCF 001917365.1 A. radioresistens SA188'
'GCF 000582925.1 A. sp. 1294596'
'GCF 000286595.1 A. radioresistens WC-A-157'
'GCF 000972345.1 A. radioresistens CM38'
'GCF 000175675.1 A. radioresistens SK82'
'GCF 000582235.1 A. sp. 1239920'
'GCF 000162115.1 A. radioresistens SH164'
'GCF 000581215.1 A. sp. 272263'
'GCF 000368885.1 A. radioresistens NIPH 2130'
'GCF 002836895.1 A. radioresistens Dog 1A IMI'
'GCF 000682155.1 A. sp. 263903-1'
'GCF 000580635.1 A. sp. 1461402'
'GCF 000581035.1 A. sp. 869535'
'GCF 000582135.1 A. sp. 983759'
'GCF 000581195.1 A. sp. 230853'
'GCF 000308075.1 A. radioresistens NBRC 102413'
'GCF 000368905.1 A. radioresistens CIP 103788'
'GCF 000301795.1 A. radioresistens TG02010'
'GCF 000620525.1 A. sp. UNC436CL71CviS28'
'GCF 002834555.1 A. radioresistens 50v1'
'GCF 001612555.1 A. sp. TGL-Y2'
'GCF 900109815.1 A. sp. DSM 11652'
'GCF 000632455.1 A. sp. Ver3'
'GCF 000368565.1 A. gerneri CIP 107464'
'GCF 000430245.1 A. gerneri MTCC 9824'
'GCF 000747725.1 A. gerneri KCTC 12415'
'GCF 001704115.1 A. larvae BRTC-1'
'GCF 000413895.1 A. rudis CIP 110305'
'GCF 000829675.1 A. rudis DSM 24031'
'GCF 002135415.1 A. sp. ANC 3832'
'GCF 002135345.1 A. sp. ANC 4648'
'GCF 002135235.1 A. sp. ANC 4999'
'GCF 001704615.1 A. defluvii WCHA30'
'GCF 000214135.1 A. sp. P8-3-8'
'GCF 002233755.1 A. piscicola LW15'
'GCF 002165345.1 A. sp. WCHA62'
'GCF 001696605.2 A. sp. WCHA60'
'GCF 002370525.1 A. guillouiae unspec01'
'GCF 000705635.1 A. sp. ETR1'
'GCF 000368485.1 A. guillouiae NIPH 991'
'GCF 001425285.1 A. sp. Root1280'
'GCF 000368145.1 A. guillouiae CIP 63.46'
'GCF 000414055.1 A. guillouiae MSP4-18'
'GCF 000829655.1 A. guillouiae KCTC 23200'
'GCF 000368505.1 A. bereziniae NIPH 3'
'GCF 002813045.1 A. bereziniae XH901'
'GCF 001005515.1 A. sp. Ag2'
'GCF 001500155.1 A. bereziniae HPC229'
'GCF 000825165.1 A. bereziniae CHI-40-1'
'GCF 000335555.1 A. sp. WC-743'
'GCF 001055215.1 A. bereziniae 507 ABAU'
'GCF 000248295.1 A. bereziniae LMG 1003'
'GCF 000753985.1 A. bereziniae KCTC 23199'
'GCF 000368925.1 A. bereziniae CIP 70.12'
'GCF 002165375.1 A. sp. WCHA5'
'GCF 002165295.1 A. sp. WCHA52'
'GCF 002135355.1 A. sp. ANC 4169'
'GCF 002135315.1 A. sp. ANC 3903'
'GCF 002135245.1 A. sp. ANC 4218'
'GCF 900096895.1 A. kookii ANC 4667'

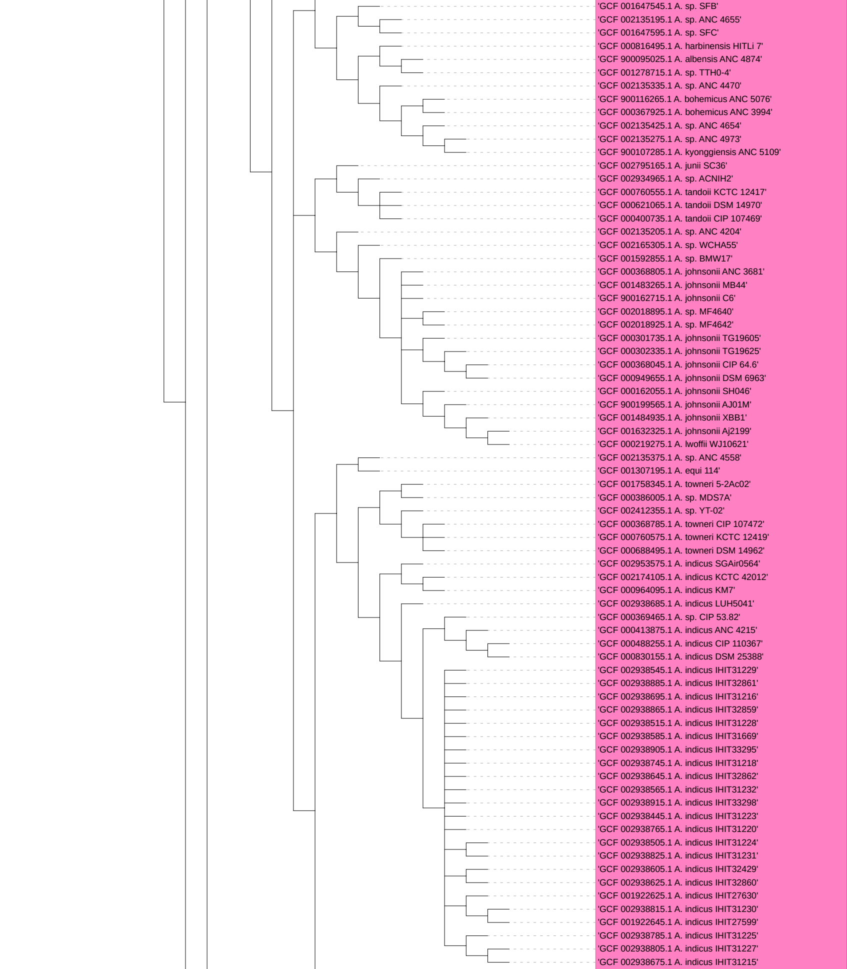
'GCF 001647545.1 A. sp. SFB'
'GCF 002135195.1 A. sp. ANC 4655'
'GCF 001647595.1 A. sp. SFC'
'GCF 000816495.1 A. harbinensis HITLi 7'
'GCF 900095025.1 A. albensis ANC 4874'
'GCF 001278715.1 A. sp. TTH0-4'
'GCF 002135335.1 A. sp. ANC 4470'
'GCF 900116265.1 A. bohemicus ANC 5076'
'GCF 000367925.1 A. bohemicus ANC 3994'
'GCF 002135425.1 A. sp. ANC 4654'
'GCF 002135275.1 A. sp. ANC 4973'
'GCF 900107285.1 A. kyonggiensis ANC 5109'
'GCF 002795165.1 A. junii SC36'
'GCF 002934965.1 A. sp. ACNIH2'
'GCF 000760555.1 A. tandoii KCTC 12417'
'GCF 000621065.1 A. tandoii DSM 14970'
'GCF 000400735.1 A. tandoii CIP 107469'
'GCF 002135205.1 A. sp. ANC 4204'
'GCF 002165305.1 A. sp. WCHA55'
'GCF 001592855.1 A. sp. BMW17'
'GCF 000368805.1 A. johnsonii ANC 3681'
'GCF 001483265.1 A. johnsonii MB44'
'GCF 900162715.1 A. johnsonii C6'
'GCF 002018895.1 A. sp. MF4640'
'GCF 002018925.1 A. sp. MF4642'
'GCF 000301735.1 A. johnsonii TG19605'
'GCF 000302335.1 A. johnsonii TG19625'
'GCF 000368045.1 A. johnsonii CIP 64.6'
'GCF 000949655.1 A. johnsonii DSM 6963'
'GCF 000162055.1 A. johnsonii SH046'
'GCF 900199565.1 A. johnsonii AJ01M'
'GCF 001484935.1 A. johnsonii XBB1'
'GCF 001632325.1 A. johnsonii Aj2199'
'GCF 000219275.1 A. lwoffii WJ10621'
'GCF 002135375.1 A. sp. ANC 4558'
'GCF 001307195.1 A. equi 114'
'GCF 001758345.1 A. towneri 5-2Ac02'
'GCF 000386005.1 A. sp. MDS7A'
'GCF 002412355.1 A. sp. YT-02'
'GCF 000368785.1 A. towneri CIP 107472'
'GCF 000760575.1 A. towneri KCTC 12419'
'GCF 000688495.1 A. towneri DSM 14962'
'GCF 002953575.1 A. indicus SGAir0564'
'GCF 002174105.1 A. indicus KCTC 42012'
'GCF 000964095.1 A. indicus KM7'
'GCF 002938685.1 A. indicus LUH5041'
'GCF 000369465.1 A. sp. CIP 53.82'
'GCF 000413875.1 A. indicus ANC 4215'
'GCF 000488255.1 A. indicus CIP 110367'
'GCF 000830155.1 A. indicus DSM 25388'
'GCF 002938545.1 A. indicus IHIT31229'
'GCF 002938885.1 A. indicus IHIT32861'
'GCF 002938695.1 A. indicus IHIT31216'
'GCF 002938865.1 A. indicus IHIT32859'
'GCF 002938515.1 A. indicus IHIT31228'
'GCF 002938585.1 A. indicus IHIT31669'
'GCF 002938905.1 A. indicus IHIT33295'
'GCF 002938745.1 A. indicus IHIT31218'
'GCF 002938645.1 A. indicus IHIT32862'
'GCF 002938565.1 A. indicus IHIT31232'
'GCF 002938915.1 A. indicus IHIT33298'
'GCF 002938445.1 A. indicus IHIT31223'
'GCF 002938765.1 A. indicus IHIT31220'
'GCF 002938505.1 A. indicus IHIT31224'
'GCF 002938825.1 A. indicus IHIT31231'
'GCF 002938605.1 A. indicus IHIT32429'
'GCF 002938625.1 A. indicus IHIT32860'
'GCF 001922625.1 A. indicus IHIT27630'
'GCF 002938815.1 A. indicus IHIT31230'
'GCF 001922645.1 A. indicus IHIT27599'
'GCF 002938785.1 A. indicus IHIT31225'
'GCF 002938805.1 A. indicus IHIT31227'
'GCF 002938675.1 A. indicus IHIT31215'

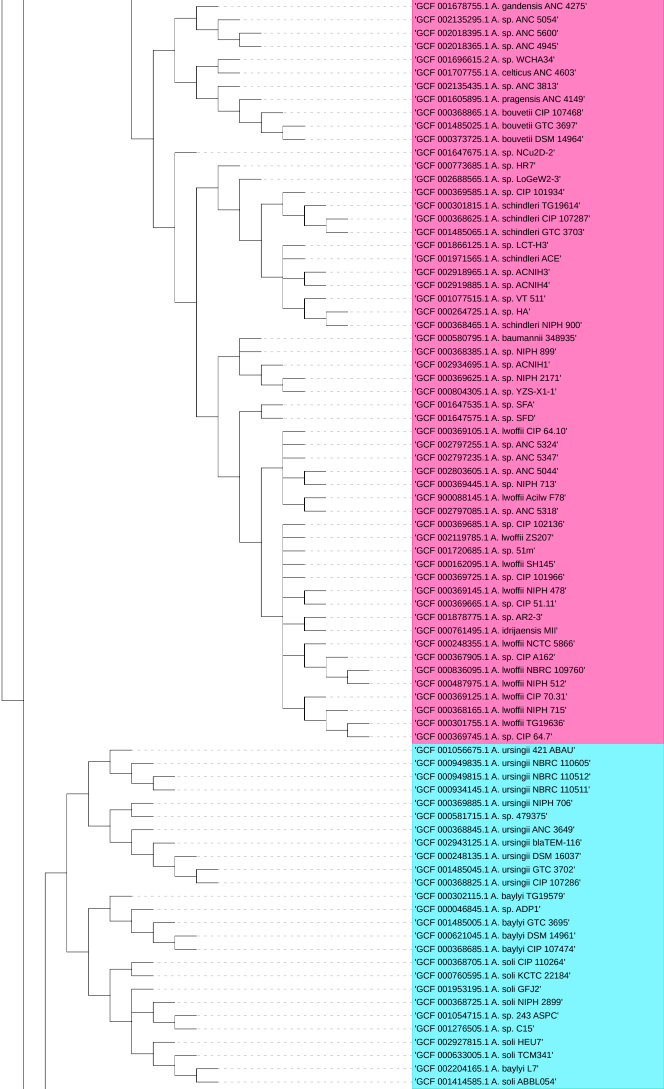

'GCF 001678755.1 A. gandensis ANC 4275'
'GCF 002135295.1 A. sp. ANC 5054'
'GCF 002018395.1 A. sp. ANC 5600'
'GCF 002018365.1 A. sp. ANC 4945'
'GCF 001696615.2 A. sp. WCHA34'
'GCF 001707755.1 A. celticus ANC 4603'
'GCF 002135435.1 A. sp. ANC 3813'
'GCF 001605895.1 A. pragensis ANC 4149'
'GCF 000368865.1 A. bouvetii CIP 107468'
'GCF 001485025.1 A. bouvetii GTC 3697'
'GCF 000373725.1 A. bouvetii DSM 14964'
'GCF 001647675.1 A. sp. NCu2D-2'
'GCF 000773685.1 A. sp. HR7'
'GCF 002688565.1 A. sp. LoGeW2-3'
'GCF 000369585.1 A. sp. CIP 101934'
'GCF 000301815.1 A. schindleri TG19614'
'GCF 000368625.1 A. schindleri CIP 107287'
'GCF 001485065.1 A. schindleri GTC 3703'
'GCF 001866125.1 A. sp. LCT-H3'
'GCF 001971565.1 A. schindleri ACE'
'GCF 002918965.1 A. sp. ACNIH3'
'GCF 002919885.1 A. sp. ACNIH4'
'GCF 001077515.1 A. sp. VT 511'
'GCF 000264725.1 A. sp. HA'
'GCF 000368465.1 A. schindleri NIPH 900'
'GCF 000580795.1 A. baumannii 348935'
'GCF 000368385.1 A. sp. NIPH 899'
'GCF 002934695.1 A. sp. ACNIH1'
'GCF 000369625.1 A. sp. NIPH 2171'
'GCF 000804305.1 A. sp. YZS-X1-1'
'GCF 001647535.1 A. sp. SFA'
'GCF 001647575.1 A. sp. SFD'
'GCF 000369105.1 A. lwoffii CIP 64.10'
'GCF 002797255.1 A. sp. ANC 5324'
'GCF 002797235.1 A. sp. ANC 5347'
'GCF 002803605.1 A. sp. ANC 5044'
'GCF 000369445.1 A. sp. NIPH 713'
'GCF 900088145.1 A. lwoffii Acilw F78'
'GCF 002797085.1 A. sp. ANC 5318'
'GCF 000369685.1 A. sp. CIP 102136'
'GCF 002119785.1 A. lwoffii ZS207'
'GCF 001720685.1 A. sp. 51m'
'GCF 000162095.1 A. lwoffii SH145'
'GCF 000369725.1 A. sp. CIP 101966'
'GCF 000369145.1 A. lwoffii NIPH 478'
'GCF 000369665.1 A. sp. CIP 51.11'
'GCF 001878775.1 A. sp. AR2-3'
'GCF 000761495.1 A. idrijaensis MII'
'GCF 000248355.1 A. lwoffii NCTC 5866'
'GCF 000367905.1 A. sp. CIP A162'
'GCF 000836095.1 A. lwoffii NBRC 109760'
'GCF 000487975.1 A. lwoffii NIPH 512'
'GCF 000369125.1 A. lwoffii CIP 70.31'
'GCF 000368165.1 A. lwoffii NIPH 715'
'GCF 000301755.1 A. lwoffii TG19636'
'GCF 000369745.1 A. sp. CIP 64.7'
'GCF 001056675.1 A. ursingii 421 ABAU'
'GCF 000949835.1 A. ursingii NBRC 110605'
'GCF 000949815.1 A. ursingii NBRC 110512'
'GCF 000934145.1 A. ursingii NBRC 110511'
'GCF 000369885.1 A. ursingii NIPH 706'
'GCF 000581715.1 A. sp. 479375'
'GCF 000368845.1 A. ursingii ANC 3649'
'GCF 002943125.1 A. ursingii blaTEM-116'
'GCF 000248135.1 A. ursingii DSM 16037'
'GCF 001485045.1 A. ursingii GTC 3702'
'GCF 000368825.1 A. ursingii CIP 107286'
'GCF 000302115.1 A. baylyi TG19579'
'GCF 000046845.1 A. sp. ADP1'
'GCF 001485005.1 A. baylyi GTC 3695'
'GCF 000621045.1 A. baylyi DSM 14961'
'GCF 000368685.1 A. baylyi CIP 107474'
'GCF 000368705.1 A. soli CIP 110264'
'GCF 000760595.1 A. soli KCTC 22184'
'GCF 001953195.1 A. soli GFJ2'
'GCF 000368725.1 A. soli NIPH 2899'
'GCF 001054715.1 A. sp. 243 ASPC'
'GCF 001276505.1 A. sp. C15'
'GCF 002927815.1 A. soli HEU7'
'GCF 000633005.1 A. soli TCM341'
'GCF 002204165.1 A. baylyi L7'
'GCF 001414585.1 A. soli ABBL054'

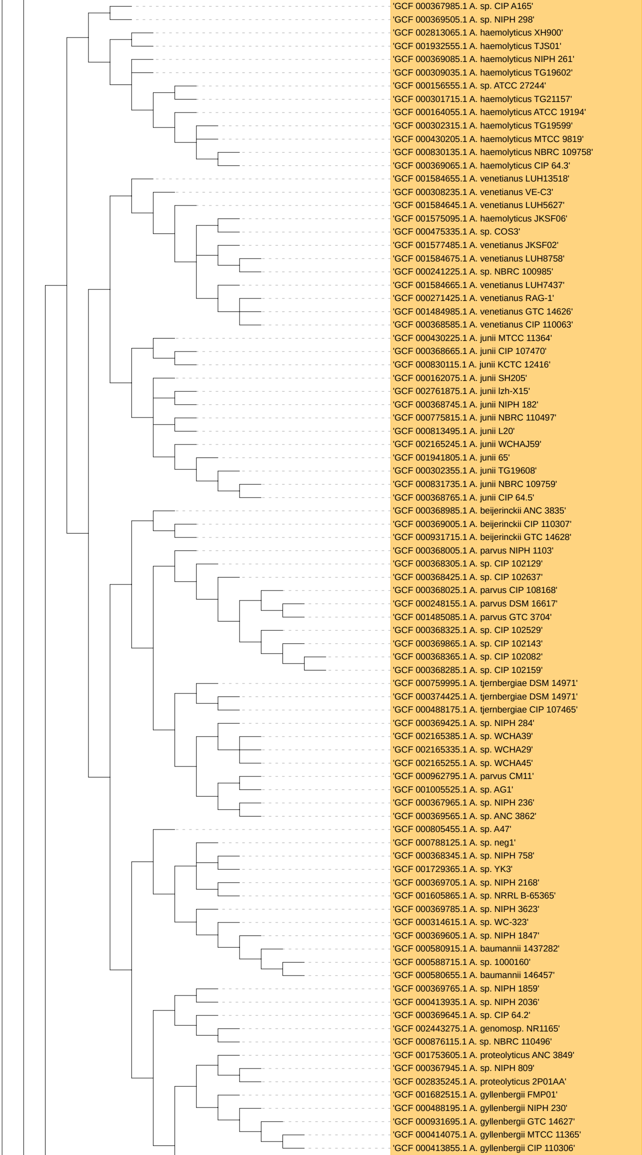
'GCF 000367985.1 A. sp. CIP A165'
'GCF 000369505.1 A. sp. NIPH 298'
'GCF 002813065.1 A. haemolyticus XH900'
'GCF 001932555.1 A. haemolyticus TJS01'
'GCF 000369085.1 A. haemolyticus NIPH 261'
'GCF 000309035.1 A. haemolyticus TG19602'
'GCF 000156555.1 A. sp. ATCC 27244'
'GCF 000301715.1 A. haemolyticus TG21157'
'GCF 000164055.1 A. haemolyticus ATCC 19194'
'GCF 000302315.1 A. haemolyticus TG19599'
'GCF 000430205.1 A. haemolyticus MTCC 9819'
'GCF 000830135.1 A. haemolyticus NBRC 109758'
'GCF 000369065.1 A. haemolyticus CIP 64.3'
'GCF 001584655.1 A. venetianus LUH13518'
'GCF 000308235.1 A. venetianus VE-C3'
'GCF 001584645.1 A. venetianus LUH5627'
'GCF 001575095.1 A. haemolyticus JKSF06'
'GCF 000475335.1 A. sp. COS3'
'GCF 001577485.1 A. venetianus JKSF02'
'GCF 001584675.1 A. venetianus LUH8758'
'GCF 000241225.1 A. sp. NBRC 100985'
'GCF 001584665.1 A. venetianus LUH7437'
'GCF 000271425.1 A. venetianus RAG-1'
'GCF 001484985.1 A. venetianus GTC 14626'
'GCF 000368585.1 A. venetianus CIP 110063'
'GCF 000430225.1 A. junii MTCC 11364'
'GCF 000368665.1 A. junii CIP 107470'
'GCF 000830115.1 A. junii KCTC 12416'
'GCF 000162075.1 A. junii SH205'
'GCF 002761875.1 A. junii lzh-X15'
'GCF 000368745.1 A. junii NIPH 182'
'GCF 000775815.1 A. junii NBRC 110497'
'GCF 000813495.1 A. junii L20'
'GCF 002165245.1 A. junii WCHAJ59'
'GCF 001941805.1 A. junii 65'
'GCF 000302355.1 A. junii TG19608'
'GCF 000831735.1 A. junii NBRC 109759'
'GCF 000368765.1 A. junii CIP 64.5'
'GCF 000368985.1 A. beijerinckii ANC 3835'
'GCF 000369005.1 A. beijerinckii CIP 110307'
'GCF 000931715.1 A. beijerinckii GTC 14628'
'GCF 000368005.1 A. parvus NIPH 1103'
'GCF 000368305.1 A. sp. CIP 102129'
'GCF 000368425.1 A. sp. CIP 102637'
'GCF 000368025.1 A. parvus CIP 108168'
'GCF 000248155.1 A. parvus DSM 16617'
'GCF 001485085.1 A. parvus GTC 3704'
'GCF 000368325.1 A. sp. CIP 102529'
'GCF 000369865.1 A. sp. CIP 102143'
'GCF 000368365.1 A. sp. CIP 102082'
'GCF 000368285.1 A. sp. CIP 102159'
'GCF 000759995.1 A. tjernbergiae DSM 14971'
'GCF 000374425.1 A. tjernbergiae DSM 14971'
'GCF 000488175.1 A. tjernbergiae CIP 107465'
'GCF 000369425.1 A. sp. NIPH 284'
'GCF 002165385.1 A. sp. WCHA39'
'GCF 002165335.1 A. sp. WCHA29'
'GCF 002165255.1 A. sp. WCHA45'
'GCF 000962795.1 A. parvus CM11'
'GCF 001005525.1 A. sp. AG1'
'GCF 000367965.1 A. sp. NIPH 236'
'GCF 000369565.1 A. sp. ANC 3862'
'GCF 000805455.1 A. sp. A47'
'GCF 000788125.1 A. sp. neg1'
'GCF 000368345.1 A. sp. NIPH 758'
'GCF 001729365.1 A. sp. YK3'
'GCF 000369705.1 A. sp. NIPH 2168'
'GCF 001605865.1 A. sp. NRRL B-65365'
'GCF 000369785.1 A. sp. NIPH 3623'
'GCF 000314615.1 A. sp. WC-323'
'GCF 000369605.1 A. sp. NIPH 1847'
'GCF 000580915.1 A. baumannii 1437282'
'GCF 000588715.1 A. sp. 1000160'
'GCF 000580655.1 A. baumannii 146457'
'GCF 000369765.1 A. sp. NIPH 1859'
'GCF 000413935.1 A. sp. NIPH 2036'
'GCF 000369645.1 A. sp. CIP 64.2'
'GCF 002443275.1 A. genomosp. NR1165'
'GCF 000876115.1 A. sp. NBRC 110496'
'GCF 001753605.1 A. proteolyticus ANC 3849'
'GCF 000367945.1 A. sp. NIPH 809'
'GCF 002835245.1 A. proteolyticus 2P01AA'
'GCF 001682515.1 A. gyllenbergii FMP01'
'GCF 000488195.1 A. gyllenbergii NIPH 230'
'GCF 000931695.1 A. gyllenbergii GTC 14627'
'GCF 000414075.1 A. gyllenbergii MTCC 11365'
'GCF 000413855.1 A. gyllenbergii CIP 110306'

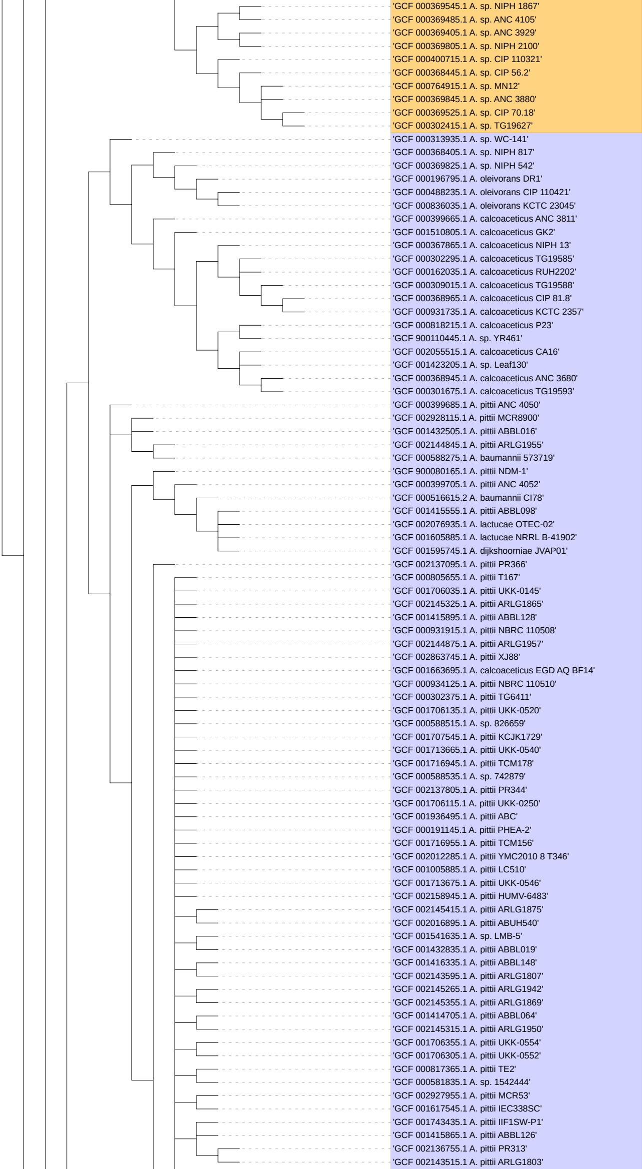
'GCF 000369545.1 A. sp. NIPH 1867'
'GCF 000369485.1 A. sp. ANC 4105'
'GCF 000369405.1 A. sp. ANC 3929'
'GCF 000369805.1 A. sp. NIPH 2100'
'GCF 000400715.1 A. sp. CIP 110321'
'GCF 000368445.1 A. sp. CIP 56.2'
'GCF 000764915.1 A. sp. MN12'
'GCF 000369845.1 A. sp. ANC 3880'
'GCF 000369525.1 A. sp. CIP 70.18'
'GCF 000302415.1 A. sp. TG19627'
'GCF 000313935.1 A. sp. WC-141'
'GCF 000368405.1 A. sp. NIPH 817'
'GCF 000369825.1 A. sp. NIPH 542'
'GCF 000196795.1 A. oleivorans DR1'
'GCF 000488235.1 A. oleivorans CIP 110421'
'GCF 000836035.1 A. oleivorans KCTC 23045'
'GCF 000399665.1 A. calcoaceticus ANC 3811'
'GCF 001510805.1 A. calcoaceticus GK2'
'GCF 000367865.1 A. calcoaceticus NIPH 13'
'GCF 000302295.1 A. calcoaceticus TG19585'
'GCF 000162035.1 A. calcoaceticus RUH2202'
'GCF 000309015.1 A. calcoaceticus TG19588'
'GCF 000368965.1 A. calcoaceticus CIP 81.8'
'GCF 000931735.1 A. calcoaceticus KCTC 2357'
'GCF 000818215.1 A. calcoaceticus P23'
'GCF 900110445.1 A. sp. YR461'
'GCF 002055515.1 A. calcoaceticus CA16'
'GCF 001423205.1 A. sp. Leaf130'
'GCF 000368945.1 A. calcoaceticus ANC 3680'
'GCF 000301675.1 A. calcoaceticus TG19593'
'GCF 000399685.1 A. pittii ANC 4050'
'GCF 002928115.1 A. pittii MCR8900'
'GCF 001432505.1 A. pittii ABBL016'
'GCF 002144845.1 A. pittii ARLG1955'
'GCF 000588275.1 A. baumannii 573719'
'GCF 900080165.1 A. pittii NDM-1'
'GCF 000399705.1 A. pittii ANC 4052'
'GCF 000516615.2 A. baumannii CI78'
'GCF 001415555.1 A. pittii ABBL098'
'GCF 002076935.1 A. lactucae OTEC-02'
'GCF 001605885.1 A. lactucae NRRL B-41902'
'GCF 001595745.1 A. dijkshoorniae JVAP01'
'GCF 002137095.1 A. pittii PR366'
'GCF 000805655.1 A. pittii T167'
'GCF 001706035.1 A. pittii UKK-0145'
'GCF 002145325.1 A. pittii ARLG1865'
'GCF 001415895.1 A. pittii ABBL128'
'GCF 000931915.1 A. pittii NBRC 110508'
'GCF 002144875.1 A. pittii ARLG1957'
'GCF 002863745.1 A. pittii XJ88'
'GCF 001663695.1 A. calcoaceticus EGD AQ BF14'
'GCF 000934125.1 A. pittii NBRC 110510'
'GCF 000302375.1 A. pittii TG6411'
'GCF 001706135.1 A. pittii UKK-0520'
'GCF 000588515.1 A. sp. 826659'
'GCF 001707545.1 A. pittii KCJK1729'
'GCF 001713665.1 A. pittii UKK-0540'
'GCF 001716945.1 A. pittii TCM178'
'GCF 000588535.1 A. sp. 742879'
'GCF 002137805.1 A. pittii PR344'
'GCF 001706115.1 A. pittii UKK-0250'
'GCF 001936495.1 A. pittii ABC'
'GCF 000191145.1 A. pittii PHEA-2'
'GCF 001716955.1 A. pittii TCM156'
'GCF 002012285.1 A. pittii YMC2010 8 T346'
'GCF 001005885.1 A. pittii LC510'
'GCF 001713675.1 A. pittii UKK-0546'
'GCF 002158945.1 A. pittii HUMV-6483'
'GCF 002145415.1 A. pittii ARLG1875'
'GCF 002016895.1 A. pittii ABUH540'
'GCF 001541635.1 A. sp. LMB-5'
'GCF 001432835.1 A. pittii ABBL019'
'GCF 001416335.1 A. pittii ABBL148'
'GCF 002143595.1 A. pittii ARLG1807'
'GCF 002145265.1 A. pittii ARLG1942'
'GCF 002145355.1 A. pittii ARLG1869'
'GCF 001414705.1 A. pittii ABBL064'
'GCF 002145315.1 A. pittii ARLG1950'
'GCF 001706355.1 A. pittii UKK-0554'
'GCF 001706305.1 A. pittii UKK-0552'
'GCF 000817365.1 A. pittii TE2'
'GCF 000581835.1 A. sp. 1542444'
'GCF 002927955.1 A. pittii MCR53'
'GCF 001617545.1 A. pittii IEC338SC'
'GCF 001743435.1 A. pittii IIF1SW-P1'
'GCF 001415865.1 A. pittii ABBL126'
'GCF 002136755.1 A. pittii PR313'
'GCF 002143515.1 A. pittii ARLG1803'

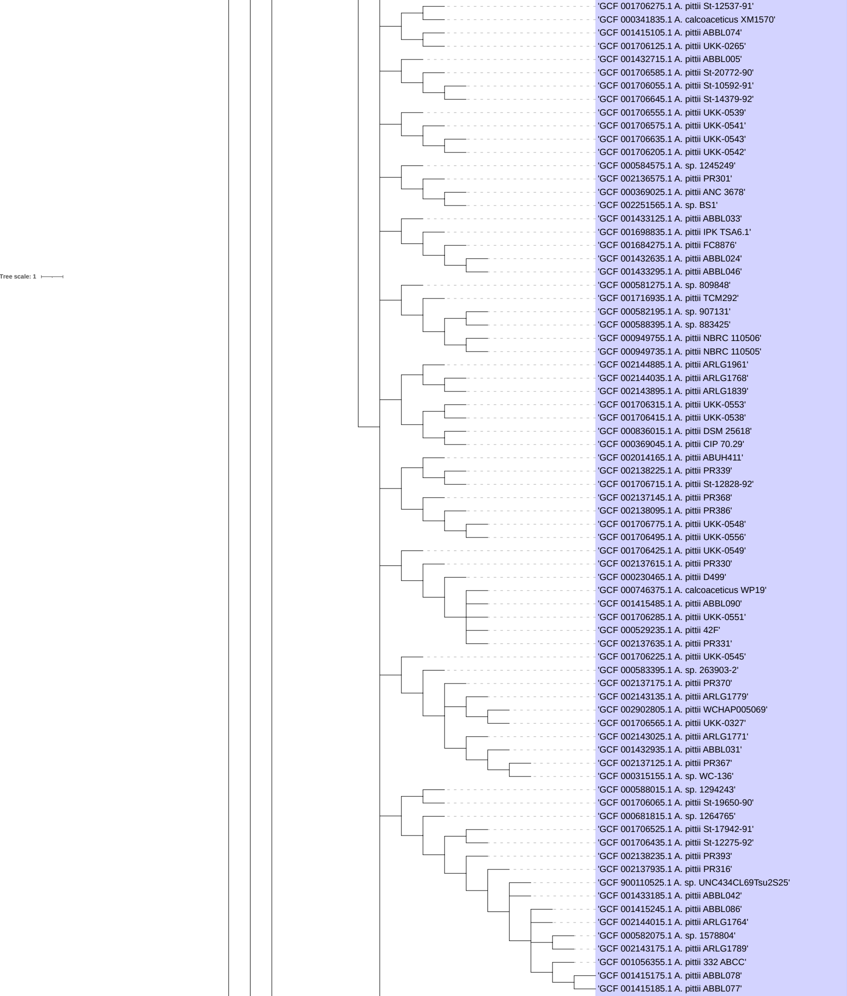
Tree scale: 1
'GCF 001706275.1 A. pittii St-12537-91'
'GCF 000341835.1 A. calcoaceticus XM1570'
'GCF 001415105.1 A. pittii ABBL074'
'GCF 001706125.1 A. pittii UKK-0265'
'GCF 001432715.1 A. pittii ABBL005'
'GCF 001706585.1 A. pittii St-20772-90'
'GCF 001706055.1 A. pittii St-10592-91'
'GCF 001706645.1 A. pittii St-14379-92'
'GCF 001706555.1 A. pittii UKK-0539'
'GCF 001706575.1 A. pittii UKK-0541'
'GCF 001706635.1 A. pittii UKK-0543'
'GCF 001706205.1 A. pittii UKK-0542'
'GCF 000584575.1 A. sp. 1245249'
'GCF 002136575.1 A. pittii PR301'
'GCF 000369025.1 A. pittii ANC 3678'
'GCF 002251565.1 A. sp. BS1'
'GCF 001433125.1 A. pittii ABBL033'
'GCF 001698835.1 A. pittii IPK TSA6.1'
'GCF 001684275.1 A. pittii FC8876'
'GCF 001432635.1 A. pittii ABBL024'
'GCF 001433295.1 A. pittii ABBL046'
'GCF 000581275.1 A. sp. 809848'
'GCF 001716935.1 A. pittii TCM292'
'GCF 000582195.1 A. sp. 907131'
'GCF 000588395.1 A. sp. 883425'
'GCF 000949755.1 A. pittii NBRC 110506'
'GCF 000949735.1 A. pittii NBRC 110505'
'GCF 002144885.1 A. pittii ARLG1961'
'GCF 002144035.1 A. pittii ARLG1768'
'GCF 002143895.1 A. pittii ARLG1839'
'GCF 001706315.1 A. pittii UKK-0553'
'GCF 001706415.1 A. pittii UKK-0538'
'GCF 000836015.1 A. pittii DSM 25618'
'GCF 000369045.1 A. pittii CIP 70.29'
'GCF 002014165.1 A. pittii ABUH411'
'GCF 002138225.1 A. pittii PR339'
'GCF 001706715.1 A. pittii St-12828-92'
'GCF 002137145.1 A. pittii PR368'
'GCF 002138095.1 A. pittii PR386'
'GCF 001706775.1 A. pittii UKK-0548'
'GCF 001706495.1 A. pittii UKK-0556'
'GCF 001706425.1 A. pittii UKK-0549'
'GCF 002137615.1 A. pittii PR330'
'GCF 000230465.1 A. pittii D499'
'GCF 000746375.1 A. calcoaceticus WP19'
'GCF 001415485.1 A. pittii ABBL090'
'GCF 001706285.1 A. pittii UKK-0551'
'GCF 000529235.1 A. pittii 42F'
'GCF 002137635.1 A. pittii PR331'
'GCF 001706225.1 A. pittii UKK-0545'
'GCF 000583395.1 A. sp. 263903-2'
'GCF 002137175.1 A. pittii PR370'
'GCF 002143135.1 A. pittii ARLG1779'
'GCF 002902805.1 A. pittii WCHAP005069'
'GCF 001706565.1 A. pittii UKK-0327'
'GCF 002143025.1 A. pittii ARLG1771'
'GCF 001432935.1 A. pittii ABBL031'
'GCF 002137125.1 A. pittii PR367'
'GCF 000315155.1 A. sp. WC-136'
'GCF 000588015.1 A. sp. 1294243'
'GCF 001706065.1 A. pittii St-19650-90'
'GCF 000681815.1 A. sp. 1264765'
'GCF 001706525.1 A. pittii St-17942-91'
'GCF 001706435.1 A. pittii St-12275-92'
'GCF 002138235.1 A. pittii PR393'
'GCF 002137935.1 A. pittii PR316'
'GCF 900110525.1 A. sp. UNC434CL69Tsu2S25'
'GCF 001433185.1 A. pittii ABBL042'
'GCF 001415245.1 A. pittii ABBL086'
'GCF 002144015.1 A. pittii ARLG1764'
'GCF 000582075.1 A. sp. 1578804'
'GCF 002143175.1 A. pittii ARLG1789'
'GCF 001056355.1 A. pittii 332 ABCC'
'GCF 001415175.1 A. pittii ABBL078'
'GCF 001415185.1 A. pittii ABBL077'

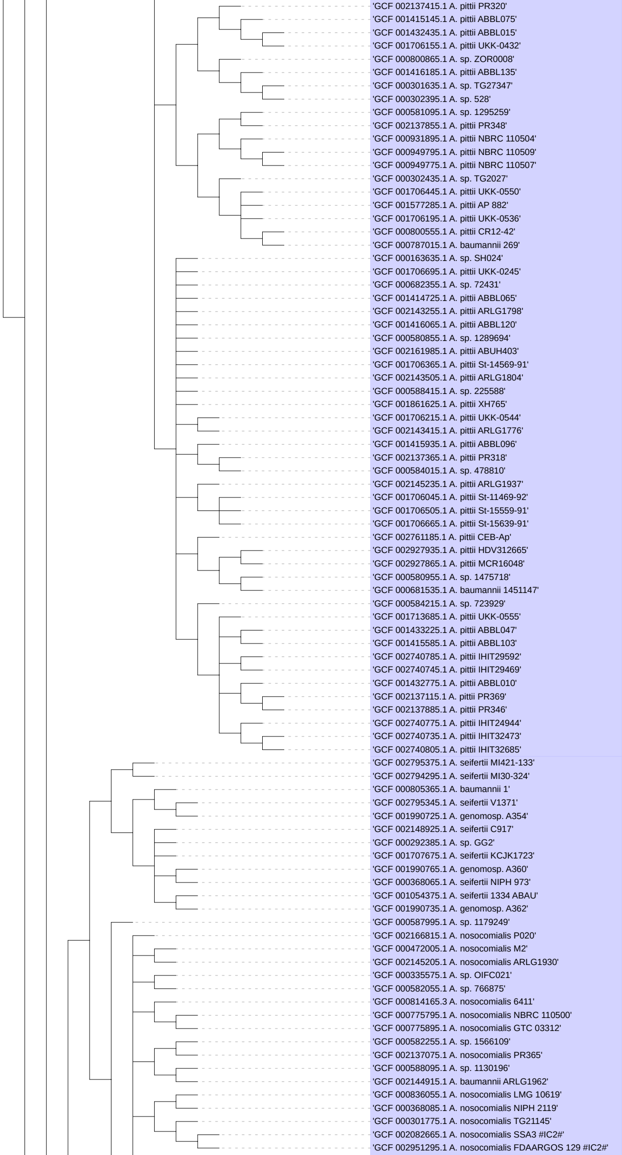
'GCF 002137415.1 A. pittii PR320'
'GCF 001415145.1 A. pittii ABBL075'
'GCF 001432435.1 A. pittii ABBL015'
'GCF 001706155.1 A. pittii UKK-0432'
'GCF 000800865.1 A. sp. ZOR0008'
'GCF 001416185.1 A. pittii ABBL135'
'GCF 000301635.1 A. sp. TG27347'
'GCF 000302395.1 A. sp. 528'
'GCF 000581095.1 A. sp. 1295259'
'GCF 002137855.1 A. pittii PR348'
'GCF 000931895.1 A. pittii NBRC 110504'
'GCF 000949795.1 A. pittii NBRC 110509'
'GCF 000949775.1 A. pittii NBRC 110507'
'GCF 000302435.1 A. sp. TG2027'
'GCF 001706445.1 A. pittii UKK-0550'
'GCF 001577285.1 A. pittii AP 882'
'GCF 001706195.1 A. pittii UKK-0536'
'GCF 000800555.1 A. pittii CR12-42'
'GCF 000787015.1 A. baumannii 269'
'GCF 000163635.1 A. sp. SH024'
'GCF 001706695.1 A. pittii UKK-0245'
'GCF 000682355.1 A. sp. 72431'
'GCF 001414725.1 A. pittii ABBL065'
'GCF 002143255.1 A. pittii ARLG1798'
'GCF 001416065.1 A. pittii ABBL120'
'GCF 000580855.1 A. sp. 1289694'
'GCF 002161985.1 A. pittii ABUH403'
'GCF 001706365.1 A. pittii St-14569-91'
'GCF 002143505.1 A. pittii ARLG1804'
'GCF 000588415.1 A. sp. 225588'
'GCF 001861625.1 A. pittii XH765'
'GCF 001706215.1 A. pittii UKK-0544'
'GCF 002143415.1 A. pittii ARLG1776'
'GCF 001415935.1 A. pittii ABBL096'
'GCF 002137365.1 A. pittii PR318'
'GCF 000584015.1 A. sp. 478810'
'GCF 002145235.1 A. pittii ARLG1937'
'GCF 001706045.1 A. pittii St-11469-92'
'GCF 001706505.1 A. pittii St-15559-91'
'GCF 001706665.1 A. pittii St-15639-91'
'GCF 002761185.1 A. pittii CEB-Ap'
'GCF 002927935.1 A. pittii HDV312665'
'GCF 002927865.1 A. pittii MCR16048'
'GCF 000580955.1 A. sp. 1475718'
'GCF 000681535.1 A. baumannii 1451147'
'GCF 000584215.1 A. sp. 723929'
'GCF 001713685.1 A. pittii UKK-0555'
'GCF 001433225.1 A. pittii ABBL047'
'GCF 001415585.1 A. pittii ABBL103'
'GCF 002740785.1 A. pittii IHIT29592'
'GCF 002740745.1 A. pittii IHIT29469'
'GCF 001432775.1 A. pittii ABBL010'
'GCF 002137115.1 A. pittii PR369'
'GCF 002137885.1 A. pittii PR346'
'GCF 002740775.1 A. pittii IHIT24944'
'GCF 002740735.1 A. pittii IHIT32473'
'GCF 002740805.1 A. pittii IHIT32685'
'GCF 002795375.1 A. seifertii MI421-133'
'GCF 002794295.1 A. seifertii MI30-324'
'GCF 000805365.1 A. baumannii 1'
'GCF 002795345.1 A. seifertii V1371'
'GCF 001990725.1 A. genomosp. A354'
'GCF 002148925.1 A. seifertii C917'
'GCF 000292385.1 A. sp. GG2'
'GCF 001707675.1 A. seifertii KCJK1723'
'GCF 001990765.1 A. genomosp. A360'
'GCF 000368065.1 A. seifertii NIPH 973'
'GCF 001054375.1 A. seifertii 1334 ABAU'
'GCF 001990735.1 A. genomosp. A362'
'GCF 000587995.1 A. sp. 1179249'
'GCF 002166815.1 A. nosocomialis P020'
'GCF 000472005.1 A. nosocomialis M2'
'GCF 002145205.1 A. nosocomialis ARLG1930'
'GCF 000335575.1 A. sp. OIFC021'
'GCF 000582055.1 A. sp. 766875'
'GCF 000814165.3 A. nosocomialis 6411'
'GCF 000775795.1 A. nosocomialis NBRC 110500'
'GCF 000775895.1 A. nosocomialis GTC 03312'
'GCF 000582255.1 A. sp. 1566109'
'GCF 002137075.1 A. nosocomialis PR365'
'GCF 000588095.1 A. sp. 1130196'
'GCF 002144915.1 A. baumannii ARLG1962'
'GCF 000836055.1 A. nosocomialis LMG 10619'
'GCF 000368085.1 A. nosocomialis NIPH 2119'
'GCF 000301775.1 A. nosocomialis TG21145'
'GCF 002082665.1 A. nosocomialis SSA3 #IC2#'
'GCF 002951295.1 A. nosocomialis FDAARGOS 129 #IC2#'

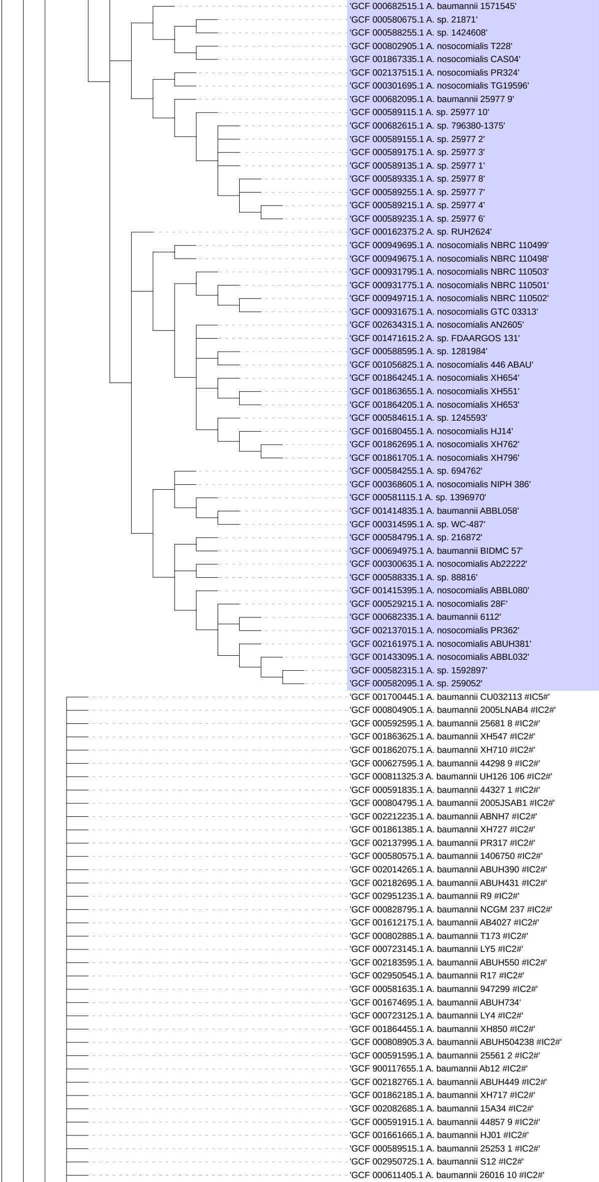
'GCF 000682515.1 A. baumannii 1571545'
'GCF 000580675.1 A. sp. 21871'
'GCF 000588255.1 A. sp. 1424608'
'GCF 000802905.1 A. nosocomialis T228'
'GCF 001867335.1 A. nosocomialis CAS04'
'GCF 002137515.1 A. nosocomialis PR324'
'GCF 000301695.1 A. nosocomialis TG19596'
'GCF 000682095.1 A. baumannii 25977 9'
'GCF 000589115.1 A. sp. 25977 10'
'GCF 000682615.1 A. sp. 796380-1375'
'GCF 000589155.1 A. sp. 25977 2'
'GCF 000589175.1 A. sp. 25977 3'
'GCF 000589135.1 A. sp. 25977 1'
'GCF 000589335.1 A. sp. 25977 8'
'GCF 000589255.1 A. sp. 25977 7'
'GCF 000589215.1 A. sp. 25977 4'
'GCF 000589235.1 A. sp. 25977 6'
'GCF 000162375.2 A. sp. RUH2624'
'GCF 000949695.1 A. nosocomialis NBRC 110499'
'GCF 000949675.1 A. nosocomialis NBRC 110498'
'GCF 000931795.1 A. nosocomialis NBRC 110503'
'GCF 000931775.1 A. nosocomialis NBRC 110501'
'GCF 000949715.1 A. nosocomialis NBRC 110502'
'GCF 000931675.1 A. nosocomialis GTC 03313'
'GCF 002634315.1 A. nosocomialis AN2605'
'GCF 001471615.2 A. sp. FDAARGOS 131'
'GCF 000588595.1 A. sp. 1281984'
'GCF 001056825.1 A. nosocomialis 446 ABAU'
'GCF 001864245.1 A. nosocomialis XH654'
'GCF 001863655.1 A. nosocomialis XH551'
'GCF 001864205.1 A. nosocomialis XH653'
'GCF 000584615.1 A. sp. 1245593'
'GCF 001680455.1 A. nosocomialis HJ14'
'GCF 001862695.1 A. nosocomialis XH762'
'GCF 001861705.1 A. nosocomialis XH796'
'GCF 000584255.1 A. sp. 694762'
'GCF 000368605.1 A. nosocomialis NIPH 386'
'GCF 000581115.1 A. sp. 1396970'
'GCF 001414835.1 A. baumannii ABBL058'
'GCF 000314595.1 A. sp. WC-487'
'GCF 000584795.1 A. sp. 216872'
'GCF 000694975.1 A. baumannii BIDMC 57'
'GCF 000300635.1 A. nosocomialis Ab22222'
'GCF 000588335.1 A. sp. 88816'
'GCF 001415395.1 A. nosocomialis ABBL080'
'GCF 000529215.1 A. nosocomialis 28F'
'GCF 000682335.1 A. baumannii 6112'
'GCF 002137015.1 A. nosocomialis PR362'
'GCF 002161975.1 A. nosocomialis ABUH381'
'GCF 001433095.1 A. nosocomialis ABBL032'
'GCF 000582315.1 A. sp. 1592897'
'GCF 000582095.1 A. sp. 259052'
'GCF 001700445.1 A. baumannii CU032113 #IC5#'
'GCF 000804905.1 A. baumannii 2005LNAB4 #IC2#'
'GCF 000592595.1 A. baumannii 25681 8 #IC2#'
'GCF 001863625.1 A. baumannii XH547 #IC2#'
'GCF 001862075.1 A. baumannii XH710 #IC2#'
'GCF 000627595.1 A. baumannii 44298 9 #IC2#'
'GCF 000811325.3 A. baumannii UH126 106 #IC2#'
'GCF 000591835.1 A. baumannii 44327 1 #IC2#'
'GCF 000804795.1 A. baumannii 2005JSAB1 #IC2#'
'GCF 002212235.1 A. baumannii ABNH7 #IC2#'
'GCF 001861385.1 A. baumannii XH727 #IC2#'
'GCF 002137995.1 A. baumannii PR317 #IC2#'
'GCF 000580575.1 A. baumannii 1406750 #IC2#'
'GCF 002014265.1 A. baumannii ABUH390 #IC2#'
'GCF 002182695.1 A. baumannii ABUH431 #IC2#'
'GCF 002951235.1 A. baumannii R9 #IC2#'
'GCF 000828795.1 A. baumannii NCGM 237 #IC2#'
'GCF 001612175.1 A. baumannii AB4027 #IC2#'
'GCF 000802885.1 A. baumannii T173 #IC2#'
'GCF 000723145.1 A. baumannii LY5 #IC2#'
'GCF 002183595.1 A. baumannii ABUH550 #IC2#'
'GCF 002950545.1 A. baumannii R17 #IC2#'
'GCF 000581635.1 A. baumannii 947299 #IC2#'
'GCF 001674695.1 A. baumannii ABUH734'
'GCF 000723125.1 A. baumannii LY4 #IC2#'
'GCF 001864455.1 A. baumannii XH850 #IC2#'
'GCF 000808905.3 A. baumannii ABUH504238 #IC2#'
'GCF 000591595.1 A. baumannii 25561 2 #IC2#'
'GCF 900117655.1 A. baumannii Ab12 #IC2#'
'GCF 002182765.1 A. baumannii ABUH449 #IC2#'
'GCF 001862185.1 A. baumannii XH717 #IC2#'
'GCF 002082685.1 A. baumannii 15A34 #IC2#'
'GCF 000591915.1 A. baumannii 44857 9 #IC2#'
'GCF 001661665.1 A. baumannii HJ01 #IC2#'
'GCF 000589515.1 A. baumannii 25253 1 #IC2#'
'GCF 002950725.1 A. baumannii S12 #IC2#'
'GCF 000611405.1 A. baumannii 26016 10 #IC2#'

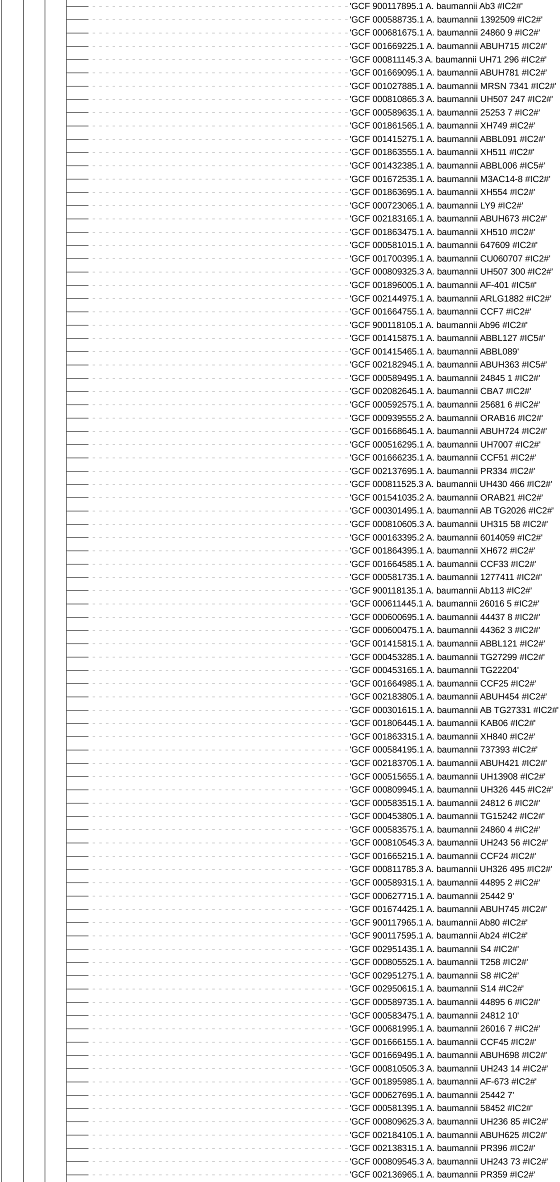
'GCF 900117895.1 A. baumannii Ab3 #IC2#'
'GCF 000588735.1 A. baumannii 1392509 #IC2#'
'GCF 000681675.1 A. baumannii 24860 9 #IC2#'
'GCF 001669225.1 A. baumannii ABUH715 #IC2#'
'GCF 000811145.3 A. baumannii UH71 296 #IC2#'
'GCF 001669095.1 A. baumannii ABUH781 #IC2#'
'GCF 001027885.1 A. baumannii MRSN 7341 #IC2#'
'GCF 000810865.3 A. baumannii UH507 247 #IC2#'
'GCF 000589635.1 A. baumannii 25253 7 #IC2#'
'GCF 001861565.1 A. baumannii XH749 #IC2#'
'GCF 001415275.1 A. baumannii ABBL091 #IC2#'
'GCF 001863555.1 A. baumannii XH511 #IC2#'
'GCF 001432385.1 A. baumannii ABBL006 #IC5#'
'GCF 001672535.1 A. baumannii M3AC14-8 #IC2#'
'GCF 001863695.1 A. baumannii XH554 #IC2#'
'GCF 000723065.1 A. baumannii LY9 #IC2#'
'GCF 002183165.1 A. baumannii ABUH673 #IC2#'
'GCF 001863475.1 A. baumannii XH510 #IC2#'
'GCF 000581015.1 A. baumannii 647609 #IC2#'
'GCF 001700395.1 A. baumannii CU060707 #IC2#'
'GCF 000809325.3 A. baumannii UH507 300 #IC2#'
'GCF 001896005.1 A. baumannii AF-401 #IC5#'
'GCF 002144975.1 A. baumannii ARLG1882 #IC2#'
'GCF 001664755.1 A. baumannii CCF7 #IC2#'
'GCF 900118105.1 A. baumannii Ab96 #IC2#'
'GCF 001415875.1 A. baumannii ABBL127 #IC5#'
'GCF 001415465.1 A. baumannii ABBL089'
'GCF 002182945.1 A. baumannii ABUH363 #IC5#'
'GCF 000589495.1 A. baumannii 24845 1 #IC2#'
'GCF 002082645.1 A. baumannii CBA7 #IC2#'
'GCF 000592575.1 A. baumannii 25681 6 #IC2#'
'GCF 000939555.2 A. baumannii ORAB16 #IC2#'
'GCF 001668645.1 A. baumannii ABUH724 #IC2#'
'GCF 000516295.1 A. baumannii UH7007 #IC2#'
'GCF 001666235.1 A. baumannii CCF51 #IC2#'
'GCF 002137695.1 A. baumannii PR334 #IC2#'
'GCF 000811525.3 A. baumannii UH430 466 #IC2#'
'GCF 001541035.2 A. baumannii ORAB21 #IC2#'
'GCF 000301495.1 A. baumannii AB TG2026 #IC2#'
'GCF 000810605.3 A. baumannii UH315 58 #IC2#'
'GCF 000163395.2 A. baumannii 6014059 #IC2#'
'GCF 001864395.1 A. baumannii XH672 #IC2#'
'GCF 001664585.1 A. baumannii CCF33 #IC2#'
'GCF 000581735.1 A. baumannii 1277411 #IC2#'
'GCF 900118135.1 A. baumannii Ab113 #IC2#'
'GCF 000611445.1 A. baumannii 26016 5 #IC2#'
'GCF 000600695.1 A. baumannii 44437 8 #IC2#'
'GCF 000600475.1 A. baumannii 44362 3 #IC2#'
'GCF 001415815.1 A. baumannii ABBL121 #IC2#'
'GCF 000453285.1 A. baumannii TG27299 #IC2#'
'GCF 000453165.1 A. baumannii TG22204'
'GCF 001664985.1 A. baumannii CCF25 #IC2#'
'GCF 002183805.1 A. baumannii ABUH454 #IC2#'
'GCF 000301615.1 A. baumannii AB TG27331 #IC2#'
'GCF 001806445.1 A. baumannii KAB06 #IC2#'
'GCF 001863315.1 A. baumannii XH840 #IC2#'
'GCF 000584195.1 A. baumannii 737393 #IC2#'
'GCF 002183705.1 A. baumannii ABUH421 #IC2#'
'GCF 000515655.1 A. baumannii UH13908 #IC2#'
'GCF 000809945.1 A. baumannii UH326 445 #IC2#'
'GCF 000583515.1 A. baumannii 24812 6 #IC2#'
'GCF 000453805.1 A. baumannii TG15242 #IC2#'
'GCF 000583575.1 A. baumannii 24860 4 #IC2#'
'GCF 000810545.3 A. baumannii UH243 56 #IC2#'
'GCF 001665215.1 A. baumannii CCF24 #IC2#'
'GCF 000811785.3 A. baumannii UH326 495 #IC2#'
'GCF 000589315.1 A. baumannii 44895 2 #IC2#'
'GCF 000627715.1 A. baumannii 25442 9'
'GCF 001674425.1 A. baumannii ABUH745 #IC2#'
'GCF 900117965.1 A. baumannii Ab80 #IC2#'
'GCF 900117595.1 A. baumannii Ab24 #IC2#'
'GCF 002951435.1 A. baumannii S4 #IC2#'
'GCF 000805525.1 A. baumannii T258 #IC2#'
'GCF 002951275.1 A. baumannii S8 #IC2#'
'GCF 002950615.1 A. baumannii S14 #IC2#'
'GCF 000589735.1 A. baumannii 44895 6 #IC2#'
'GCF 000583475.1 A. baumannii 24812 10'
'GCF 000681995.1 A. baumannii 26016 7 #IC2#'
'GCF 001666155.1 A. baumannii CCF45 #IC2#'
'GCF 001669495.1 A. baumannii ABUH698 #IC2#'
'GCF 000810505.3 A. baumannii UH243 14 #IC2#'
'GCF 001895985.1 A. baumannii AF-673 #IC2#'
'GCF 000627695.1 A. baumannii 25442 7'
'GCF 000581395.1 A. baumannii 58452 #IC2#'
'GCF 000809625.3 A. baumannii UH236 85 #IC2#'
'GCF 002184105.1 A. baumannii ABUH625 #IC2#'
'GCF 002138315.1 A. baumannii PR396 #IC2#'
'GCF 000809545.3 A. baumannii UH243 73 #IC2#'
'GCF 002136965.1 A. baumannii PR359 #IC2#'

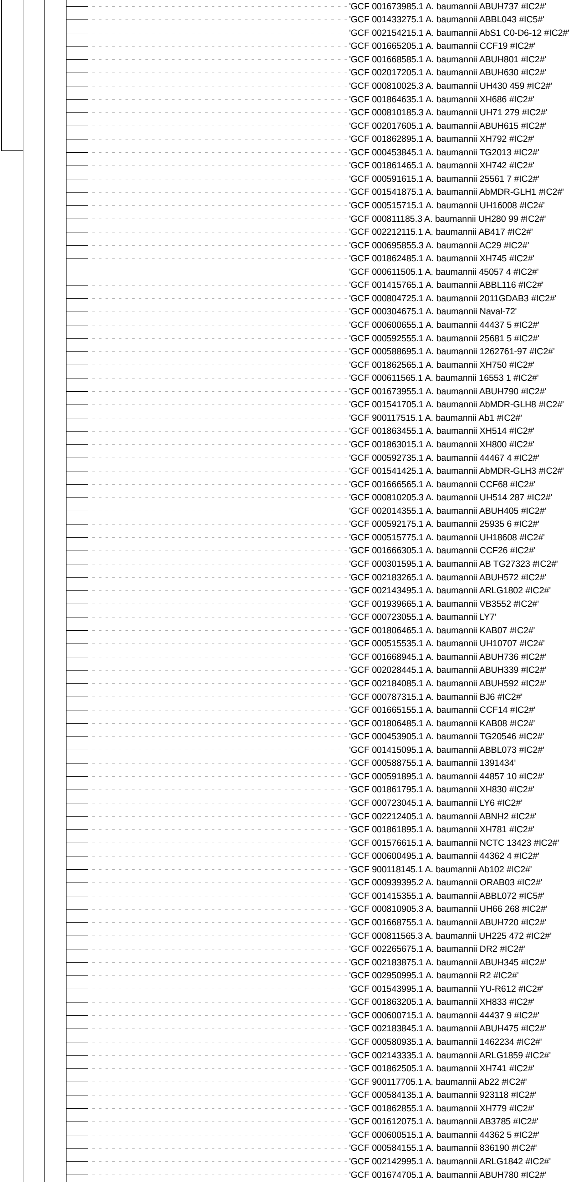

'GCF 001673985.1 A. baumannii ABUH737 #IC2#'
'GCF 001433275.1 A. baumannii ABBL043 #IC5#'
'GCF 002154215.1 A. baumannii AbS1 C0-D6-12 #IC2#'
'GCF 001665205.1 A. baumannii CCF19 #IC2#'
'GCF 001668585.1 A. baumannii ABUH801 #IC2#'
'GCF 002017205.1 A. baumannii ABUH630 #IC2#'
'GCF 000810025.3 A. baumannii UH430 459 #IC2#'
'GCF 001864635.1 A. baumannii XH686 #IC2#'
'GCF 000810185.3 A. baumannii UH71 279 #IC2#'
'GCF 002017605.1 A. baumannii ABUH615 #IC2#'
'GCF 001862895.1 A. baumannii XH792 #IC2#'
'GCF 000453845.1 A. baumannii TG2013 #IC2#'
'GCF 001861465.1 A. baumannii XH742 #IC2#'
'GCF 000591615.1 A. baumannii 25561 7 #IC2#'
'GCF 001541875.1 A. baumannii AbMDR-GLH1 #IC2#'
'GCF 000515715.1 A. baumannii UH16008 #IC2#'
'GCF 000811185.3 A. baumannii UH280 99 #IC2#'
'GCF 002212115.1 A. baumannii AB417 #IC2#'
'GCF 000695855.3 A. baumannii AC29 #IC2#'
'GCF 001862485.1 A. baumannii XH745 #IC2#'
'GCF 000611505.1 A. baumannii 45057 4 #IC2#'
'GCF 001415765.1 A. baumannii ABBL116 #IC2#'
'GCF 000804725.1 A. baumannii 2011GDAB3 #IC2#'
'GCF 000304675.1 A. baumannii Naval-72'
'GCF 000600655.1 A. baumannii 44437 5 #IC2#'
'GCF 000592555.1 A. baumannii 25681 5 #IC2#'
'GCF 000588695.1 A. baumannii 1262761-97 #IC2#'
'GCF 001862565.1 A. baumannii XH750 #IC2#'
'GCF 000611565.1 A. baumannii 16553 1 #IC2#'
'GCF 001673955.1 A. baumannii ABUH790 #IC2#'
'GCF 001541705.1 A. baumannii AbMDR-GLH8 #IC2#'
'GCF 900117515.1 A. baumannii Ab1 #IC2#'
'GCF 001863455.1 A. baumannii XH514 #IC2#'
'GCF 001863015.1 A. baumannii XH800 #IC2#'
'GCF 000592735.1 A. baumannii 44467 4 #IC2#'
'GCF 001541425.1 A. baumannii AbMDR-GLH3 #IC2#'
'GCF 001666565.1 A. baumannii CCF68 #IC2#'
'GCF 000810205.3 A. baumannii UH514 287 #IC2#'
'GCF 002014355.1 A. baumannii ABUH405 #IC2#'
'GCF 000592175.1 A. baumannii 25935 6 #IC2#'
'GCF 000515775.1 A. baumannii UH18608 #IC2#'
'GCF 001666305.1 A. baumannii CCF26 #IC2#'
'GCF 000301595.1 A. baumannii AB TG27323 #IC2#'
'GCF 002183265.1 A. baumannii ABUH572 #IC2#'
'GCF 002143495.1 A. baumannii ARLG1802 #IC2#'
'GCF 001939665.1 A. baumannii VB3552 #IC2#'
'GCF 000723055.1 A. baumannii LY7'
'GCF 001806465.1 A. baumannii KAB07 #IC2#'
'GCF 000515535.1 A. baumannii UH10707 #IC2#'
'GCF 001668945.1 A. baumannii ABUH736 #IC2#'
'GCF 002028445.1 A. baumannii ABUH339 #IC2#'
'GCF 002184085.1 A. baumannii ABUH592 #IC2#'
'GCF 000787315.1 A. baumannii BJ6 #IC2#'
'GCF 001665155.1 A. baumannii CCF14 #IC2#'
'GCF 001806485.1 A. baumannii KAB08 #IC2#'
'GCF 000453905.1 A. baumannii TG20546 #IC2#'
'GCF 001415095.1 A. baumannii ABBL073 #IC2#'
'GCF 000588755.1 A. baumannii 1391434'
'GCF 000591895.1 A. baumannii 44857 10 #IC2#'
'GCF 001861795.1 A. baumannii XH830 #IC2#'
'GCF 000723045.1 A. baumannii LY6 #IC2#'
'GCF 002212405.1 A. baumannii ABNH2 #IC2#'
'GCF 001861895.1 A. baumannii XH781 #IC2#'
'GCF 001576615.1 A. baumannii NCTC 13423 #IC2#'
'GCF 000600495.1 A. baumannii 44362 4 #IC2#'
'GCF 900118145.1 A. baumannii Ab102 #IC2#'
'GCF 000939395.2 A. baumannii ORAB03 #IC2#'
'GCF 001415355.1 A. baumannii ABBL072 #IC5#'
'GCF 000810905.3 A. baumannii UH66 268 #IC2#'
'GCF 001668755.1 A. baumannii ABUH720 #IC2#'
'GCF 000811565.3 A. baumannii UH225 472 #IC2#'
'GCF 002265675.1 A. baumannii DR2 #IC2#'
'GCF 002183875.1 A. baumannii ABUH345 #IC2#'
'GCF 002950995.1 A. baumannii R2 #IC2#'
'GCF 001543995.1 A. baumannii YU-R612 #IC2#'
'GCF 001863205.1 A. baumannii XH833 #IC2#'
'GCF 000600715.1 A. baumannii 44437 9 #IC2#'
'GCF 002183845.1 A. baumannii ABUH475 #IC2#'
'GCF 000580935.1 A. baumannii 1462234 #IC2#'
'GCF 002143335.1 A. baumannii ARLG1859 #IC2#'
'GCF 001862505.1 A. baumannii XH741 #IC2#'
'GCF 900117705.1 A. baumannii Ab22 #IC2#'
'GCF 000584135.1 A. baumannii 923118 #IC2#'
'GCF 001862855.1 A. baumannii XH779 #IC2#'
'GCF 001612075.1 A. baumannii AB3785 #IC2#'
'GCF 000600515.1 A. baumannii 44362 5 #IC2#'
'GCF 000584155.1 A. baumannii 836190 #IC2#'
'GCF 002142995.1 A. baumannii ARLG1842 #IC2#'
'GCF 001674705.1 A. baumannii ABUH780 #IC2#'

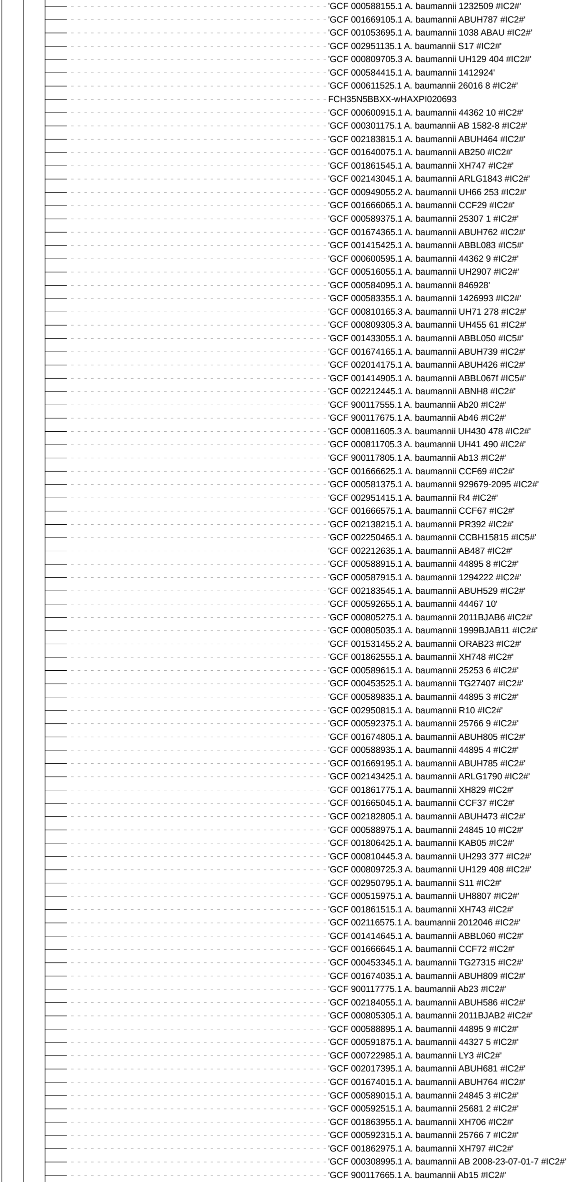
'GCF 000588155.1 A. baumannii 1232509 #IC2#'
'GCF 001669105.1 A. baumannii ABUH787 #IC2#'
'GCF 001053695.1 A. baumannii 1038 ABAU #IC2#'
'GCF 002951135.1 A. baumannii S17 #IC2#'
'GCF 000809705.3 A. baumannii UH129 404 #IC2#'
'GCF 000584415.1 A. baumannii 1412924'
'GCF 000611525.1 A. baumannii 26016 8 #IC2#'
FCH35N5BBXX-wHAXPI020693
'GCF 000600915.1 A. baumannii 44362 10 #IC2#'
'GCF 000301175.1 A. baumannii AB 1582-8 #IC2#'
'GCF 002183815.1 A. baumannii ABUH464 #IC2#'
'GCF 001640075.1 A. baumannii AB250 #IC2#'
'GCF 001861545.1 A. baumannii XH747 #IC2#'
'GCF 002143045.1 A. baumannii ARLG1843 #IC2#'
'GCF 000949055.2 A. baumannii UH66 253 #IC2#'
'GCF 001666065.1 A. baumannii CCF29 #IC2#'
'GCF 000589375.1 A. baumannii 25307 1 #IC2#'
'GCF 001674365.1 A. baumannii ABUH762 #IC2#'
'GCF 001415425.1 A. baumannii ABBL083 #IC5#'
'GCF 000600595.1 A. baumannii 44362 9 #IC2#'
'GCF 000516055.1 A. baumannii UH2907 #IC2#'
'GCF 000584095.1 A. baumannii 846928'
'GCF 000583355.1 A. baumannii 1426993 #IC2#'
'GCF 000810165.3 A. baumannii UH71 278 #IC2#'
'GCF 000809305.3 A. baumannii UH455 61 #IC2#'
'GCF 001433055.1 A. baumannii ABBL050 #IC5#'
'GCF 001674165.1 A. baumannii ABUH739 #IC2#'
'GCF 002014175.1 A. baumannii ABUH426 #IC2#'
'GCF 001414905.1 A. baumannii ABBL067f #IC5#'
'GCF 002212445.1 A. baumannii ABNH8 #IC2#'
'GCF 900117555.1 A. baumannii Ab20 #IC2#'
'GCF 900117675.1 A. baumannii Ab46 #IC2#'
'GCF 000811605.3 A. baumannii UH430 478 #IC2#'
'GCF 000811705.3 A. baumannii UH41 490 #IC2#'
'GCF 900117805.1 A. baumannii Ab13 #IC2#'
'GCF 001666625.1 A. baumannii CCF69 #IC2#'
'GCF 000581375.1 A. baumannii 929679-2095 #IC2#'
'GCF 002951415.1 A. baumannii R4 #IC2#'
'GCF 001666575.1 A. baumannii CCF67 #IC2#'
'GCF 002138215.1 A. baumannii PR392 #IC2#'
'GCF 002250465.1 A. baumannii CCBH15815 #IC5#'
'GCF 002212635.1 A. baumannii AB487 #IC2#'
'GCF 000588915.1 A. baumannii 44895 8 #IC2#'
'GCF 000587915.1 A. baumannii 1294222 #IC2#'
'GCF 002183545.1 A. baumannii ABUH529 #IC2#'
'GCF 000592655.1 A. baumannii 44467 10'
'GCF 000805275.1 A. baumannii 2011BJAB6 #IC2#'
'GCF 000805035.1 A. baumannii 1999BJAB11 #IC2#'
'GCF 001531455.2 A. baumannii ORAB23 #IC2#'
'GCF 001862555.1 A. baumannii XH748 #IC2#'
'GCF 000589615.1 A. baumannii 25253 6 #IC2#'
'GCF 000453525.1 A. baumannii TG27407 #IC2#'
'GCF 000589835.1 A. baumannii 44895 3 #IC2#'
'GCF 002950815.1 A. baumannii R10 #IC2#'
'GCF 000592375.1 A. baumannii 25766 9 #IC2#'
'GCF 001674805.1 A. baumannii ABUH805 #IC2#'
'GCF 000588935.1 A. baumannii 44895 4 #IC2#'
'GCF 001669195.1 A. baumannii ABUH785 #IC2#'
'GCF 002143425.1 A. baumannii ARLG1790 #IC2#'
'GCF 001861775.1 A. baumannii XH829 #IC2#'
'GCF 001665045.1 A. baumannii CCF37 #IC2#'
'GCF 002182805.1 A. baumannii ABUH473 #IC2#'
'GCF 000588975.1 A. baumannii 24845 10 #IC2#'
'GCF 001806425.1 A. baumannii KAB05 #IC2#'
'GCF 000810445.3 A. baumannii UH293 377 #IC2#'
'GCF 000809725.3 A. baumannii UH129 408 #IC2#'
'GCF 002950795.1 A. baumannii S11 #IC2#'
'GCF 000515975.1 A. baumannii UH8807 #IC2#'
'GCF 001861515.1 A. baumannii XH743 #IC2#'
'GCF 002116575.1 A. baumannii 2012046 #IC2#'
'GCF 001414645.1 A. baumannii ABBL060 #IC2#'
'GCF 001666645.1 A. baumannii CCF72 #IC2#'
'GCF 000453345.1 A. baumannii TG27315 #IC2#'
'GCF 001674035.1 A. baumannii ABUH809 #IC2#'
'GCF 900117775.1 A. baumannii Ab23 #IC2#'
'GCF 002184055.1 A. baumannii ABUH586 #IC2#'
'GCF 000805305.1 A. baumannii 2011BJAB2 #IC2#'
'GCF 000588895.1 A. baumannii 44895 9 #IC2#'
'GCF 000591875.1 A. baumannii 44327 5 #IC2#'
'GCF 000722985.1 A. baumannii LY3 #IC2#'
'GCF 002017395.1 A. baumannii ABUH681 #IC2#'
'GCF 001674015.1 A. baumannii ABUH764 #IC2#'
'GCF 000589015.1 A. baumannii 24845 3 #IC2#'
'GCF 000592515.1 A. baumannii 25681 2 #IC2#'
'GCF 001863955.1 A. baumannii XH706 #IC2#'
'GCF 000592315.1 A. baumannii 25766 7 #IC2#'
'GCF 001862975.1 A. baumannii XH797 #IC2#'
'GCF 000308995.1 A. baumannii AB 2008-23-07-01-7 #IC2#'
'GCF 900117665.1 A. baumannii Ab15 #IC2#'

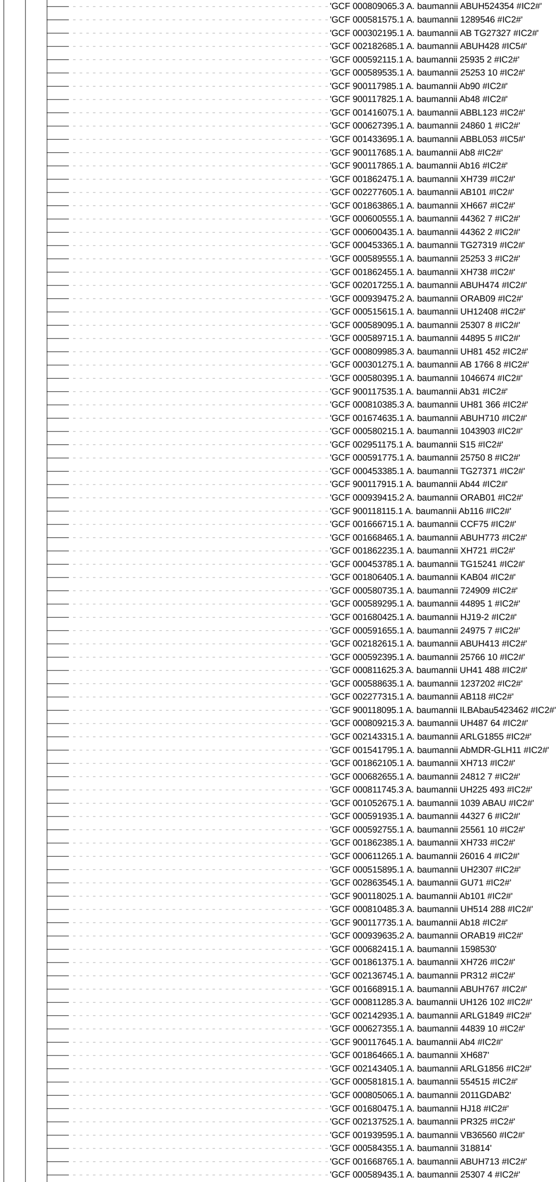

'GCF 000809065.3 A. baumannii ABUH524354 #IC2#'
'GCF 000581575.1 A. baumannii 1289546 #IC2#'
'GCF 000302195.1 A. baumannii AB TG27327 #IC2#'
'GCF 002182685.1 A. baumannii ABUH428 #IC5#'
'GCF 000592115.1 A. baumannii 25935 2 #IC2#'
'GCF 000589535.1 A. baumannii 25253 10 #IC2#'
'GCF 900117985.1 A. baumannii Ab90 #IC2#'
'GCF 900117825.1 A. baumannii Ab48 #IC2#'
'GCF 001416075.1 A. baumannii ABBL123 #IC2#'
'GCF 000627395.1 A. baumannii 24860 1 #IC2#'
'GCF 001433695.1 A. baumannii ABBL053 #IC5#'
'GCF 900117685.1 A. baumannii Ab8 #IC2#'
'GCF 900117865.1 A. baumannii Ab16 #IC2#'
'GCF 001862475.1 A. baumannii XH739 #IC2#'
'GCF 002277605.1 A. baumannii AB101 #IC2#'
'GCF 001863865.1 A. baumannii XH667 #IC2#'
'GCF 000600555.1 A. baumannii 44362 7 #IC2#'
'GCF 000600435.1 A. baumannii 44362 2 #IC2#'
'GCF 000453365.1 A. baumannii TG27319 #IC2#'
'GCF 000589555.1 A. baumannii 25253 3 #IC2#'
'GCF 001862455.1 A. baumannii XH738 #IC2#'
'GCF 002017255.1 A. baumannii ABUH474 #IC2#'
'GCF 000939475.2 A. baumannii ORAB09 #IC2#'
'GCF 000515615.1 A. baumannii UH12408 #IC2#'
'GCF 000589095.1 A. baumannii 25307 8 #IC2#'
'GCF 000589715.1 A. baumannii 44895 5 #IC2#'
'GCF 000809985.3 A. baumannii UH81 452 #IC2#'
'GCF 000301275.1 A. baumannii AB 1766 8 #IC2#'
'GCF 000580395.1 A. baumannii 1046674 #IC2#'
'GCF 900117535.1 A. baumannii Ab31 #IC2#'
'GCF 000810385.3 A. baumannii UH81 366 #IC2#'
'GCF 001674635.1 A. baumannii ABUH710 #IC2#'
'GCF 000580215.1 A. baumannii 1043903 #IC2#'
'GCF 002951175.1 A. baumannii S15 #IC2#'
'GCF 000591775.1 A. baumannii 25750 8 #IC2#'
'GCF 000453385.1 A. baumannii TG27371 #IC2#'
'GCF 900117915.1 A. baumannii Ab44 #IC2#'
'GCF 000939415.2 A. baumannii ORAB01 #IC2#'
'GCF 900118115.1 A. baumannii Ab116 #IC2#'
'GCF 001666715.1 A. baumannii CCF75 #IC2#'
'GCF 001668465.1 A. baumannii ABUH773 #IC2#'
'GCF 001862235.1 A. baumannii XH721 #IC2#'
'GCF 000453785.1 A. baumannii TG15241 #IC2#'
'GCF 001806405.1 A. baumannii KAB04 #IC2#'
'GCF 000580735.1 A. baumannii 724909 #IC2#'
'GCF 000589295.1 A. baumannii 44895 1 #IC2#'
'GCF 001680425.1 A. baumannii HJ19-2 #IC2#'
'GCF 000591655.1 A. baumannii 24975 7 #IC2#'
'GCF 002182615.1 A. baumannii ABUH413 #IC2#'
'GCF 000592395.1 A. baumannii 25766 10 #IC2#'
'GCF 000811625.3 A. baumannii UH41 488 #IC2#'
'GCF 000588635.1 A. baumannii 1237202 #IC2#'
'GCF 002277315.1 A. baumannii AB118 #IC2#'
'GCF 900118095.1 A. baumannii ILBAbau5423462 #IC2#'
'GCF 000809215.3 A. baumannii UH487 64 #IC2#'
'GCF 002143315.1 A. baumannii ARLG1855 #IC2#'
'GCF 001541795.1 A. baumannii AbMDR-GLH11 #IC2#'
'GCF 001862105.1 A. baumannii XH713 #IC2#'
'GCF 000682655.1 A. baumannii 24812 7 #IC2#'
'GCF 000811745.3 A. baumannii UH225 493 #IC2#'
'GCF 001052675.1 A. baumannii 1039 ABAU #IC2#'
'GCF 000591935.1 A. baumannii 44327 6 #IC2#'
'GCF 000592755.1 A. baumannii 25561 10 #IC2#'
'GCF 001862385.1 A. baumannii XH733 #IC2#'
'GCF 000611265.1 A. baumannii 26016 4 #IC2#'
'GCF 000515895.1 A. baumannii UH2307 #IC2#'
'GCF 002863545.1 A. baumannii GU71 #IC2#'
'GCF 900118025.1 A. baumannii Ab101 #IC2#'
'GCF 000810485.3 A. baumannii UH514 288 #IC2#'
'GCF 900117735.1 A. baumannii Ab18 #IC2#'
'GCF 000939635.2 A. baumannii ORAB19 #IC2#'
'GCF 000682415.1 A. baumannii 1598530'
'GCF 001861375.1 A. baumannii XH726 #IC2#'
'GCF 002136745.1 A. baumannii PR312 #IC2#'
'GCF 001668915.1 A. baumannii ABUH767 #IC2#'
'GCF 000811285.3 A. baumannii UH126 102 #IC2#'
'GCF 002142935.1 A. baumannii ARLG1849 #IC2#'
'GCF 000627355.1 A. baumannii 44839 10 #IC2#'
'GCF 900117645.1 A. baumannii Ab4 #IC2#'
'GCF 001864665.1 A. baumannii XH687'
'GCF 002143405.1 A. baumannii ARLG1856 #IC2#'
'GCF 000581815.1 A. baumannii 554515 #IC2#'
'GCF 000805065.1 A. baumannii 2011GDAB2'
'GCF 001680475.1 A. baumannii HJ18 #IC2#'
'GCF 002137525.1 A. baumannii PR325 #IC2#'
'GCF 001939595.1 A. baumannii VB36560 #IC2#'
'GCF 000584355.1 A. baumannii 318814'
'GCF 001668765.1 A. baumannii ABUH713 #IC2#'
'GCF 000589435.1 A. baumannii 25307 4 #IC2#'

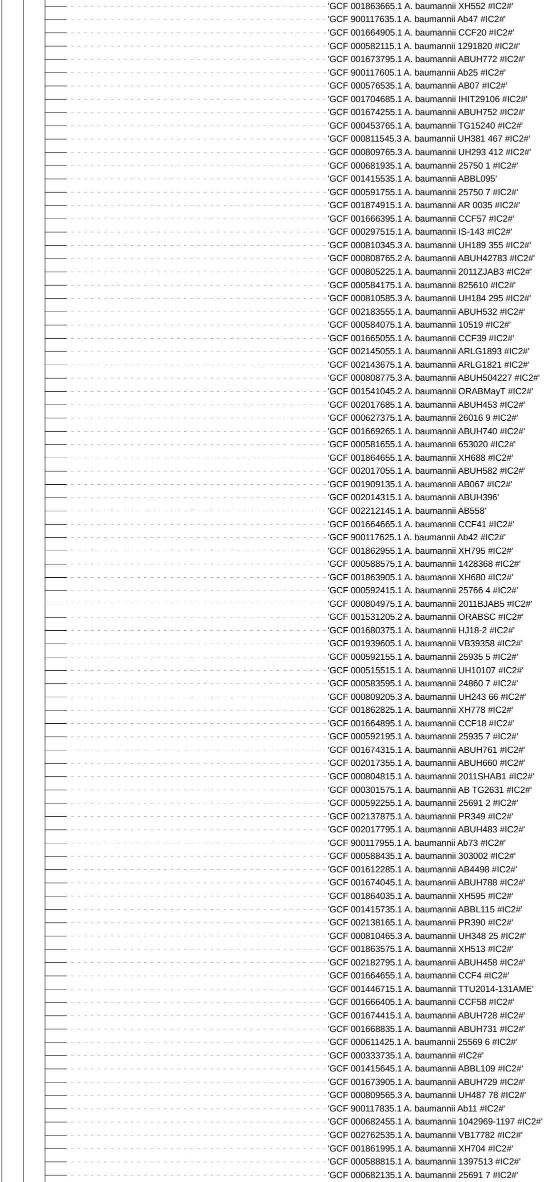
'GCF 001863665.1 A. baumannii XH552 #IC2#'
'GCF 900117635.1 A. baumannii Ab47 #IC2#'
'GCF 001664905.1 A. baumannii CCF20 #IC2#'
'GCF 000582115.1 A. baumannii 1291820 #IC2#'
'GCF 001673795.1 A. baumannii ABUH772 #IC2#'
'GCF 900117605.1 A. baumannii Ab25 #IC2#'
'GCF 000576535.1 A. baumannii AB07 #IC2#'
'GCF 001704685.1 A. baumannii IHIT29106 #IC2#'
'GCF 001674255.1 A. baumannii ABUH752 #IC2#'
'GCF 000453765.1 A. baumannii TG15240 #IC2#'
'GCF 000811545.3 A. baumannii UH381 467 #IC2#'
'GCF 000809765.3 A. baumannii UH293 412 #IC2#'
'GCF 000681935.1 A. baumannii 25750 1 #IC2#'
'GCF 001415535.1 A. baumannii ABBL095'
'GCF 000591755.1 A. baumannii 25750 7 #IC2#'
'GCF 001874915.1 A. baumannii AR 0035 #IC2#'
'GCF 001666395.1 A. baumannii CCF57 #IC2#'
'GCF 000297515.1 A. baumannii IS-143 #IC2#'
'GCF 000810345.3 A. baumannii UH189 355 #IC2#'
'GCF 000808765.2 A. baumannii ABUH42783 #IC2#'
'GCF 000805225.1 A. baumannii 2011ZJAB3 #IC2#'
'GCF 000584175.1 A. baumannii 825610 #IC2#'
'GCF 000810585.3 A. baumannii UH184 295 #IC2#'
'GCF 002183555.1 A. baumannii ABUH532 #IC2#'
'GCF 000584075.1 A. baumannii 10519 #IC2#'
'GCF 001665055.1 A. baumannii CCF39 #IC2#'
'GCF 002145055.1 A. baumannii ARLG1893 #IC2#'
'GCF 002143675.1 A. baumannii ARLG1821 #IC2#'
'GCF 000808775.3 A. baumannii ABUH504227 #IC2#'
'GCF 001541045.2 A. baumannii ORABMayT #IC2#'
'GCF 002017685.1 A. baumannii ABUH453 #IC2#'
'GCF 000627375.1 A. baumannii 26016 9 #IC2#'
'GCF 001669265.1 A. baumannii ABUH740 #IC2#'
'GCF 000581655.1 A. baumannii 653020 #IC2#'
'GCF 001864655.1 A. baumannii XH688 #IC2#'
'GCF 002017055.1 A. baumannii ABUH582 #IC2#'
'GCF 001909135.1 A. baumannii AB067 #IC2#'
'GCF 002014315.1 A. baumannii ABUH396'
'GCF 002212145.1 A. baumannii AB558'
'GCF 001664665.1 A. baumannii CCF41 #IC2#'
'GCF 900117625.1 A. baumannii Ab42 #IC2#'
'GCF 001862955.1 A. baumannii XH795 #IC2#'
'GCF 000588575.1 A. baumannii 1428368 #IC2#'
'GCF 001863905.1 A. baumannii XH680 #IC2#'
'GCF 000592415.1 A. baumannii 25766 4 #IC2#'
'GCF 000804975.1 A. baumannii 2011BJAB5 #IC2#'
'GCF 001531205.2 A. baumannii ORABSC #IC2#'
'GCF 001680375.1 A. baumannii HJ18-2 #IC2#'
'GCF 001939605.1 A. baumannii VB39358 #IC2#'
'GCF 000592155.1 A. baumannii 25935 5 #IC2#'
'GCF 000515515.1 A. baumannii UH10107 #IC2#'
'GCF 000583595.1 A. baumannii 24860 7 #IC2#'
'GCF 000809205.3 A. baumannii UH243 66 #IC2#'
'GCF 001862825.1 A. baumannii XH778 #IC2#'
'GCF 001664895.1 A. baumannii CCF18 #IC2#'
'GCF 000592195.1 A. baumannii 25935 7 #IC2#'
'GCF 001674315.1 A. baumannii ABUH761 #IC2#'
'GCF 002017355.1 A. baumannii ABUH660 #IC2#'
'GCF 000804815.1 A. baumannii 2011SHAB1 #IC2#'
'GCF 000301575.1 A. baumannii AB TG2631 #IC2#'
'GCF 000592255.1 A. baumannii 25691 2 #IC2#'
'GCF 002137875.1 A. baumannii PR349 #IC2#'
'GCF 002017795.1 A. baumannii ABUH483 #IC2#'
'GCF 900117955.1 A. baumannii Ab73 #IC2#'
'GCF 000588435.1 A. baumannii 303002 #IC2#'
'GCF 001612285.1 A. baumannii AB4498 #IC2#'
'GCF 001674045.1 A. baumannii ABUH788 #IC2#'
'GCF 001864035.1 A. baumannii XH595 #IC2#'
'GCF 001415735.1 A. baumannii ABBL115 #IC2#'
'GCF 002138165.1 A. baumannii PR390 #IC2#'
'GCF 000810465.3 A. baumannii UH348 25 #IC2#'
'GCF 001863575.1 A. baumannii XH513 #IC2#'
'GCF 002182795.1 A. baumannii ABUH458 #IC2#'
'GCF 001664655.1 A. baumannii CCF4 #IC2#'
'GCF 001446715.1 A. baumannii TTU2014-131AME'
'GCF 001666405.1 A. baumannii CCF58 #IC2#'
'GCF 001674415.1 A. baumannii ABUH728 #IC2#'
'GCF 001668835.1 A. baumannii ABUH731 #IC2#'
'GCF 000611425.1 A. baumannii 25569 6 #IC2#'
'GCF 000333735.1 A. baumannii #IC2#'
'GCF 001415645.1 A. baumannii ABBL109 #IC2#'
'GCF 001673905.1 A. baumannii ABUH729 #IC2#'
'GCF 000809565.3 A. baumannii UH487 78 #IC2#'
'GCF 900117835.1 A. baumannii Ab11 #IC2#'
'GCF 000682455.1 A. baumannii 1042969-1197 #IC2#'
'GCF 002762535.1 A. baumannii VB17782 #IC2#'
'GCF 001861995.1 A. baumannii XH704 #IC2#'
'GCF 000588815.1 A. baumannii 1397513 #IC2#'
'GCF 000682135.1 A. baumannii 25691 7 #IC2#'

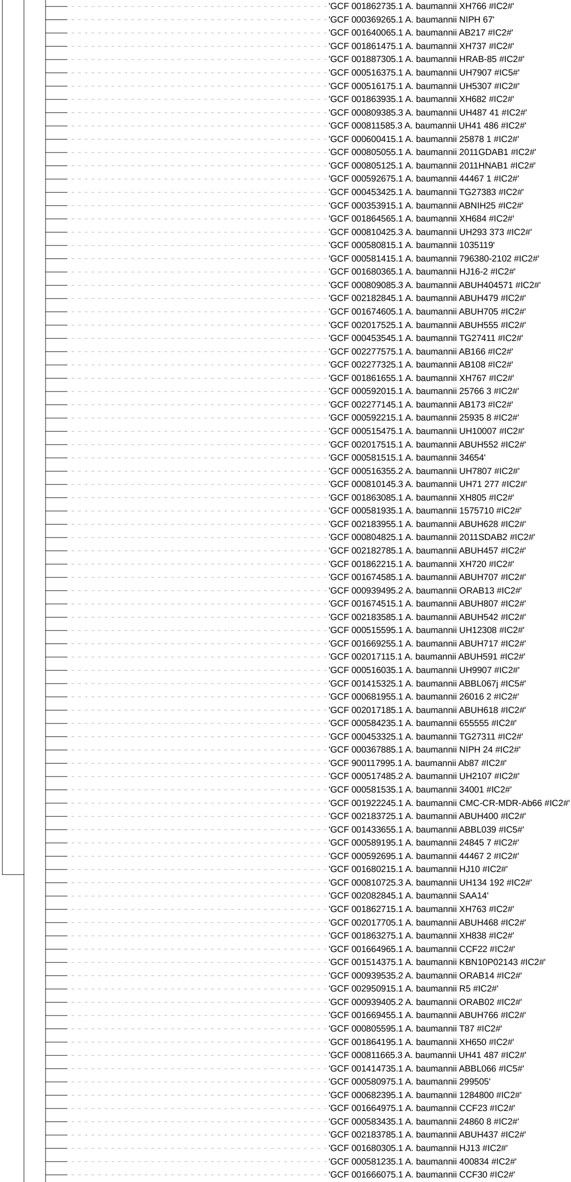
'GCF 001862735.1 A. baumannii XH766 #IC2#'
'GCF 000369265.1 A. baumannii NIPH 67'
'GCF 001640065.1 A. baumannii AB217 #IC2#'
'GCF 001861475.1 A. baumannii XH737 #IC2#'
'GCF 001887305.1 A. baumannii HRAB-85 #IC2#'
'GCF 000516375.1 A. baumannii UH7907 #IC5#'
'GCF 000516175.1 A. baumannii UH5307 #IC2#'
'GCF 001863935.1 A. baumannii XH682 #IC2#'
'GCF 000809385.3 A. baumannii UH487 41 #IC2#'
'GCF 000811585.3 A. baumannii UH41 486 #IC2#'
'GCF 000600415.1 A. baumannii 25878 1 #IC2#'
'GCF 000805055.1 A. baumannii 2011GDAB1 #IC2#'
'GCF 000805125.1 A. baumannii 2011HNAB1 #IC2#'
'GCF 000592675.1 A. baumannii 44467 1 #IC2#'
'GCF 000453425.1 A. baumannii TG27383 #IC2#'
'GCF 000353915.1 A. baumannii ABNIH25 #IC2#'
'GCF 001864565.1 A. baumannii XH684 #IC2#'
'GCF 000810425.3 A. baumannii UH293 373 #IC2#'
'GCF 000580815.1 A. baumannii 1035119'
'GCF 000581415.1 A. baumannii 796380-2102 #IC2#'
'GCF 001680365.1 A. baumannii HJ16-2 #IC2#'
'GCF 000809085.3 A. baumannii ABUH404571 #IC2#'
'GCF 002182845.1 A. baumannii ABUH479 #IC2#'
'GCF 001674605.1 A. baumannii ABUH705 #IC2#'
'GCF 002017525.1 A. baumannii ABUH555 #IC2#'
'GCF 000453545.1 A. baumannii TG27411 #IC2#'
'GCF 002277575.1 A. baumannii AB166 #IC2#'
'GCF 002277325.1 A. baumannii AB108 #IC2#'
'GCF 001861655.1 A. baumannii XH767 #IC2#'
'GCF 000592015.1 A. baumannii 25766 3 #IC2#'
'GCF 002277145.1 A. baumannii AB173 #IC2#'
'GCF 000592215.1 A. baumannii 25935 8 #IC2#'
'GCF 000515475.1 A. baumannii UH10007 #IC2#'
'GCF 002017515.1 A. baumannii ABUH552 #IC2#'
'GCF 000581515.1 A. baumannii 34654'
'GCF 000516355.2 A. baumannii UH7807 #IC2#'
'GCF 000810145.3 A. baumannii UH71 277 #IC2#'
'GCF 001863085.1 A. baumannii XH805 #IC2#'
'GCF 000581935.1 A. baumannii 1575710 #IC2#'
'GCF 002183955.1 A. baumannii ABUH628 #IC2#'
'GCF 000804825.1 A. baumannii 2011SDAB2 #IC2#'
'GCF 002182785.1 A. baumannii ABUH457 #IC2#'
'GCF 001862215.1 A. baumannii XH720 #IC2#'
'GCF 001674585.1 A. baumannii ABUH707 #IC2#'
'GCF 000939495.2 A. baumannii ORAB13 #IC2#'
'GCF 001674515.1 A. baumannii ABUH807 #IC2#'
'GCF 002183585.1 A. baumannii ABUH542 #IC2#'
'GCF 000515595.1 A. baumannii UH12308 #IC2#'
'GCF 001669255.1 A. baumannii ABUH717 #IC2#'
'GCF 002017115.1 A. baumannii ABUH591 #IC2#'
'GCF 000516035.1 A. baumannii UH9907 #IC2#'
'GCF 001415325.1 A. baumannii ABBL067j #IC5#'
'GCF 000681955.1 A. baumannii 26016 2 #IC2#'
'GCF 002017185.1 A. baumannii ABUH618 #IC2#'
'GCF 000584235.1 A. baumannii 655555 #IC2#'
'GCF 000453325.1 A. baumannii TG27311 #IC2#'
'GCF 000367885.1 A. baumannii NIPH 24 #IC2#'
'GCF 900117995.1 A. baumannii Ab87 #IC2#'
'GCF 000517485.2 A. baumannii UH2107 #IC2#'
'GCF 000581535.1 A. baumannii 34001 #IC2#'
'GCF 001922245.1 A. baumannii CMC-CR-MDR-Ab66 #IC2#'
'GCF 002183725.1 A. baumannii ABUH400 #IC2#'
'GCF 001433655.1 A. baumannii ABBL039 #IC5#'
'GCF 000589195.1 A. baumannii 24845 7 #IC2#'
'GCF 000592695.1 A. baumannii 44467 2 #IC2#'
'GCF 001680215.1 A. baumannii HJ10 #IC2#'
'GCF 000810725.3 A. baumannii UH134 192 #IC2#'
'GCF 002082845.1 A. baumannii SAA14'
'GCF 001862715.1 A. baumannii XH763 #IC2#'
'GCF 002017705.1 A. baumannii ABUH468 #IC2#'
'GCF 001863275.1 A. baumannii XH838 #IC2#'
'GCF 001664965.1 A. baumannii CCF22 #IC2#'
'GCF 001514375.1 A. baumannii KBN10P02143 #IC2#'
'GCF 000939535.2 A. baumannii ORAB14 #IC2#'
'GCF 002950915.1 A. baumannii R5 #IC2#'
'GCF 000939405.2 A. baumannii ORAB02 #IC2#'
'GCF 001669455.1 A. baumannii ABUH766 #IC2#'
'GCF 000805595.1 A. baumannii T87 #IC2#'
'GCF 001864195.1 A. baumannii XH650 #IC2#'
'GCF 000811665.3 A. baumannii UH41 487 #IC2#'
'GCF 001414735.1 A. baumannii ABBL066 #IC5#'
'GCF 000580975.1 A. baumannii 299505'
'GCF 000682395.1 A. baumannii 1284800 #IC2#'
'GCF 001664975.1 A. baumannii CCF23 #IC2#'
'GCF 000583435.1 A. baumannii 24860 8 #IC2#'
'GCF 002183785.1 A. baumannii ABUH437 #IC2#'
'GCF 001680305.1 A. baumannii HJ13 #IC2#'
'GCF 000581235.1 A. baumannii 400834 #IC2#'
'GCF 001666075.1 A. baumannii CCF30 #IC2#'

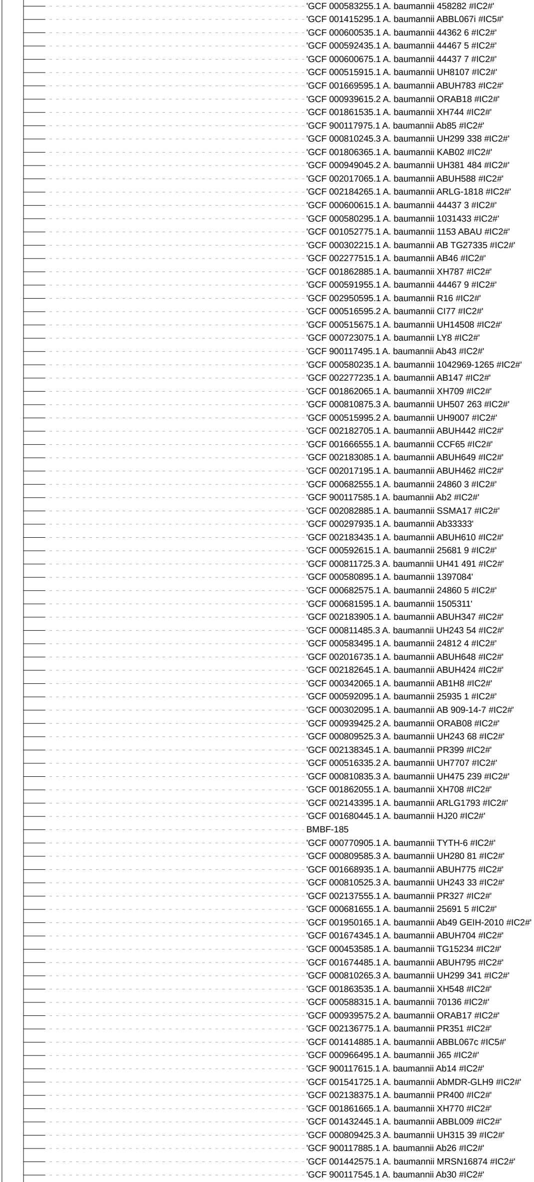
'GCF 000583255.1 A. baumannii 458282 #IC2#'
'GCF 001415295.1 A. baumannii ABBL067i #IC5#'
'GCF 000600535.1 A. baumannii 44362 6 #IC2#'
'GCF 000592435.1 A. baumannii 44467 5 #IC2#'
'GCF 000600675.1 A. baumannii 44437 7 #IC2#'
'GCF 000515915.1 A. baumannii UH8107 #IC2#'
'GCF 001669595.1 A. baumannii ABUH783 #IC2#'
'GCF 000939615.2 A. baumannii ORAB18 #IC2#'
'GCF 001861535.1 A. baumannii XH744 #IC2#'
'GCF 900117975.1 A. baumannii Ab85 #IC2#'
'GCF 000810245.3 A. baumannii UH299 338 #IC2#'
'GCF 001806365.1 A. baumannii KAB02 #IC2#'
'GCF 000949045.2 A. baumannii UH381 484 #IC2#'
'GCF 002017065.1 A. baumannii ABUH588 #IC2#'
'GCF 002184265.1 A. baumannii ARLG-1818 #IC2#'
'GCF 000600615.1 A. baumannii 44437 3 #IC2#'
'GCF 000580295.1 A. baumannii 1031433 #IC2#'
'GCF 001052775.1 A. baumannii 1153 ABAU #IC2#'
'GCF 000302215.1 A. baumannii AB TG27335 #IC2#'
'GCF 002277515.1 A. baumannii AB46 #IC2#'
'GCF 001862885.1 A. baumannii XH787 #IC2#'
'GCF 000591955.1 A. baumannii 44467 9 #IC2#'
'GCF 002950595.1 A. baumannii R16 #IC2#'
'GCF 000516595.2 A. baumannii CI77 #IC2#'
'GCF 000515675.1 A. baumannii UH14508 #IC2#'
'GCF 000723075.1 A. baumannii LY8 #IC2#'
'GCF 900117495.1 A. baumannii Ab43 #IC2#'
'GCF 000580235.1 A. baumannii 1042969-1265 #IC2#'
'GCF 002277235.1 A. baumannii AB147 #IC2#'
'GCF 001862065.1 A. baumannii XH709 #IC2#'
'GCF 000810875.3 A. baumannii UH507 263 #IC2#'
'GCF 000515995.2 A. baumannii UH9007 #IC2#'
'GCF 002182705.1 A. baumannii ABUH442 #IC2#'
'GCF 001666555.1 A. baumannii CCF65 #IC2#'
'GCF 002183085.1 A. baumannii ABUH649 #IC2#'
'GCF 002017195.1 A. baumannii ABUH462 #IC2#'
'GCF 000682555.1 A. baumannii 24860 3 #IC2#'
'GCF 900117585.1 A. baumannii Ab2 #IC2#'
'GCF 002082885.1 A. baumannii SSMA17 #IC2#'
'GCF 000297935.1 A. baumannii Ab33333'
'GCF 002183435.1 A. baumannii ABUH610 #IC2#'
'GCF 000592615.1 A. baumannii 25681 9 #IC2#'
'GCF 000811725.3 A. baumannii UH41 491 #IC2#'
'GCF 000580895.1 A. baumannii 1397084'
'GCF 000682575.1 A. baumannii 24860 5 #IC2#'
'GCF 000681595.1 A. baumannii 1505311'
'GCF 002183905.1 A. baumannii ABUH347 #IC2#'
'GCF 000811485.3 A. baumannii UH243 54 #IC2#'
'GCF 000583495.1 A. baumannii 24812 4 #IC2#'
'GCF 002016735.1 A. baumannii ABUH648 #IC2#'
'GCF 002182645.1 A. baumannii ABUH424 #IC2#'
'GCF 000342065.1 A. baumannii AB1H8 #IC2#'
'GCF 000592095.1 A. baumannii 25935 1 #IC2#'
'GCF 000302095.1 A. baumannii AB 909-14-7 #IC2#'
'GCF 000939425.2 A. baumannii ORAB08 #IC2#'
'GCF 000809525.3 A. baumannii UH243 68 #IC2#'
'GCF 002138345.1 A. baumannii PR399 #IC2#'
'GCF 000516335.2 A. baumannii UH7707 #IC2#'
'GCF 000810835.3 A. baumannii UH475 239 #IC2#'
'GCF 001862055.1 A. baumannii XH708 #IC2#'
'GCF 002143395.1 A. baumannii ARLG1793 #IC2#'
'GCF 001680445.1 A. baumannii HJ20 #IC2#'
BMBF-185
'GCF 000770905.1 A. baumannii TYTH-6 #IC2#'
'GCF 000809585.3 A. baumannii UH280 81 #IC2#'
'GCF 001668935.1 A. baumannii ABUH775 #IC2#'
'GCF 000810525.3 A. baumannii UH243 33 #IC2#'
'GCF 002137555.1 A. baumannii PR327 #IC2#'
'GCF 000681655.1 A. baumannii 25691 5 #IC2#'
'GCF 001950165.1 A. baumannii Ab49 GEIH-2010 #IC2#'
'GCF 001674345.1 A. baumannii ABUH704 #IC2#'
'GCF 000453585.1 A. baumannii TG15234 #IC2#'
'GCF 001674485.1 A. baumannii ABUH795 #IC2#'
'GCF 000810265.3 A. baumannii UH299 341 #IC2#'
'GCF 001863535.1 A. baumannii XH548 #IC2#'
'GCF 000588315.1 A. baumannii 70136 #IC2#'
'GCF 000939575.2 A. baumannii ORAB17 #IC2#'
'GCF 002136775.1 A. baumannii PR351 #IC2#'
'GCF 001414885.1 A. baumannii ABBL067c #IC5#'
'GCF 000966495.1 A. baumannii J65 #IC2#'
'GCF 900117615.1 A. baumannii Ab14 #IC2#'
'GCF 001541725.1 A. baumannii AbMDR-GLH9 #IC2#'
'GCF 002138375.1 A. baumannii PR400 #IC2#'
'GCF 001861665.1 A. baumannii XH770 #IC2#'
'GCF 001432445.1 A. baumannii ABBL009 #IC2#'
'GCF 000809425.3 A. baumannii UH315 39 #IC2#'
'GCF 900117885.1 A. baumannii Ab26 #IC2#'
'GCF 001442575.1 A. baumannii MRSN16874 #IC2#'
'GCF 900117545.1 A. baumannii Ab30 #IC2#'

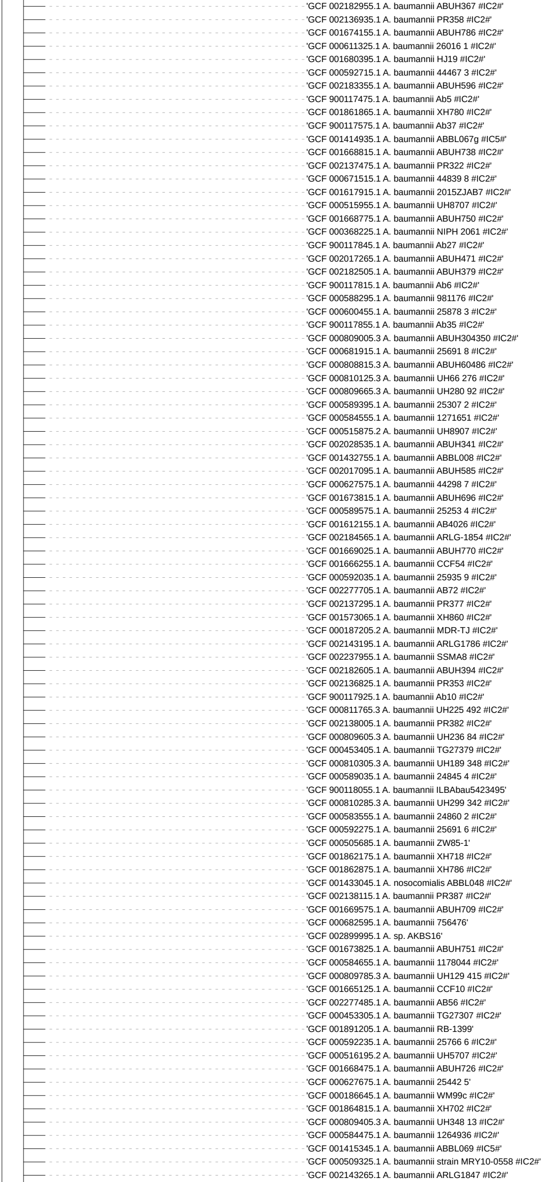
'GCF 002182955.1 A. baumannii ABUH367 #IC2#'
'GCF 002136935.1 A. baumannii PR358 #IC2#'
'GCF 001674155.1 A. baumannii ABUH786 #IC2#'
'GCF 000611325.1 A. baumannii 26016 1 #IC2#'
'GCF 001680395.1 A. baumannii HJ19 #IC2#'
'GCF 000592715.1 A. baumannii 44467 3 #IC2#'
'GCF 002183355.1 A. baumannii ABUH596 #IC2#'
'GCF 900117475.1 A. baumannii Ab5 #IC2#'
'GCF 001861865.1 A. baumannii XH780 #IC2#'
'GCF 900117575.1 A. baumannii Ab37 #IC2#'
'GCF 001414935.1 A. baumannii ABBL067g #IC5#'
'GCF 001668815.1 A. baumannii ABUH738 #IC2#'
'GCF 002137475.1 A. baumannii PR322 #IC2#'
'GCF 000671515.1 A. baumannii 44839 8 #IC2#'
'GCF 001617915.1 A. baumannii 2015ZJAB7 #IC2#'
'GCF 000515955.1 A. baumannii UH8707 #IC2#'
'GCF 001668775.1 A. baumannii ABUH750 #IC2#'
'GCF 000368225.1 A. baumannii NIPH 2061 #IC2#'
'GCF 900117845.1 A. baumannii Ab27 #IC2#'
'GCF 002017265.1 A. baumannii ABUH471 #IC2#'
'GCF 002182505.1 A. baumannii ABUH379 #IC2#'
'GCF 900117815.1 A. baumannii Ab6 #IC2#'
'GCF 000588295.1 A. baumannii 981176 #IC2#'
'GCF 000600455.1 A. baumannii 25878 3 #IC2#'
'GCF 900117855.1 A. baumannii Ab35 #IC2#'
'GCF 000809005.3 A. baumannii ABUH304350 #IC2#'
'GCF 000681915.1 A. baumannii 25691 8 #IC2#'
'GCF 000808815.3 A. baumannii ABUH60486 #IC2#'
'GCF 000810125.3 A. baumannii UH66 276 #IC2#'
'GCF 000809665.3 A. baumannii UH280 92 #IC2#'
'GCF 000589395.1 A. baumannii 25307 2 #IC2#'
'GCF 000584555.1 A. baumannii 1271651 #IC2#'
'GCF 000515875.2 A. baumannii UH8907 #IC2#'
'GCF 002028535.1 A. baumannii ABUH341 #IC2#'
'GCF 001432755.1 A. baumannii ABBL008 #IC2#'
'GCF 002017095.1 A. baumannii ABUH585 #IC2#'
'GCF 000627575.1 A. baumannii 44298 7 #IC2#'
'GCF 001673815.1 A. baumannii ABUH696 #IC2#'
'GCF 000589575.1 A. baumannii 25253 4 #IC2#'
'GCF 001612155.1 A. baumannii AB4026 #IC2#'
'GCF 002184565.1 A. baumannii ARLG-1854 #IC2#'
'GCF 001669025.1 A. baumannii ABUH770 #IC2#'
'GCF 001666255.1 A. baumannii CCF54 #IC2#'
'GCF 000592035.1 A. baumannii 25935 9 #IC2#'
'GCF 002277705.1 A. baumannii AB72 #IC2#'
'GCF 002137295.1 A. baumannii PR377 #IC2#'
'GCF 001573065.1 A. baumannii XH860 #IC2#'
'GCF 000187205.2 A. baumannii MDR-TJ #IC2#'
'GCF 002143195.1 A. baumannii ARLG1786 #IC2#'
'GCF 002237955.1 A. baumannii SSMA8 #IC2#'
'GCF 002182605.1 A. baumannii ABUH394 #IC2#'
'GCF 002136825.1 A. baumannii PR353 #IC2#'
'GCF 900117925.1 A. baumannii Ab10 #IC2#'
'GCF 000811765.3 A. baumannii UH225 492 #IC2#'
'GCF 002138005.1 A. baumannii PR382 #IC2#'
'GCF 000809605.3 A. baumannii UH236 84 #IC2#'
'GCF 000453405.1 A. baumannii TG27379 #IC2#'
'GCF 000810305.3 A. baumannii UH189 348 #IC2#'
'GCF 000589035.1 A. baumannii 24845 4 #IC2#'
'GCF 900118055.1 A. baumannii ILBAbau5423495'
'GCF 000810285.3 A. baumannii UH299 342 #IC2#'
'GCF 000583555.1 A. baumannii 24860 2 #IC2#'
'GCF 000592275.1 A. baumannii 25691 6 #IC2#'
'GCF 000505685.1 A. baumannii ZW85-1'
'GCF 001862175.1 A. baumannii XH718 #IC2#'
'GCF 001862875.1 A. baumannii XH786 #IC2#'
'GCF 001433045.1 A. nosocomialis ABBL048 #IC2#'
'GCF 002138115.1 A. baumannii PR387 #IC2#'
'GCF 001669575.1 A. baumannii ABUH709 #IC2#'
'GCF 000682595.1 A. baumannii 756476'
'GCF 002899995.1 A. sp. AKBS16'
'GCF 001673825.1 A. baumannii ABUH751 #IC2#'
'GCF 000584655.1 A. baumannii 1178044 #IC2#'
'GCF 000809785.3 A. baumannii UH129 415 #IC2#'
'GCF 001665125.1 A. baumannii CCF10 #IC2#'
'GCF 002277485.1 A. baumannii AB56 #IC2#'
'GCF 000453305.1 A. baumannii TG27307 #IC2#'
'GCF 001891205.1 A. baumannii RB-1399'
'GCF 000592235.1 A. baumannii 25766 6 #IC2#'
'GCF 000516195.2 A. baumannii UH5707 #IC2#'
'GCF 001668475.1 A. baumannii ABUH726 #IC2#'
'GCF 000627675.1 A. baumannii 25442 5'
'GCF 000186645.1 A. baumannii WM99c #IC2#'
'GCF 001864815.1 A. baumannii XH702 #IC2#'
'GCF 000809405.3 A. baumannii UH348 13 #IC2#'
'GCF 000584475.1 A. baumannii 1264936 #IC2#'
'GCF 001415345.1 A. baumannii ABBL069 #IC5#'
'GCF 000509325.1 A. baumannii strain MRY10-0558 #IC2#'
'GCF 002143265.1 A. baumannii ARLG1847 #IC2#'

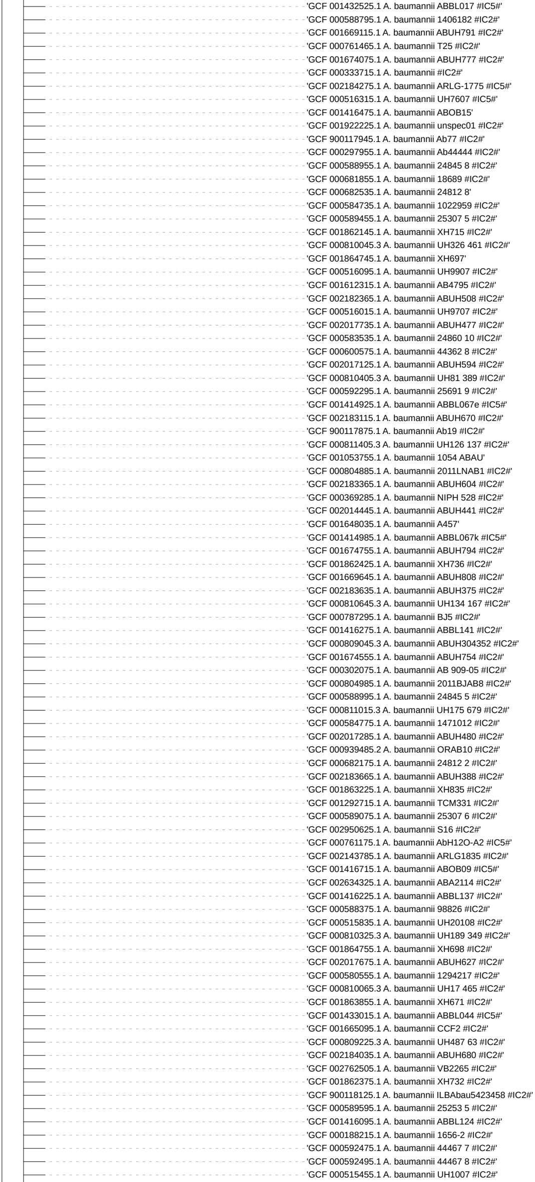
'GCF 001432525.1 A. baumannii ABBL017 #IC5#'
'GCF 000588795.1 A. baumannii 1406182 #IC2#'
'GCF 001669115.1 A. baumannii ABUH791 #IC2#'
'GCF 000761465.1 A. baumannii T25 #IC2#'
'GCF 001674075.1 A. baumannii ABUH777 #IC2#'
'GCF 000333715.1 A. baumannii #IC2#'
'GCF 002184275.1 A. baumannii ARLG-1775 #IC5#'
'GCF 000516315.1 A. baumannii UH7607 #IC5#'
'GCF 001416475.1 A. baumannii ABOB15'
'GCF 001922225.1 A. baumannii unspec01 #IC2#'
'GCF 900117945.1 A. baumannii Ab77 #IC2#'
'GCF 000297955.1 A. baumannii Ab44444 #IC2#'
'GCF 000588955.1 A. baumannii 24845 8 #IC2#'
'GCF 000681855.1 A. baumannii 18689 #IC2#'
'GCF 000682535.1 A. baumannii 24812 8'
'GCF 000584735.1 A. baumannii 1022959 #IC2#'
'GCF 000589455.1 A. baumannii 25307 5 #IC2#'
'GCF 001862145.1 A. baumannii XH715 #IC2#'
'GCF 000810045.3 A. baumannii UH326 461 #IC2#'
'GCF 001864745.1 A. baumannii XH697'
'GCF 000516095.1 A. baumannii UH9907 #IC2#'
'GCF 001612315.1 A. baumannii AB4795 #IC2#'
'GCF 002182365.1 A. baumannii ABUH508 #IC2#'
'GCF 000516015.1 A. baumannii UH9707 #IC2#'
'GCF 002017735.1 A. baumannii ABUH477 #IC2#'
'GCF 000583535.1 A. baumannii 24860 10 #IC2#'
'GCF 000600575.1 A. baumannii 44362 8 #IC2#'
'GCF 002017125.1 A. baumannii ABUH594 #IC2#'
'GCF 000810405.3 A. baumannii UH81 389 #IC2#'
'GCF 000592295.1 A. baumannii 25691 9 #IC2#'
'GCF 001414925.1 A. baumannii ABBL067e #IC5#'
'GCF 002183115.1 A. baumannii ABUH670 #IC2#'
'GCF 900117875.1 A. baumannii Ab19 #IC2#'
'GCF 000811405.3 A. baumannii UH126 137 #IC2#'
'GCF 001053755.1 A. baumannii 1054 ABAU'
'GCF 000804885.1 A. baumannii 2011LNAB1 #IC2#'
'GCF 002183365.1 A. baumannii ABUH604 #IC2#'
'GCF 000369285.1 A. baumannii NIPH 528 #IC2#'
'GCF 002014445.1 A. baumannii ABUH441 #IC2#'
'GCF 001648035.1 A. baumannii A457'
'GCF 001414985.1 A. baumannii ABBL067k #IC5#'
'GCF 001674755.1 A. baumannii ABUH794 #IC2#'
'GCF 001862425.1 A. baumannii XH736 #IC2#'
'GCF 001669645.1 A. baumannii ABUH808 #IC2#'
'GCF 002183635.1 A. baumannii ABUH375 #IC2#'
'GCF 000810645.3 A. baumannii UH134 167 #IC2#'
'GCF 000787295.1 A. baumannii BJ5 #IC2#'
'GCF 001416275.1 A. baumannii ABBL141 #IC2#'
'GCF 000809045.3 A. baumannii ABUH304352 #IC2#'
'GCF 001674555.1 A. baumannii ABUH754 #IC2#'
'GCF 000302075.1 A. baumannii AB 909-05 #IC2#'
'GCF 000804985.1 A. baumannii 2011BJAB8 #IC2#'
'GCF 000588995.1 A. baumannii 24845 5 #IC2#'
'GCF 000811015.3 A. baumannii UH175 679 #IC2#'
'GCF 000584775.1 A. baumannii 1471012 #IC2#'
'GCF 002017285.1 A. baumannii ABUH480 #IC2#'
'GCF 000939485.2 A. baumannii ORAB10 #IC2#'
'GCF 000682175.1 A. baumannii 24812 2 #IC2#'
'GCF 002183665.1 A. baumannii ABUH388 #IC2#'
'GCF 001863225.1 A. baumannii XH835 #IC2#'
'GCF 001292715.1 A. baumannii TCM331 #IC2#'
'GCF 000589075.1 A. baumannii 25307 6 #IC2#'
'GCF 002950625.1 A. baumannii S16 #IC2#'
'GCF 000761175.1 A. baumannii AbH12O-A2 #IC5#'
'GCF 002143785.1 A. baumannii ARLG1835 #IC2#'
'GCF 001416715.1 A. baumannii ABOB09 #IC5#'
'GCF 002634325.1 A. baumannii ABA2114 #IC2#'
'GCF 001416225.1 A. baumannii ABBL137 #IC2#'
'GCF 000588375.1 A. baumannii 98826 #IC2#'
'GCF 000515835.1 A. baumannii UH20108 #IC2#'
'GCF 000810325.3 A. baumannii UH189 349 #IC2#'
'GCF 001864755.1 A. baumannii XH698 #IC2#'
'GCF 002017675.1 A. baumannii ABUH627 #IC2#'
'GCF 000580555.1 A. baumannii 1294217 #IC2#'
'GCF 000810065.3 A. baumannii UH17 465 #IC2#'
'GCF 001863855.1 A. baumannii XH671 #IC2#'
'GCF 001433015.1 A. baumannii ABBL044 #IC5#'
'GCF 001665095.1 A. baumannii CCF2 #IC2#'
'GCF 000809225.3 A. baumannii UH487 63 #IC2#'
'GCF 002184035.1 A. baumannii ABUH680 #IC2#'
'GCF 002762505.1 A. baumannii VB2265 #IC2#'
'GCF 001862375.1 A. baumannii XH732 #IC2#'
'GCF 900118125.1 A. baumannii ILBAbau5423458 #IC2#'
'GCF 000589595.1 A. baumannii 25253 5 #IC2#'
'GCF 001416095.1 A. baumannii ABBL124 #IC2#'
'GCF 000188215.1 A. baumannii 1656-2 #IC2#'
'GCF 000592475.1 A. baumannii 44467 7 #IC2#'
'GCF 000592495.1 A. baumannii 44467 8 #IC2#'
'GCF 000515455.1 A. baumannii UH1007 #IC2#'

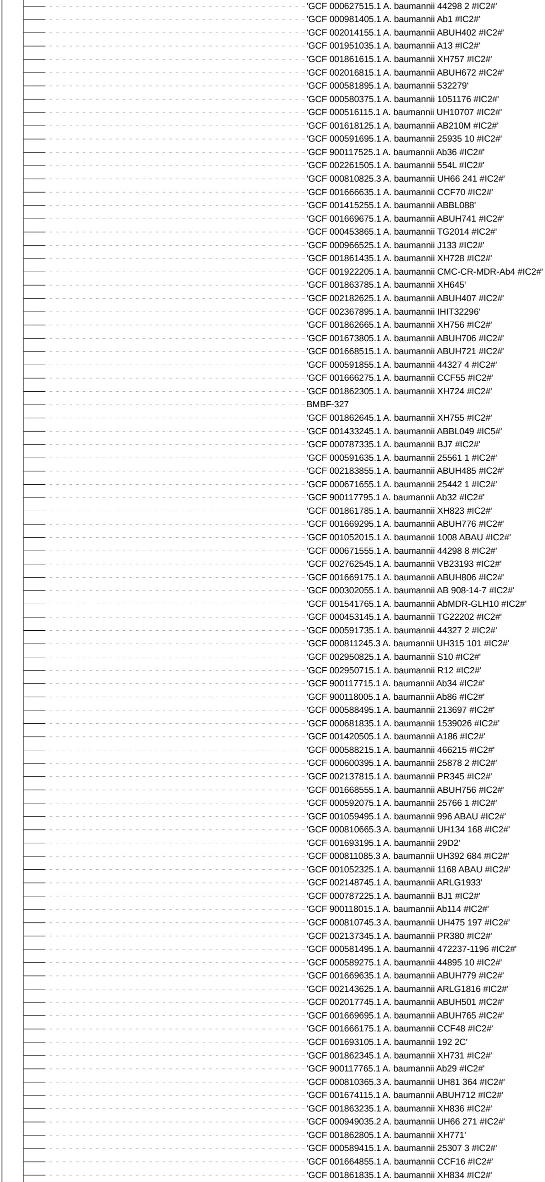

'GCF 000627515.1 A. baumannii 44298 2 #IC2#'
'GCF 000981405.1 A. baumannii Ab1 #IC2#'
'GCF 002014155.1 A. baumannii ABUH402 #IC2#'
'GCF 001951035.1 A. baumannii A13 #IC2#'
'GCF 001861615.1 A. baumannii XH757 #IC2#'
'GCF 002016815.1 A. baumannii ABUH672 #IC2#'
'GCF 000581895.1 A. baumannii 532279'
'GCF 000580375.1 A. baumannii 1051176 #IC2#'
'GCF 000516115.1 A. baumannii UH10707 #IC2#'
'GCF 001618125.1 A. baumannii AB210M #IC2#'
'GCF 000591695.1 A. baumannii 25935 10 #IC2#'
'GCF 900117525.1 A. baumannii Ab36 #IC2#'
'GCF 002261505.1 A. baumannii 554L #IC2#'
'GCF 000810825.3 A. baumannii UH66 241 #IC2#'
'GCF 001666635.1 A. baumannii CCF70 #IC2#'
'GCF 001415255.1 A. baumannii ABBL088'
'GCF 001669675.1 A. baumannii ABUH741 #IC2#'
'GCF 000453865.1 A. baumannii TG2014 #IC2#'
'GCF 000966525.1 A. baumannii J133 #IC2#'
'GCF 001861435.1 A. baumannii XH728 #IC2#'
'GCF 001922205.1 A. baumannii CMC-CR-MDR-Ab4 #IC2#'
'GCF 001863785.1 A. baumannii XH645'
'GCF 002182625.1 A. baumannii ABUH407 #IC2#'
'GCF 002367895.1 A. baumannii IHIT32296'
'GCF 001862665.1 A. baumannii XH756 #IC2#'
'GCF 001673805.1 A. baumannii ABUH706 #IC2#'
'GCF 001668515.1 A. baumannii ABUH721 #IC2#'
'GCF 000591855.1 A. baumannii 44327 4 #IC2#'
'GCF 001666275.1 A. baumannii CCF55 #IC2#'
'GCF 001862305.1 A. baumannii XH724 #IC2#'
BMBF-327
'GCF 001862645.1 A. baumannii XH755 #IC2#'
'GCF 001433245.1 A. baumannii ABBL049 #IC5#'
'GCF 000787335.1 A. baumannii BJ7 #IC2#'
'GCF 000591635.1 A. baumannii 25561 1 #IC2#'
'GCF 002183855.1 A. baumannii ABUH485 #IC2#'
'GCF 000671655.1 A. baumannii 25442 1 #IC2#'
'GCF 900117795.1 A. baumannii Ab32 #IC2#'
'GCF 001861785.1 A. baumannii XH823 #IC2#'
'GCF 001669295.1 A. baumannii ABUH776 #IC2#'
'GCF 001052015.1 A. baumannii 1008 ABAU #IC2#'
'GCF 000671555.1 A. baumannii 44298 8 #IC2#'
'GCF 002762545.1 A. baumannii VB23193 #IC2#'
'GCF 001669175.1 A. baumannii ABUH806 #IC2#'
'GCF 000302055.1 A. baumannii AB 908-14-7 #IC2#'
'GCF 001541765.1 A. baumannii AbMDR-GLH10 #IC2#'
'GCF 000453145.1 A. baumannii TG22202 #IC2#'
'GCF 000591735.1 A. baumannii 44327 2 #IC2#'
'GCF 000811245.3 A. baumannii UH315 101 #IC2#'
'GCF 002950825.1 A. baumannii S10 #IC2#'
'GCF 002950715.1 A. baumannii R12 #IC2#'
'GCF 900117715.1 A. baumannii Ab34 #IC2#'
'GCF 900118005.1 A. baumannii Ab86 #IC2#'
'GCF 000588495.1 A. baumannii 213697 #IC2#'
'GCF 000681835.1 A. baumannii 1539026 #IC2#'
'GCF 001420505.1 A. baumannii A186 #IC2#'
'GCF 000588215.1 A. baumannii 466215 #IC2#'
'GCF 000600395.1 A. baumannii 25878 2 #IC2#'
'GCF 002137815.1 A. baumannii PR345 #IC2#'
'GCF 001668555.1 A. baumannii ABUH756 #IC2#'
'GCF 000592075.1 A. baumannii 25766 1 #IC2#'
'GCF 001059495.1 A. baumannii 996 ABAU #IC2#'
'GCF 000810665.3 A. baumannii UH134 168 #IC2#'
'GCF 001693195.1 A. baumannii 29D2'
'GCF 000811085.3 A. baumannii UH392 684 #IC2#'
'GCF 001052325.1 A. baumannii 1168 ABAU #IC2#'
'GCF 002148745.1 A. baumannii ARLG1933'
'GCF 000787225.1 A. baumannii BJ1 #IC2#'
'GCF 900118015.1 A. baumannii Ab114 #IC2#'
'GCF 000810745.3 A. baumannii UH475 197 #IC2#'
'GCF 002137345.1 A. baumannii PR380 #IC2#'
'GCF 000581495.1 A. baumannii 472237-1196 #IC2#'
'GCF 000589275.1 A. baumannii 44895 10 #IC2#'
'GCF 001669635.1 A. baumannii ABUH779 #IC2#'
'GCF 002143625.1 A. baumannii ARLG1816 #IC2#'
'GCF 002017745.1 A. baumannii ABUH501 #IC2#'
'GCF 001669695.1 A. baumannii ABUH765 #IC2#'
'GCF 001666175.1 A. baumannii CCF48 #IC2#'
'GCF 001693105.1 A. baumannii 192 2C'
'GCF 001862345.1 A. baumannii XH731 #IC2#'
'GCF 900117765.1 A. baumannii Ab29 #IC2#'
'GCF 000810365.3 A. baumannii UH81 364 #IC2#'
'GCF 001674115.1 A. baumannii ABUH712 #IC2#'
'GCF 001863235.1 A. baumannii XH836 #IC2#'
'GCF 000949035.2 A. baumannii UH66 271 #IC2#'
'GCF 001862805.1 A. baumannii XH771'
'GCF 000589415.1 A. baumannii 25307 3 #IC2#'
'GCF 001664855.1 A. baumannii CCF16 #IC2#'
'GCF 001861835.1 A. baumannii XH834 #IC2#'

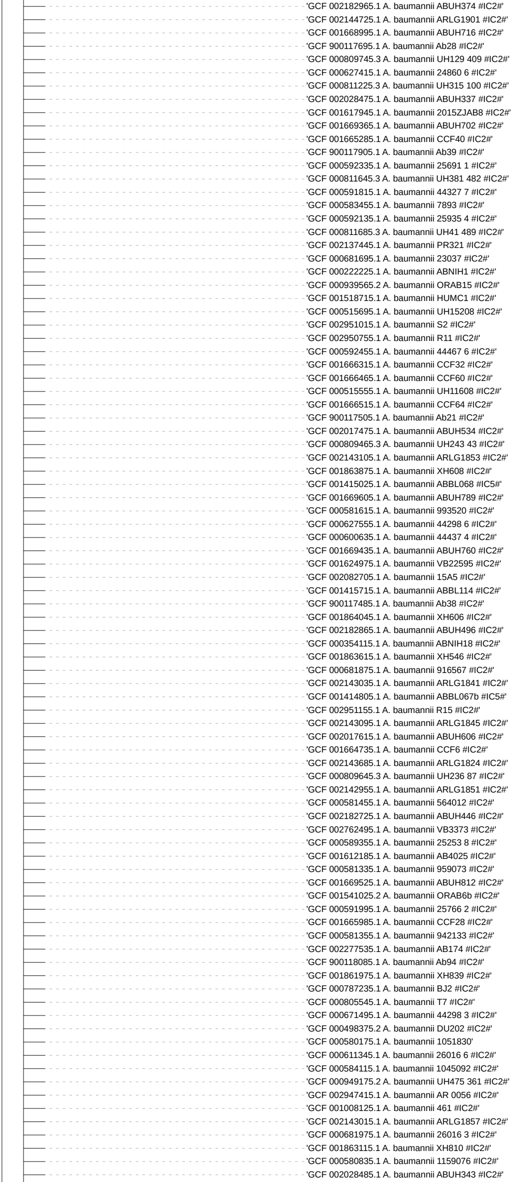
'GCF 002182965.1 A. baumannii ABUH374 #IC2#'
'GCF 002144725.1 A. baumannii ARLG1901 #IC2#'
'GCF 001668995.1 A. baumannii ABUH716 #IC2#'
'GCF 900117695.1 A. baumannii Ab28 #IC2#'
'GCF 000809745.3 A. baumannii UH129 409 #IC2#'
'GCF 000627415.1 A. baumannii 24860 6 #IC2#'
'GCF 000811225.3 A. baumannii UH315 100 #IC2#'
'GCF 002028475.1 A. baumannii ABUH337 #IC2#'
'GCF 001617945.1 A. baumannii 2015ZJAB8 #IC2#'
'GCF 001669365.1 A. baumannii ABUH702 #IC2#'
'GCF 001665285.1 A. baumannii CCF40 #IC2#'
'GCF 900117905.1 A. baumannii Ab39 #IC2#'
'GCF 000592335.1 A. baumannii 25691 1 #IC2#'
'GCF 000811645.3 A. baumannii UH381 482 #IC2#'
'GCF 000591815.1 A. baumannii 44327 7 #IC2#'
'GCF 000583455.1 A. baumannii 7893 #IC2#'
'GCF 000592135.1 A. baumannii 25935 4 #IC2#'
'GCF 000811685.3 A. baumannii UH41 489 #IC2#'
'GCF 002137445.1 A. baumannii PR321 #IC2#'
'GCF 000681695.1 A. baumannii 23037 #IC2#'
'GCF 000222225.1 A. baumannii ABNIH1 #IC2#'
'GCF 000939565.2 A. baumannii ORAB15 #IC2#'
'GCF 001518715.1 A. baumannii HUMC1 #IC2#'
'GCF 000515695.1 A. baumannii UH15208 #IC2#'
'GCF 002951015.1 A. baumannii S2 #IC2#'
'GCF 002950755.1 A. baumannii R11 #IC2#'
'GCF 000592455.1 A. baumannii 44467 6 #IC2#'
'GCF 001666315.1 A. baumannii CCF32 #IC2#'
'GCF 001666465.1 A. baumannii CCF60 #IC2#'
'GCF 000515555.1 A. baumannii UH11608 #IC2#'
'GCF 001666515.1 A. baumannii CCF64 #IC2#'
'GCF 900117505.1 A. baumannii Ab21 #IC2#'
'GCF 002017475.1 A. baumannii ABUH534 #IC2#'
'GCF 000809465.3 A. baumannii UH243 43 #IC2#'
'GCF 002143105.1 A. baumannii ARLG1853 #IC2#'
'GCF 001863875.1 A. baumannii XH608 #IC2#'
'GCF 001415025.1 A. baumannii ABBL068 #IC5#'
'GCF 001669605.1 A. baumannii ABUH789 #IC2#'
'GCF 000581615.1 A. baumannii 993520 #IC2#'
'GCF 000627555.1 A. baumannii 44298 6 #IC2#'
'GCF 000600635.1 A. baumannii 44437 4 #IC2#'
'GCF 001669435.1 A. baumannii ABUH760 #IC2#'
'GCF 001624975.1 A. baumannii VB22595 #IC2#'
'GCF 002082705.1 A. baumannii 15A5 #IC2#'
'GCF 001415715.1 A. baumannii ABBL114 #IC2#'
'GCF 900117485.1 A. baumannii Ab38 #IC2#'
'GCF 001864045.1 A. baumannii XH606 #IC2#'
'GCF 002182865.1 A. baumannii ABUH496 #IC2#'
'GCF 000354115.1 A. baumannii ABNIH18 #IC2#'
'GCF 001863615.1 A. baumannii XH546 #IC2#'
'GCF 000681875.1 A. baumannii 916567 #IC2#'
'GCF 002143035.1 A. baumannii ARLG1841 #IC2#'
'GCF 001414805.1 A. baumannii ABBL067b #IC5#'
'GCF 002951155.1 A. baumannii R15 #IC2#'
'GCF 002143095.1 A. baumannii ARLG1845 #IC2#'
'GCF 002017615.1 A. baumannii ABUH606 #IC2#'
'GCF 001664735.1 A. baumannii CCF6 #IC2#'
'GCF 002143685.1 A. baumannii ARLG1824 #IC2#'
'GCF 000809645.3 A. baumannii UH236 87 #IC2#'
'GCF 002142955.1 A. baumannii ARLG1851 #IC2#'
'GCF 000581455.1 A. baumannii 564012 #IC2#'
'GCF 002182725.1 A. baumannii ABUH446 #IC2#'
'GCF 002762495.1 A. baumannii VB3373 #IC2#'
'GCF 000589355.1 A. baumannii 25253 8 #IC2#'
'GCF 001612185.1 A. baumannii AB4025 #IC2#'
'GCF 000581335.1 A. baumannii 959073 #IC2#'
'GCF 001669525.1 A. baumannii ABUH812 #IC2#'
'GCF 001541025.2 A. baumannii ORAB6b #IC2#'
'GCF 000591995.1 A. baumannii 25766 2 #IC2#'
'GCF 001665985.1 A. baumannii CCF28 #IC2#'
'GCF 000581355.1 A. baumannii 942133 #IC2#'
'GCF 002277535.1 A. baumannii AB174 #IC2#'
'GCF 900118085.1 A. baumannii Ab94 #IC2#'
'GCF 001861975.1 A. baumannii XH839 #IC2#'
'GCF 000787235.1 A. baumannii BJ2 #IC2#'
'GCF 000805545.1 A. baumannii T7 #IC2#'
'GCF 000671495.1 A. baumannii 44298 3 #IC2#'
'GCF 000498375.2 A. baumannii DU202 #IC2#'
'GCF 000580175.1 A. baumannii 1051830'
'GCF 000611345.1 A. baumannii 26016 6 #IC2#'
'GCF 000584115.1 A. baumannii 1045092 #IC2#'
'GCF 000949175.2 A. baumannii UH475 361 #IC2#'
'GCF 002947415.1 A. baumannii AR 0056 #IC2#'
'GCF 001008125.1 A. baumannii 461 #IC2#'
'GCF 002143015.1 A. baumannii ARLG1857 #IC2#'
'GCF 000681975.1 A. baumannii 26016 3 #IC2#'
'GCF 001863115.1 A. baumannii XH810 #IC2#'
'GCF 000580835.1 A. baumannii 1159076 #IC2#'
'GCF 002028485.1 A. baumannii ABUH343 #IC2#'

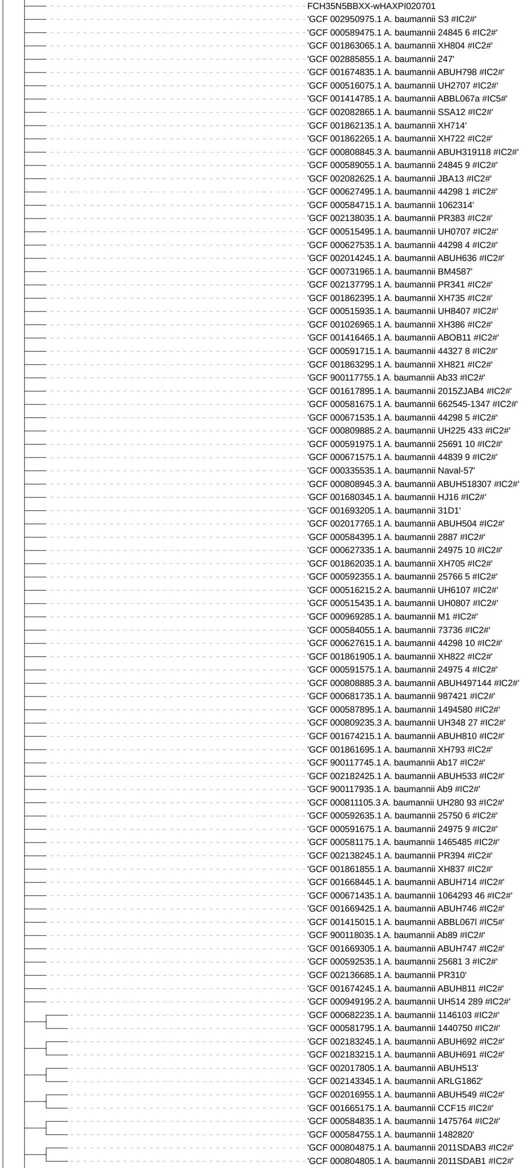
FCH35N5BBXX-wHAXPI020701
'GCF 002950975.1 A. baumannii S3 #IC2#'
'GCF 000589475.1 A. baumannii 24845 6 #IC2#'
'GCF 001863065.1 A. baumannii XH804 #IC2#'
'GCF 002885855.1 A. baumannii 247'
'GCF 001674835.1 A. baumannii ABUH798 #IC2#'
'GCF 000516075.1 A. baumannii UH2707 #IC2#'
'GCF 001414785.1 A. baumannii ABBL067a #IC5#'
'GCF 002082865.1 A. baumannii SSA12 #IC2#'
'GCF 001862135.1 A. baumannii XH714'
'GCF 001862265.1 A. baumannii XH722 #IC2#'
'GCF 000808845.3 A. baumannii ABUH319118 #IC2#'
'GCF 000589055.1 A. baumannii 24845 9 #IC2#'
'GCF 002082625.1 A. baumannii JBA13 #IC2#'
'GCF 000627495.1 A. baumannii 44298 1 #IC2#'
'GCF 000584715.1 A. baumannii 1062314'
'GCF 002138035.1 A. baumannii PR383 #IC2#'
'GCF 000515495.1 A. baumannii UH0707 #IC2#'
'GCF 000627535.1 A. baumannii 44298 4 #IC2#'
'GCF 002014245.1 A. baumannii ABUH636 #IC2#'
'GCF 000731965.1 A. baumannii BM4587'
'GCF 002137795.1 A. baumannii PR341 #IC2#'
'GCF 001862395.1 A. baumannii XH735 #IC2#'
'GCF 000515935.1 A. baumannii UH8407 #IC2#'
'GCF 001026965.1 A. baumannii XH386 #IC2#'
'GCF 001416465.1 A. baumannii ABOB11 #IC2#'
'GCF 000591715.1 A. baumannii 44327 8 #IC2#'
'GCF 001863295.1 A. baumannii XH821 #IC2#'
'GCF 900117755.1 A. baumannii Ab33 #IC2#'
'GCF 001617895.1 A. baumannii 2015ZJAB4 #IC2#'
'GCF 000581675.1 A. baumannii 662545-1347 #IC2#'
'GCF 000671535.1 A. baumannii 44298 5 #IC2#'
'GCF 000809885.2 A. baumannii UH225 433 #IC2#'
'GCF 000591975.1 A. baumannii 25691 10 #IC2#'
'GCF 000671575.1 A. baumannii 44839 9 #IC2#'
'GCF 000335535.1 A. baumannii Naval-57'
'GCF 000808945.3 A. baumannii ABUH518307 #IC2#'
'GCF 001680345.1 A. baumannii HJ16 #IC2#'
'GCF 001693205.1 A. baumannii 31D1'
'GCF 002017765.1 A. baumannii ABUH504 #IC2#'
'GCF 000584395.1 A. baumannii 2887 #IC2#'
'GCF 000627335.1 A. baumannii 24975 10 #IC2#'
'GCF 001862035.1 A. baumannii XH705 #IC2#'
'GCF 000592355.1 A. baumannii 25766 5 #IC2#'
'GCF 000516215.2 A. baumannii UH6107 #IC2#'
'GCF 000515435.1 A. baumannii UH0807 #IC2#'
'GCF 000969285.1 A. baumannii M1 #IC2#'
'GCF 000584055.1 A. baumannii 73736 #IC2#'
'GCF 000627615.1 A. baumannii 44298 10 #IC2#'
'GCF 001861905.1 A. baumannii XH822 #IC2#'
'GCF 000591575.1 A. baumannii 24975 4 #IC2#'
'GCF 000808885.3 A. baumannii ABUH497144 #IC2#'
'GCF 000681735.1 A. baumannii 987421 #IC2#'
'GCF 000587895.1 A. baumannii 1494580 #IC2#'
'GCF 000809235.3 A. baumannii UH348 27 #IC2#'
'GCF 001674215.1 A. baumannii ABUH810 #IC2#'
'GCF 001861695.1 A. baumannii XH793 #IC2#'
'GCF 900117745.1 A. baumannii Ab17 #IC2#'
'GCF 002182425.1 A. baumannii ABUH533 #IC2#'
'GCF 900117935.1 A. baumannii Ab9 #IC2#'
'GCF 000811105.3 A. baumannii UH280 93 #IC2#'
'GCF 000592635.1 A. baumannii 25750 6 #IC2#'
'GCF 000591675.1 A. baumannii 24975 9 #IC2#'
'GCF 000581175.1 A. baumannii 1465485 #IC2#'
'GCF 002138245.1 A. baumannii PR394 #IC2#'
'GCF 001861855.1 A. baumannii XH837 #IC2#'
'GCF 001668445.1 A. baumannii ABUH714 #IC2#'
'GCF 000671435.1 A. baumannii 1064293 46 #IC2#'
'GCF 001669425.1 A. baumannii ABUH746 #IC2#'
'GCF 001415015.1 A. baumannii ABBL067l #IC5#'
'GCF 900118035.1 A. baumannii Ab89 #IC2#'
'GCF 001669305.1 A. baumannii ABUH747 #IC2#'
'GCF 000592535.1 A. baumannii 25681 3 #IC2#'
'GCF 002136685.1 A. baumannii PR310'
'GCF 001674245.1 A. baumannii ABUH811 #IC2#'
'GCF 000949195.2 A. baumannii UH514 289 #IC2#'
'GCF 000682235.1 A. baumannii 1146103 #IC2#'
'GCF 000581795.1 A. baumannii 1440750 #IC2#'
'GCF 002183245.1 A. baumannii ABUH692 #IC2#'
'GCF 002183215.1 A. baumannii ABUH691 #IC2#'
'GCF 002017805.1 A. baumannii ABUH513'
'GCF 002143345.1 A. baumannii ARLG1862'
'GCF 002016955.1 A. baumannii ABUH549 #IC2#'
'GCF 001665175.1 A. baumannii CCF15 #IC2#'
'GCF 000584835.1 A. baumannii 1475764 #IC2#'
'GCF 000584755.1 A. baumannii 1482820'
'GCF 000804875.1 A. baumannii 2011SDAB3 #IC2#'
'GCF 000804805.1 A. baumannii 2011SDAB1 #IC2#'

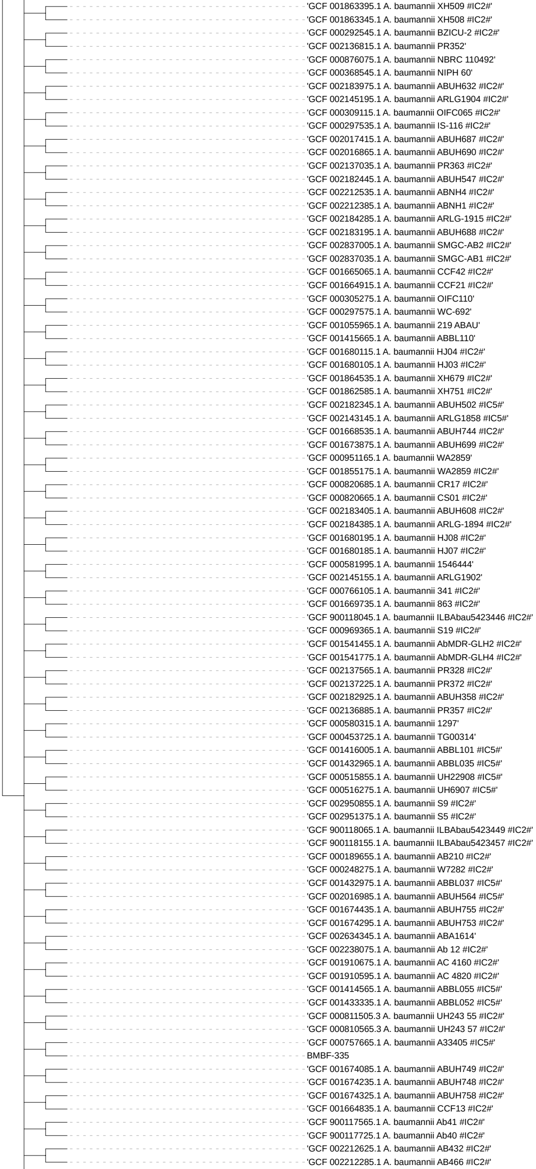

'GCF 001863395.1 A. baumannii XH509 #IC2#'
'GCF 001863345.1 A. baumannii XH508 #IC2#'
'GCF 000292545.1 A. baumannii BZICU-2 #IC2#'
'GCF 002136815.1 A. baumannii PR352'
'GCF 000876075.1 A. baumannii NBRC 110492'
'GCF 000368545.1 A. baumannii NIPH 60'
'GCF 002183975.1 A. baumannii ABUH632 #IC2#'
'GCF 002145195.1 A. baumannii ARLG1904 #IC2#'
'GCF 000309115.1 A. baumannii OIFC065 #IC2#'
'GCF 000297535.1 A. baumannii IS-116 #IC2#'
'GCF 002017415.1 A. baumannii ABUH687 #IC2#'
'GCF 002016865.1 A. baumannii ABUH690 #IC2#'
'GCF 002137035.1 A. baumannii PR363 #IC2#'
'GCF 002182445.1 A. baumannii ABUH547 #IC2#'
'GCF 002212535.1 A. baumannii ABNH4 #IC2#'
'GCF 002212385.1 A. baumannii ABNH1 #IC2#'
'GCF 002184285.1 A. baumannii ARLG-1915 #IC2#'
'GCF 002183195.1 A. baumannii ABUH688 #IC2#'
'GCF 002837005.1 A. baumannii SMGC-AB2 #IC2#'
'GCF 002837035.1 A. baumannii SMGC-AB1 #IC2#'
'GCF 001665065.1 A. baumannii CCF42 #IC2#'
'GCF 001664915.1 A. baumannii CCF21 #IC2#'
'GCF 000305275.1 A. baumannii OIFC110'
'GCF 000297575.1 A. baumannii WC-692'
'GCF 001055965.1 A. baumannii 219 ABAU'
'GCF 001415665.1 A. baumannii ABBL110'
'GCF 001680115.1 A. baumannii HJ04 #IC2#'
'GCF 001680105.1 A. baumannii HJ03 #IC2#'
'GCF 001864535.1 A. baumannii XH679 #IC2#'
'GCF 001862585.1 A. baumannii XH751 #IC2#'
'GCF 002182345.1 A. baumannii ABUH502 #IC5#'
'GCF 002143145.1 A. baumannii ARLG1858 #IC5#'
'GCF 001668535.1 A. baumannii ABUH744 #IC2#'
'GCF 001673875.1 A. baumannii ABUH699 #IC2#'
'GCF 000951165.1 A. baumannii WA2859'
'GCF 001855175.1 A. baumannii WA2859 #IC2#'
'GCF 000820685.1 A. baumannii CR17 #IC2#'
'GCF 000820665.1 A. baumannii CS01 #IC2#'
'GCF 002183405.1 A. baumannii ABUH608 #IC2#'
'GCF 002184385.1 A. baumannii ARLG-1894 #IC2#'
'GCF 001680195.1 A. baumannii HJ08 #IC2#'
'GCF 001680185.1 A. baumannii HJ07 #IC2#'
'GCF 000581995.1 A. baumannii 1546444'
'GCF 002145155.1 A. baumannii ARLG1902'
'GCF 000766105.1 A. baumannii 341 #IC2#'
'GCF 001669735.1 A. baumannii 863 #IC2#'
'GCF 900118045.1 A. baumannii ILBAbau5423446 #IC2#'
'GCF 000969365.1 A. baumannii S19 #IC2#'
'GCF 001541455.1 A. baumannii AbMDR-GLH2 #IC2#'
'GCF 001541775.1 A. baumannii AbMDR-GLH4 #IC2#'
'GCF 002137565.1 A. baumannii PR328 #IC2#'
'GCF 002137225.1 A. baumannii PR372 #IC2#'
'GCF 002182925.1 A. baumannii ABUH358 #IC2#'
'GCF 002136885.1 A. baumannii PR357 #IC2#'
'GCF 000580315.1 A. baumannii 1297'
'GCF 000453725.1 A. baumannii TG00314'
'GCF 001416005.1 A. baumannii ABBL101 #IC5#'
'GCF 001432965.1 A. baumannii ABBL035 #IC5#'
'GCF 000515855.1 A. baumannii UH22908 #IC5#'
'GCF 000516275.1 A. baumannii UH6907 #IC5#'
'GCF 002950855.1 A. baumannii S9 #IC2#'
'GCF 002951375.1 A. baumannii S5 #IC2#'
'GCF 900118065.1 A. baumannii ILBAbau5423449 #IC2#'
'GCF 900118155.1 A. baumannii ILBAbau5423457 #IC2#'
'GCF 000189655.1 A. baumannii AB210 #IC2#'
'GCF 000248275.1 A. baumannii W7282 #IC2#'
'GCF 001432975.1 A. baumannii ABBL037 #IC5#'
'GCF 002016985.1 A. baumannii ABUH564 #IC5#'
'GCF 001674435.1 A. baumannii ABUH755 #IC2#'
'GCF 001674295.1 A. baumannii ABUH753 #IC2#'
'GCF 002634345.1 A. baumannii ABA1614'
'GCF 002238075.1 A. baumannii Ab 12 #IC2#'
'GCF 001910675.1 A. baumannii AC 4160 #IC2#'
'GCF 001910595.1 A. baumannii AC 4820 #IC2#'
'GCF 001414565.1 A. baumannii ABBL055 #IC5#'
'GCF 001433335.1 A. baumannii ABBL052 #IC5#'
'GCF 000811505.3 A. baumannii UH243 55 #IC2#'
'GCF 000810565.3 A. baumannii UH243 57 #IC2#'
'GCF 000757665.1 A. baumannii A33405 #IC5#'
BMBF-335
'GCF 001674085.1 A. baumannii ABUH749 #IC2#'
'GCF 001674235.1 A. baumannii ABUH748 #IC2#'
'GCF 001674325.1 A. baumannii ABUH758 #IC2#'
'GCF 001664835.1 A. baumannii CCF13 #IC2#'
'GCF 900117565.1 A. baumannii Ab41 #IC2#'
'GCF 900117725.1 A. baumannii Ab40 #IC2#'
'GCF 002212625.1 A. baumannii AB432 #IC2#'
'GCF 002212285.1 A. baumannii AB466 #IC2#'

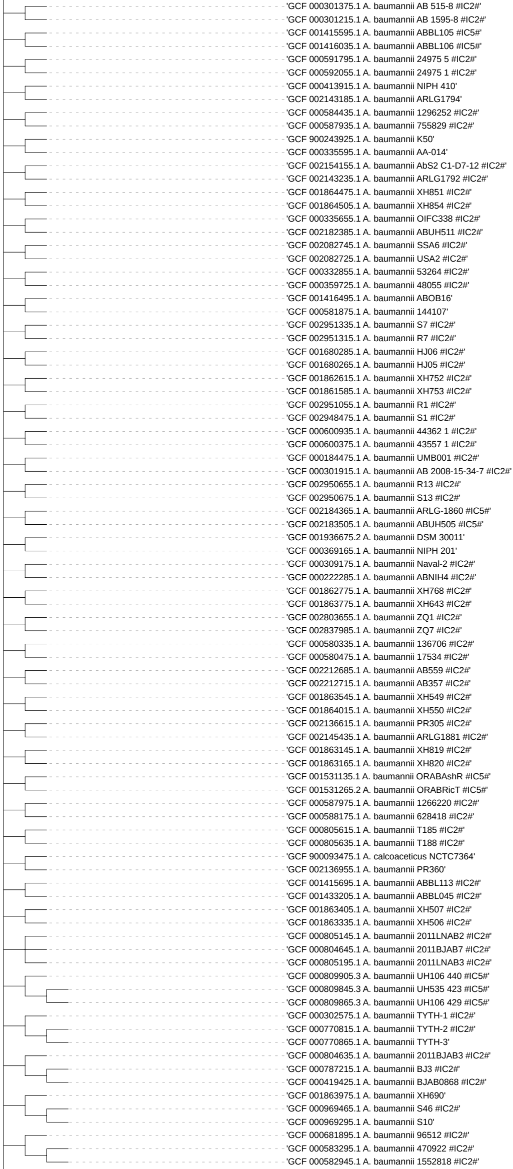

'GCF 000301375.1 A. baumannii AB 515-8 #IC2#'
'GCF 000301215.1 A. baumannii AB 1595-8 #IC2#'
'GCF 001415595.1 A. baumannii ABBL105 #IC5#'
'GCF 001416035.1 A. baumannii ABBL106 #IC5#'
'GCF 000591795.1 A. baumannii 24975 5 #IC2#'
'GCF 000592055.1 A. baumannii 24975 1 #IC2#'
'GCF 000413915.1 A. baumannii NIPH 410'
'GCF 002143185.1 A. baumannii ARLG1794'
'GCF 000584435.1 A. baumannii 1296252 #IC2#'
'GCF 000587935.1 A. baumannii 755829 #IC2#'
'GCF 900243925.1 A. baumannii K50'
'GCF 000335595.1 A. baumannii AA-014'
'GCF 002154155.1 A. baumannii AbS2 C1-D7-12 #IC2#'
'GCF 002143235.1 A. baumannii ARLG1792 #IC2#'
'GCF 001864475.1 A. baumannii XH851 #IC2#'
'GCF 001864505.1 A. baumannii XH854 #IC2#'
'GCF 000335655.1 A. baumannii OIFC338 #IC2#'
'GCF 002182385.1 A. baumannii ABUH511 #IC2#'
'GCF 002082745.1 A. baumannii SSA6 #IC2#'
'GCF 002082725.1 A. baumannii USA2 #IC2#'
'GCF 000332855.1 A. baumannii 53264 #IC2#'
'GCF 000359725.1 A. baumannii 48055 #IC2#'
'GCF 001416495.1 A. baumannii ABOB16'
'GCF 000581875.1 A. baumannii 144107'
'GCF 002951335.1 A. baumannii S7 #IC2#'
'GCF 002951315.1 A. baumannii R7 #IC2#'
'GCF 001680285.1 A. baumannii HJ06 #IC2#'
'GCF 001680265.1 A. baumannii HJ05 #IC2#'
'GCF 001862615.1 A. baumannii XH752 #IC2#'
'GCF 001861585.1 A. baumannii XH753 #IC2#'
'GCF 002951055.1 A. baumannii R1 #IC2#'
'GCF 002948475.1 A. baumannii S1 #IC2#'
'GCF 000600935.1 A. baumannii 44362 1 #IC2#'
'GCF 000600375.1 A. baumannii 43557 1 #IC2#'
'GCF 000184475.1 A. baumannii UMB001 #IC2#'
'GCF 000301915.1 A. baumannii AB 2008-15-34-7 #IC2#'
'GCF 002950655.1 A. baumannii R13 #IC2#'
'GCF 002950675.1 A. baumannii S13 #IC2#'
'GCF 002184365.1 A. baumannii ARLG-1860 #IC5#'
'GCF 002183505.1 A. baumannii ABUH505 #IC5#'
'GCF 001936675.2 A. baumannii DSM 30011'
'GCF 000369165.1 A. baumannii NIPH 201'
'GCF 000309175.1 A. baumannii Naval-2 #IC2#'
'GCF 000222285.1 A. baumannii ABNIH4 #IC2#'
'GCF 001862775.1 A. baumannii XH768 #IC2#'
'GCF 001863775.1 A. baumannii XH643 #IC2#'
'GCF 002803655.1 A. baumannii ZQ1 #IC2#'
'GCF 002837985.1 A. baumannii ZQ7 #IC2#'
'GCF 000580335.1 A. baumannii 136706 #IC2#'
'GCF 000580475.1 A. baumannii 17534 #IC2#'
'GCF 002212685.1 A. baumannii AB559 #IC2#'
'GCF 002212715.1 A. baumannii AB357 #IC2#'
'GCF 001863545.1 A. baumannii XH549 #IC2#'
'GCF 001864015.1 A. baumannii XH550 #IC2#'
'GCF 002136615.1 A. baumannii PR305 #IC2#'
'GCF 002145435.1 A. baumannii ARLG1881 #IC2#'
'GCF 001863145.1 A. baumannii XH819 #IC2#'
'GCF 001863165.1 A. baumannii XH820 #IC2#'
'GCF 001531135.1 A. baumannii ORABAshR #IC5#'
'GCF 001531265.2 A. baumannii ORABRicT #IC5#'
'GCF 000587975.1 A. baumannii 1266220 #IC2#'
'GCF 000588175.1 A. baumannii 628418 #IC2#'
'GCF 000805615.1 A. baumannii T185 #IC2#'
'GCF 000805635.1 A. baumannii T188 #IC2#'
'GCF 900093475.1 A. calcoaceticus NCTC7364'
'GCF 002136955.1 A. baumannii PR360'
'GCF 001415695.1 A. baumannii ABBL113 #IC2#'
'GCF 001433205.1 A. baumannii ABBL045 #IC2#'
'GCF 001863405.1 A. baumannii XH507 #IC2#'
'GCF 001863335.1 A. baumannii XH506 #IC2#'
'GCF 000805145.1 A. baumannii 2011LNAB2 #IC2#'
'GCF 000804645.1 A. baumannii 2011BJAB7 #IC2#'
'GCF 000805195.1 A. baumannii 2011LNAB3 #IC2#'
'GCF 000809905.3 A. baumannii UH106 440 #IC5#'
'GCF 000809845.3 A. baumannii UH535 423 #IC5#'
'GCF 000809865.3 A. baumannii UH106 429 #IC5#'
'GCF 000302575.1 A. baumannii TYTH-1 #IC2#'
'GCF 000770815.1 A. baumannii TYTH-2 #IC2#'
'GCF 000770865.1 A. baumannii TYTH-3'
'GCF 000804635.1 A. baumannii 2011BJAB3 #IC2#'
'GCF 000787215.1 A. baumannii BJ3 #IC2#'
'GCF 000419425.1 A. baumannii BJAB0868 #IC2#'
'GCF 001863975.1 A. baumannii XH690'
'GCF 000969465.1 A. baumannii S46 #IC2#'
'GCF 000969295.1 A. baumannii S10'
'GCF 000681895.1 A. baumannii 96512 #IC2#'
'GCF 000583295.1 A. baumannii 470922 #IC2#'
'GCF 000582945.1 A. baumannii 1552818 #IC2#'

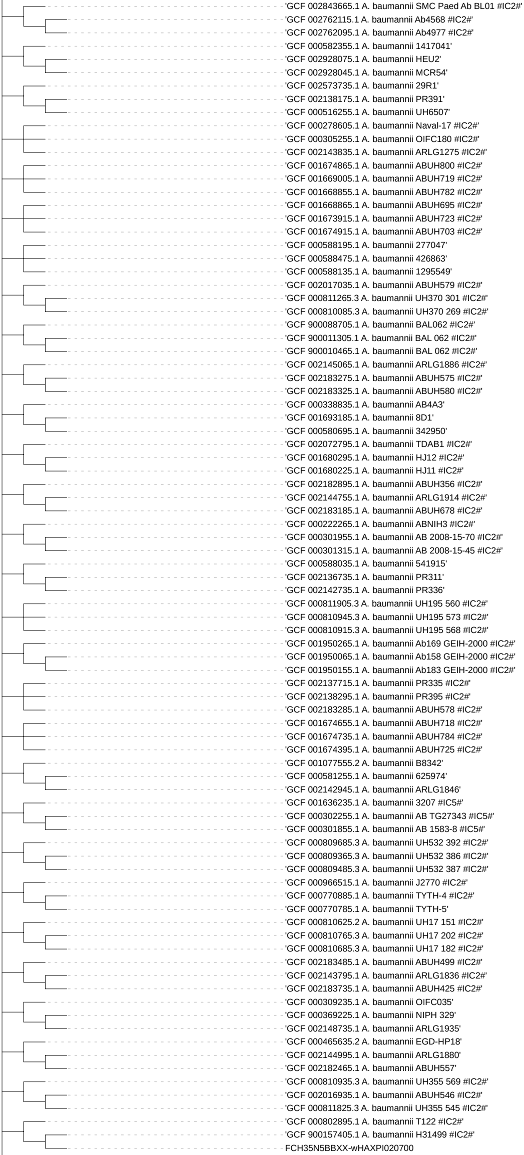
'GCF 002843665.1 A. baumannii SMC Paed Ab BL01 #IC2#'
'GCF 002762115.1 A. baumannii Ab4568 #IC2#'
'GCF 002762095.1 A. baumannii Ab4977 #IC2#'
'GCF 000582355.1 A. baumannii 1417041'
'GCF 002928075.1 A. baumannii HEU2'
'GCF 002928045.1 A. baumannii MCR54'
'GCF 002573735.1 A. baumannii 29R1'
'GCF 002138175.1 A. baumannii PR391'
'GCF 000516255.1 A. baumannii UH6507'
'GCF 000278605.1 A. baumannii Naval-17 #IC2#'
'GCF 000305255.1 A. baumannii OIFC180 #IC2#'
'GCF 002143835.1 A. baumannii ARLG1275 #IC2#'
'GCF 001674865.1 A. baumannii ABUH800 #IC2#'
'GCF 001669005.1 A. baumannii ABUH719 #IC2#'
'GCF 001668855.1 A. baumannii ABUH782 #IC2#'
'GCF 001668865.1 A. baumannii ABUH695 #IC2#'
'GCF 001673915.1 A. baumannii ABUH723 #IC2#'
'GCF 001674915.1 A. baumannii ABUH703 #IC2#'
'GCF 000588195.1 A. baumannii 277047'
'GCF 000588475.1 A. baumannii 426863'
'GCF 000588135.1 A. baumannii 1295549'
'GCF 002017035.1 A. baumannii ABUH579 #IC2#'
'GCF 000811265.3 A. baumannii UH370 301 #IC2#'
'GCF 000810085.3 A. baumannii UH370 269 #IC2#'
'GCF 900088705.1 A. baumannii BAL062 #IC2#'
'GCF 900011305.1 A. baumannii BAL 062 #IC2#'
'GCF 900010465.1 A. baumannii BAL 062 #IC2#'
'GCF 002145065.1 A. baumannii ARLG1886 #IC2#'
'GCF 002183275.1 A. baumannii ABUH575 #IC2#'
'GCF 002183325.1 A. baumannii ABUH580 #IC2#'
'GCF 000338835.1 A. baumannii AB4A3'
'GCF 001693185.1 A. baumannii 8D1'
'GCF 000580695.1 A. baumannii 342950'
'GCF 002072795.1 A. baumannii TDAB1 #IC2#'
'GCF 001680295.1 A. baumannii HJ12 #IC2#'
'GCF 001680225.1 A. baumannii HJ11 #IC2#'
'GCF 002182895.1 A. baumannii ABUH356 #IC2#'
'GCF 002144755.1 A. baumannii ARLG1914 #IC2#'
'GCF 002183185.1 A. baumannii ABUH678 #IC2#'
'GCF 000222265.1 A. baumannii ABNIH3 #IC2#'
'GCF 000301955.1 A. baumannii AB 2008-15-70 #IC2#'
'GCF 000301315.1 A. baumannii AB 2008-15-45 #IC2#'
'GCF 000588035.1 A. baumannii 541915'
'GCF 002136735.1 A. baumannii PR311'
'GCF 002142735.1 A. baumannii PR336'
'GCF 000811905.3 A. baumannii UH195 560 #IC2#'
'GCF 000810945.3 A. baumannii UH195 573 #IC2#'
'GCF 000810915.3 A. baumannii UH195 568 #IC2#'
'GCF 001950265.1 A. baumannii Ab169 GEIH-2000 #IC2#'
'GCF 001950065.1 A. baumannii Ab158 GEIH-2000 #IC2#'
'GCF 001950155.1 A. baumannii Ab183 GEIH-2000 #IC2#'
'GCF 002137715.1 A. baumannii PR335 #IC2#'
'GCF 002138295.1 A. baumannii PR395 #IC2#'
'GCF 002183285.1 A. baumannii ABUH578 #IC2#'
'GCF 001674655.1 A. baumannii ABUH718 #IC2#'
'GCF 001674735.1 A. baumannii ABUH784 #IC2#'
'GCF 001674395.1 A. baumannii ABUH725 #IC2#'
'GCF 001077555.2 A. baumannii B8342'
'GCF 000581255.1 A. baumannii 625974'
'GCF 002142945.1 A. baumannii ARLG1846'
'GCF 001636235.1 A. baumannii 3207 #IC5#'
'GCF 000302255.1 A. baumannii AB TG27343 #IC5#'
'GCF 000301855.1 A. baumannii AB 1583-8 #IC5#'
'GCF 000809685.3 A. baumannii UH532 392 #IC2#'
'GCF 000809365.3 A. baumannii UH532 386 #IC2#'
'GCF 000809485.3 A. baumannii UH532 387 #IC2#'
'GCF 000966515.1 A. baumannii J2770 #IC2#'
'GCF 000770885.1 A. baumannii TYTH-4 #IC2#'
'GCF 000770785.1 A. baumannii TYTH-5'
'GCF 000810625.2 A. baumannii UH17 151 #IC2#'
'GCF 000810765.3 A. baumannii UH17 202 #IC2#'
'GCF 000810685.3 A. baumannii UH17 182 #IC2#'
'GCF 002183485.1 A. baumannii ABUH499 #IC2#'
'GCF 002143795.1 A. baumannii ARLG1836 #IC2#'
'GCF 002183735.1 A. baumannii ABUH425 #IC2#'
'GCF 000309235.1 A. baumannii OIFC035'
'GCF 000369225.1 A. baumannii NIPH 329'
'GCF 002148735.1 A. baumannii ARLG1935'
'GCF 000465635.2 A. baumannii EGD-HP18'
'GCF 002144995.1 A. baumannii ARLG1880'
'GCF 002182465.1 A. baumannii ABUH557'
'GCF 000810935.3 A. baumannii UH355 569 #IC2#'
'GCF 002016935.1 A. baumannii ABUH546 #IC2#'
'GCF 000811825.3 A. baumannii UH355 545 #IC2#'
'GCF 000802895.1 A. baumannii T122 #IC2#'
'GCF 900157405.1 A. baumannii H31499 #IC2#'
FCH35N5BBXX-wHAXPI020700

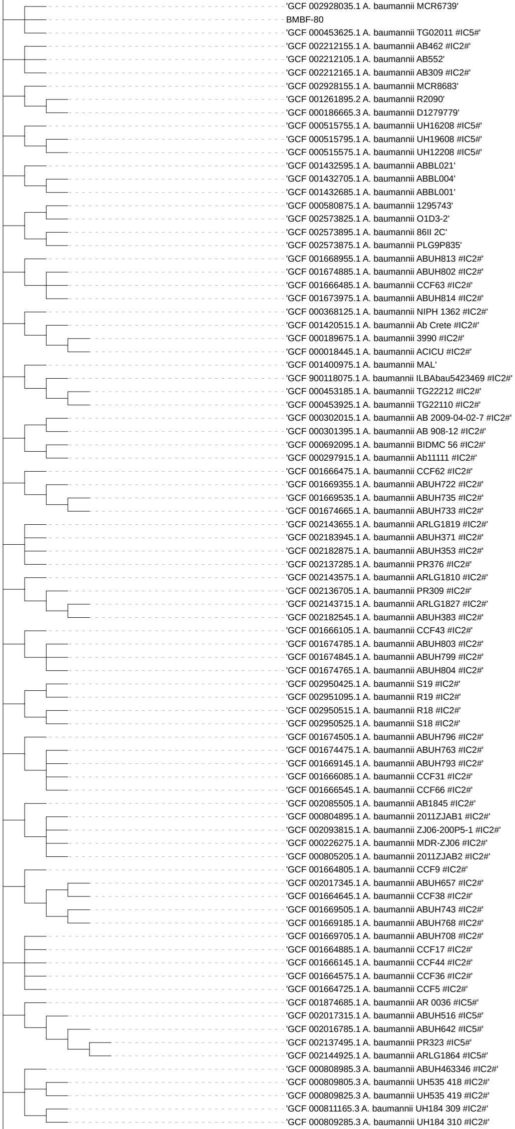

'GCF 002928035.1 A. baumannii MCR6739'
BMBF-80
'GCF 000453625.1 A. baumannii TG02011 #IC5#'
'GCF 002212155.1 A. baumannii AB462 #IC2#'
'GCF 002212105.1 A. baumannii AB552'
'GCF 002212165.1 A. baumannii AB309 #IC2#'
'GCF 002928155.1 A. baumannii MCR8683'
'GCF 001261895.2 A. baumannii R2090'
'GCF 000186665.3 A. baumannii D1279779'
'GCF 000515755.1 A. baumannii UH16208 #IC5#'
'GCF 000515795.1 A. baumannii UH19608 #IC5#'
'GCF 000515575.1 A. baumannii UH12208 #IC5#'
'GCF 001432595.1 A. baumannii ABBL021'
'GCF 001432705.1 A. baumannii ABBL004'
'GCF 001432685.1 A. baumannii ABBL001'
'GCF 000580875.1 A. baumannii 1295743'
'GCF 002573825.1 A. baumannii O1D3-2'
'GCF 002573895.1 A. baumannii 86II 2C'
'GCF 002573875.1 A. baumannii PLG9P835'
'GCF 001668955.1 A. baumannii ABUH813 #IC2#'
'GCF 001674885.1 A. baumannii ABUH802 #IC2#'
'GCF 001666485.1 A. baumannii CCF63 #IC2#'
'GCF 001673975.1 A. baumannii ABUH814 #IC2#'
'GCF 000368125.1 A. baumannii NIPH 1362 #IC2#'
'GCF 001420515.1 A. baumannii Ab Crete #IC2#'
'GCF 000189675.1 A. baumannii 3990 #IC2#'
'GCF 000018445.1 A. baumannii ACICU #IC2#'
'GCF 001400975.1 A. baumannii MAL'
'GCF 900118075.1 A. baumannii ILBAbau5423469 #IC2#'
'GCF 000453185.1 A. baumannii TG22212 #IC2#'
'GCF 000453925.1 A. baumannii TG22110 #IC2#'
'GCF 000302015.1 A. baumannii AB 2009-04-02-7 #IC2#'
'GCF 000301395.1 A. baumannii AB 908-12 #IC2#'
'GCF 000692095.1 A. baumannii BIDMC 56 #IC2#'
'GCF 000297915.1 A. baumannii Ab11111 #IC2#'
'GCF 001666475.1 A. baumannii CCF62 #IC2#'
'GCF 001669355.1 A. baumannii ABUH722 #IC2#'
'GCF 001669535.1 A. baumannii ABUH735 #IC2#'
'GCF 001674665.1 A. baumannii ABUH733 #IC2#'
'GCF 002143655.1 A. baumannii ARLG1819 #IC2#'
'GCF 002183945.1 A. baumannii ABUH371 #IC2#'
'GCF 002182875.1 A. baumannii ABUH353 #IC2#'
'GCF 002137285.1 A. baumannii PR376 #IC2#'
'GCF 002143575.1 A. baumannii ARLG1810 #IC2#'
'GCF 002136705.1 A. baumannii PR309 #IC2#'
'GCF 002143715.1 A. baumannii ARLG1827 #IC2#'
'GCF 002182545.1 A. baumannii ABUH383 #IC2#'
'GCF 001666105.1 A. baumannii CCF43 #IC2#'
'GCF 001674785.1 A. baumannii ABUH803 #IC2#'
'GCF 001674845.1 A. baumannii ABUH799 #IC2#'
'GCF 001674765.1 A. baumannii ABUH804 #IC2#'
'GCF 002950425.1 A. baumannii S19 #IC2#'
'GCF 002951095.1 A. baumannii R19 #IC2#'
'GCF 002950515.1 A. baumannii R18 #IC2#'
'GCF 002950525.1 A. baumannii S18 #IC2#'
'GCF 001674505.1 A. baumannii ABUH796 #IC2#'
'GCF 001674475.1 A. baumannii ABUH763 #IC2#'
'GCF 001669145.1 A. baumannii ABUH793 #IC2#'
'GCF 001666085.1 A. baumannii CCF31 #IC2#'
'GCF 001666545.1 A. baumannii CCF66 #IC2#'
'GCF 002085505.1 A. baumannii AB1845 #IC2#'
'GCF 000804895.1 A. baumannii 2011ZJAB1 #IC2#'
'GCF 002093815.1 A. baumannii ZJ06-200P5-1 #IC2#'
'GCF 000226275.1 A. baumannii MDR-ZJ06 #IC2#'
'GCF 000805205.1 A. baumannii 2011ZJAB2 #IC2#'
'GCF 001664805.1 A. baumannii CCF9 #IC2#'
'GCF 002017345.1 A. baumannii ABUH657 #IC2#'
'GCF 001664645.1 A. baumannii CCF38 #IC2#'
'GCF 001669505.1 A. baumannii ABUH743 #IC2#'
'GCF 001669185.1 A. baumannii ABUH768 #IC2#'
'GCF 001669705.1 A. baumannii ABUH708 #IC2#'
'GCF 001664885.1 A. baumannii CCF17 #IC2#'
'GCF 001666145.1 A. baumannii CCF44 #IC2#'
'GCF 001664575.1 A. baumannii CCF36 #IC2#'
'GCF 001664725.1 A. baumannii CCF5 #IC2#'
'GCF 001874685.1 A. baumannii AR 0036 #IC5#'
'GCF 002017315.1 A. baumannii ABUH516 #IC5#'
'GCF 002016785.1 A. baumannii ABUH642 #IC5#'
'GCF 002137495.1 A. baumannii PR323 #IC5#'
'GCF 002144925.1 A. baumannii ARLG1864 #IC5#'
'GCF 000808985.3 A. baumannii ABUH463346 #IC2#'
'GCF 000809805.3 A. baumannii UH535 418 #IC2#'
'GCF 000809825.3 A. baumannii UH535 419 #IC2#'
'GCF 000811165.3 A. baumannii UH184 309 #IC2#'
'GCF 000809285.3 A. baumannii UH184 310 #IC2#'

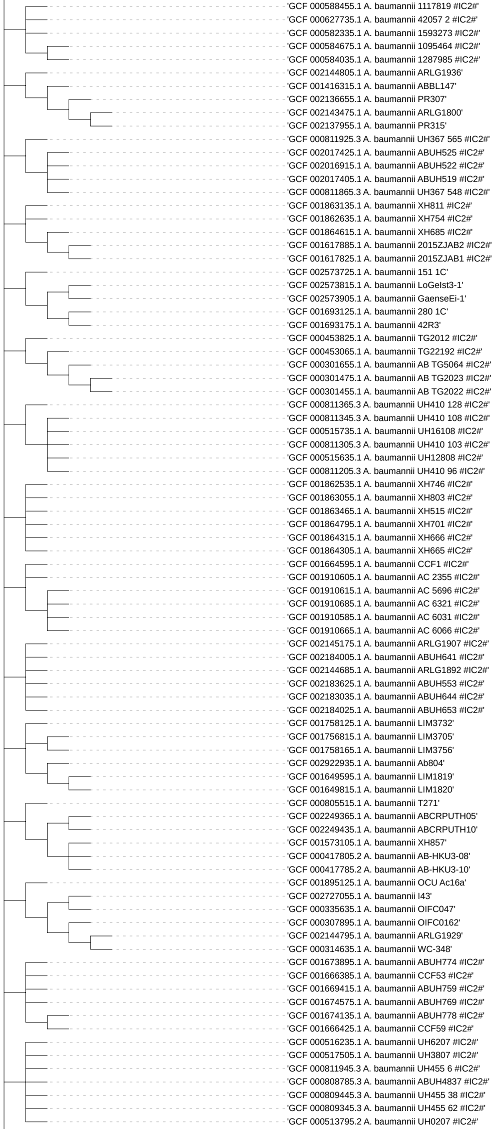

'GCF 000588455.1 A. baumannii 1117819 #IC2#'
'GCF 000627735.1 A. baumannii 42057 2 #IC2#'
'GCF 000582335.1 A. baumannii 1593273 #IC2#'
'GCF 000584675.1 A. baumannii 1095464 #IC2#'
'GCF 000584035.1 A. baumannii 1287985 #IC2#'
'GCF 002144805.1 A. baumannii ARLG1936'
'GCF 001416315.1 A. baumannii ABBL147'
'GCF 002136655.1 A. baumannii PR307'
'GCF 002143475.1 A. baumannii ARLG1800'
'GCF 002137955.1 A. baumannii PR315'
'GCF 000811925.3 A. baumannii UH367 565 #IC2#'
'GCF 002017425.1 A. baumannii ABUH525 #IC2#'
'GCF 002016915.1 A. baumannii ABUH522 #IC2#'
'GCF 002017405.1 A. baumannii ABUH519 #IC2#'
'GCF 000811865.3 A. baumannii UH367 548 #IC2#'
'GCF 001863135.1 A. baumannii XH811 #IC2#'
'GCF 001862635.1 A. baumannii XH754 #IC2#'
'GCF 001864615.1 A. baumannii XH685 #IC2#'
'GCF 001617885.1 A. baumannii 2015ZJAB2 #IC2#'
'GCF 001617825.1 A. baumannii 2015ZJAB1 #IC2#'
'GCF 002573725.1 A. baumannii 151 1C'
'GCF 002573815.1 A. baumannii LoGeIst3-1'
'GCF 002573905.1 A. baumannii GaenseEi-1'
'GCF 001693125.1 A. baumannii 280 1C'
'GCF 001693175.1 A. baumannii 42R3'
'GCF 000453825.1 A. baumannii TG2012 #IC2#'
'GCF 000453065.1 A. baumannii TG22192 #IC2#'
'GCF 000301655.1 A. baumannii AB TG5064 #IC2#'
'GCF 000301475.1 A. baumannii AB TG2023 #IC2#'
'GCF 000301455.1 A. baumannii AB TG2022 #IC2#'
'GCF 000811365.3 A. baumannii UH410 128 #IC2#'
'GCF 000811345.3 A. baumannii UH410 108 #IC2#'
'GCF 000515735.1 A. baumannii UH16108 #IC2#'
'GCF 000811305.3 A. baumannii UH410 103 #IC2#'
'GCF 000515635.1 A. baumannii UH12808 #IC2#'
'GCF 000811205.3 A. baumannii UH410 96 #IC2#'
'GCF 001862535.1 A. baumannii XH746 #IC2#'
'GCF 001863055.1 A. baumannii XH803 #IC2#'
'GCF 001863465.1 A. baumannii XH515 #IC2#'
'GCF 001864795.1 A. baumannii XH701 #IC2#'
'GCF 001864315.1 A. baumannii XH666 #IC2#'
'GCF 001864305.1 A. baumannii XH665 #IC2#'
'GCF 001664595.1 A. baumannii CCF1 #IC2#'
'GCF 001910605.1 A. baumannii AC 2355 #IC2#'
'GCF 001910615.1 A. baumannii AC 5696 #IC2#'
'GCF 001910685.1 A. baumannii AC 6321 #IC2#'
'GCF 001910585.1 A. baumannii AC 6031 #IC2#'
'GCF 001910665.1 A. baumannii AC 6066 #IC2#'
'GCF 002145175.1 A. baumannii ARLG1907 #IC2#'
'GCF 002184005.1 A. baumannii ABUH641 #IC2#'
'GCF 002144685.1 A. baumannii ARLG1892 #IC2#'
'GCF 002183625.1 A. baumannii ABUH553 #IC2#'
'GCF 002183035.1 A. baumannii ABUH644 #IC2#'
'GCF 002184025.1 A. baumannii ABUH653 #IC2#'
'GCF 001758125.1 A. baumannii LIM3732'
'GCF 001756815.1 A. baumannii LIM3705'
'GCF 001758165.1 A. baumannii LIM3756'
'GCF 002922935.1 A. baumannii Ab804'
'GCF 001649595.1 A. baumannii LIM1819'
'GCF 001649815.1 A. baumannii LIM1820'
'GCF 000805515.1 A. baumannii T271'
'GCF 002249365.1 A. baumannii ABCRPUTH05'
'GCF 002249435.1 A. baumannii ABCRPUTH10'
'GCF 001573105.1 A. baumannii XH857'
'GCF 000417805.2 A. baumannii AB-HKU3-08'
'GCF 000417785.2 A. baumannii AB-HKU3-10'
'GCF 001895125.1 A. baumannii OCU Ac16a'
'GCF 002727055.1 A. baumannii I43'
'GCF 000335635.1 A. baumannii OIFC047'
'GCF 000307895.1 A. baumannii OIFC0162'
'GCF 002144795.1 A. baumannii ARLG1929'
'GCF 000314635.1 A. baumannii WC-348'
'GCF 001673895.1 A. baumannii ABUH774 #IC2#'
'GCF 001666385.1 A. baumannii CCF53 #IC2#'
'GCF 001669415.1 A. baumannii ABUH759 #IC2#'
'GCF 001674575.1 A. baumannii ABUH769 #IC2#'
'GCF 001674135.1 A. baumannii ABUH778 #IC2#'
'GCF 001666425.1 A. baumannii CCF59 #IC2#'
'GCF 000516235.1 A. baumannii UH6207 #IC2#'
'GCF 000517505.1 A. baumannii UH3807 #IC2#'
'GCF 000811945.3 A. baumannii UH455 6 #IC2#'
'GCF 000808785.3 A. baumannii ABUH4837 #IC2#'
'GCF 000809445.3 A. baumannii UH455 38 #IC2#'
'GCF 000809345.3 A. baumannii UH455 62 #IC2#'
'GCF 000513795.2 A. baumannii UH0207 #IC2#'

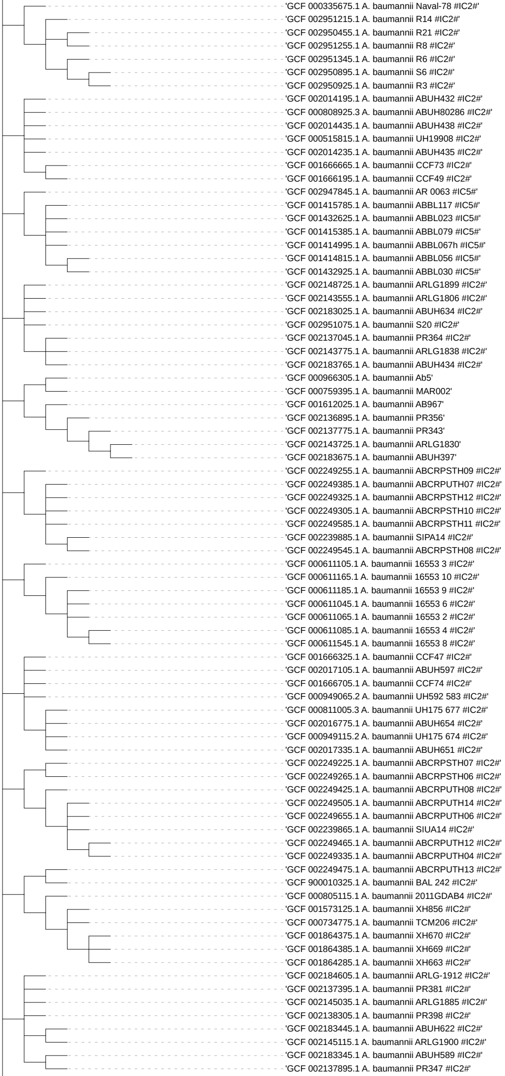
'GCF 000335675.1 A. baumannii Naval-78 #IC2#'
'GCF 002951215.1 A. baumannii R14 #IC2#'
'GCF 002950455.1 A. baumannii R21 #IC2#'
'GCF 002951255.1 A. baumannii R8 #IC2#'
'GCF 002951345.1 A. baumannii R6 #IC2#'
'GCF 002950895.1 A. baumannii S6 #IC2#'
'GCF 002950925.1 A. baumannii R3 #IC2#'
'GCF 002014195.1 A. baumannii ABUH432 #IC2#'
'GCF 000808925.3 A. baumannii ABUH80286 #IC2#'
'GCF 002014435.1 A. baumannii ABUH438 #IC2#'
'GCF 000515815.1 A. baumannii UH19908 #IC2#'
'GCF 002014235.1 A. baumannii ABUH435 #IC2#'
'GCF 001666665.1 A. baumannii CCF73 #IC2#'
'GCF 001666195.1 A. baumannii CCF49 #IC2#'
'GCF 002947845.1 A. baumannii AR 0063 #IC5#'
'GCF 001415785.1 A. baumannii ABBL117 #IC5#'
'GCF 001432625.1 A. baumannii ABBL023 #IC5#'
'GCF 001415385.1 A. baumannii ABBL079 #IC5#'
'GCF 001414995.1 A. baumannii ABBL067h #IC5#'
'GCF 001414815.1 A. baumannii ABBL056 #IC5#'
'GCF 001432925.1 A. baumannii ABBL030 #IC5#'
'GCF 002148725.1 A. baumannii ARLG1899 #IC2#'
'GCF 002143555.1 A. baumannii ARLG1806 #IC2#'
'GCF 002183025.1 A. baumannii ABUH634 #IC2#'
'GCF 002951075.1 A. baumannii S20 #IC2#'
'GCF 002137045.1 A. baumannii PR364 #IC2#'
'GCF 002143775.1 A. baumannii ARLG1838 #IC2#'
'GCF 002183765.1 A. baumannii ABUH434 #IC2#'
'GCF 000966305.1 A. baumannii Ab5'
'GCF 000759395.1 A. baumannii MAR002'
'GCF 001612025.1 A. baumannii AB967'
'GCF 002136895.1 A. baumannii PR356'
'GCF 002137775.1 A. baumannii PR343'
'GCF 002143725.1 A. baumannii ARLG1830'
'GCF 002183675.1 A. baumannii ABUH397'
'GCF 002249255.1 A. baumannii ABCRPSTH09 #IC2#'
'GCF 002249385.1 A. baumannii ABCRPUTH07 #IC2#'
'GCF 002249325.1 A. baumannii ABCRPSTH12 #IC2#'
'GCF 002249305.1 A. baumannii ABCRPSTH10 #IC2#'
'GCF 002249585.1 A. baumannii ABCRPSTH11 #IC2#'
'GCF 002239885.1 A. baumannii SIPA14 #IC2#'
'GCF 002249545.1 A. baumannii ABCRPSTH08 #IC2#'
'GCF 000611105.1 A. baumannii 16553 3 #IC2#'
'GCF 000611165.1 A. baumannii 16553 10 #IC2#'
'GCF 000611185.1 A. baumannii 16553 9 #IC2#'
'GCF 000611045.1 A. baumannii 16553 6 #IC2#'
'GCF 000611065.1 A. baumannii 16553 2 #IC2#'
'GCF 000611085.1 A. baumannii 16553 4 #IC2#'
'GCF 000611545.1 A. baumannii 16553 8 #IC2#'
'GCF 001666325.1 A. baumannii CCF47 #IC2#'
'GCF 002017105.1 A. baumannii ABUH597 #IC2#'
'GCF 001666705.1 A. baumannii CCF74 #IC2#'
'GCF 000949065.2 A. baumannii UH592 583 #IC2#'
'GCF 000811005.3 A. baumannii UH175 677 #IC2#'
'GCF 002016775.1 A. baumannii ABUH654 #IC2#'
'GCF 000949115.2 A. baumannii UH175 674 #IC2#'
'GCF 002017335.1 A. baumannii ABUH651 #IC2#'
'GCF 002249225.1 A. baumannii ABCRPSTH07 #IC2#'
'GCF 002249265.1 A. baumannii ABCRPSTH06 #IC2#'
'GCF 002249425.1 A. baumannii ABCRPUTH08 #IC2#'
'GCF 002249505.1 A. baumannii ABCRPUTH14 #IC2#'
'GCF 002249655.1 A. baumannii ABCRPUTH06 #IC2#'
'GCF 002239865.1 A. baumannii SIUA14 #IC2#'
'GCF 002249465.1 A. baumannii ABCRPUTH12 #IC2#'
'GCF 002249335.1 A. baumannii ABCRPUTH04 #IC2#'
'GCF 002249475.1 A. baumannii ABCRPUTH13 #IC2#'
'GCF 900010325.1 A. baumannii BAL 242 #IC2#'
'GCF 000805115.1 A. baumannii 2011GDAB4 #IC2#'
'GCF 001573125.1 A. baumannii XH856 #IC2#'
'GCF 000734775.1 A. baumannii TCM206 #IC2#'
'GCF 001864375.1 A. baumannii XH670 #IC2#'
'GCF 001864385.1 A. baumannii XH669 #IC2#'
'GCF 001864285.1 A. baumannii XH663 #IC2#'
'GCF 002184605.1 A. baumannii ARLG-1912 #IC2#'
'GCF 002137395.1 A. baumannii PR381 #IC2#'
'GCF 002145035.1 A. baumannii ARLG1885 #IC2#'
'GCF 002138305.1 A. baumannii PR398 #IC2#'
'GCF 002183445.1 A. baumannii ABUH622 #IC2#'
'GCF 002145115.1 A. baumannii ARLG1900 #IC2#'
'GCF 002183345.1 A. baumannii ABUH589 #IC2#'
'GCF 002137895.1 A. baumannii PR347 #IC2#'

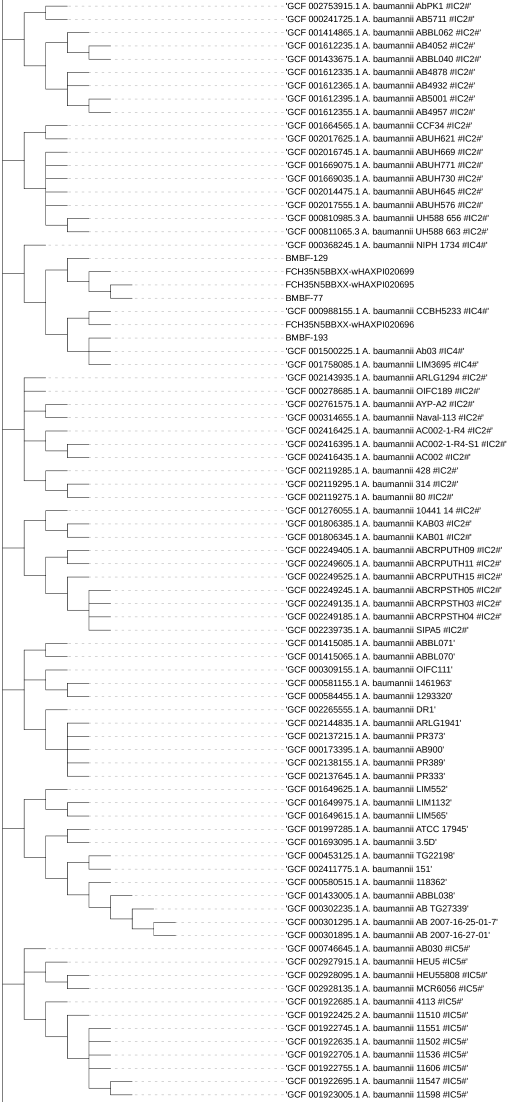
'GCF 002753915.1 A. baumannii AbPK1 #IC2#'
'GCF 000241725.1 A. baumannii AB5711 #IC2#'
'GCF 001414865.1 A. baumannii ABBL062 #IC2#'
'GCF 001612235.1 A. baumannii AB4052 #IC2#'
'GCF 001433675.1 A. baumannii ABBL040 #IC2#'
'GCF 001612335.1 A. baumannii AB4878 #IC2#'
'GCF 001612365.1 A. baumannii AB4932 #IC2#'
'GCF 001612395.1 A. baumannii AB5001 #IC2#'
'GCF 001612355.1 A. baumannii AB4957 #IC2#'
'GCF 001664565.1 A. baumannii CCF34 #IC2#'
'GCF 002017625.1 A. baumannii ABUH621 #IC2#'
'GCF 002016745.1 A. baumannii ABUH669 #IC2#'
'GCF 001669075.1 A. baumannii ABUH771 #IC2#'
'GCF 001669035.1 A. baumannii ABUH730 #IC2#'
'GCF 002014475.1 A. baumannii ABUH645 #IC2#'
'GCF 002017555.1 A. baumannii ABUH576 #IC2#'
'GCF 000810985.3 A. baumannii UH588 656 #IC2#'
'GCF 000811065.3 A. baumannii UH588 663 #IC2#'
'GCF 000368245.1 A. baumannii NIPH 1734 #IC4#'
BMBF-129
FCH35N5BBXX-wHAXPI020699
FCH35N5BBXX-wHAXPI020695
BMBF-77
'GCF 000988155.1 A. baumannii CCBH5233 #IC4#'
FCH35N5BBXX-wHAXPI020696
BMBF-193
'GCF 001500225.1 A. baumannii Ab03 #IC4#'
'GCF 001758085.1 A. baumannii LIM3695 #IC4#'
'GCF 002143935.1 A. baumannii ARLG1294 #IC2#'
'GCF 000278685.1 A. baumannii OIFC189 #IC2#'
'GCF 002761575.1 A. baumannii AYP-A2 #IC2#'
'GCF 000314655.1 A. baumannii Naval-113 #IC2#'
'GCF 002416425.1 A. baumannii AC002-1-R4 #IC2#'
'GCF 002416395.1 A. baumannii AC002-1-R4-S1 #IC2#'
'GCF 002416435.1 A. baumannii AC002 #IC2#'
'GCF 002119285.1 A. baumannii 428 #IC2#'
'GCF 002119295.1 A. baumannii 314 #IC2#'
'GCF 002119275.1 A. baumannii 80 #IC2#'
'GCF 001276055.1 A. baumannii 10441 14 #IC2#'
'GCF 001806385.1 A. baumannii KAB03 #IC2#'
'GCF 001806345.1 A. baumannii KAB01 #IC2#'
'GCF 002249405.1 A. baumannii ABCRPUTH09 #IC2#'
'GCF 002249605.1 A. baumannii ABCRPUTH11 #IC2#'
'GCF 002249525.1 A. baumannii ABCRPUTH15 #IC2#'
'GCF 002249245.1 A. baumannii ABCRPSTH05 #IC2#'
'GCF 002249135.1 A. baumannii ABCRPSTH03 #IC2#'
'GCF 002249185.1 A. baumannii ABCRPSTH04 #IC2#'
'GCF 002239735.1 A. baumannii SIPA5 #IC2#'
'GCF 001415085.1 A. baumannii ABBL071'
'GCF 001415065.1 A. baumannii ABBL070'
'GCF 000309155.1 A. baumannii OIFC111'
'GCF 000581155.1 A. baumannii 1461963'
'GCF 000584455.1 A. baumannii 1293320'
'GCF 002265555.1 A. baumannii DR1'
'GCF 002144835.1 A. baumannii ARLG1941'
'GCF 002137215.1 A. baumannii PR373'
'GCF 000173395.1 A. baumannii AB900'
'GCF 002138155.1 A. baumannii PR389'
'GCF 002137645.1 A. baumannii PR333'
'GCF 001649625.1 A. baumannii LIM552'
'GCF 001649975.1 A. baumannii LIM1132'
'GCF 001649615.1 A. baumannii LIM565'
'GCF 001997285.1 A. baumannii ATCC 17945'
'GCF 001693095.1 A. baumannii 3.5D'
'GCF 000453125.1 A. baumannii TG22198'
'GCF 002411775.1 A. baumannii 151'
'GCF 000580515.1 A. baumannii 118362'
'GCF 001433005.1 A. baumannii ABBL038'
'GCF 000302235.1 A. baumannii AB TG27339'
'GCF 000301295.1 A. baumannii AB 2007-16-25-01-7'
'GCF 000301895.1 A. baumannii AB 2007-16-27-01'
'GCF 000746645.1 A. baumannii AB030 #IC5#'
'GCF 002927915.1 A. baumannii HEU5 #IC5#'
'GCF 002928095.1 A. baumannii HEU55808 #IC5#'
'GCF 002928135.1 A. baumannii MCR6056 #IC5#'
'GCF 001922685.1 A. baumannii 4113 #IC5#'
'GCF 001922425.2 A. baumannii 11510 #IC5#'
'GCF 001922745.1 A. baumannii 11551 #IC5#'
'GCF 001922635.1 A. baumannii 11502 #IC5#'
'GCF 001922705.1 A. baumannii 11536 #IC5#'
'GCF 001922755.1 A. baumannii 11606 #IC5#'
'GCF 001922695.1 A. baumannii 11547 #IC5#'
'GCF 001923005.1 A. baumannii 11598 #IC5#'

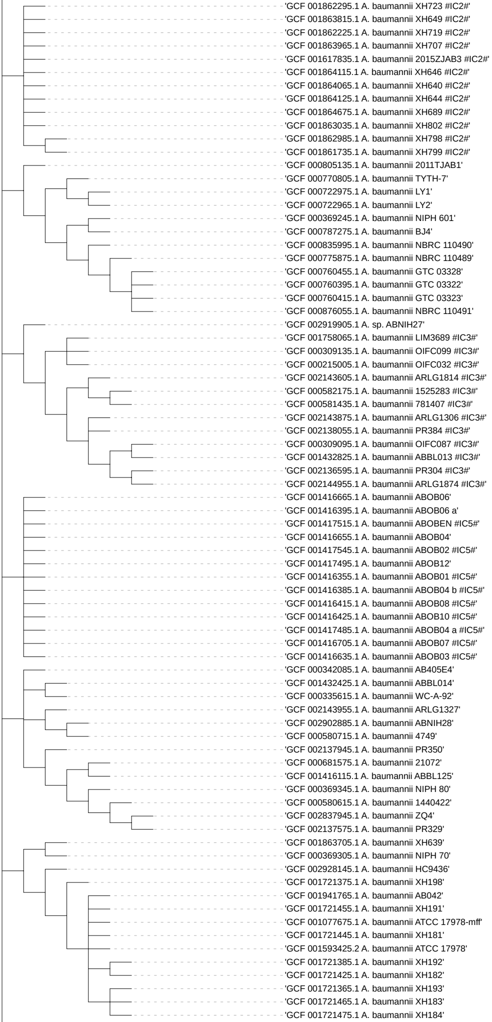
'GCF 001862295.1 A. baumannii XH723 #IC2#'
'GCF 001863815.1 A. baumannii XH649 #IC2#'
'GCF 001862225.1 A. baumannii XH719 #IC2#'
'GCF 001863965.1 A. baumannii XH707 #IC2#'
'GCF 001617835.1 A. baumannii 2015ZJAB3 #IC2#'
'GCF 001864115.1 A. baumannii XH646 #IC2#'
'GCF 001864065.1 A. baumannii XH640 #IC2#'
'GCF 001864125.1 A. baumannii XH644 #IC2#'
'GCF 001864675.1 A. baumannii XH689 #IC2#'
'GCF 001863035.1 A. baumannii XH802 #IC2#'
'GCF 001862985.1 A. baumannii XH798 #IC2#'
'GCF 001861735.1 A. baumannii XH799 #IC2#'
'GCF 000805135.1 A. baumannii 2011TJAB1'
'GCF 000770805.1 A. baumannii TYTH-7'
'GCF 000722975.1 A. baumannii LY1'
'GCF 000722965.1 A. baumannii LY2'
'GCF 000369245.1 A. baumannii NIPH 601'
'GCF 000787275.1 A. baumannii BJ4'
'GCF 000835995.1 A. baumannii NBRC 110490'
'GCF 000775875.1 A. baumannii NBRC 110489'
'GCF 000760455.1 A. baumannii GTC 03328'
'GCF 000760395.1 A. baumannii GTC 03322'
'GCF 000760415.1 A. baumannii GTC 03323'
'GCF 000876055.1 A. baumannii NBRC 110491'
'GCF 002919905.1 A. sp. ABNIH27'
'GCF 001758065.1 A. baumannii LIM3689 #IC3#'
'GCF 000309135.1 A. baumannii OIFC099 #IC3#'
'GCF 000215005.1 A. baumannii OIFC032 #IC3#'
'GCF 002143605.1 A. baumannii ARLG1814 #IC3#'
'GCF 000582175.1 A. baumannii 1525283 #IC3#'
'GCF 000581435.1 A. baumannii 781407 #IC3#'
'GCF 002143875.1 A. baumannii ARLG1306 #IC3#'
'GCF 002138055.1 A. baumannii PR384 #IC3#'
'GCF 000309095.1 A. baumannii OIFC087 #IC3#'
'GCF 001432825.1 A. baumannii ABBL013 #IC3#'
'GCF 002136595.1 A. baumannii PR304 #IC3#'
'GCF 002144955.1 A. baumannii ARLG1874 #IC3#'
'GCF 001416665.1 A. baumannii ABOB06'
'GCF 001416395.1 A. baumannii ABOB06 a'
'GCF 001417515.1 A. baumannii ABOBEN #IC5#'
'GCF 001416655.1 A. baumannii ABOB04'
'GCF 001417545.1 A. baumannii ABOB02 #IC5#'
'GCF 001417495.1 A. baumannii ABOB12'
'GCF 001416355.1 A. baumannii ABOB01 #IC5#'
'GCF 001416385.1 A. baumannii ABOB04 b #IC5#'
'GCF 001416415.1 A. baumannii ABOB08 #IC5#'
'GCF 001416425.1 A. baumannii ABOB10 #IC5#'
'GCF 001417485.1 A. baumannii ABOB04 a #IC5#'
'GCF 001416705.1 A. baumannii ABOB07 #IC5#'
'GCF 001416635.1 A. baumannii ABOB03 #IC5#'
'GCF 000342085.1 A. baumannii AB405E4'
'GCF 001432425.1 A. baumannii ABBL014'
'GCF 000335615.1 A. baumannii WC-A-92'
'GCF 002143955.1 A. baumannii ARLG1327'
'GCF 002902885.1 A. baumannii ABNIH28'
'GCF 000580715.1 A. baumannii 4749'
'GCF 002137945.1 A. baumannii PR350'
'GCF 000681575.1 A. baumannii 21072'
'GCF 001416115.1 A. baumannii ABBL125'
'GCF 000369345.1 A. baumannii NIPH 80'
'GCF 000580615.1 A. baumannii 1440422'
'GCF 002837945.1 A. baumannii ZQ4'
'GCF 002137575.1 A. baumannii PR329'
'GCF 001863705.1 A. baumannii XH639'
'GCF 000369305.1 A. baumannii NIPH 70'
'GCF 002928145.1 A. baumannii HC9436'
'GCF 001721375.1 A. baumannii XH198'
'GCF 001941765.1 A. baumannii AB042'
'GCF 001721455.1 A. baumannii XH191'
'GCF 001077675.1 A. baumannii ATCC 17978-mff'
'GCF 001721445.1 A. baumannii XH181'
'GCF 001593425.2 A. baumannii ATCC 17978'
'GCF 001721385.1 A. baumannii XH192'
'GCF 001721425.1 A. baumannii XH182'
'GCF 001721365.1 A. baumannii XH193'
'GCF 001721465.1 A. baumannii XH183'
'GCF 001721475.1 A. baumannii XH184'

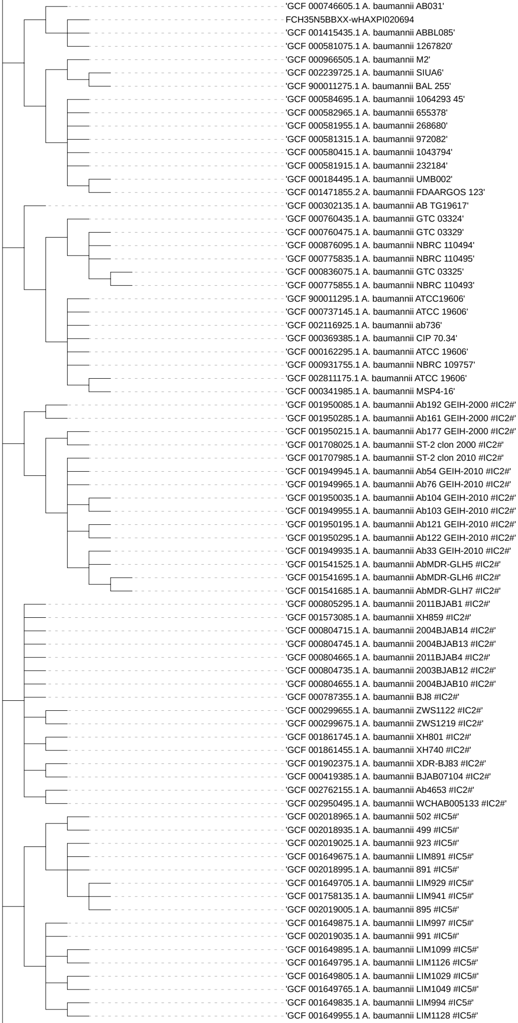
'GCF 000746605.1 A. baumannii AB031'
FCH35N5BBXX-wHAXPI020694
'GCF 001415435.1 A. baumannii ABBL085'
'GCF 000581075.1 A. baumannii 1267820'
'GCF 000966505.1 A. baumannii M2'
'GCF 002239725.1 A. baumannii SIUA6'
'GCF 900011275.1 A. baumannii BAL 255'
'GCF 000584695.1 A. baumannii 1064293 45'
'GCF 000582965.1 A. baumannii 655378'
'GCF 000581955.1 A. baumannii 268680'
'GCF 000581315.1 A. baumannii 972082'
'GCF 000580415.1 A. baumannii 1043794'
'GCF 000581915.1 A. baumannii 232184'
'GCF 000184495.1 A. baumannii UMB002'
'GCF 001471855.2 A. baumannii FDAARGOS 123'
'GCF 000302135.1 A. baumannii AB TG19617'
'GCF 000760435.1 A. baumannii GTC 03324'
'GCF 000760475.1 A. baumannii GTC 03329'
'GCF 000876095.1 A. baumannii NBRC 110494'
'GCF 000775835.1 A. baumannii NBRC 110495'
'GCF 000836075.1 A. baumannii GTC 03325'
'GCF 000775855.1 A. baumannii NBRC 110493'
'GCF 900011295.1 A. baumannii ATCC19606'
'GCF 000737145.1 A. baumannii ATCC 19606'
'GCF 002116925.1 A. baumannii ab736'
'GCF 000369385.1 A. baumannii CIP 70.34'
'GCF 000162295.1 A. baumannii ATCC 19606'
'GCF 000931755.1 A. baumannii NBRC 109757'
'GCF 002811175.1 A. baumannii ATCC 19606'
'GCF 000341985.1 A. baumannii MSP4-16'
'GCF 001950085.1 A. baumannii Ab192 GEIH-2000 #IC2#'
'GCF 001950285.1 A. baumannii Ab161 GEIH-2000 #IC2#'
'GCF 001950215.1 A. baumannii Ab177 GEIH-2000 #IC2#'
'GCF 001708025.1 A. baumannii ST-2 clon 2000 #IC2#'
'GCF 001707985.1 A. baumannii ST-2 clon 2010 #IC2#'
'GCF 001949945.1 A. baumannii Ab54 GEIH-2010 #IC2#'
'GCF 001949965.1 A. baumannii Ab76 GEIH-2010 #IC2#'
'GCF 001950035.1 A. baumannii Ab104 GEIH-2010 #IC2#'
'GCF 001949955.1 A. baumannii Ab103 GEIH-2010 #IC2#'
'GCF 001950195.1 A. baumannii Ab121 GEIH-2010 #IC2#'
'GCF 001950295.1 A. baumannii Ab122 GEIH-2010 #IC2#'
'GCF 001949935.1 A. baumannii Ab33 GEIH-2010 #IC2#'
'GCF 001541525.1 A. baumannii AbMDR-GLH5 #IC2#'
'GCF 001541695.1 A. baumannii AbMDR-GLH6 #IC2#'
'GCF 001541685.1 A. baumannii AbMDR-GLH7 #IC2#'
'GCF 000805295.1 A. baumannii 2011BJAB1 #IC2#'
'GCF 001573085.1 A. baumannii XH859 #IC2#'
'GCF 000804715.1 A. baumannii 2004BJAB14 #IC2#'
'GCF 000804745.1 A. baumannii 2004BJAB13 #IC2#'
'GCF 000804665.1 A. baumannii 2011BJAB4 #IC2#'
'GCF 000804735.1 A. baumannii 2003BJAB12 #IC2#'
'GCF 000804655.1 A. baumannii 2004BJAB10 #IC2#'
'GCF 000787355.1 A. baumannii BJ8 #IC2#'
'GCF 000299655.1 A. baumannii ZWS1122 #IC2#'
'GCF 000299675.1 A. baumannii ZWS1219 #IC2#'
'GCF 001861745.1 A. baumannii XH801 #IC2#'
'GCF 001861455.1 A. baumannii XH740 #IC2#'
'GCF 001902375.1 A. baumannii XDR-BJ83 #IC2#'
'GCF 000419385.1 A. baumannii BJAB07104 #IC2#'
'GCF 002762155.1 A. baumannii Ab4653 #IC2#'
'GCF 002950495.1 A. baumannii WCHAB005133 #IC2#'
'GCF 002018965.1 A. baumannii 502 #IC5#'
'GCF 002018935.1 A. baumannii 499 #IC5#'
'GCF 002019025.1 A. baumannii 923 #IC5#'
'GCF 001649675.1 A. baumannii LIM891 #IC5#'
'GCF 002018995.1 A. baumannii 891 #IC5#'
'GCF 001649705.1 A. baumannii LIM929 #IC5#'
'GCF 001758135.1 A. baumannii LIM941 #IC5#'
'GCF 002019005.1 A. baumannii 895 #IC5#'
'GCF 001649875.1 A. baumannii LIM997 #IC5#'
'GCF 002019035.1 A. baumannii 991 #IC5#'
'GCF 001649895.1 A. baumannii LIM1099 #IC5#'
'GCF 001649795.1 A. baumannii LIM1126 #IC5#'
'GCF 001649805.1 A. baumannii LIM1029 #IC5#'
'GCF 001649765.1 A. baumannii LIM1049 #IC5#'
'GCF 001649835.1 A. baumannii LIM994 #IC5#'
'GCF 001649955.1 A. baumannii LIM1128 #IC5#'

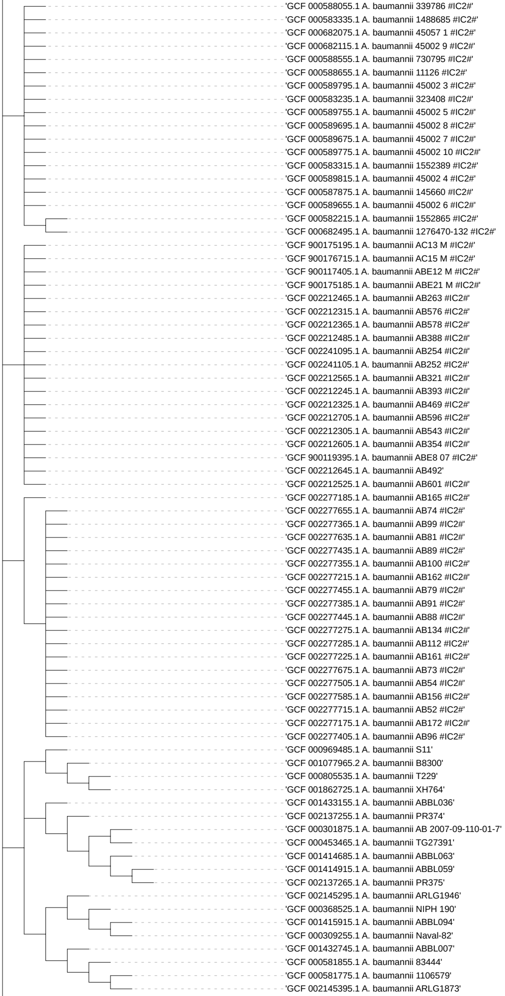
'GCF 000588055.1 A. baumannii 339786 #IC2#'
'GCF 000583335.1 A. baumannii 1488685 #IC2#'
'GCF 000682075.1 A. baumannii 45057 1 #IC2#'
'GCF 000682115.1 A. baumannii 45002 9 #IC2#'
'GCF 000588555.1 A. baumannii 730795 #IC2#'
'GCF 000588655.1 A. baumannii 11126 #IC2#'
'GCF 000589795.1 A. baumannii 45002 3 #IC2#'
'GCF 000583235.1 A. baumannii 323408 #IC2#'
'GCF 000589755.1 A. baumannii 45002 5 #IC2#'
'GCF 000589695.1 A. baumannii 45002 8 #IC2#'
'GCF 000589675.1 A. baumannii 45002 7 #IC2#'
'GCF 000589775.1 A. baumannii 45002 10 #IC2#'
'GCF 000583315.1 A. baumannii 1552389 #IC2#'
'GCF 000589815.1 A. baumannii 45002 4 #IC2#'
'GCF 000587875.1 A. baumannii 145660 #IC2#'
'GCF 000589655.1 A. baumannii 45002 6 #IC2#'
'GCF 000582215.1 A. baumannii 1552865 #IC2#'
'GCF 000682495.1 A. baumannii 1276470-132 #IC2#'
'GCF 900175195.1 A. baumannii AC13 M #IC2#'
'GCF 900176715.1 A. baumannii AC15 M #IC2#'
'GCF 900117405.1 A. baumannii ABE12 M #IC2#'
'GCF 900175185.1 A. baumannii ABE21 M #IC2#'
'GCF 002212465.1 A. baumannii AB263 #IC2#'
'GCF 002212315.1 A. baumannii AB576 #IC2#'
'GCF 002212365.1 A. baumannii AB578 #IC2#'
'GCF 002212485.1 A. baumannii AB388 #IC2#'
'GCF 002241095.1 A. baumannii AB254 #IC2#'
'GCF 002241105.1 A. baumannii AB252 #IC2#'
'GCF 002212565.1 A. baumannii AB321 #IC2#'
'GCF 002212245.1 A. baumannii AB393 #IC2#'
'GCF 002212325.1 A. baumannii AB469 #IC2#'
'GCF 002212705.1 A. baumannii AB596 #IC2#'
'GCF 002212305.1 A. baumannii AB543 #IC2#'
'GCF 002212605.1 A. baumannii AB354 #IC2#'
'GCF 900119395.1 A. baumannii ABE8 07 #IC2#'
'GCF 002212645.1 A. baumannii AB492'
'GCF 002212525.1 A. baumannii AB601 #IC2#'
'GCF 002277185.1 A. baumannii AB165 #IC2#'
'GCF 002277655.1 A. baumannii AB74 #IC2#'
'GCF 002277365.1 A. baumannii AB99 #IC2#'
'GCF 002277635.1 A. baumannii AB81 #IC2#'
'GCF 002277435.1 A. baumannii AB89 #IC2#'
'GCF 002277355.1 A. baumannii AB100 #IC2#'
'GCF 002277215.1 A. baumannii AB162 #IC2#'
'GCF 002277455.1 A. baumannii AB79 #IC2#'
'GCF 002277385.1 A. baumannii AB91 #IC2#'
'GCF 002277445.1 A. baumannii AB88 #IC2#'
'GCF 002277275.1 A. baumannii AB134 #IC2#'
'GCF 002277285.1 A. baumannii AB112 #IC2#'
'GCF 002277225.1 A. baumannii AB161 #IC2#'
'GCF 002277675.1 A. baumannii AB73 #IC2#'
'GCF 002277505.1 A. baumannii AB54 #IC2#'
'GCF 002277585.1 A. baumannii AB156 #IC2#'
'GCF 002277715.1 A. baumannii AB52 #IC2#'
'GCF 002277175.1 A. baumannii AB172 #IC2#'
'GCF 002277405.1 A. baumannii AB96 #IC2#'
'GCF 000969485.1 A. baumannii S11'
'GCF 001077965.2 A. baumannii B8300'
'GCF 000805535.1 A. baumannii T229'
'GCF 001862725.1 A. baumannii XH764'
'GCF 001433155.1 A. baumannii ABBL036'
'GCF 002137255.1 A. baumannii PR374'
'GCF 000301875.1 A. baumannii AB 2007-09-110-01-7'
'GCF 000453465.1 A. baumannii TG27391'
'GCF 001414685.1 A. baumannii ABBL063'
'GCF 001414915.1 A. baumannii ABBL059'
'GCF 002137265.1 A. baumannii PR375'
'GCF 002145295.1 A. baumannii ARLG1946'
'GCF 000368525.1 A. baumannii NIPH 190'
'GCF 001415915.1 A. baumannii ABBL094'
'GCF 000309255.1 A. baumannii Naval-82'
'GCF 001432745.1 A. baumannii ABBL007'
'GCF 000581855.1 A. baumannii 83444'
'GCF 000581775.1 A. baumannii 1106579'
'GCF 002145395.1 A. baumannii ARLG1873'

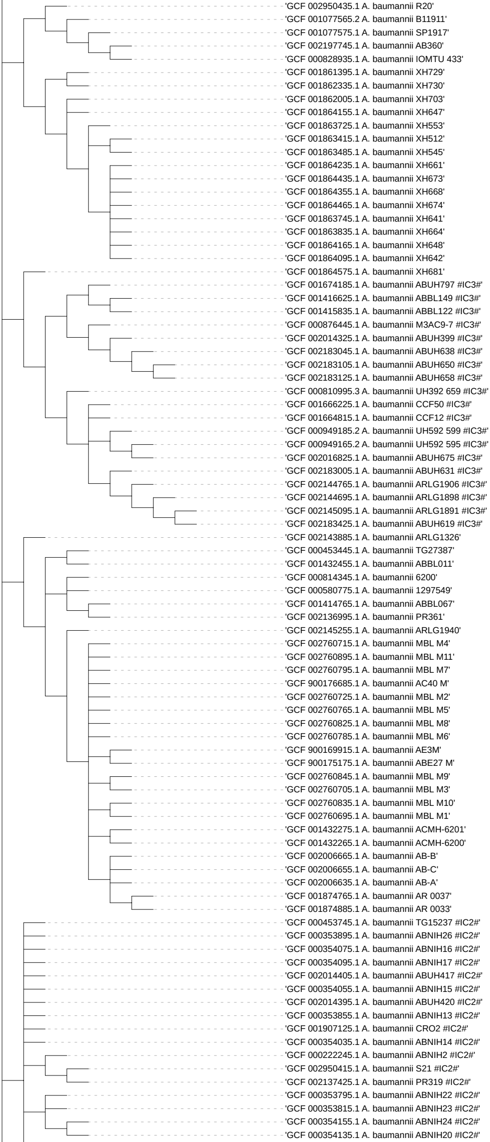
'GCF 002950435.1 A. baumannii R20'
'GCF 001077565.2 A. baumannii B11911'
'GCF 001077575.1 A. baumannii SP1917'
'GCF 002197745.1 A. baumannii AB360'
'GCF 000828935.1 A. baumannii IOMTU 433'
'GCF 001861395.1 A. baumannii XH729'
'GCF 001862335.1 A. baumannii XH730'
'GCF 001862005.1 A. baumannii XH703'
'GCF 001864155.1 A. baumannii XH647'
'GCF 001863725.1 A. baumannii XH553'
'GCF 001863415.1 A. baumannii XH512'
'GCF 001863485.1 A. baumannii XH545'
'GCF 001864235.1 A. baumannii XH661'
'GCF 001864435.1 A. baumannii XH673'
'GCF 001864355.1 A. baumannii XH668'
'GCF 001864465.1 A. baumannii XH674'
'GCF 001863745.1 A. baumannii XH641'
'GCF 001863835.1 A. baumannii XH664'
'GCF 001864165.1 A. baumannii XH648'
'GCF 001864095.1 A. baumannii XH642'
'GCF 001864575.1 A. baumannii XH681'
'GCF 001674185.1 A. baumannii ABUH797 #IC3#'
'GCF 001416625.1 A. baumannii ABBL149 #IC3#'
'GCF 001415835.1 A. baumannii ABBL122 #IC3#'
'GCF 000876445.1 A. baumannii M3AC9-7 #IC3#'
'GCF 002014325.1 A. baumannii ABUH399 #IC3#'
'GCF 002183045.1 A. baumannii ABUH638 #IC3#'
'GCF 002183105.1 A. baumannii ABUH650 #IC3#'
'GCF 002183125.1 A. baumannii ABUH658 #IC3#'
'GCF 000810995.3 A. baumannii UH392 659 #IC3#'
'GCF 001666225.1 A. baumannii CCF50 #IC3#'
'GCF 001664815.1 A. baumannii CCF12 #IC3#'
'GCF 000949185.2 A. baumannii UH592 599 #IC3#'
'GCF 000949165.2 A. baumannii UH592 595 #IC3#'
'GCF 002016825.1 A. baumannii ABUH675 #IC3#'
'GCF 002183005.1 A. baumannii ABUH631 #IC3#'
'GCF 002144765.1 A. baumannii ARLG1906 #IC3#'
'GCF 002144695.1 A. baumannii ARLG1898 #IC3#'
'GCF 002145095.1 A. baumannii ARLG1891 #IC3#'
'GCF 002183425.1 A. baumannii ABUH619 #IC3#'
'GCF 002143885.1 A. baumannii ARLG1326'
'GCF 000453445.1 A. baumannii TG27387'
'GCF 001432455.1 A. baumannii ABBL011'
'GCF 000814345.1 A. baumannii 6200'
'GCF 000580775.1 A. baumannii 1297549'
'GCF 001414765.1 A. baumannii ABBL067'
'GCF 002136995.1 A. baumannii PR361'
'GCF 002145255.1 A. baumannii ARLG1940'
'GCF 002760715.1 A. baumannii MBL M4'
'GCF 002760895.1 A. baumannii MBL M11'
'GCF 002760795.1 A. baumannii MBL M7'
'GCF 900176685.1 A. baumannii AC40 M'
'GCF 002760725.1 A. baumannii MBL M2'
'GCF 002760765.1 A. baumannii MBL M5'
'GCF 002760825.1 A. baumannii MBL M8'
'GCF 002760785.1 A. baumannii MBL M6'
'GCF 900169915.1 A. baumannii AE3M'
'GCF 900175175.1 A. baumannii ABE27 M'
'GCF 002760845.1 A. baumannii MBL M9'
'GCF 002760705.1 A. baumannii MBL M3'
'GCF 002760835.1 A. baumannii MBL M10'
'GCF 002760695.1 A. baumannii MBL M1'
'GCF 001432275.1 A. baumannii ACMH-6201'
'GCF 001432265.1 A. baumannii ACMH-6200'
'GCF 002006665.1 A. baumannii AB-B'
'GCF 002006655.1 A. baumannii AB-C'
'GCF 002006635.1 A. baumannii AB-A'
'GCF 001874765.1 A. baumannii AR 0037'
'GCF 001874885.1 A. baumannii AR 0033'
'GCF 000453745.1 A. baumannii TG15237 #IC2#'
'GCF 000353895.1 A. baumannii ABNIH26 #IC2#'
'GCF 000354075.1 A. baumannii ABNIH16 #IC2#'
'GCF 000354095.1 A. baumannii ABNIH17 #IC2#'
'GCF 002014405.1 A. baumannii ABUH417 #IC2#'
'GCF 000354055.1 A. baumannii ABNIH15 #IC2#'
'GCF 002014395.1 A. baumannii ABUH420 #IC2#'
'GCF 000353855.1 A. baumannii ABNIH13 #IC2#'
'GCF 001907125.1 A. baumannii CRO2 #IC2#'
'GCF 000354035.1 A. baumannii ABNIH14 #IC2#'
'GCF 000222245.1 A. baumannii ABNIH2 #IC2#'
'GCF 002950415.1 A. baumannii S21 #IC2#'
'GCF 002137425.1 A. baumannii PR319 #IC2#'
'GCF 000353795.1 A. baumannii ABNIH22 #IC2#'
'GCF 000353815.1 A. baumannii ABNIH23 #IC2#'
'GCF 000354155.1 A. baumannii ABNIH24 #IC2#'
'GCF 000354135.1 A. baumannii ABNIH20 #IC2#'

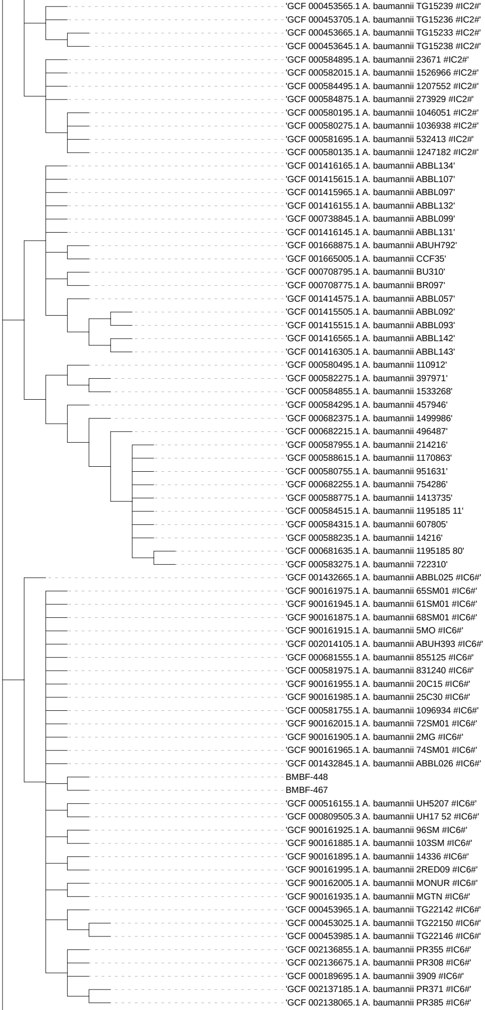
'GCF 000453565.1 A. baumannii TG15239 #IC2#'
'GCF 000453705.1 A. baumannii TG15236 #IC2#'
'GCF 000453665.1 A. baumannii TG15233 #IC2#'
'GCF 000453645.1 A. baumannii TG15238 #IC2#'
'GCF 000584895.1 A. baumannii 23671 #IC2#'
'GCF 000582015.1 A. baumannii 1526966 #IC2#'
'GCF 000584495.1 A. baumannii 1207552 #IC2#'
'GCF 000584875.1 A. baumannii 273929 #IC2#'
'GCF 000580195.1 A. baumannii 1046051 #IC2#'
'GCF 000580275.1 A. baumannii 1036938 #IC2#'
'GCF 000581695.1 A. baumannii 532413 #IC2#'
'GCF 000580135.1 A. baumannii 1247182 #IC2#'
'GCF 001416165.1 A. baumannii ABBL134'
'GCF 001415615.1 A. baumannii ABBL107'
'GCF 001415965.1 A. baumannii ABBL097'
'GCF 001416155.1 A. baumannii ABBL132'
'GCF 000738845.1 A. baumannii ABBL099'
'GCF 001416145.1 A. baumannii ABBL131'
'GCF 001668875.1 A. baumannii ABUH792'
'GCF 001665005.1 A. baumannii CCF35'
'GCF 000708795.1 A. baumannii BU310'
'GCF 000708775.1 A. baumannii BR097'
'GCF 001414575.1 A. baumannii ABBL057'
'GCF 001415505.1 A. baumannii ABBL092'
'GCF 001415515.1 A. baumannii ABBL093'
'GCF 001416565.1 A. baumannii ABBL142'
'GCF 001416305.1 A. baumannii ABBL143'
'GCF 000580495.1 A. baumannii 110912'
'GCF 000582275.1 A. baumannii 397971'
'GCF 000584855.1 A. baumannii 1533268'
'GCF 000584295.1 A. baumannii 457946'
'GCF 000682375.1 A. baumannii 1499986'
'GCF 000682215.1 A. baumannii 496487'
'GCF 000587955.1 A. baumannii 214216'
'GCF 000588615.1 A. baumannii 1170863'
'GCF 000580755.1 A. baumannii 951631'
'GCF 000682255.1 A. baumannii 754286'
'GCF 000588775.1 A. baumannii 1413735'
'GCF 000584515.1 A. baumannii 1195185 11'
'GCF 000584315.1 A. baumannii 607805'
'GCF 000588235.1 A. baumannii 14216'
'GCF 000681635.1 A. baumannii 1195185 80'
'GCF 000583275.1 A. baumannii 722310'
'GCF 001432665.1 A. baumannii ABBL025 #IC6#'
'GCF 900161975.1 A. baumannii 65SM01 #IC6#'
'GCF 900161945.1 A. baumannii 61SM01 #IC6#'
'GCF 900161875.1 A. baumannii 68SM01 #IC6#'
'GCF 900161915.1 A. baumannii 5MO #IC6#'
'GCF 002014105.1 A. baumannii ABUH393 #IC6#'
'GCF 000681555.1 A. baumannii 855125 #IC6#'
'GCF 000581975.1 A. baumannii 831240 #IC6#'
'GCF 900161955.1 A. baumannii 20C15 #IC6#'
'GCF 900161985.1 A. baumannii 25C30 #IC6#'
'GCF 000581755.1 A. baumannii 1096934 #IC6#'
'GCF 900162015.1 A. baumannii 72SM01 #IC6#'
'GCF 900161905.1 A. baumannii 2MG #IC6#'
'GCF 900161965.1 A. baumannii 74SM01 #IC6#'
'GCF 001432845.1 A. baumannii ABBL026 #IC6#'
BMBF-448
BMBF-467
'GCF 000516155.1 A. baumannii UH5207 #IC6#'
'GCF 000809505.3 A. baumannii UH17 52 #IC6#'
'GCF 900161925.1 A. baumannii 96SM #IC6#'
'GCF 900161885.1 A. baumannii 103SM #IC6#'
'GCF 900161895.1 A. baumannii 14336 #IC6#'
'GCF 900161995.1 A. baumannii 2RED09 #IC6#'
'GCF 900162005.1 A. baumannii MONUR #IC6#'
'GCF 900161935.1 A. baumannii MGTN #IC6#'
'GCF 000453965.1 A. baumannii TG22142 #IC6#'
'GCF 000453025.1 A. baumannii TG22150 #IC6#'
'GCF 000453985.1 A. baumannii TG22146 #IC6#'
'GCF 002136855.1 A. baumannii PR355 #IC6#'
'GCF 002136675.1 A. baumannii PR308 #IC6#'
'GCF 000189695.1 A. baumannii 3909 #IC6#'
'GCF 002137185.1 A. baumannii PR371 #IC6#'
'GCF 002138065.1 A. baumannii PR385 #IC6#'

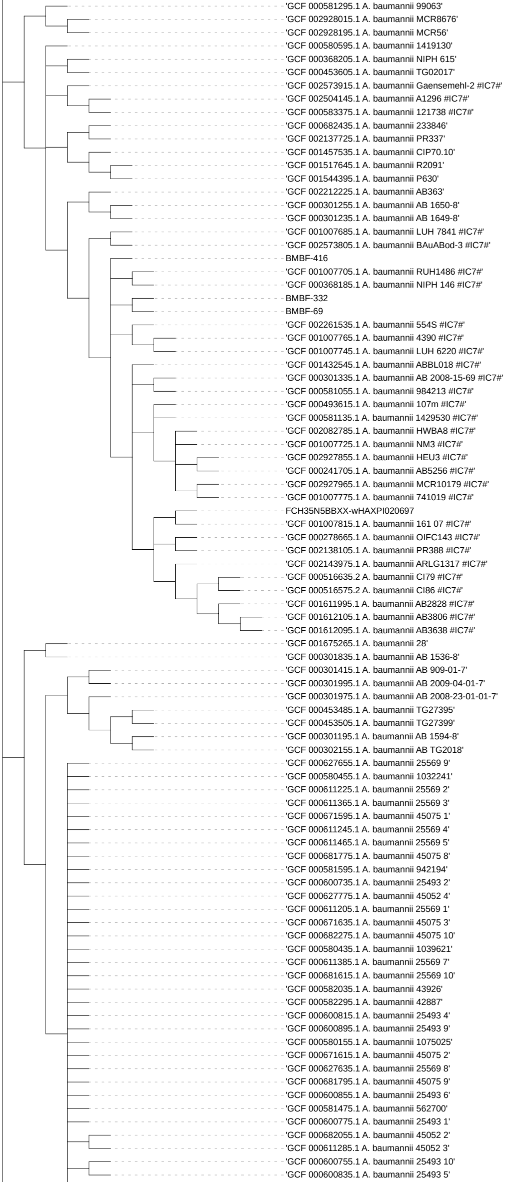

'GCF 000581295.1 A. baumannii 99063'
'GCF 002928015.1 A. baumannii MCR8676'
'GCF 002928195.1 A. baumannii MCR56'
'GCF 000580595.1 A. baumannii 1419130'
'GCF 000368205.1 A. baumannii NIPH 615'
'GCF 000453605.1 A. baumannii TG02017'
'GCF 002573915.1 A. baumannii Gaensemehl-2 #IC7#'
'GCF 002504145.1 A. baumannii A1296 #IC7#'
'GCF 000583375.1 A. baumannii 121738 #IC7#'
'GCF 000682435.1 A. baumannii 233846'
'GCF 002137725.1 A. baumannii PR337'
'GCF 001457535.1 A. baumannii CIP70.10'
'GCF 001517645.1 A. baumannii R2091'
'GCF 001544395.1 A. baumannii P630'
'GCF 002212225.1 A. baumannii AB363'
'GCF 000301255.1 A. baumannii AB 1650-8'
'GCF 000301235.1 A. baumannii AB 1649-8'
'GCF 001007685.1 A. baumannii LUH 7841 #IC7#'
'GCF 002573805.1 A. baumannii BAuABod-3 #IC7#'
BMBF-416
'GCF 001007705.1 A. baumannii RUH1486 #IC7#'
'GCF 000368185.1 A. baumannii NIPH 146 #IC7#'
BMBF-332
BMBF-69
'GCF 002261535.1 A. baumannii 554S #IC7#'
'GCF 001007765.1 A. baumannii 4390 #IC7#'
'GCF 001007745.1 A. baumannii LUH 6220 #IC7#'
'GCF 001432545.1 A. baumannii ABBL018 #IC7#'
'GCF 000301335.1 A. baumannii AB 2008-15-69 #IC7#'
'GCF 000581055.1 A. baumannii 984213 #IC7#'
'GCF 000493615.1 A. baumannii 107m #IC7#'
'GCF 000581135.1 A. baumannii 1429530 #IC7#'
'GCF 002082785.1 A. baumannii HWBA8 #IC7#'
'GCF 001007725.1 A. baumannii NM3 #IC7#'
'GCF 002927855.1 A. baumannii HEU3 #IC7#'
'GCF 000241705.1 A. baumannii AB5256 #IC7#'
'GCF 002927965.1 A. baumannii MCR10179 #IC7#'
'GCF 001007775.1 A. baumannii 741019 #IC7#'
FCH35N5BBXX-wHAXPI020697
'GCF 001007815.1 A. baumannii 161 07 #IC7#'
'GCF 000278665.1 A. baumannii OIFC143 #IC7#'
'GCF 002138105.1 A. baumannii PR388 #IC7#'
'GCF 002143975.1 A. baumannii ARLG1317 #IC7#'
'GCF 000516635.2 A. baumannii CI79 #IC7#'
'GCF 000516575.2 A. baumannii CI86 #IC7#'
'GCF 001611995.1 A. baumannii AB2828 #IC7#'
'GCF 001612105.1 A. baumannii AB3806 #IC7#'
'GCF 001612095.1 A. baumannii AB3638 #IC7#'
'GCF 001675265.1 A. baumannii 28'
'GCF 000301835.1 A. baumannii AB 1536-8'
'GCF 000301415.1 A. baumannii AB 909-01-7'
'GCF 000301995.1 A. baumannii AB 2009-04-01-7'
'GCF 000301975.1 A. baumannii AB 2008-23-01-01-7'
'GCF 000453485.1 A. baumannii TG27395'
'GCF 000453505.1 A. baumannii TG27399'
'GCF 000301195.1 A. baumannii AB 1594-8'
'GCF 000302155.1 A. baumannii AB TG2018'
'GCF 000627655.1 A. baumannii 25569 9'
'GCF 000580455.1 A. baumannii 1032241'
'GCF 000611225.1 A. baumannii 25569 2'
'GCF 000611365.1 A. baumannii 25569 3'
'GCF 000671595.1 A. baumannii 45075 1'
'GCF 000611245.1 A. baumannii 25569 4'
'GCF 000611465.1 A. baumannii 25569 5'
'GCF 000681775.1 A. baumannii 45075 8'
'GCF 000581595.1 A. baumannii 942194'
'GCF 000600735.1 A. baumannii 25493 2'
'GCF 000627775.1 A. baumannii 45052 4'
'GCF 000611205.1 A. baumannii 25569 1'
'GCF 000671635.1 A. baumannii 45075 3'
'GCF 000682275.1 A. baumannii 45075 10'
'GCF 000580435.1 A. baumannii 1039621'
'GCF 000611385.1 A. baumannii 25569 7'
'GCF 000681615.1 A. baumannii 25569 10'
'GCF 000582035.1 A. baumannii 43926'
'GCF 000582295.1 A. baumannii 42887'
'GCF 000600815.1 A. baumannii 25493 4'
'GCF 000600895.1 A. baumannii 25493 9'
'GCF 000580155.1 A. baumannii 1075025'
'GCF 000671615.1 A. baumannii 45075 2'
'GCF 000627635.1 A. baumannii 25569 8'
'GCF 000681795.1 A. baumannii 45075 9'
'GCF 000600855.1 A. baumannii 25493 6'
'GCF 000581475.1 A. baumannii 562700'
'GCF 000600775.1 A. baumannii 25493 1'
'GCF 000682055.1 A. baumannii 45052 2'
'GCF 000611285.1 A. baumannii 45052 3'
'GCF 000600755.1 A. baumannii 25493 10'
'GCF 000600835.1 A. baumannii 25493 5'

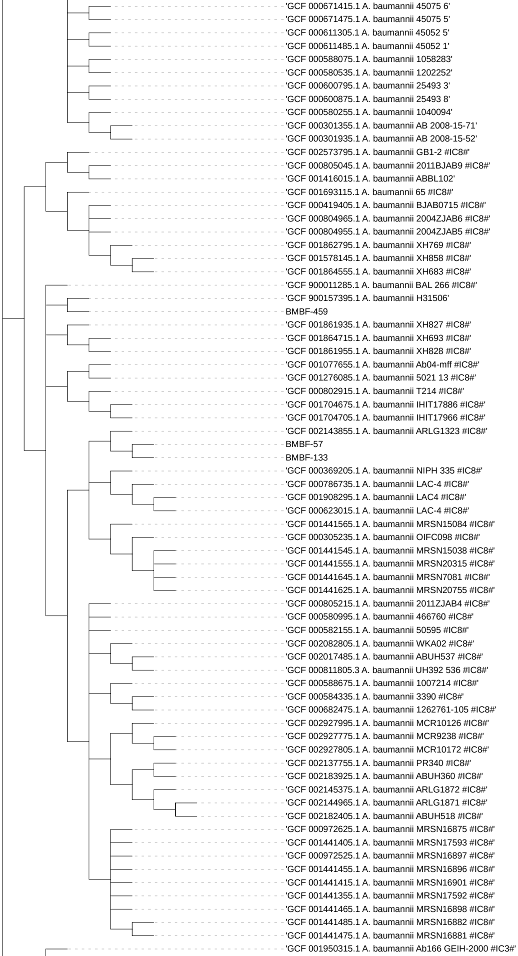

'GCF 000671415.1 A. baumannii 45075 6'
'GCF 000671475.1 A. baumannii 45075 5'
'GCF 000611305.1 A. baumannii 45052 5'
'GCF 000611485.1 A. baumannii 45052 1'
'GCF 000588075.1 A. baumannii 1058283'
'GCF 000580535.1 A. baumannii 1202252'
'GCF 000600795.1 A. baumannii 25493 3'
'GCF 000600875.1 A. baumannii 25493 8'
'GCF 000580255.1 A. baumannii 1040094'
'GCF 000301355.1 A. baumannii AB 2008-15-71'
'GCF 000301935.1 A. baumannii AB 2008-15-52'
'GCF 002573795.1 A. baumannii GB1-2 #IC8#'
'GCF 000805045.1 A. baumannii 2011BJAB9 #IC8#'
'GCF 001416015.1 A. baumannii ABBL102'
'GCF 001693115.1 A. baumannii 65 #IC8#'
'GCF 000419405.1 A. baumannii BJAB0715 #IC8#'
'GCF 000804965.1 A. baumannii 2004ZJAB6 #IC8#'
'GCF 000804955.1 A. baumannii 2004ZJAB5 #IC8#'
'GCF 001862795.1 A. baumannii XH769 #IC8#'
'GCF 001578145.1 A. baumannii XH858 #IC8#'
'GCF 001864555.1 A. baumannii XH683 #IC8#'
'GCF 900011285.1 A. baumannii BAL 266 #IC8#'
'GCF 900157395.1 A. baumannii H31506'
BMBF-459
'GCF 001861935.1 A. baumannii XH827 #IC8#'
'GCF 001864715.1 A. baumannii XH693 #IC8#'
'GCF 001861955.1 A. baumannii XH828 #IC8#'
'GCF 001077655.1 A. baumannii Ab04-mff #IC8#'
'GCF 001276085.1 A. baumannii 5021 13 #IC8#'
'GCF 000802915.1 A. baumannii T214 #IC8#'
'GCF 001704675.1 A. baumannii IHIT17886 #IC8#'
'GCF 001704705.1 A. baumannii IHIT17966 #IC8#'
'GCF 002143855.1 A. baumannii ARLG1323 #IC8#'
BMBF-57
BMBF-133
'GCF 000369205.1 A. baumannii NIPH 335 #IC8#'
'GCF 000786735.1 A. baumannii LAC-4 #IC8#'
'GCF 001908295.1 A. baumannii LAC4 #IC8#'
'GCF 000623015.1 A. baumannii LAC-4 #IC8#'
'GCF 001441565.1 A. baumannii MRSN15084 #IC8#'
'GCF 000305235.1 A. baumannii OIFC098 #IC8#'
'GCF 001441545.1 A. baumannii MRSN15038 #IC8#'
'GCF 001441555.1 A. baumannii MRSN20315 #IC8#'
'GCF 001441645.1 A. baumannii MRSN7081 #IC8#'
'GCF 001441625.1 A. baumannii MRSN20755 #IC8#'
'GCF 000805215.1 A. baumannii 2011ZJAB4 #IC8#'
'GCF 000580995.1 A. baumannii 466760 #IC8#'
'GCF 000582155.1 A. baumannii 50595 #IC8#'
'GCF 002082805.1 A. baumannii WKA02 #IC8#'
'GCF 002017485.1 A. baumannii ABUH537 #IC8#'
'GCF 000811805.3 A. baumannii UH392 536 #IC8#'
'GCF 000588675.1 A. baumannii 1007214 #IC8#'
'GCF 000584335.1 A. baumannii 3390 #IC8#'
'GCF 000682475.1 A. baumannii 1262761-105 #IC8#'
'GCF 002927995.1 A. baumannii MCR10126 #IC8#'
'GCF 002927775.1 A. baumannii MCR9238 #IC8#'
'GCF 002927805.1 A. baumannii MCR10172 #IC8#'
'GCF 002137755.1 A. baumannii PR340 #IC8#'
'GCF 002183925.1 A. baumannii ABUH360 #IC8#'
'GCF 002145375.1 A. baumannii ARLG1872 #IC8#'
'GCF 002144965.1 A. baumannii ARLG1871 #IC8#'
'GCF 002182405.1 A. baumannii ABUH518 #IC8#'
'GCF 000972625.1 A. baumannii MRSN16875 #IC8#'
'GCF 001441405.1 A. baumannii MRSN17593 #IC8#'
'GCF 000972525.1 A. baumannii MRSN16897 #IC8#'
'GCF 001441455.1 A. baumannii MRSN16896 #IC8#'
'GCF 001441415.1 A. baumannii MRSN16901 #IC8#'
'GCF 001441355.1 A. baumannii MRSN17592 #IC8#'
'GCF 001441465.1 A. baumannii MRSN16898 #IC8#'
'GCF 001441485.1 A. baumannii MRSN16882 #IC8#'
'GCF 001441475.1 A. baumannii MRSN16881 #IC8#'
'GCF 001950315.1 A. baumannii Ab166 GEIH-2000 #IC3#'

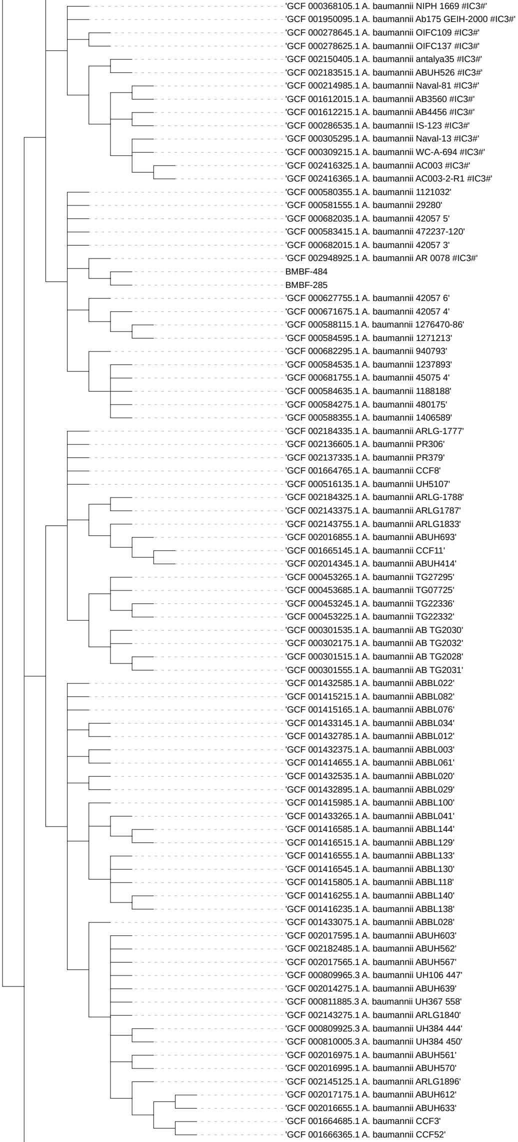
'GCF 000368105.1 A. baumannii NIPH 1669 #IC3#'
'GCF 001950095.1 A. baumannii Ab175 GEIH-2000 #IC3#'
'GCF 000278645.1 A. baumannii OIFC109 #IC3#'
'GCF 000278625.1 A. baumannii OIFC137 #IC3#'
'GCF 002150405.1 A. baumannii antalya35 #IC3#'
'GCF 002183515.1 A. baumannii ABUH526 #IC3#'
'GCF 000214985.1 A. baumannii Naval-81 #IC3#'
'GCF 001612015.1 A. baumannii AB3560 #IC3#'
'GCF 001612215.1 A. baumannii AB4456 #IC3#'
'GCF 000286535.1 A. baumannii IS-123 #IC3#'
'GCF 000305295.1 A. baumannii Naval-13 #IC3#'
'GCF 000309215.1 A. baumannii WC-A-694 #IC3#'
'GCF 002416325.1 A. baumannii AC003 #IC3#'
'GCF 002416365.1 A. baumannii AC003-2-R1 #IC3#'
'GCF 000580355.1 A. baumannii 1121032'
'GCF 000581555.1 A. baumannii 29280'
'GCF 000682035.1 A. baumannii 42057 5'
'GCF 000583415.1 A. baumannii 472237-120'
'GCF 000682015.1 A. baumannii 42057 3'
'GCF 002948925.1 A. baumannii AR 0078 #IC3#'
BMBF-484
BMBF-285
'GCF 000627755.1 A. baumannii 42057 6'
'GCF 000671675.1 A. baumannii 42057 4'
'GCF 000588115.1 A. baumannii 1276470-86'
'GCF 000584595.1 A. baumannii 1271213'
'GCF 000682295.1 A. baumannii 940793'
'GCF 000584535.1 A. baumannii 1237893'
'GCF 000681755.1 A. baumannii 45075 4'
'GCF 000584635.1 A. baumannii 1188188'
'GCF 000584275.1 A. baumannii 480175'
'GCF 000588355.1 A. baumannii 1406589'
'GCF 002184335.1 A. baumannii ARLG-1777'
'GCF 002136605.1 A. baumannii PR306'
'GCF 002137335.1 A. baumannii PR379'
'GCF 001664765.1 A. baumannii CCF8'
'GCF 000516135.1 A. baumannii UH5107'
'GCF 002184325.1 A. baumannii ARLG-1788'
'GCF 002143375.1 A. baumannii ARLG1787'
'GCF 002143755.1 A. baumannii ARLG1833'
'GCF 002016855.1 A. baumannii ABUH693'
'GCF 001665145.1 A. baumannii CCF11'
'GCF 002014345.1 A. baumannii ABUH414'
'GCF 000453265.1 A. baumannii TG27295'
'GCF 000453685.1 A. baumannii TG07725'
'GCF 000453245.1 A. baumannii TG22336'
'GCF 000453225.1 A. baumannii TG22332'
'GCF 000301535.1 A. baumannii AB TG2030'
'GCF 000302175.1 A. baumannii AB TG2032'
'GCF 000301515.1 A. baumannii AB TG2028'
'GCF 000301555.1 A. baumannii AB TG2031'
'GCF 001432585.1 A. baumannii ABBL022'
'GCF 001415215.1 A. baumannii ABBL082'
'GCF 001415165.1 A. baumannii ABBL076'
'GCF 001433145.1 A. baumannii ABBL034'
'GCF 001432785.1 A. baumannii ABBL012'
'GCF 001432375.1 A. baumannii ABBL003'
'GCF 001414655.1 A. baumannii ABBL061'
'GCF 001432535.1 A. baumannii ABBL020'
'GCF 001432895.1 A. baumannii ABBL029'
'GCF 001415985.1 A. baumannii ABBL100'
'GCF 001433265.1 A. baumannii ABBL041'
'GCF 001416585.1 A. baumannii ABBL144'
'GCF 001416515.1 A. baumannii ABBL129'
'GCF 001416555.1 A. baumannii ABBL133'
'GCF 001416545.1 A. baumannii ABBL130'
'GCF 001415805.1 A. baumannii ABBL118'
'GCF 001416255.1 A. baumannii ABBL140'
'GCF 001416235.1 A. baumannii ABBL138'
'GCF 001433075.1 A. baumannii ABBL028'
'GCF 002017595.1 A. baumannii ABUH603'
'GCF 002182485.1 A. baumannii ABUH562'
'GCF 002017565.1 A. baumannii ABUH567'
'GCF 000809965.3 A. baumannii UH106 447'
'GCF 002014275.1 A. baumannii ABUH639'
'GCF 000811885.3 A. baumannii UH367 558'
'GCF 002143275.1 A. baumannii ARLG1840'
'GCF 000809925.3 A. baumannii UH384 444'
'GCF 000810005.3 A. baumannii UH384 450'
'GCF 002016975.1 A. baumannii ABUH561'
'GCF 002016995.1 A. baumannii ABUH570'
'GCF 002145125.1 A. baumannii ARLG1896'
'GCF 002017175.1 A. baumannii ABUH612'
'GCF 002016655.1 A. baumannii ABUH633'
'GCF 001664685.1 A. baumannii CCF3'
'GCF 001666365.1 A. baumannii CCF52'

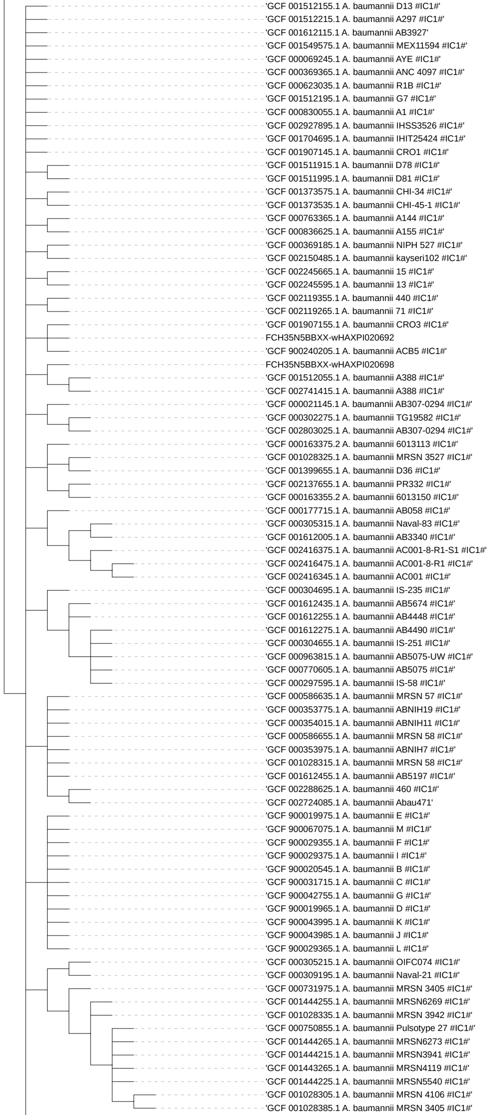
'GCF 001512155.1 A. baumannii D13 #IC1#'
'GCF 001512215.1 A. baumannii A297 #IC1#'
'GCF 001612115.1 A. baumannii AB3927'
'GCF 001549575.1 A. baumannii MEX11594 #IC1#'
'GCF 000069245.1 A. baumannii AYE #IC1#'
'GCF 000369365.1 A. baumannii ANC 4097 #IC1#'
'GCF 000623035.1 A. baumannii R1B #IC1#'
'GCF 001512195.1 A. baumannii G7 #IC1#'
'GCF 000830055.1 A. baumannii A1 #IC1#'
'GCF 002927895.1 A. baumannii IHSS3526 #IC1#'
'GCF 001704695.1 A. baumannii IHIT25424 #IC1#'
'GCF 001907145.1 A. baumannii CRO1 #IC1#'
'GCF 001511915.1 A. baumannii D78 #IC1#'
'GCF 001511995.1 A. baumannii D81 #IC1#'
'GCF 001373575.1 A. baumannii CHI-34 #IC1#'
'GCF 001373535.1 A. baumannii CHI-45-1 #IC1#'
'GCF 000763365.1 A. baumannii A144 #IC1#'
'GCF 000836625.1 A. baumannii A155 #IC1#'
'GCF 000369185.1 A. baumannii NIPH 527 #IC1#'
'GCF 002150485.1 A. baumannii kayseri102 #IC1#'
'GCF 002245665.1 A. baumannii 15 #IC1#'
'GCF 002245595.1 A. baumannii 13 #IC1#'
'GCF 002119355.1 A. baumannii 440 #IC1#'
'GCF 002119265.1 A. baumannii 71 #IC1#'
'GCF 001907155.1 A. baumannii CRO3 #IC1#'
FCH35N5BBXX-wHAXPI020692
'GCF 900240205.1 A. baumannii ACB5 #IC1#'
FCH35N5BBXX-wHAXPI020698
'GCF 001512055.1 A. baumannii A388 #IC1#'
'GCF 002741415.1 A. baumannii A388 #IC1#'
'GCF 000021145.1 A. baumannii AB307-0294 #IC1#'
'GCF 000302275.1 A. baumannii TG19582 #IC1#'
'GCF 002803025.1 A. baumannii AB307-0294 #IC1#'
'GCF 000163375.2 A. baumannii 6013113 #IC1#'
'GCF 001028325.1 A. baumannii MRSN 3527 #IC1#'
'GCF 001399655.1 A. baumannii D36 #IC1#'
'GCF 002137655.1 A. baumannii PR332 #IC1#'
'GCF 000163355.2 A. baumannii 6013150 #IC1#'
'GCF 000177715.1 A. baumannii AB058 #IC1#'
'GCF 000305315.1 A. baumannii Naval-83 #IC1#'
'GCF 001612005.1 A. baumannii AB3340 #IC1#'
'GCF 002416375.1 A. baumannii AC001-8-R1-S1 #IC1#'
'GCF 002416475.1 A. baumannii AC001-8-R1 #IC1#'
'GCF 002416345.1 A. baumannii AC001 #IC1#'
'GCF 000304695.1 A. baumannii IS-235 #IC1#'
'GCF 001612435.1 A. baumannii AB5674 #IC1#'
'GCF 001612255.1 A. baumannii AB4448 #IC1#'
'GCF 001612275.1 A. baumannii AB4490 #IC1#'
'GCF 000304655.1 A. baumannii IS-251 #IC1#'
'GCF 000963815.1 A. baumannii AB5075-UW #IC1#'
'GCF 000770605.1 A. baumannii AB5075 #IC1#'
'GCF 000297595.1 A. baumannii IS-58 #IC1#'
'GCF 000586635.1 A. baumannii MRSN 57 #IC1#'
'GCF 000353775.1 A. baumannii ABNIH19 #IC1#'
'GCF 000354015.1 A. baumannii ABNIH11 #IC1#'
'GCF 000586655.1 A. baumannii MRSN 58 #IC1#'
'GCF 000353975.1 A. baumannii ABNIH7 #IC1#'
'GCF 001028315.1 A. baumannii MRSN 58 #IC1#'
'GCF 001612455.1 A. baumannii AB5197 #IC1#'
'GCF 002288625.1 A. baumannii 460 #IC1#'
'GCF 002724085.1 A. baumannii Abau471'
'GCF 900019975.1 A. baumannii E #IC1#'
'GCF 900067075.1 A. baumannii M #IC1#'
'GCF 900029355.1 A. baumannii F #IC1#'
'GCF 900029375.1 A. baumannii I #IC1#'
'GCF 900020545.1 A. baumannii B #IC1#'
'GCF 900031715.1 A. baumannii C #IC1#'
'GCF 900042755.1 A. baumannii G #IC1#'
'GCF 900019965.1 A. baumannii D #IC1#'
'GCF 900043995.1 A. baumannii K #IC1#'
'GCF 900043985.1 A. baumannii J #IC1#'
'GCF 900029365.1 A. baumannii L #IC1#'
'GCF 000305215.1 A. baumannii OIFC074 #IC1#'
'GCF 000309195.1 A. baumannii Naval-21 #IC1#'
'GCF 000731975.1 A. baumannii MRSN 3405 #IC1#'
'GCF 001444255.1 A. baumannii MRSN6269 #IC1#'
'GCF 001028335.1 A. baumannii MRSN 3942 #IC1#'
'GCF 000750855.1 A. baumannii Pulsotype 27 #IC1#'
'GCF 001444265.1 A. baumannii MRSN6273 #IC1#'
'GCF 001444215.1 A. baumannii MRSN3941 #IC1#'
'GCF 001443265.1 A. baumannii MRSN4119 #IC1#'
'GCF 001444225.1 A. baumannii MRSN5540 #IC1#'
'GCF 001028305.1 A. baumannii MRSN 4106 #IC1#'
'GCF 001028385.1 A. baumannii MRSN 3405 #IC1#'

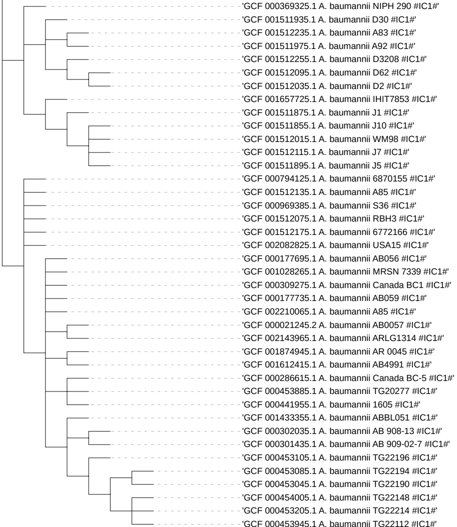

'GCF 000369325.1 A. baumannii NIPH 290 #IC1#'
'GCF 001511935.1 A. baumannii D30 #IC1#'
'GCF 001512235.1 A. baumannii A83 #IC1#'
'GCF 001511975.1 A. baumannii A92 #IC1#'
'GCF 001512255.1 A. baumannii D3208 #IC1#'
'GCF 001512095.1 A. baumannii D62 #IC1#'
'GCF 001512035.1 A. baumannii D2 #IC1#'
'GCF 001657725.1 A. baumannii IHIT7853 #IC1#'
'GCF 001511875.1 A. baumannii J1 #IC1#'
'GCF 001511855.1 A. baumannii J10 #IC1#'
'GCF 001512015.1 A. baumannii WM98 #IC1#'
'GCF 001512115.1 A. baumannii J7 #IC1#'
'GCF 001511895.1 A. baumannii J5 #IC1#'
'GCF 000794125.1 A. baumannii 6870155 #IC1#'
'GCF 001512135.1 A. baumannii A85 #IC1#'
'GCF 000969385.1 A. baumannii S36 #IC1#'
'GCF 001512075.1 A. baumannii RBH3 #IC1#'
'GCF 001512175.1 A. baumannii 6772166 #IC1#'
'GCF 002082825.1 A. baumannii USA15 #IC1#'
'GCF 000177695.1 A. baumannii AB056 #IC1#'
'GCF 001028265.1 A. baumannii MRSN 7339 #IC1#'
'GCF 000309275.1 A. baumannii Canada BC1 #IC1#'
'GCF 000177735.1 A. baumannii AB059 #IC1#'
'GCF 002210065.1 A. baumannii A85 #IC1#'
'GCF 000021245.2 A. baumannii AB0057 #IC1#'
'GCF 002143965.1 A. baumannii ARLG1314 #IC1#'
'GCF 001874945.1 A. baumannii AR 0045 #IC1#'
'GCF 001612415.1 A. baumannii AB4991 #IC1#'
'GCF 000286615.1 A. baumannii Canada BC-5 #IC1#'
'GCF 000453885.1 A. baumannii TG20277 #IC1#'
'GCF 000441955.1 A. baumannii 1605 #IC1#'
'GCF 001433355.1 A. baumannii ABBL051 #IC1#'
'GCF 000302035.1 A. baumannii AB 908-13 #IC1#'
'GCF 000301435.1 A. baumannii AB 909-02-7 #IC1#'
'GCF 000453105.1 A. baumannii TG22196 #IC1#'
'GCF 000453085.1 A. baumannii TG22194 #IC1#'
'GCF 000453045.1 A. baumannii TG22190 #IC1#'
'GCF 000454005.1 A. baumannii TG22148 #IC1#'
'GCF 000453205.1 A. baumannii TG22214 #IC1#'
'GCF 000453945.1 A. baumannii TG22112 #IC1#'
