# Supplementary material for: Evolutionarily stable gene clusters shed light on the common grounds of pathogenicity in the Acinetobacter calcoaceticus-baumannii complex
Source: PLoS Genet. 2022 Jun 2;18(6):e1010020. doi: 10.1371/journal.pgen.1010020 (PMC9162365; doi:10.1371/journal.pgen.1010020)
Supplement: Attachment — Submitted filename: Answer2reviewers_v1-submit.docx [file pgen.1010020.s030.docx]

# Answer to the reviewers’ comments

Before we address the individual points raised by the two reviewers, we would like to thank both for taking the time to critically assess our manuscript, and for the very careful and constructive review. We have organized our response in the following way.

- We first provide the *reviewer comment in italics using blue font color*. To allow a better tracking, we have enumerated the individual comments.
- You will find our response directly below the comment in normal font (black)
- In case, we changed or added text in the manuscript when following the reviewers’ suggestion, we provide the new text below our answer starting with ‘We write now:’ *followed by the new text in italics (black).*

Please note, we provide no text if we amended the writing style or corrected typos or incorrect punctuation.

We hope that this structure helps to streamline the re-review.

Sincerely,

Ingo Ebersberger (on behalf of all authors)

## Reviewer 1

General comments:
*GC 1 – A phenotypic conformation was achieved for one of their findings, kynurine utilisation, but it seems rather preliminary. Although this may have been adequate if it was performed for multiple findings across the manuscript, such as QS, I believe further controls for kynurine utilisation are appropriate, as detailed below.*
We agree with this comment, and we have added additional experiments to better characterize the kynurenine cluster. Please see our answer to *SC 31* for details. Still, we would like to bring to the attention of the reviewer that the main focus of this study is *in silico*. Although we would have wished to perform experiments that shed light on many of the promising gene clusters we have described here, this was beyond the scope of this study.

*GC 2 – Consider dampening the conclusions regarding associations between gene presence/absence and a role in virulence. The results are indicative of these roles.*
We went through the text and rephrased parts that give the impression that we are overly confident with respect to the role of the gene clusters we describe. It was never our intention to go beyond an indication. We are very well aware of the limitations of our study when it comes to the interpretation of the results in the context of bacterial virulence. It is our hope that follow-up studies take up the baton and test the role of particularly promising gene clusters in bacterial virulence.

*GC 3 - The numbering of the supplementary material is confusing.*
We regret that the numbering appears not really accessible.

- Concerning the layout of the numbering, we followed the submission guidelines specified on the [PLoS Genetics web page](https://journals.plos.org/plosgenetics/s/submission-guidelines). Specifically, it reads there:

Thus, instead of writing Fig. S1A, we write S1 Figure A, for example. We further numbered supplementary tables, figures, text and data in the order they appear in the main text. If the manuscript gets accepted, we will be in touch with the production team to rule out any misunderstandings here.

- We noted that the order in which few SMs were mentioned was wrong in individual cases. This has been corrected now, and we apologize for the confusion.

*GC 4 – Please don’t insert too many sentence breaks, such as seen in line 37, it restricts readability.*
Thank you very much for pointing this out. We have now revised the manuscript with this suggestion in mind. To give one example, we have rephrased the corresponding sentence. We write now:
*“We shortlisted 150 ESGCs that either co-emerged with the pathogenic ACB clade or are preferentially found therein.”*

Specific Comments:
*SC 1 – Line 29: The functional context (eg GO) was largely the means of study in this manuscript too.*
The reviewer refers to the sentence in the abstract where we have stated “Yet, existing studies are limited in (…) resolution by largely analyzing genes isolated from their functional contexts.” It appears that our wording was not precise enough. When we wrote ‘functional context’, we had functionally integrated proteins from the same gene cluster in mind. We have rephrased the sentence and write now:

“*Yet, existing studies are limited in (…) resolution by largely analyzing genes independent of their organization in functional gene clusters.”*

*SC 2 – Line 32: no italics for atcc 19606*
corrected

*SC 3 – Line 43: Improve clarity on “show”, eg bioinformatic vs phenotypic.*
We agree and added “experimentally”, to make the intent of show clear. We write now:

*“Specifically, we could show experimentally that members of the ACB clade can use kynurenine as a sole carbon and energy source (…)”*

*SC 4 – Line 47: Instead consider emphasizing that this study represents a very comprehensive resource for future research into novel therapeutic strategies.*
Here, we have to guess the intention of the reviewer, since the ‘instead’ leaves us a bit lost. We wrote in line 47 “They (the rich and unbiased set of novel testable hypotheses mentioned in the previous sentence) disclose promising routes for future therapeutic strategies”. Following the reviewer’s suggestion, we have rephrased this last sentence and write now:

*“In summary, this study provides a rich and unbiased set of novel testable hypotheses on how pathogenic Acinetobacter interact with and ultimately infect their human host. It is a comprehensive resource for future research into novel therapeutic strategies.”.*

*SC 5 – Line 54: Dampen the word “essential”*
We rephrased the sentence and write now:

*“Understanding the genetics of host colonization and infection can substantially help in devising novel ways of treatment.”*

*SC 6 – Line 58-59: Remove “comprising the A. baumannii-calcoaceticus complex”*
Done

*SC 7 – Line 65-66: this reads clunky, please revise.*
We have rephrased this sentence. We write now:

*“Humans produce this substance to contain bacterial invaders and to fine-tune the innate immune response. But A. baumannii and closely related pathogens found a way to feed on kynurenine. This suggests that the pathogens might be able to dysregulate the human immune response.”*

*SC 8 – Line 73: Delete “with”*
Done

*SC 9 - Line 77: Revise this sentence.*
The sentence was rephrased. We write now:

*“Acinetobacter baumannii is the most critical member of the ACB complex. On a global scale, this species alone signs responsible for up to 5% of the total bacterial infections in hospitals.”*

*SC 10 – Line 83: Please include additional info on international clones, consider providing inforamtion regarding IC8 in particular.*
We added two short sentences to explain the clonal nature of Acinetobacter and the concept of international clones. Unfortunately, characterizing information in the literature regarding international lineage IC 8 or any of its sub-lineages is highly elusive. Specifically, there is not much helpful information on general phenotypic differences between IC8 to other clonal lineages apart from that it is less prevalent compared to ICs 1, 2, and 4. We write now:

*“Many outbreaks worldwide can be attributed to one of eight genetically well distinguishable clonal complexes within the population of A. baumannii, all sharing the resistance against carbapenem [9, 10].”*

*SC 11 – Line 92: The entire section on the three main approaches does not read very well. Please make this more concise and don’t include the numerical list.*

Following the reviewers request, we have rephrased and shortened the description of the three main approaches. We write now:

*Three main approaches have been used to elucidate the molecular basis of Acinetobacter virulence. Candidate approaches have scanned for virulence factors previously characterized in other bacterial pathogens [4, 7, 21-25]. To extend the scope beyond pre-compiled virulence factor catalogs, genome-wide approaches have assessed the effect of gene knockouts on the infection process (e.g. [12, 26]), or have identified the transcriptional changes under conditions the bacterium typically encounters in the human host (e. g. [23, 27]). However, experiments are usually performed only on a small set of model strains (e.g. [28]) and the limited set of tested conditions cannot reflect the diversity of infection sites in the human body. Moreover, factors contributing only indirectly to virulence, such as metabolic pathways that facilitate the tapping of host resources {Eisenreich, 2010 #2096}, are hard to capture. Comparative genomics provide complementary evidences in the search of virulence related traits. The genus Acinetobacter encompasses to date 72 (validly) named and mostly nonpathogenic species [31] isolated from habitats that range from floral nectar to animals [32]. This diversity represents a perfect setup to identify genomic changes that correlate with the evolutionary emergence of the pathogenic potential [30].*

*SC 12 – Line 118: What is the difference between “genome plasticity” and "flexible genome" as described in line 115. This is a bit confusing.*

We agree that this was confusing. We now use only ‘flexible genome’ and write now:

*They indicated that a major driver of A. baumannii’s success as a pathogen is its remarkably flexible genome [11][36] which is characterized by high mutation rates [37] paired with the ability to acquire new, or alter the structure or expression of existing genes [13-15]. This promotes a rapid adaptation to novel and adverse environmental conditions, as well as the spread of antimicrobial resistance determinants [38][39].*

*SC 13 – Line 125: The balance between background and intro to new findings is inadequate. Line 135- 138: Be mindful of repetition between the abstract and this section.*
We have now shortened this paragraph by removing the repetition between the abstract and this section. We write now:

*“Here, we exploit the availability of thousands of Acinetobacter spp. genomes in the public databases (NCBI RefSeq; [44]) to shed light on the evolution of the pathogenic ACB complex at a resolution that extends from a genus-wide overview to the level of individual clonal lineages within A. baumannii. For the first time, we integrate genus-wide ortholog searches with analyses of gene order conservation providing a highly-resolved view on the joint evolutionary fate of neighboring genes using the type strain ATCC 19606 as a reference. This revealed 150 evolutionarily stable gene clusters (ESGC_ACB_) that are prevalent in the ACB complex and rare or absent in the other members of the genus. The functional annotations of these ESGCs provide insights into the genetic and functional specifics of a clade comprising mostly pathogens, and thus direct the focus to key processes likely relevant for the adaptation of the bacterium to the human host. We find that the ACB complex acquired novel genetic modules for the regulation and formation of biofilms, for the scavenging of micronutrients, and have substantially extended their capabilities to exploit a diverse set of carbon sources.”*

*SC 14 – Line 140-145: Probably too much detail and this has now transitioned to background again.*
We agree with the criticism, and we have now removed the corresponding sentences from the introduction (see our answer to *SC 13*)

*SC 15 – S1 Table: I can only find 38 A. nosocomialis, whilst it indicates 69 in Figure 5, please clarify.*
Table S1 lists the downloaded source material featuring the original (species) labels for reference. In the section “Consistency-based phylogeny of the genus *Acinetobacter*” we describe our approach to a) reassign unlabeled genomes (i.e. *Acinetobacter sp*.) and b) correct falsely labeled (i.e. genomes with wrong species assignments) within the ACB complex, which allowed to increase sample size for *A. nosocomialis*. The results are detailed in Table S4. For a visual representation we like to refer to Figure S3 (phylogeny).

*SC 16 – Fig 1: This figure serves no primary function in this manuscript. Please move to sup material.*
We have placed this figure into the main manuscript to direct the attention of the readers to the aspect that our data can and should be re-used. If there are no strong arguments against it, we would like to keep it there. We now have added the following sentences to the manuscript that briefly explain the relevance of the Aci-Dash, and we hope that the reviewer follows our intention here. We write now:

*“The Acinetobacter-Dashboard*

*“Acinetobacter research worldwide benefits from the FAIR principle where scientific data is findable, accessible, interoperable and reproducible {Wilkinson, 2016 #2746}. As a first step in this direction, we have developed the web application Aci-Dash (https://aci-dash.ingress.rancher.computational.bio/), which currently hosts the 232 strains of Set-R (Fig. 1). For each strain, the user can obtain information about the sample origin and get access to all genes annotated in the respective genome together with an overview of its abundance in the other 231 strains. For example, this allows the rapid identification of genes that are specific to a strain, a clade, or that are part of the core genome. Moreover, genes can be subselected based on their genomic position, which allows to explore the phylogenetic profiles of neighboring genes. Interactive plots make it straightforward to retrieve further information about individual or groups of genes, such as their assignment to COG or KO groups, or their representation in virulence databases (see above). Thus, Aci-Dash is the first web-based platform to interactively browse and explore the Acinetobacter pan-genome that is spanned by the 232 strains of Set-R.”*

*SC 17 – Line 209: Using both However and though in one sentence does not aid readability.*
Corrected

*SC 18 – Line 213: Please include a ref to 2C when covering environment/host presence.*
Done

*SC 19 – Line 217-221: Please revise wording*
We rephrased this part and write now:

*“In the species that diverged after A. baumannii last shared a common ancestor with A. baylyi (Ab clade) and with A. haemolyticus (HA clade), we find increasingly often human pathogens. This suggests a progressive adaptation to humans as a host [59].”*

*SC 20 - Line 226: Remove “at least”*
Done

*SC 21 – Line 241-242: Please refer to figure/table and/or name these.*
We refer now to table S1

*SC 22 – Line 332: Why are CsuC and D conserved and not lost?*
We assume that the reviewer refers to Fig. 7B which seems to indicate that CsuC and CsuD are present in the non-ACB clade. This is an artefact of the ortholog search where two paralogs, prpC and prpD have been picked up. We had commented on this explicitly in the figure caption where we write

*“Note the deviating abundance pattern for CsuC and CsuD, which is due to the presence of* *prpC and prpD, two paralogous genes from the photoregulated pilus ABCD (prpABCD), in the respective orthologous groups. Microsynteny analyses confirmed that the entire Csu cluster forms one evolutionary unit (S1 Data id:0410).”*

*SC 23 – Line 332: This does not appear true for the “L” and “CA” clades, please verify.*
This is correct and should have been made clearer. We write now:

*“With few exceptions, ESGC_ACB_-0162 and ESGC_ACB_-0410 share similar abundance patterns (cf. Fig 5). This is in line with the finding that the regulation of the Csu cluster is under the direct control of Ac-505 [28, 67]. Thus, Ac-505 likely acts as a modulator between biofilm formation on abiotic and biotic surfaces. However, contrary to the QS_Lux_ - NRPS supercluster, the Csu cluster was lost multiple times independently in the ACB clade, e.g. in the CA and the L clades (Fig 5).”*

*SC 24 – Line 359: This intro seems a bit lengthy, consider moving this material to the background section.*
We have revised the sentences to reduce length and remove introductory tone. We write now:

*“Essential metals, such as iron and zinc, are actively sequestered by the host to starve invading pathogens [79]. This likely results in a strong selective pressure for the pathogenic ACB clade to optimize scavenging systems such that the reduced bioavailability of these metal ions in the host can be counterbalanced.”*

*SC 25 – Line 390: Please use abbreviations if introduced earlier.*
Done

*SC 26 – Line 392: Include full stop after (Fig 8)*
Done

*SC 27 – Line 401: Why is the charge included here but not for Zn?*
We replaced Zn with Zn^2+^ where it was appropriate.

*SC 28 – Line 416: Or inhibit niches that minimize ROS exposure?*
This is a very interesting hypothesis that may explain how bacteria may still survive in the host when they appear to have lost the Mn-dependent ability to handle oxidative stress. However, this is a sub-scenario of the case when they actually “are more vulnerable to ROS”, which we have mentioned in the manuscript enlisted. We prefer to not go further into detail here, as we fear that this would become too speculative.

*SC 29 – Line 434: Individual = specific/various?*
We replace “individual” with “few”. We write now:

*“Glucose and gluconate serve as carbon and energy sources for few species in the genus Acinetobacter”*

*SC 30 – Line 466: Should this be “do”?*
Yes, thank you. This has been corrected

*SC 31 – Line 551: Controls such as complementation and growth in the presence of tryptophan are needed.*

We have now added the suggested experiments. We have generated two knock-out lines where we complement the deleted Kyn-cluster. We now show that the complementation restores growth on Kynurenine. Moreover, we show that both the wild type and the Kyn-KO strain grow equally good on tryptophan. This is in line with our observation that there is no identifiable enzyme encoded in the *A. baumannii* genome that can catalyze the formation of tryptophane to formyl-kynurenine. Moreover, it reveals that tryptophan degradation is accomplished via a different pathway. We have added now the following information to the manuscript:

- Main manuscript Fig. 10 – This figure provides now as Panel D the growth on Kynurenine 2.5 mM and Anthranilic acid 2.5 mM for the following strains: Ab04, Ab04 Δkyn, as well as on two complementation strains Ab04 Δkyn::kyn. We write in the figure caption:

*"The kynurenine cluster is necessary and sufficient for growth on kynurenine but not on anthranilic acid. Growth of Ab04, Ab04 Δkyn, and of two Ab04 Δkyn::kyn strains (C1 and C2) on minimal medium supplemented with kynurenine (top) and anthranilate (bottom).”*

- With respect to growth on tryptophane, we have added Supplementary Fig. 8 and write

“*A deletion of the Kyn-cluster abolished growth on kynurenine but neither on anthranilate, the product of KynU (Fig 10D), nor on casamino acid or tryptophan (S8 Fig)*.”

- In the methods section, we now explain the generation of the complementation strains. We write:

*“To generate genetic complementation, the genes deleted in the Ab04 ∆kyn mutant strain were cloned into the pUC18T-mini-Tn7T-Apr vector and introduced to the mutant strain via four-parental mating methods as described previously {Ducas-Mowchun, 2019 #2747}. Briefly, overnight cultures from the recipient strain, HB101(pRK2013) strain, EC100D(pTNS2) strain, and E. coli containing the pUC18T-mini-Tn7T-Apr construct were normalized and mixed 1:1. The suspension was centrifuged, re-suspended in 25 μl of LB, spotted on a pre-warmed LB agar plate and incubated overnight at 37°C. The bacteria were scraped from the plate, resuspended in 1 ml of LB, and plated on LB agar plates supplemented with chloramphenicol and Apramycin to select transconjugants.* Correct insertion of the constructs was verified by PCR amplification and DNA sequencing using the primers listed in S11 Table.*“*

*SC 32 – Further, does competition with more readily available carbon sources influence the activity of Kyn?*
This is an interesting aspect. However, we believe that following this up is beyond the scope of this manuscript. While we could assemble a list of carbon sources that might be more readily available in the human body, we cannot reproduce the nutritional landscape where the Kyn cluster is active. This would render conclusions from such an experiment speculative and hard to generalize. We hope that the reviewer agrees with us that such experiments should be left for a follow up study.

*SC 33 – Line 826: Remove “chosen”*
Done

## Reviewer 2

General comments.

*GC 1 – Several studies highlighted that A. baumannii is able to express several virulence factors, including those required for biofilm formation, desiccation resistance, secretion systems, micronutrient acquisition systems, and twitching motility, as well as adherence to and invasion of human epithelial cells in vitro and in vivo (I draw you attention to some as follows:* [*https://doi.org/10.1128/mSystems.00604-20*](https://doi.org/10.1128/mSystems.00604-20)*;* [*https://doi.org/10.1016/j.ijid.2020.09.352*](https://doi.org/10.1016/j.ijid.2020.09.352)*;* [*https://doi.org/10.1038/ncomms13414*](https://doi.org/10.1038/ncomms13414)*;* [*https://doi.org/10.1038/s41598-018-21841-9*](https://doi.org/10.1038/s41598-018-21841-9)*;* [*https://doi.org/10.1128/mBio.02193-16*](https://doi.org/10.1128/mBio.02193-16)*).*
We are grateful for the reviewer to point out these publications, which we thus far had not included in our reference list. We carefully went through these papers and selected the following to be added to our introduction: Wrigth et al. mBio (2017) [31]; Qin et al. Sci Rep (2020) [30]; Wu et al. Nat Comm (2016) [32]. We chose to not add Ambrosi et al. 2020 and Mea et al. 2020, because we found that their focus was too specific to allow for a meaningful integration. Specifically, we write now:
*“To extend the scope beyond pre-compiled virulence factor catalogs, a diverse set of genome-wide experimental approaches have been pursued. Among others, they assessed the effect of gene knockouts on the infection process (e.g. [14, 28]), investigated transcriptional changes under conditions the bacterium encounters in the human host (e. g. [25, 29, 30]), studied adaptation evolution of bacteria inside the human host [31], and reconstructed the protein interaction network contributing to the understanding of bacterial antibiotic resistance mechanisms [32]. “*

*GC 2 – As highlighted by the authors, genomic analyses could critical pathogen interacts with its host, which substantially eases the identification of novel targets for innovative therapeutic strategies. It should be noted that he capacity of an organism to adapt via changing its spatial, structural or functional relation to its external or internal milieu. On the other hand, each environmental conditions could define the behavior of a pathogen and its adaptation.*
We are perfectly in line with the reviewer here. We are convinced that the capacity of an organism to dynamically adapt to its internal or external milieu, e.g. via adjusting its gene expression pattern or via a mutation/selection process (e.g. Wright et al. (2017)) plays an important role for understanding host-pathogen interaction. However, given the scope of our analysis we cannot reach this level of resolution. Instead, we aim at reconstructing the genetic basis on which these dynamic processes then act.

*GC 3 – As concluded by the authors their genomic analyses resulted in the hypotheses on how pathogenic Acinetobacter interact with and ultimately infect their human host. This conclusion is too ambitious without direct in vitro/in vivo investigations on AB strains upon contact with their host cells and several influential environmental conditions such as pH, osmotic stress, OMPs expression, receptor signaling pathways either during normal growth or upon in vitro and in vivo. Accordingly, several specific downstream signaling pathways are involved that enable A. baumannii to enter alveolar host cell epithelia that could be totally display differently from their genomic content, gene assembly and even genetically closed strains. These above-mentioned points should be somehow mention in this study.*

Thank you very much for pointing this out. We once again went through the manuscript and made sure that we are not too bold in our claims. In this context we would like to emphasize that we are fully aware that all of our *in-silico* analyses only result in testable hypotheses, where the Collins dictionary defines a *hypothesis as an idea which is suggested as a possible explanation for a particular situation or condition, but which has not yet been proved to be correct*. Even in the case of the Kyn-cluster, we explicitly state that it is ‘tempting to speculate’ that members of the ACB clade may interfere with the human kynurenine metabolism which has consquences for the fine-tuning of the human immune response. We are very much looking forward to see the results of the experimentalists that will hopefully soon start challenging our ideas

## Minor comments:

*MC 1 – Line 37 and Abstract: the following “They unveil, at an unprecedented resolution, the genetic makeup that coincides with the manifestation of the pathogenic phenotype in the last common ancestor of the ACB clade” is not clear. Please rephrase it in a simpler and understandable way.*

We have rephrased the sentence and trust that this makes now the information more readily available. We write now:

*“*They provide a high-resolution picture of genetic and functional changes that coincide with the manifestation of the pathogenic phenotype in the ACB clade.*”*

*MC 2 – Line 644: eight international clone types were included in this study. How and which parameters have been chosen to include them in the study. Are they selected for example, based on clonal diversity, phylogenetic index, etc?!*

We would like to clarify that we applied no filtering to include or exclude any isolates from this study apart from the sequence quality criteria that we explicitly listed in the manuscript. In essence, we took each and every *Acinetobacter* isolate from the NCBI RefSeq database that matched our quality criteria. Concerning the eight International Clone types, they represent clonal lineages in the population of *A. baumannii* whose representatives are found worldwide. We mention this now in the introduction where we write:

“Many outbreaks worldwide can be attributed to one of eight genetically well distinguishable clonal complexes within the population of *A. baumannii,* all sharing the resistance against carbapenem [9, 10].”

*MC 3 – Why pre-cultures were grown in MM with 20 mM Na-acetate?*

Na-acetate is a carbon and energy source that is very accessible to all *Acinetobacter*. Moreover, by using the same carbon source across different strains it is possible to measure and compare the lag time until a strain has adapted to a new carbon source.

*MC 4 - And what is the reson to choose Carnitine, Malate, and Glucarate as carbon source). Only because of their sugar KEGG pathway??*

We would like to draw the reviewer’s attention to our Figure 9. We chose Carnitine, Glucarate and Glucose/Gluconate for the growth experiments, because we detected the corresponding pathways to be largely confined to the pathogenic ACB clade. The intention was to show that the taxa can indeed grow on these substances. Interestingly, and we discuss this in the section “Glucose / Gluconate metabolism”, we can show that the tested strains do not grow on Glucose and Gluconate despite having the necessary enzymes encoded in their genome. We chose additionally D-Malate, because we only predicted that members of the ACB complex are in possession of a D-malate dehydrogenase, which is necessary for using D-Malate as a carbon and energy source.

*MC 5 – What is the intention to select A. baumannii, A. baylyi and A. calcoaceticus strains? Please provide strains identity.*
We apologize for having provided the information about which strain had been used only in the results section. We specify the strains now also in the Materials and Methods section: For the experiment we used the following strains: *A. baumannii* ATCC 19606, *A. baylyi* ADP1, and *A. calcoaceticus* DSM 30006. We selected strains that are established as experimental models in the lab of Dr. Beate Averhoff. We have added the strain identity now also to the Materials and Methods section. We write now:

*“A. baumannii* ATCC 19606*, A. baylyi* ADP1*, and* A. calcoaceticus *DSM 30006 strains were grown at 37 °C (A. baumannii) or 30 °C (A. baylyi and A. calcoaceticus) in mineral medium (MM)”*

*MC 6 – Are there any differences between M9 minimal medium and mineral medium (MM)!!*
Yes, there are subtle differences. Both are mineral media but using different Nitrogen sources. We would like to bring to the attention of the reviewer that the corresponding experiments have been done in two different labs, each following their own Standard Operating Procedures. We provide the information about the M9 medium in the Methods section. We write:

*“Overnight cultures were washed three times with PBS and diluted to an OD_600_ of 0.01 in 150 μl of M9 minimal medium (1X M9 salts (Becton Dickinson, cat # 248510); 2mM MgSO4; 0.1 mM CaCl2) supplemented with 0.2% casamino acids (M9CAA)…”*

*MC 7 - Where did you show the “Growth Experiments for Carnitine, Malate, and Glucarate”? these 2 simple experiments con not achieve following “they potentially interfere with the kynurenine-dependent fine-tuning of the human innate immune response” conclusion. Additional in vitro and in vivo experiments are required.*

We show the results of the growth experiments in the supplementary figure S6. Apparently, we missed to mention this in the main text for both Carnitine and Glucarate, and we are grateful for the reviewer to spot this!

Addressing the second issue: We have shown that *A. baumannii* can grow on kynurenine, as we have predicted based on the detection of the Kyn-cluster (See also our answer to SC 31 from Reviewer 1). From here on, we just describe possible implications that describe the potential that lies in this observation. We hope that the reviewer agrees with us that the phrasing ‘potentially interfere’ is sufficiently soft to make clear that we speculate here.
